# Nitrogen-to-functionalized carbon atom transmutation of pyridine†

**DOI:** 10.1039/d4sc04413d

**Published:** 2024-08-26

**Authors:** Fu-Peng Wu, Madina Lenz, Adhya Suresh, Achyut R. Gogoi, Jasper L. Tyler, Constantin G. Daniliuc, Osvaldo Gutierrez, Frank Glorius

**Affiliations:** a Organisch-Chemisches Institut, Universität Münster Corrensstraße 40 48149 Münster Germany glorius@uni-muenster.de; b Department of Chemistry, Texas A&M University 3255 TAMU, 580 Ross St 77843 College Station TX USA og.labs@tamu.edu

## Abstract

The targeted and selective replacement of a single atom in an aromatic system represents a powerful strategy for the rapid interconversion of molecular scaffolds. Herein, we report a pyridine-to-benzene transformation *via* nitrogen-to-carbon skeletal editing. This approach proceeds *via* a sequence of pyridine ring-opening, imine hydrolysis, olefination, electrocyclization, and aromatization to achieve the desired transmutation. The most notable features of this transformation are the ability to directly install a wide variety of versatile functional groups in the benzene scaffolding, including ester, ketone, amide, nitrile, and phosphate ester fragments, as well as the inclusion of *meta*-substituted pyridines which have thus far been elusive for related strategies.

## Abstract 2

The targeted and selective replacement of a single atom in an aromatic system represents a powerful strategy for the rapid interconversion of molecular scaffolds.

## Graphical abstract

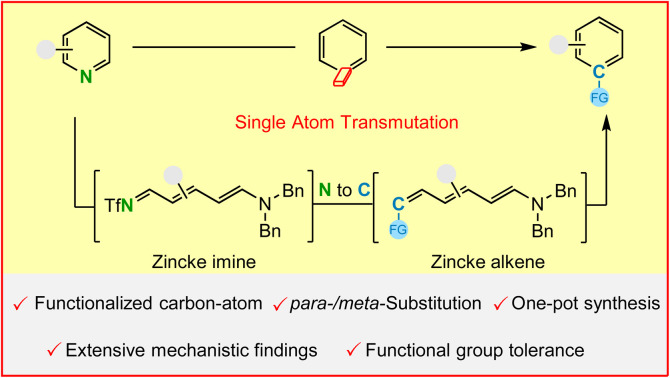

## Introduction

**Fig. 1:**
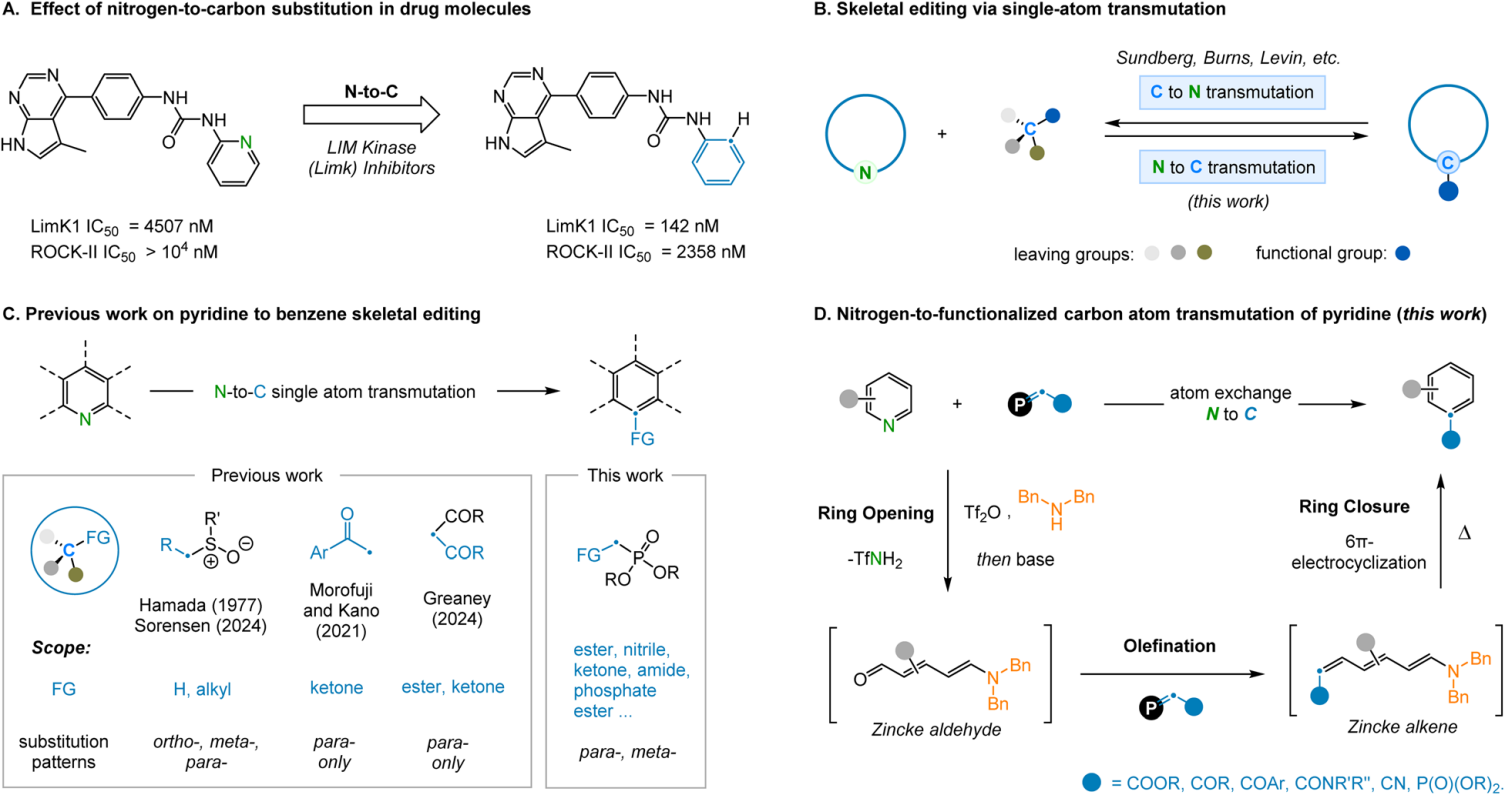
(A) Effect of nitrogen-to-carbon substitution in drug molecules. (B) Skeletal editing *via* single-atom (N/C) transmutation. (C) Previous work on pyridine to benzene skeletal editing. (D) This work: nitrogen-to-functionalized carbon atom transmutation of pyridine.

Single-atom skeletal editing has recently gained unprecedented amounts of attention as a unique strategy for drug design and development.^1^ This is most prominently demonstrated in structure–activity relationship (SAR) studies where it can be observed that replacing a single atom in the core scaffold of a compound can have an enormous effect on molecular properties.^2^ For example, the replacement of the nitrogen atom in pyridine for a carbon atom has been shown in certain systems to increase metabolic stability and bioavailability, reducing the risk of adverse side-reactions (Fig. 1A).^3^ Additionally, nitrogen-containing heterocycles, such as pyridine, represent some of the most common scaffolds in pharmaceuticals^4^ and, as a consequence, related analogues of these structures are constantly sought-after. Although the concept of atom transmutation is simple, the practical barriers to achieving this transformation are considerable, principally due to the inherent difficulty associated with arene dearomatization and cleaving strong σ-bonds. Nevertheless, single-atom skeletal editing reactions have been reported with increasing frequency over recent years.^5^ For example, transformations such as oxygen-to-nitrogen swapping,^6^ nitrogen-atom deletion^7^ and single carbon atom insertion^8^ have all been demonstrated across a variety of systems. Furthermore, the conversion of functionalized benzene rings to pyridine, *via* atom transmutation, has become established as a result of pioneering work, most notably by the groups of Sundberg,^9^ Burns,^10^ and Levin.^11^ However, our attention was drawn to the reverse of this transformation, as the extra available bond associated with the replacement of a nitrogen atom for carbon provides the potential to install additional functionality at this position (Fig. 1B). This fundamental transformation has been previously achieved *via* intramolecular rearrangements^12^ and multi-atom replacement reactions,^13^ however, single-atom transmutations^14^ are noticeably rarer.

In 1977, Hamada and Takeuchi^15^ discovered that benzo[*h*]quinoline *N*-oxide could be converted to anthracene using DMSO as the source of carbon atoms, but this strategy was found to be unsuitable for other substrates (Fig. 1C). Recently, Sorensen and coworkers improved this method by employing *n*-butyllithium, effectively enhancing conversion efficiency and expanding the substrate scope.^16*a*^ Similarly, Kano and Morofuji^17^ reported the synthesis of 4-aryl substituted benzene compounds by reacting Zincke-imines, obtained *via* pyridine ring-opening, with methyl ketone derived enolates. Although impressive, this two-step approach requires the isolation of the intermediate Zincke-imine in order to facilitate the desired electrocyclization process. In addition, the functionality installed was inherently restricted to ketones and only 4-substituted benzene products could be generated. A further recent report from Greaney and coworkers disclosed a pyridine-to-benzene transformation using malonate nucleophilic addition, although this was limited to *para*-substituted pyridines.^16*b*^ Finally, this strategy of ring-opening and ring-closing has been applied to the isotopic labelling of pyridine nitrogen atoms.^18^

Encouraged by the potential of this emerging field, we set out to identify novel ways to achieve a pyridine-to-benzene transformation *via* N-to-C atom swapping. Inspired by the work of McNally and coworkers on pyridine halogenation^19^ and the studies of Juttz and Wagner on electrocyclization,^20^ we speculated that Tf_2_O promoted pyridine ring-opening could be followed by the selective olefination of the resulting NTf-imine to access Zincke alkenes (Fig. 1D). These intermediates would be predisposed towards 6π-electrocyclization^21^ to access a single benzene isomer. In this article, we focus on the reaction pathways of *meta*-substituted electrocyclizations and the insertion of a new functional group on the newly installed carbon atom (–C_FG_). Thus, we report the successful development of a new nitrogen-to-functionalized carbon (N–C_FG_) atom reaction to convert pyridine rings to benzene analogues. Depending on the substituents appended to the olefination reagent, various functionalized benzenes can be effectively accessed using this protocol.

## Results and discussion

To validate our reaction design, 3-phenyl-pyridine was used as a model substrate, since *meta*-substituted pyridines have thus far been elusive for related synthetic strategies. We employed Tf_2_O, amine, and KO^*t*^Bu to achieve ring-opening of 3-phenyl-pyridine to afford 3-phenyl-NTf-Zincke imine intermediate 1a (Table 1).^18^ We initially observed that olefination of 1a with phosphaneylidene P1 as the carbon source provided direct access to the desired benzene product 4a in 14% yield (Table 1, entries 1–2). Unfortunately, an extensive screen of reaction solvents was unable to improve the reaction outcome (Table 1, entry 3). However, when olefination reagent P2 was employed in combination with THF, the product yield was improved to 22% (Table 1, entry 4). As well as 4a, Zincke aldehyde 2a and Zincke alkene 3a were also observed under these conditions, suggesting that the reaction mechanism likely involves imine hydrolysis followed by olefination and electrocyclization. With this in mind, we attempted to probe these individual steps in isolation in order to gain a better understanding of the transformation.

**Table 1:** Initial reaction screening^a^

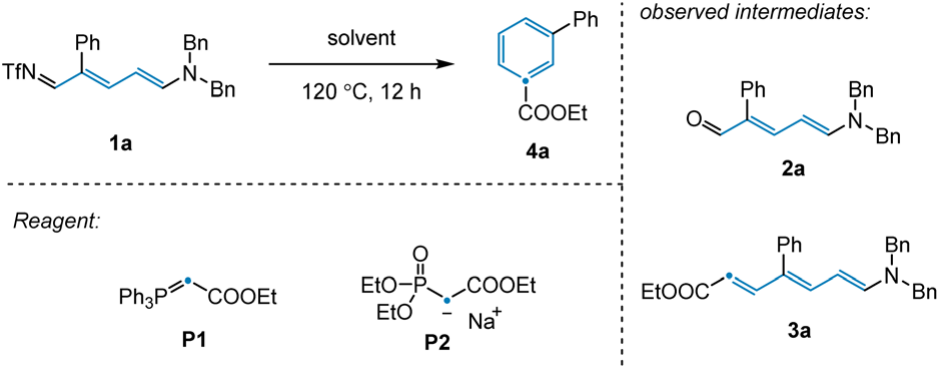			
Entry	Solvent	Reagent	4a, yield (%)
1	Toluene (r.t.)	P1	Trace
2	Toluene	P1	14
3	EtOAc, CH_2_Cl_2_, THF, DMF, MeCN, or EtOH	P1	<10
4	THF	P2	22

a Reaction conditions: Zincke imine (0.05 mmol), P1 (1.5 eq.) or P2 (1.5 eq., prepared from ethyl 2-(diethoxyphosphoryl)acetate and NaH), stirred at 120 °C for 12 h. Yields were determined by ^1^H NMR using CH_2_Br_2_ as an internal standard.

Despite being reported as an effective method for imine hydrolysis in previous transformations,^22^ silica gel was found to be entirely unsuitable in this case (Table 2, entry 1). Additionally, employing HCl and NaOH respectively, failed to access the desired aldehyde 2a (Table 2, entries 2–3). Conversely, when imine 1a was subjected to NaO^*t*^Bu and H_2_O, we observed full conversion and obtained 99% of the Zincke aldehyde (Table 2, entry 4). Subsequent control experiment showed that imine quickly decomposes in the absence of H_2_O (Table 2, entry 5). It was later discovered that hydrolysis could also be efficiently achieved after just 15 minutes upon heating the imine under basic conditions (Table 2, entry 6).

**Table 2:** Optimization of Zincke-imine hydrolysis^a^

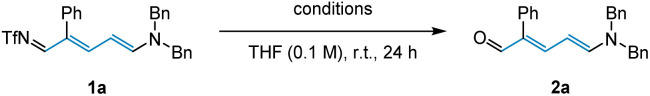			
Entry	Conditions	RSM (%)	4a, yield (%)
1	Silica gel (50 mg mL^−1^)	100	0
2	HCl (1 M)	100	0
3	NaOH (1 M)	100	0
4	NaO^*t*^Bu, H_2_O (1 : 1, 1.1 eq.)	0	99
5	NaO^*t*^Bu (1.1 eq.)	0	0
6^b^	NaO^*t*^Bu, H_2_O (1 : 1, 1.1 eq.)	0	99

a Reaction conditions: Zincke imine (0.05 mmol), base or acid, and THF (0.1 M) stirred at room temperature for 24 h.

b 120 °C for 15 min.

**Scheme 1:**
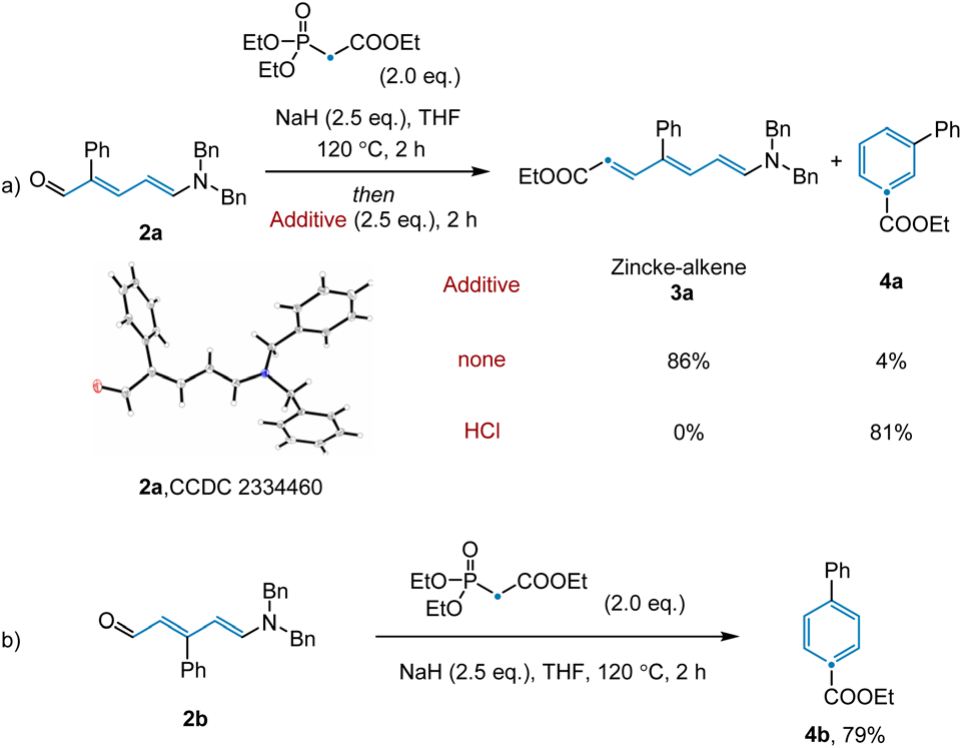
Optimization of olefination and ring closure.

Having achieved this next step of the transformation, we turned our attention to the olefination and subsequent ring closure of Zincke aldehyde 2a (Scheme 1a). Although the thermal Horner–Wadsworth–Emmons reaction (HWE) was expected to promote both of these steps, only trace desired product 4a was observed under standard olefination conditions. Upon scanning additives, we found that basic conditions inhibited electrocyclization, while acidic conditions promoted reactivity for this substrate (see ESI†). Notably, in the synthesis of *para*-substituted pyridine 4b, no additive was required to access the desired product (Scheme 1b, see below for discussion). Given that the conditions employed for the individual transformations in this sequence were relatively simple, we hypothesized that all steps, including Tf_2_O promoted pyridine ring-opening, could be combined into a convenient protocol. Indeed, this was found to be the case, with desired benzene product 4a being prepared in 70% yield from the corresponding substituted pyridine.

**Fig. 2:**
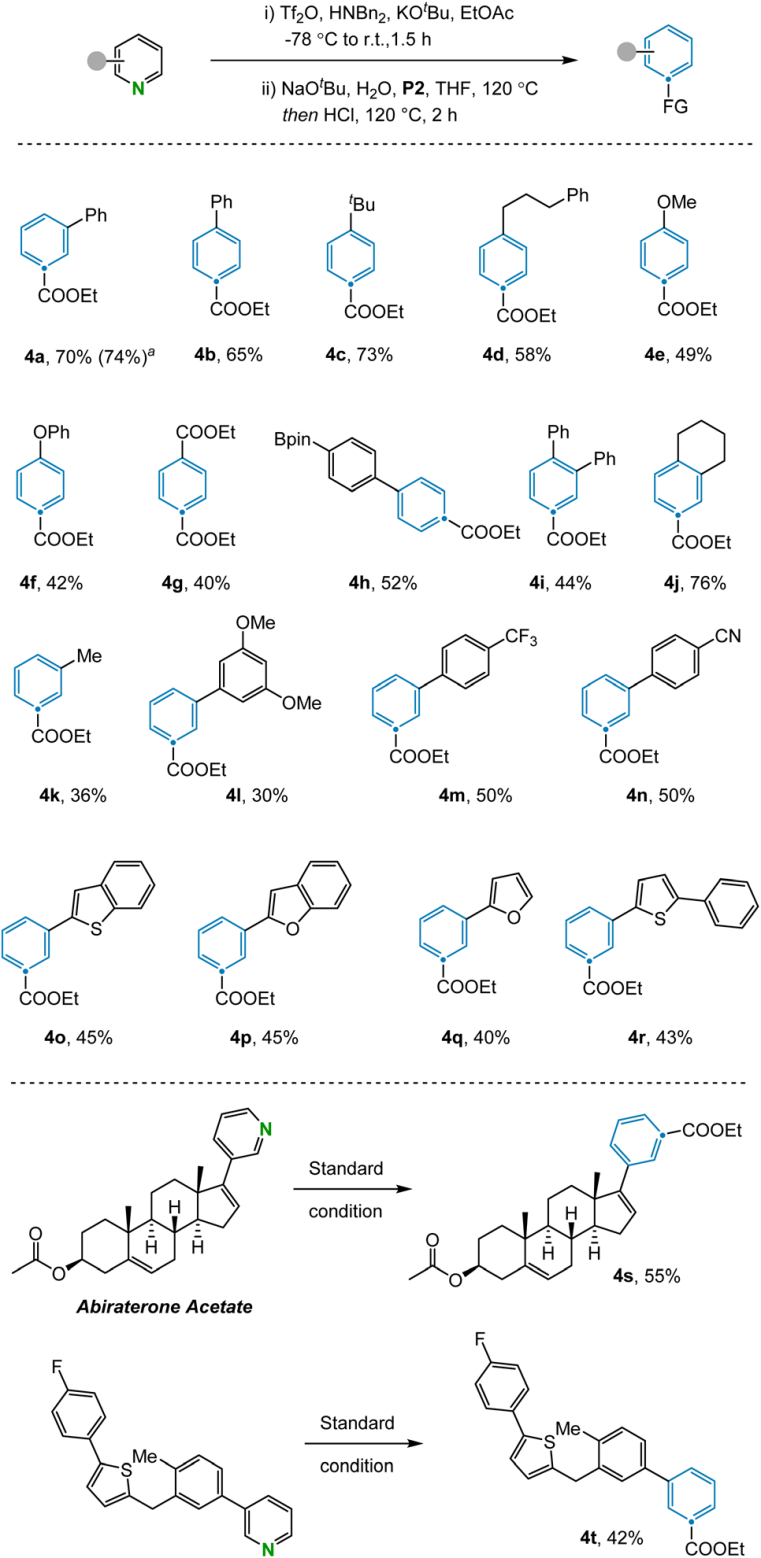
Scope of the pyridines. Standard conditions: (i) pyridine (0.2 mmol), Tf_2_O (0.24 mmol), Bn_2_NH (0.24 mmol), KO^*t*^Bu (0.24 mmol), and EtOAc (3 mL) at −78 °C to room temperature for 1.5 h. (ii) NaO^*t*^Bu (0.22 mmol), H_2_O (0.22 mmol), heated to 120 °C for 15 min, then P2 (0.4 mmol), HCl (0.72 mmol, 4 M in dioxane) and THF (3 mL), heated to 120 °C for 4 h. Isolated yields given. ^*a*^Yield determined by ^1^H NMR.

With the optimized reaction conditions in hand, the scope of the N-to-C_(CO2Et)_ atom transmutation of pyridine was explored (Fig. 2). A range of *para*-substituted pyridines bearing various substituents including aryl (4b) and alkyl groups (4c, 4d), ethers (4e, 4f), esters (4g), and boronic esters (4h), performed well in the reaction. The method could also be used to access trisubstituted benzene compounds (4i, 4j) from disubstituted pyridines in moderate to good yields. Various aromatic systems containing *meta*-substituents such as trifluoromethyl (4m) and cyano (4n) groups were compatible. Furthermore, pyridines containing heterocyclic rings like benzothiophene (4o), benzofuran (4p), furan (4q) and thiophene (4r) were efficiently converted to the corresponding benzene products. Despite the success of utilizing *meta*- and *para*-substituted pyridines, subjecting *ortho*-substituted pyridines failed to afford the desired benzene products due to the inability of the corresponding Zincke ketones to engage in the olefination reaction. Pyrimidines are not compatible with this reaction as the corresponding aza-Zincke imine intermediates undergo preferential hydrolysis of the non-terminal imine under basic conditions.^23^ In addition, some sensitive functional groups such as phenols, anilines and halogens were not tolerated under the reaction conditions (see ESI Section 4†). To illustrate the potential of the transformation in late-stage modification, we applied it to complex compounds. Abiraterone acetate and bioactive fragment molecule are readily converted to the corresponding arenes (4s, 4t) in moderate yields.

**Fig. 3:**
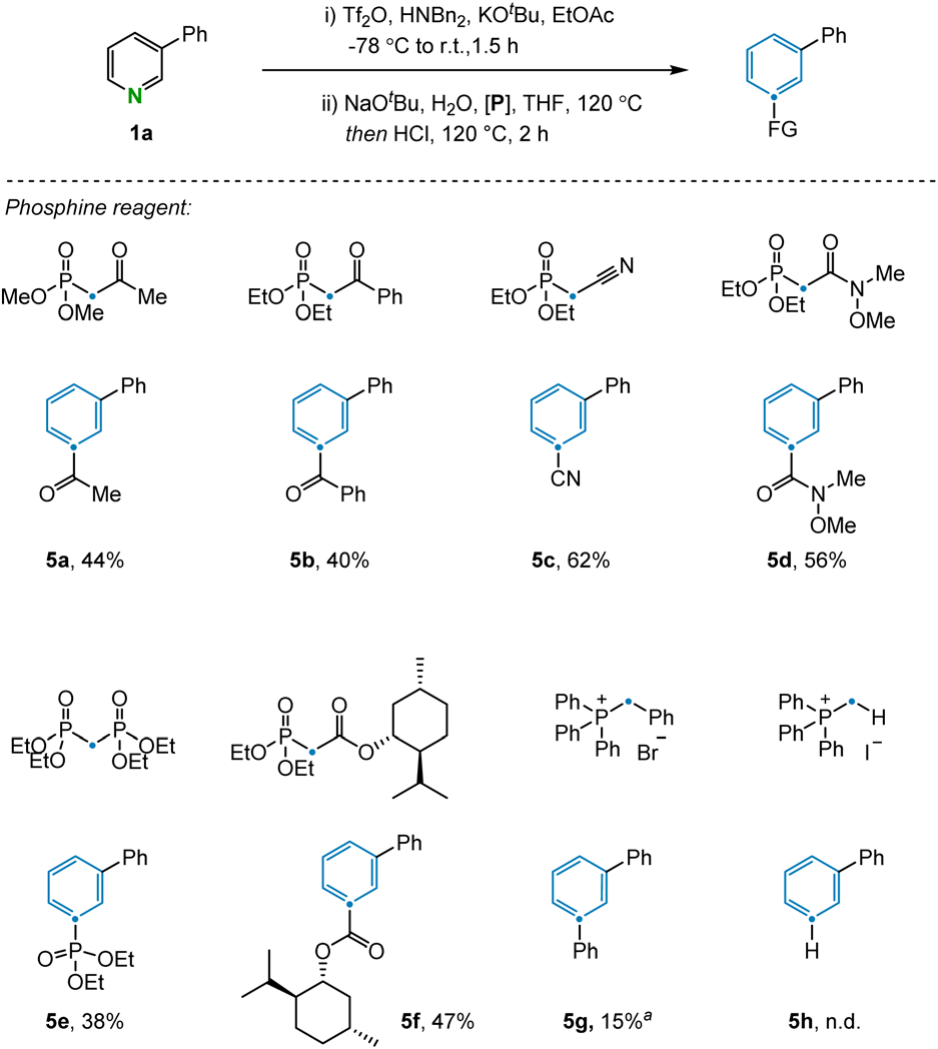
Scope of the olefination partners. Standard conditions: (i) 3-phenyl pyridine (0.2 mmol), Tf_2_O (0.24 mmol), Bn_2_NH (0.24 mmol), KO^*t*^Bu (0.24 mmol), and EtOAc (3 mL) at −78 °C to room temperature for 1.5 h. (ii) NaO^*t*^Bu (0.22 mmol), H_2_O (0.22 mmol), heated to 120 °C for 15 min, then phosphine reagent (0.4 mmol), HCl (0.72 mmol, 4 M in dioxane) and THF (3 mL), heated to 120 °C for 4 h. Isolated yields given. ^*a*^Yield determined by ^1^H NMR.

The transformation was further examined by exploring alternative olefination partners that could be used to install diverse functionality on the benzene products (Fig. 3). A range of synthetically useful functionalized carbon atoms could undergo transmutation with the pyridine nitrogen to deliver decorated benzene compounds that contain both aliphatic (5a) and aryl (5b) ketones as well as nitrile (5c), amide (5d), phosphate ester (5e), and ester (5f) functional groups. However, when the reagent benzyltriphenylphosphonium was examined under the reaction condition, only 15% yield was obtained due to incomplete conversion in the electrocyclization step (5g). The same issue was also observed during attempts to install the corresponding C–H bond in the product, with no indication of benzene formation in this case. However, transmutation of N-to-CH can still be conceptually be achieved *via* a sequence of ester hydrolysis and subsequent decarboxylation.^24^ Alternative phosphonium reagents bearing electron-donating groups were evaluated but failed to produce the desired products, presumably because electron-rich 6π-systems are not conducive to cyclization (see ESI Section 4.2†).

**Fig. 4:**
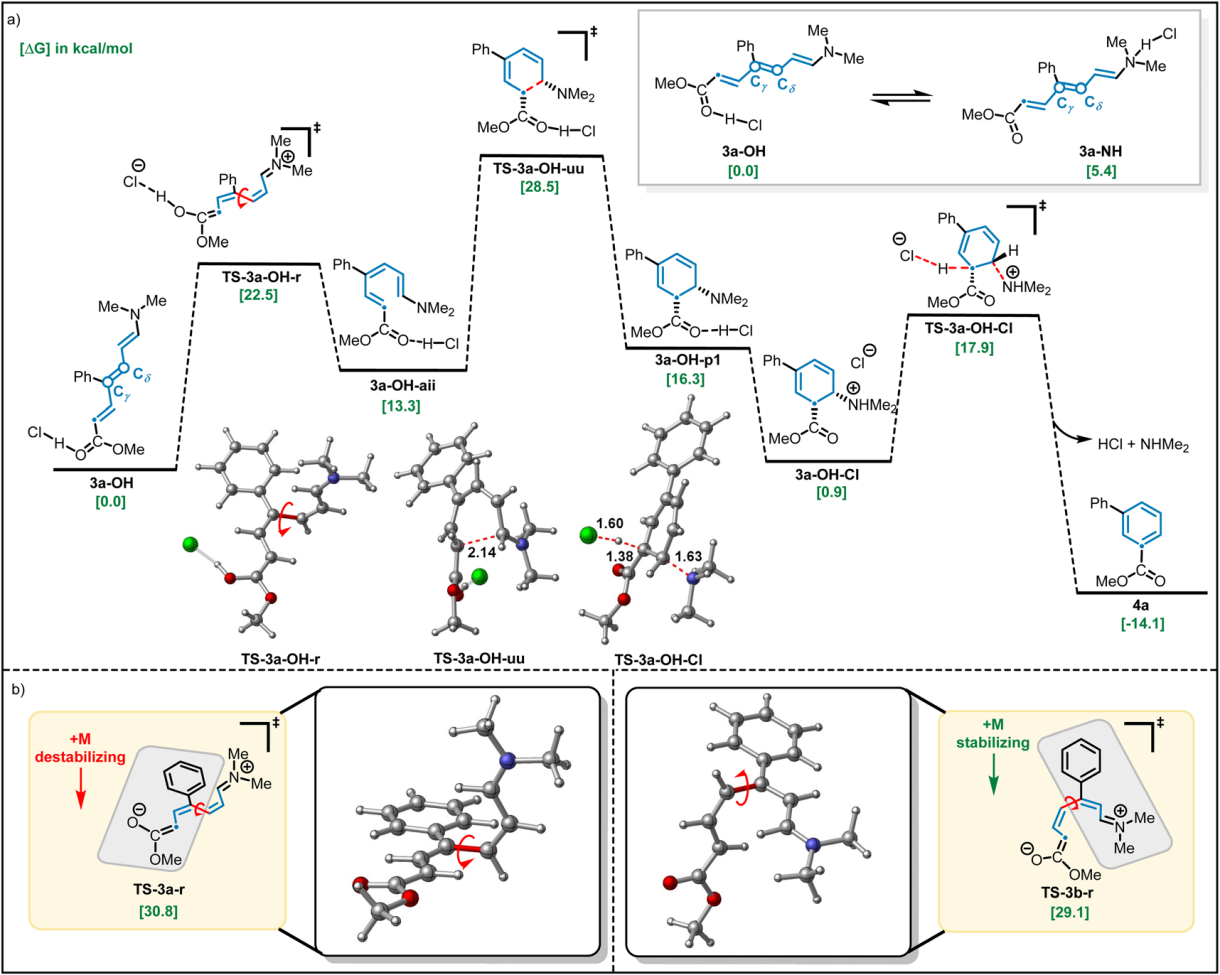
Computational studies on the impact of (a) acid additive on the barrier for isomerization-electrocyclization and (b) *para*- *vs. meta* substituents on the barriers for isomerization. All structures were calculated at the UB3LYP-D3/aug-CC-PVTZ-CPCM(THF)//UB3LYP-d3/def2-svp-CPCM(THF) level of theory. Relative (Δ*G*_rel_) energies are in kcal mol^−1^.

As discussed above (Scheme 1), we observed that the use of basic or acidic conditions had a profound effect on the reactivity of *meta*-aryl substituted pyridines while the corresponding *para*-substituted pyridine system was considerably less sensitive. To shed light on the mechanism, and particularly the effect of the substitution pattern (*meta vs. para*) and additives on reactivity, we employed dispersion-corrected density functional theory calculations (see ESI†). We initiated our studies by exploring the propensity of Zincke intermediates to undergo electrocyclization using 3a as model system. As shown in Fig. 4, our calculations suggest that under acidic conditions, carbonyl protonation (3a-OH) is significantly more energetically favored (5.4 kcal mol^−1^) over enamine-protonation (3a-NH), presumably due to the disruption of charge delocalization in 3a-NH upon protonation (see S8 and S9† for more details). Notably, upon protonation under acidic conditions, 3a-OH could rapidly undergo a C_γ_–C_δ_ bond rotation *via*TS-3a-OH-r (22.5 kcal mol^−1^) to get to the productive (3a-OH-aii) isomer required for subsequent electrocyclization (28.5 kcal mol^−1^*via*TS-3a-OH-uu). Finally, a proton transfer leads to the thermodynamically favorable 3a-OH-Cl intermediate which, after deprotonation, restores aromaticity and leads to the final product 4a.

Consistent with the lower reactivity observed for the ring-closing step in the absence of HCl (4% *vs.* 81% yield; Scheme 1), calculations show that when model Zincke intermediate 3a is not protonated, a much higher (30.8 kcal mol^−1^) barrier for the rate-limiting C_γ_–C_δ_ rotation (barrier for electrocyclization is lower, 28.6 kcal mol^−1^, than bond rotation, see Fig. S14†) is observed. These results suggest that the role of strong acid is to catalyze the C_γ_–C_δ_ isomerization and, in turn, allow for the formation of the productive isomer to undergo electrocyclization. Furthermore, under basic conditions, *meta*-substituted phenyl pyridine is observed to give a much lower yield when compared to the analogous *para*-substituted pyridine (4% *vs.* 79% respectively, see Scheme 1). Consistent with the experiments, dispersion-corrected DFT calculations predict that under basic conditions, the rate for C_γ_–C_δ_ rotation (see Fig. S14 and S15† for the full profile) is ∼10× faster (*i.e.*, 1.7 kcal mol^−1^ lower in energy) for *para*-substituted pyridine (3b) as compared to *meta*-substituted pyridine (3a) (see Fig. 4b). This lower barrier is attributed to the positive mesomeric effect (+M effect) of the phenyl ring which destabilizes the C_γ_–C_δ_ rotation transition state of 3a because the phenyl ring is coplanar with the ester group predominantly containing negative charge (TS-3a-r; Fig. 4b). In contrast, the positive mesomeric effect (+M effect) of the phenyl ring stabilizes the C_γ_–C_δ_ rotation transition state of 3b (TS-3b-r) as the phenyl ring is coplanar with the amine moiety predominantly containing positive charge. We sought to validate this hypothesis by theoretically introducing electron-donating as well as electron-withdrawing groups at the *para*-position of the phenyl ring that would create a strong +M or −M effect. The predicted barriers for the *para*-substituted substrates support our hypothesis about the crucial role played by the +M effect of the phenyl ring (see Fig. S16† for a detailed analysis).

## Conclusions

In summary, we have developed a convenient N-to-C single-atom transmutation reaction to convert pyridines to benzenes. This method exhibits remarkable functional group tolerance and could effectively convert both *para*- and *meta*-substituted pyridines. This strategy also allows a variety of functionalized carbon atoms to be installed in place of nitrogen to directly edit the molecular scaffold of these heterocycles. The DFT results reveal the effect of acidic or basic conditions on the electrocyclization of *para*- and *meta*-substituents. We anticipate that this strategy will provide new avenues for single-atom transmutation as a strategy to directly manipulate the core of heteroaromatic scaffolds.

## Author contributions

F. G. and F.-P. W. conceived the project. F.-P. W. performed the initial screening experiments. F.-P. W. and M. L. performed synthetic experiments. A. S. and A. R. G. conducted computations. C. G. D. analysed X-ray structures. F.-P. W. and F. G. supervised research. F.-P. W., J. L. T., O. G. and F. G. wrote the manuscript with contributions from all authors.

## Conflicts of interest

There are no conflicts to declare.

## Supplementary Material

SC-015-D4SC04413D-s001

SC-015-D4SC04413D-s002

## Data Availability

General information, detailed experimental procedures, characterization data for compounds, and NMR, HPLC spectra are available in the ESI.†

## References

[cit1] Jurczyk J., Woo J., Kim S. F., Dherange B. D., Sarpong R., Levin M. D. (2022). Nat. Synth..

[cit2] Meanwell N. A. (2011). J. Med. Chem..

[cit3] Yin Y., Zheng K., Eid N., Howard S., Jeong J. H., Yi F., Guo J., Park C. M., Bibian M., Wu W., Hernandez P., Park H., Wu Y., Luo J. L., LoGrasso P. V., Feng Y. (2015). J. Med. Chem..

[cit4] Mermer A., Keles T., Sirin Y. (2021). Bioorg. Chem..

[cit5] Qiu X., Sang Y., Wu H., Xue X. S., Yan Z., Wang Y., Cheng Z., Wang X., Tan H., Song S., Zhang G., Zhang X., Houk K. N., Jiao N. (2021). Nature.

[cit6] Lyu H., Kevlishvili I., Yu X., Liu P., Dong G. (2021). Science.

[cit7] Kennedy S. H., Dherange B. D., Berger K. J., Levin M. D. (2021). Nature.

[cit8] Ma D., Martin B. S., Gallagher K. S., Saito T., Dai M. (2021). J. Am. Chem. Soc..

[cit9] Sundberg R. J., Suter S. R., Brenner M. (1972). J. Am. Chem. Soc..

[cit10] Patel S. C., Burns N. Z. (2022). J. Am. Chem. Soc..

[cit11] Pearson T. J., Shimazumi R., Driscoll J. L., Dherange B. D., Park D.-I., Levin M. D. (2023). Science.

[cit12] Sagitullin R. S., Gromov S. P., Kost A. N. (1978). Tetrahedron.

[cit13] Cheng Q., Bhattacharya D., Haring M., Cao H., Muck-Lichtenfeld C., Studer A. (2024). Nat. Chem..

[cit14] Fout A. R., Bailey B. C., Tomaszewski J., Mindiola D. J. (2007). J. Am. Chem. Soc..

[cit15] Hamada Y., Takeuchi I. (1977). J. Org. Chem..

[cit16] Falcone N. A., He S., Hoskin J. F., Mangat S., Sorensen E. J. (2024). Org. Lett..

[cit17] Morofuji T., Kinoshita H., Kano N. (2019). Chem. Commun..

[cit18] Feng M., Norlöff M., Guichard B., Kealey S., D'Anfray T., Thuéry P., Taran F., Gee A., Feuillastre S., Audisio D. (2024). Nat. Commun..

[cit19] Boyle B. T., Levy J. N., Lescure L. d., Paton R. S., McNally A. (2022). Science.

[cit20] Jutz C., Wagner R. M. (1972). Angew. Chem., Int. Ed..

[cit21] Yu T.-Q., Fu Y., Liu L., Guo Q.-X. (2006). J. Org. Chem..

[cit22] Ito S., Yokoyama R., Okujima T., Terazono T., Kubo T., Tajiri A., Watanabe M., Morita N. (2003). Org. Biomol. Chem..

[cit23] Uhlenbruck B. J. H., Josephitis C. M., Lescure L. d., Paton R. S., McNally A. (2024). Nature.

[cit24] Gooßen L. J., Linder C., Rodríguez N., Lange P. P., Fromm A. (2009). Chem. Commun..

