# Supplementary material for: Nitrogen-to-functionalized carbon atom transmutation of pyridine
Source: Chem Sci. 2024 Aug 26;15(37):15205–11. doi: 10.1039/d4sc04413d (PMC11372446; doi:10.1039/d4sc04413d)

Supplementary Information (SI) for Chemical Science.
This journal is © The Royal Society of Chemistry 2024

Nitrogen-to-functionalized carbon atom transmutation of pyridine

Fu-Peng Wu,[1] Madina Lenz,[1] Adhya Suresh[2,†], Achyut R. Gogoi[2,†], Jasper L. Tyler,[1] Constantin G. Daniliuc,[1] Osvaldo Gutierrez[2,*], and Frank Glorius[1,*]

[1]Organisch-Chemisches Institut, Universität Münster, Münster, Germany

[2]Department of Chemistry, Texas A&M University, College Station, Texas United States

*Correspondence to: glorius@uni-muenster.de; og.labs@tamu.edu

Contents

1. General information........S3

1.1 General remarks........S3

1.2 Analytical techniques and compound purification........S3

2. Reaction development........S4

2.1 Optimization of the reaction conditions........S4

2.2. General procedure for the pyridine-to-benzene reactions........S7

4. Limitations........S20

5. X-Ray analysis........S22

6. DFT calculation........S24

7. Reference........S135

8. Copies of ^{1}H, ^{13}C and ^{19}F NMR spectra........S137

1. General information

1.1 General remarks

All reagents were purchased from Alfa Aesar, Sigma-Aldrich, Merck, TCI, Fluorochem, Combi-blocks, VWR and used without further purification, except otherwise stated. All the solvents were bought from Acros in AcroSeal® bottles and were directly stored under 3 or 4 Å molecular sieves, replacing the collected volume with argon. All reactions were carried out in an oven-dried glassware under an atmosphere of argon using standard Schlenk technique, unless otherwise noted. Solvents for chromatographic purification (pentane, EtOAc, dichloromethane and methanol) were purchased as technical grade and purified by atmospheric pressure distillation. Reaction temperatures are referred to the temperature of the heating medium, unless otherwise stated.

1.2 Analytical techniques and compound purification

NMR-spectra were recorded on a Bruker Avance II 300, Avance II 400, Agilent DD2 500 or DD2 600 spectrometers. All spectral data was acquired at 295 K, except otherwise stated. Deuterated solvents were purchased from Eurisotop ($CDCl_3$, deuteration > 99.8%). ^{1}H and ^{13}C chemicals shifts (δ) are quoted in parts per million (ppm) against tetramethylsilane (TMS, δ = 0.00 ppm) and were internally referenced to residual $CHCl_3$ (7.26 ppm for 1H, 77.0 ppm for ^{13}C). Coupling constants (J) are reported in Hertz (Hz) to the nearest 0.1 Hz. The following abbreviations (or combinations thereof) were used to explain multiplicities: s = singlet, d = doublet, t = triplet, m = multiplet. High-resolution mass spectra (HRMS) were obtained by the MS service of the Organisch-Chemisches Institut, Universität Münster, using 4 electrospray ionisation (ESI) on a Bruker Daltonics, MicroToF spectrometer and calibrated using formate ion clusters. Thin layer chromatography was carried out on Merck silica gel 60 F254 pre-coated aluminium sheets and were visualized using UV light (254 nm) and stained with basic aqueous potassium permanganate. Flash chromatography was carried out using silica gel (Acros Organics, 0.035-0.070 mm, 60 Å) under a light positive pressure of argon, eluting with the specified solvent system as mentioned.

2. Reaction development

2.1 Optimization of the reaction conditions

Table 1. Ring open of pyridine.

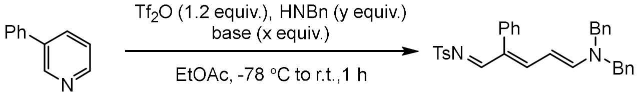

Entry	Base	x	y	RSM (%)	Yield (%)
1	Collidine	1.0	1.2	15	82
2	$NaHCO_3$	1.0	1.2	75	22
3	K_2CO_3	1.0	1.2	43	30
4	NaO^{t}Bu	1.0	1.2	37	56
5	KO^{t}Bu	1.0	1.2	26	57
6	KO^{t}Bu	1.0	1.0	21	71
7	KO^{t}Bu	1.2	1.2	12	80

Table 2. Screening of solvent for N-to-C from zincke-imine

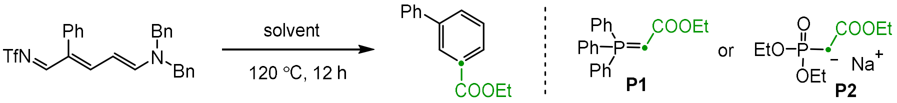

entry	solvent	reagent	yield (%)
1	Toluene	**P1**	14
2	CH_2Cl_2	**P1**	trace
3	THF	**P1**	trace
4	DMF	**P1**	trace
5	EtOAc	**P1**	9
6	CH_3CN	**P1**	0
7	EtOH	**P1**	0
8	Toluene (r.t.)	**P1**	trace
9	CH_2Cl_2	**P2**	20
10	THF	**P2**	22

Reaction conditions: Zincke imine (0.05 mmol), **P1** (1.5 eq.) or **P2** (1.5 eq., prepared from ethyl 2-(diethoxyphosphoryl)acetate and NaH), stirred at 120 °C for 12 h.

Table 3. Screening of conditions for hydrolysis step

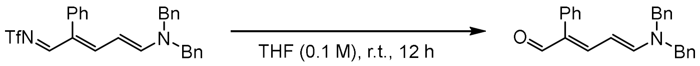

Entry	Conditions	RSM (%)	Yield (%)
1	HCl (1 M)	100	0
2	NaOH (1 M)	100	0
3	Silic gel (50 mg/mL)	100	0
4	NaOH/MeOH (1:1, 1 M)	0	0
5	110 °C instead of r.t.	100	0
6	NaO^{t}Bu (1.0 equiv.), 120 °C	0	0
7	NaO^{t}Bu (2.0 equiv.), H_2O (1.0 equiv.) 120 °C	0	99

Table 4. Screening of amount of base, temperature, and time for hydrolysis step

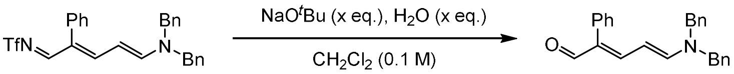

Entry	X (equiv.)	Temperature	Time	Yield (%)
1	2.0	120	15 min	99
2	1.5	120	15 min	99
3	1.2	120	15 min	95
4	1.0	120	15 min	99
9	2.0	r.t.	24 h	98

Table 5. Screening of olefination reagent

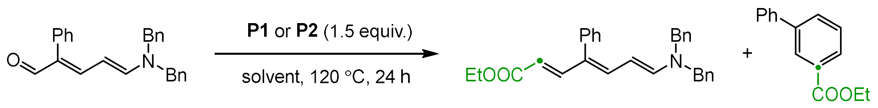

Entry	Reagent	Solvent	RSM	Alkene	Yield (%)
1	**P2**	THF	5	86	4
2	**P2**	THF/DCM	29	40	9
3	**P2**	DCM	48	6	14
4	**P2** (140 °C)	DCM	64	4	17
5	**P2** (2.0 eq.)	THF	0	94	4
6	**P1**	DCM	74	17	9

Table 6. Screening of additives for ring closure step

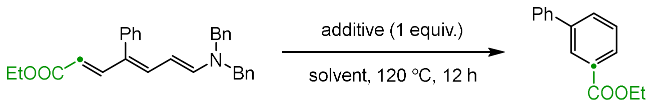

Entry	Additive	solvent	Temp. (°C)	RSM (%)	Yield (%)
1	-	THF	120	30	64
2	NaO^{t}Bu	THF	120	96	4
3	AcOH	THF	120	52	41
4	HCl (cat)	THF	120	4	94
5	TFA	Toluene	120	0	34
6	-	Toluene	120	31	58
7	-	CH_2Cl_2	120	0	86
8	-	EtOAc	120	49	44

2.2. General procedure for the pyridine-to-benzene reactions

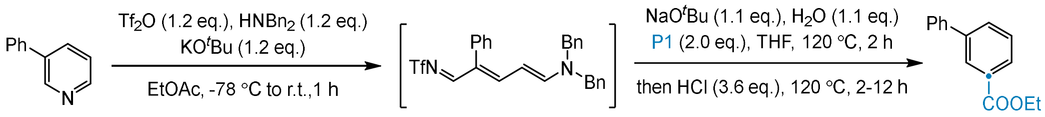

To an oven-dried 10 mL Schlenk tube equipped with a PTFE-coated oval stirring bar was evacuated and re-filled with argon four times, then the vessel was charged with the heterocycle (0.2 mmol) under an argon atmosphere. EtOAc (2 mL, 0.1 M) was added, the reaction vessel cooled to –78 ºC and Tf_2O (41 uL, 1.2 equiv.) was added dropwise over 5 minutes. The reaction was stirred for 30 minutes before dibenzylamine (46 uL, 1.2 equiv.) was added dropwise as a solution (1.0 M in EtOAc) followed by KO*t*Bu(27 mg, 1.2 equiv.). The reaction was stirred for a further 30 minutes at –78 ºC. The cooling bath was removed and the reaction was allowed to warm to room temperature while stirring for approximately 30 minutes. The mixture filter over a short plug of silica (1 cm) and the volatiles were removed under reduced pressure to give crude Zincke imines.

A new 10 mL Schlenk tube equipped with a PTFE-coated oval stirring bar was charged with NaO*t*Bu (21.1 mg, 1.1 equiv.), H_2O (4 uL, 1.1 equiv.) and THF (1 mL, 0.2 M). The crude Zincke imines was added as a solution (0.2 M in THF, 1 mL) and the reaction was stirred 10 minutes at 120 °C. After cooling, phosphonate carbanion (2.0 equiv., the phosphonate carbanion reagent prepared from ethyl 2-(diethoxyphosphoryl)acetate (2.0 equiv.) and NaH (2.5 equiv.) in THF (1 mL) at 0 ºC.) was added as a solution (1 mL, 0.4 M in THF), and the reaction continued for an additional 2 hours at 120 °C. Upon cooling to room temperature once again, HCl (180 uL, 3.6 equiv., 4 M in dioxane) was added, and the reaction was stirred for an additional 2 hours at 120 °C. After reaction complete, the resulting solution was transferred to a 25 mL round bottom flask and volatiles were removed under reduced pressure. Purification by flash column chromatography on SiO_2 (pentane/EtOAc) afforded the corresponding products.

3. Experimental data for the described substances

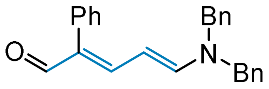

5-(Dibenzylamino)-2-phenylpenta-2,4-dienal (2a)

To an oven-dried 10 mL Schlenk tube equipped with a PTFE-coated oval stirring bar was evacuated and re-filled with argon four times, then the vessel was charged with the heterocycle (0.2 mmol) under an argon atmosphere. EtOAc (2 mL, 0.1 M) was added, the reaction vessel cooled to –78 °C and Tf_2O (41 uL, 1.2 equiv.) was added dropwise over 5 minutes. The reaction was stirred for 30 minutes before dibenzylamine (46 uL, 1.2 equiv.) was added dropwise as a solution (1.0 M in EtOAc) followed by KO^{t}Bu(27 mg, 1.2 equiv.). The reaction was stirred for a further 30 minutes at –78 °C. The cooling bath was removed and the reaction was allowed to warm to room temperature while stirring for approximately 30 minutes. The mixture filter over a short plug of silica (1 cm) and the volatiles were removed under reduced pressure to give crude Zincke imines. A new 10 mL Schlenk tube equipped with a PTFE-coated oval stirring bar was charged with NaO^{t}Bu (21.1 mg, 1.1 equiv.), H_2O (4 uL, 1.1 equiv.) and THF (1 mL, 0.2 M). The crude Zincke imines was added as a solution (0.2 M in THF, 1 mL) and the reaction was stirred 10 minutes at 120 °C. After reaction complete, the resulting solution was transferred to a 25 mL round bottom flask and volatiles were removed under reduced pressure. Purification by flash column chromatography on SiO_2 (pentane/EtOAc, 3:1 v/v, Rf = 0.4) afforded the corresponding products **2a** as yellow solid (56.5 mg, 80% yield). **X-ray** (single-crystal) Yellow block crystals of x-ray diffraction quality was obtained by slow evaporation of saturated solution of **2a** in EtOAc/n-pentane (CCDC 2334460).

^{1}H NMR (400 MHz, $CDCl_3$) δ 9.38 (s, 1H), 7.41 – 7.27 (m, 9H), 7.16 (ddd, J = 8.9, 4.9, 2.3 Hz, 7H), 7.07 (d, J = 11.9 Hz, 1H), 5.73 – 5.56 (m, 1H), 4.32 (s, 4H).

^{13}C NMR (101 MHz, $CDCl_3$) δ 191.2, 153.8, 151.9, 135.8, 134.6, 131.0, 129.7, 128.9, 128.1, 128.0, 127.5, 126.7, 97.1, 65.7.

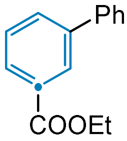

Ethyl [1,1'-biphenyl]-3-carboxylate (4a)

Prepared according to general procedure using 3-phenyl pyridine (29 uL, 0.2 mmol), ethyl 2-(diethoxyphosphoryl)acetate (80 uL, 2.0 equiv.) were used. Purification by column chromatography using pre-basified silica with pentane/EtOAc (50:1 v/v, Rf = 0.4) as eluent afforded **4a** as colorless oil (31.7 mg, 70% yield).

^{1}H NMR (400 MHz, CDCl$_3$) δ 8.31 (td, *J* = 1.9, 0.5 Hz, 1H), 8.05 (ddd, *J* = 7.8, 1.7, 1.2 Hz, 1H), 7.79 (ddd, *J* = 7.8, 2.0, 1.2 Hz, 1H), 7.67 – 7.61 (m, 2H), 7.55 – 7.44 (m, 3H), 7.42 – 7.36 (m, 1H), 4.43 (q, *J* = 7.1 Hz, 2H), 1.43 (t, *J* = 7.1 Hz, 3H).

^{13}C NMR (101 MHz, CDCl$_3$) δ 166.5, 141.4, 140.2, 131.4, 131.0, 128.8, 128.8, 128.3, 128.2, 127.7, 127.1, 61.0, 14.3.

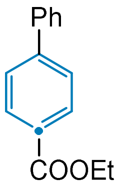

Ethyl [1,1'-biphenyl]-4-carboxylate (4b)

Prepared according to general procedure using 4-phenylpyridine (31.0 mg, 0.2 mmol), ethyl 2-(diethoxyphosphoryl)acetate (80 uL, 2.0 equiv.) were used. Purification by column chromatography using pre-basified silica with pentane/EtOAc (50:1 v/v, Rf = 0.4) as eluent afforded **4b** as white solid (29.2 mg, 65% yield).

^{1}H NMR (400 MHz, CDCl$_3$) δ 8.17 – 8.07 (m, 2H), 7.70 – 7.59 (m, 4H), 7.47 (ddd, *J* = 7.8, 6.4, 1.3 Hz, 2H), 7.43 – 7.36 (m, 1H), 4.41 (q, *J* = 7.1 Hz, 2H), 1.42 (t, *J* = 7.1 Hz, 3H).

^{13}C NMR (101 MHz, CDCl$_3$) δ 166.5, 145.5, 140.1, 130.0, 129.3, 128.9, 128.1, 127.3, 127.0, 60.9, 14.3.

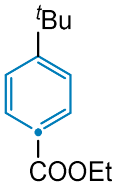

Ethyl 4-(*tert*-butyl)benzoate (4c)

Prepared according to general procedure using 4-(*tert*-butyl)pyridine (29 uL, 0.2 mmol), ethyl 2-(diethoxyphosphoryl)acetate (80 uL, 2.0 equiv.) were used. Purification by column chromatography using pre-basified silica with pentane/EtOAc (50:1 v/v, Rf = 0.4) as eluent afforded **4c** as colorless oil (30.0 mg, 73% yield).

^{1}H NMR (400 MHz, CDCl$_3$) δ 8.01 – 7.94 (m, 2H), 7.48 – 7.42 (m, 2H), 4.37 (q, *J* = 7.1 Hz, 2H), 1.39 (t, *J* = 7.1 Hz, 3H), 1.34 (s, 9H).

^{13}C NMR (101 MHz, CDCl$_3$) δ 166.6, 156.4, 129.4, 127.7, 125.3, 60.7, 35.0, 31.1, 14.3.

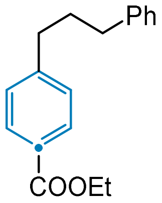

Ethyl 4-(3-phenylpropyl)benzoate (4d)

Prepared according to general procedure using 4-(3-phenylpropyl)pyridine (38 uL, 0.2 mmol), ethyl 2-(diethoxyphosphoryl)acetate (80 uL, 2.0 equiv.) were used. Purification by column chromatography using pre-basified silica with pentane/EtOAc (50:1 v/v, Rf = 0.4) as eluent afforded **5d** as colorless oil (31.3 mg, 58% yield).

^{1}H NMR (400 MHz, $CDCl_3$) δ 8.03 – 7.88 (m, 2H), 7.33 – 7.14 (m, 7H), 4.35 (q, J = 7.1 Hz, 2H), 2.66 (dt, J = 20.4, 7.7 Hz, 4H), 2.04 – 1.91 (m, 2H), 1.38 (t, J = 7.1 Hz, 3H).

^{13}C NMR (101 MHz, $CDCl_3$) δ 166.7, 147.7, 141.9, 129.6, 128.4, 128.3, 128.1, 125.8, 60.7, 35.4, 35.3, 32.6, 14.3.

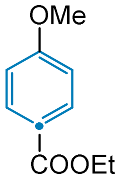

Ethyl 4-methoxybenzoate (4e)

Prepared according to general procedure using 4-methoxypyridine (20 uL, 0.2 mmol), ethyl 2-(diethoxyphosphoryl)acetate (80 uL, 2.0 equiv.) were used. Purification by column chromatography using pre-basified silica with pentane/EtOAc (20:1 v/v, Rf = 0.45) as eluent afforded **4e** as colorless oil (17.6 mg, 49% yield).

^{1}H NMR (400 MHz, $CDCl_3$) δ 8.04 – 7.95 (m, 2H), 6.96 – 6.88 (m, 2H), 4.35 (q, J = 7.1 Hz, 2H), 3.86 (s, 3H), 1.38 (t, J = 7.1 Hz, 3H).

^{13}C NMR (101 MHz, $CDCl_3$) δ 166.4, 163.2, 131.5, 123.0, 113.5, 60.6, 55.4, 14.4.

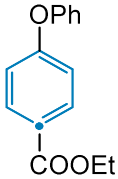

Ethyl 4-phenoxybenzoate (4f)

Prepared according to general procedure using 4-phenoxypyridine (34.2 mg, 0.2 mmol), ethyl 2-(diethoxyphosphoryl)acetate (80 uL, 2.0 equiv.) were used. Purification by column chromatography using pre-basified silica with pentane/EtOAc (50:1 v/v, Rf = 0.5) as eluent afforded **4f** as colorless oil (20.2 mg, 42% yield).

^{1}H NMR (400 MHz, CDCl$_3$) δ 8.05 – 7.97 (m, 2H), 7.43 – 7.35 (m, 2H), 7.22 – 7.15 (m, 1H), 7.09 – 7.02 (m, 2H), 7.02 – 6.95 (m, 2H), 4.36 (q, *J* = 7.1 Hz, 2H), 1.38 (t, *J* = 7.1 Hz, 3H).

^{13}C NMR (101 MHz, CDCl$_3$) δ 166.1, 161.7, 155.7, 131.6, 130.0, 124.9, 124.4, 120.0, 117.3, 60.8, 14.3.

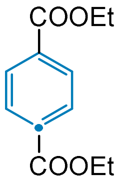

Diethyl terephthalate (4g)

Prepared according to general procedure using ethyl isonicotinate (30.2 mg, 0.2 mmol), ethyl 2-(diethoxyphosphoryl)acetate (80 uL, 2.0 equiv.) were used. Purification by column chromatography using pre-basified silica with pentane/EtOAc (20:1 v/v, Rf = 0.4) as eluent afforded **4g** as white solid (16.5 mg, 40% yield).

^{1}H NMR (400 MHz, CDCl$_3$) δ 8.10 (s, 4H), 4.41 (q, *J* = 7.1 Hz, 4H), 1.41 (t, *J* = 7.1 Hz, 6H).

^{13}C NMR (101 MHz, CDCl$_3$) δ 165.9, 134.2, 129.5, 61.4, 14.3.

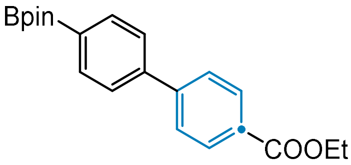

Ethyl 4'-(4,4,5,5-tetramethyl-1,3,2-dioxaborolan-2-yl)-[1,1'-biphenyl]-4-carboxylate (4h)

Prepared according to general procedure using 4-(4-(4,4,5,5-tetramethyl-1,3,2-dioxaborolan-2-yl)phenyl)pyridine (56.2 uL, 0.2 mmol), ethyl 2-(diethoxyphosphoryl)acetate (80 uL, 2.0 equiv.) were used. Purification by column chromatography using pre-basified silica with pentane/EtOAc (10:1 v/v, Rf = 0.35) as eluent afforded **4h** as yellow solid (36.4 mg, 52% yield).

^{1}H NMR (400 MHz, CDCl$_3$) δ 8.15 – 8.08 (m, 2H), 7.95 – 7.87 (m, 2H), 7.71 – 7.65 (m, 2H), 7.65 – 7.59 (m, 2H), 4.41 (q, *J* = 7.1 Hz, 2H), 1.42 (t, *J* = 7.1 Hz, 3H), 1.37 (s, 12H).

^{13}C NMR (101 MHz, CDCl$_3$) δ 166.4, 145.3, 142.6, 135.3, 130.0, 129.5, 127.1, 126.5, 83.9, 60.9, 24.9, 14.3.

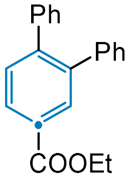

Ethyl [1,1':2',1''-terphenyl]-4'-carboxylate (4i)

Prepared according to general procedure using 3,4-diphenylpyridine (46.2 mg, 0.2 mmol), ethyl 2-(diethoxyphosphoryl)acetate (80 uL, 2.0 equiv.) were used. Purification by column chromatography using pre-basified silica with pentane/EtOAc (50:1 v/v, Rf = 0.4) as eluent afforded **4i** as yellow oil (26.4 mg, 44% yield).

^{1}H NMR (400 MHz, $CDCl_3$) δ 8.12 (d, *J* = 1.7 Hz, 1H), 8.08 (dd, *J* = 8.0, 1.9 Hz, 1H), 7.50 (d, *J* = 8.0 Hz, 1H), 7.23 (dd, *J* = 4.7, 1.6 Hz, 6H), 7.19 – 7.11 (m, 4H), 4.42 (q, *J* = 7.1 Hz, 2H), 1.42 (t, *J* = 7.1 Hz, 3H).

^{13}C NMR (101 MHz, $CDCl_3$) δ 166.4, 144.9, 140.7, 140.6, 140.6, 131.7, 130.7, 129.8, 129.7, 129.6, 128.5, 128.0, 127.1, 126.8, 61.0, 14.4.

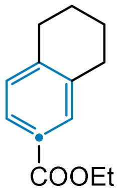

Ethyl 5,6,7,8-tetrahydronaphthalene-2-carboxylate (4j)

Prepared according to general procedure using 5,6,7,8-tetrahydroisoquinoline (26 uL, 0.2 mmol), ethyl 2-(diethoxyphosphoryl)acetate (80 uL, 2.0 equiv.) were used. Purification by column chromatography using pre-basified silica with pentane/EtOAc (50:1 v/v, Rf = 0.4) as eluent afforded **4j** as yellow oil (31.1 mg, 76% yield).

^{1}H NMR (400 MHz, $CDCl_3$) δ 7.77 – 7.70 (m, 2H), 7.13 – 7.08 (m, 1H), 4.35 (q, *J* = 7.1 Hz, 2H), 2.81 (dq, *J* = 6.7, 3.1 Hz, 4H), 1.81 (ddd, *J* = 6.7, 4.1, 2.9 Hz, 4H), 1.38 (t, *J* = 7.1 Hz, 3H).

^{13}C NMR (101 MHz, $CDCl_3$) δ 166.9, 142.7, 137.2, 130.3, 129.1, 127.6, 126.5, 60.7, 29.6, 29.3, 23.0, 22.9, 14.3.

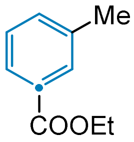

Ethyl 3-methylbenzoate (4k)

Prepared according to general procedure using 3-methylpyridine (20 uL, 0.2 mmol), ethyl 2-(diethoxyphosphoryl)acetate (80 uL, 2.0 equiv.) were used. Purification by column chromatography using pre-basified silica with pentane/EtOAc (50:1 v/v, Rf = 0.4) as eluent afforded **4k** as colorless oil (11.8 mg, 36% yield).

^{1}H NMR (400 MHz, $CDCl_3$) δ 7.91 – 7.78 (m, 2H), 7.41 – 7.28 (m, 2H), 4.37 (q, *J* = 7.1 Hz, 2H), 2.40 (d, *J* = 0.8 Hz, 3H), 1.39 (t, *J* = 7.1 Hz, 3H).

^{13}C NMR (101 MHz, $CDCl_3$) δ 166.8, 138.0, 133.5, 130.4, 130.0, 128.2, 126.6, 60.8, 21.2, 14.3.

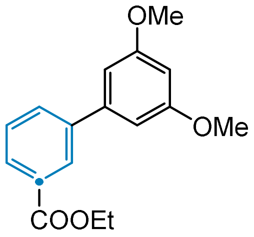

Ethyl 3',5'-dimethoxy-[1,1'-biphenyl]-3-carboxylate (4l)

Prepared according to general procedure using 3-(3,5-dimethoxyphenyl)pyridine (43.0 mg, 0.2 mmol), ethyl 2-(diethoxyphosphoryl)acetate (80 uL, 2.0 equiv.) were used. Purification by column chromatography using pre-basified silica with pentane/EtOAc (10:1 v/v, Rf = 0.4) as eluent afforded **4l** as colorless oil (17.3 mg, 30% yield).

^{1}H NMR (400 MHz, CDCl$_3$) δ 8.26 (s, 1H), 8.03 (dd, *J* = 7.7, 1.4 Hz, 1H), 7.80 – 7.71 (m, 1H), 7.50 (t, *J* = 7.7 Hz, 1H), 6.75 (d, *J* = 2.2 Hz, 2H), 6.50 (t, *J* = 2.2 Hz, 1H), 4.41 (q, *J* = 7.1 Hz, 2H), 3.86 (s, 6H), 1.42 (t, *J* = 7.1 Hz, 3H).

^{13}C NMR (101 MHz, CDCl$_3$) δ 166.5, 161.2, 142.4, 141.4, 131.5, 131.0, 128.7, 128.6, 128.2, 105.5, 99.6, 61.1, 55.5, 14.3.

HRMS (ESI): m/z calculated for [$C_{17}H_{18}O_4$] [M+Na$^+$]: 309.1097, found: 309.1096.

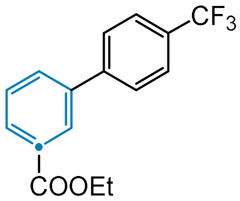

Ethyl 4'-(trifluoromethyl)-[1,1'-biphenyl]-3-carboxylate (4m)

Prepared according to general procedure using 3-(4-(trifluoromethyl)phenyl)pyridine (44.6 mg, 0.2 mmol), ethyl 2-(diethoxyphosphoryl)acetate (80 uL, 2.0 equiv.) were used. Purification by column chromatography using pre-basified silica with pentane/EtOAc (50:1 v/v, Rf = 0.4) as eluent afforded **4m** as colorless oil (29.3 mg, 50% yield).

^{1}H NMR (400 MHz, CDCl$_3$) δ 8.28 (t, *J* = 1.9 Hz, 1H), 8.09 (dt, *J* = 7.8, 1.4 Hz, 1H), 7.78 (ddd, *J* = 7.7, 2.0, 1.1 Hz, 1H), 7.72 (s, 4H), 7.55 (t, *J* = 7.8 Hz, 1H), 4.43 (q, *J* = 7.1 Hz, 2H), 1.43 (t, *J* = 7.1 Hz, 3H).

^{13}C NMR (101 MHz, CDCl$_3$) δ 166.3, 143.7, 140.0, 131.5, 131.3, 129.8 (q, *J* = 32.7 Hz), 129.2, 129.0, 128.3, 127.5, 125.8 (q, *J* = 3.7 Hz), 124.1 (q, *J* = 271.9 Hz), 61.2, 14.3.

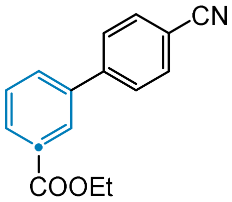

Ethyl 4'-cyano-[1,1'-biphenyl]-3-carboxylate (4n)

Prepared according to general procedure using 4-(pyridin-3-yl)benzonitrile (36.0 mg, 0.2 mmol), ethyl 2-(diethoxyphosphoryl)acetate (80 uL, 2.0 equiv.) were used. Purification by column chromatography using pre-basified silica with pentane/EtOAc (5:1 v/v, Rf = 0.3) as eluent afforded **4n** as colorless oil (25.2 mg, 50% yield).

^{1}H NMR (400 MHz, CDCl$_3$) δ 8.29 – 8.25 (m, 1H), 8.10 (dt, *J* = 7.8, 1.4 Hz, 1H), 7.80 – 7.69 (m, 5H), 7.56 (td, *J* = 7.8, 0.5 Hz, 1H), 4.42 (q, *J* = 7.1 Hz, 2H), 1.42 (t, *J* = 7.1 Hz, 3H).

^{13}C NMR (101 MHz, CDCl$_3$) δ 166.1, 144.6, 139.4, 132.7, 131.4, 131.4, 129.6, 129.2, 128.3, 127.8, 118.7, 111.4, 61.3, 14.3.

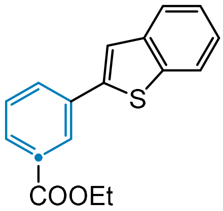

Ethyl 3-(benzo[b]thiophen-2-yl)benzoate (4o)

Prepared according to general procedure using 3-(benzo[b]thiophen-2-yl)pyridine (42.2 mg, 0.2 mmol), ethyl 2-(diethoxyphosphoryl)acetate (80 uL, 2.0 equiv.) were used. Purification by column chromatography using pre-basified silica with pentane/EtOAc (20:1 v/v, Rf = 0.3) as eluent afforded **4o** as white solid (25.2 mg, 45% yield).

^{1}H NMR (400 MHz, CDCl$_3$) δ 8.40 (s, 1H), 8.02 (dd, *J* = 7.9, 1.3 Hz, 1H), 7.91 – 7.83 (m, 2H), 7.82 – 7.77 (m, 1H), 7.63 (s, 1H), 7.50 (t, *J* = 7.8 Hz, 1H), 7.40 – 7.31 (m, 2H), 4.44 (q, *J* = 7.1 Hz, 2H), 1.44 (t, *J* = 7.1 Hz, 3H).

^{13}C NMR (101 MHz, CDCl$_3$) δ 166.2, 143.0, 140.5, 139.6, 134.6, 131.3, 130.6, 129.1, 129.0, 127.4, 124.6, 124.6, 123.7, 122.3, 120.2, 61.2, 14.3.

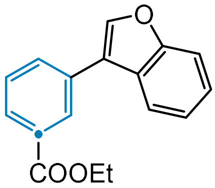

Ethyl 3-(benzofuran-3-yl)benzoate (4p)

Prepared according to general procedure using 3-(benzofuran-3-yl)pyridine (39.0 mg, 0.2 mmol), ethyl 2-(diethoxyphosphoryl)acetate (80 uL, 2.0 equiv.) were used. Purification by column chromatography using pre-basified silica with pentane/EtOAc (20:1 v/v, Rf = 0.4) as eluent afforded **4p** as colorless oil (23.7 mg, 45% yield).

^{1}H NMR (400 MHz, CDCl$_3$) δ 8.34 (td, *J* = 1.8, 0.5 Hz, 1H), 8.05 (dt, *J* = 7.9, 1.4 Hz, 1H), 7.88 – 7.82 (m, 3H), 7.60 – 7.52 (m, 2H), 7.42 – 7.32 (m, 2H), 4.43 (q, *J* = 7.1 Hz, 2H), 1.43 (t, *J* = 7.1 Hz, 3H).

^{13}C NMR (101 MHz, CDCl$_3$) δ 166.4, 155.8, 141.7, 132.4, 131.6, 131.3, 129.0, 128.5, 126.2, 124.7, 123.2, 121.5, 120.2, 111.8, 61.1, 14.3.

HRMS (ESI): m/z calculated for [$C_{17}H_{14}O_3$] [M+Na$^+$]: 289.0835, found: 289.0833.

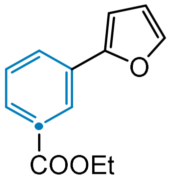

Ethyl 3-(furan-2-yl)benzoate (4q)

Prepared according to general procedure using 3-(furan-2-yl)pyridine (29.0 mg, 0.2 mmol), ethyl 2-(diethoxyphosphoryl)acetate (80 uL, 2.0 equiv.) were used. Purification by column chromatography using pre-basified silica with pentane/EtOAc (20:1 v/v, Rf = 0.4) as eluent afforded **4q** as colorless oil (17.1 mg, 40% yield).

^{1}H NMR (400 MHz, CDCl$_3$) δ 8.33 (t, *J* = 1.8 Hz, 1H), 7.93 (dt, *J* = 7.8, 1.5 Hz, 1H), 7.85 (dt, *J* = 7.8, 1.5 Hz, 1H), 7.50 (dd, *J* = 1.8, 0.8 Hz, 1H), 7.46 (t, *J* = 7.8 Hz, 1H), 6.74 (dd, *J* = 3.4, 0.8 Hz, 1H), 6.50 (dd, *J* = 3.4, 1.8 Hz, 1H), 4.41 (q, *J* = 7.1 Hz, 2H), 1.42 (t, *J* = 7.1 Hz, 3H).

^{13}C NMR (101 MHz, CDCl$_3$) δ 166.4, 153.0, 142.5, 132.9, 131.1, 128.7, 128.2, 127.8, 124.9, 111.8, 105.8, 61.1, 14.3.

HRMS (ESI): m/z calculated for [$C_{13}H_{12}O_3$] [M+Na$^+$]: 239.0679, found: 239.0677.

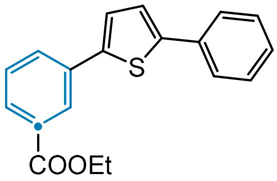

Ethyl 3-(5-phenylthiophen-2-yl)benzoate (4r)

Prepared according to general procedure using 3-(5-phenylthiophen-2-yl)pyridine (47.4 mg, 0.2 mmol), ethyl 2-(diethoxyphosphoryl)acetate (80 uL, 2.0 equiv.) were used. Purification by column chromatography using pre-basified silica with pentane/EtOAc (20:1 v/v, Rf = 0.4) as eluent afforded **4r** as colorless oil (26.3 mg, 43% yield).

^{1}H NMR (400 MHz, CDCl$_3$) δ 8.34 – 8.28 (m, 1H), 7.96 (dt, *J* = 7.9, 1.4 Hz, 1H), 7.80 (ddd, *J* = 7.8, 2.0, 1.1 Hz, 1H), 7.67 – 7.62 (m, 2H), 7.46 (td, *J* = 7.8, 0.5 Hz, 1H), 7.43 – 7.35 (m, 3H), 7.33 – 7.28 (m, 2H), 4.43 (q, *J* = 7.1 Hz, 2H), 1.43 (t, *J* = 7.1 Hz, 3H).

^{13}C NMR (101 MHz, CDCl$_3$) δ 166.3, 144.3, 142.3, 134.6, 134.1, 131.2, 129.7, 128.9, 128.3, 127.7, 126.5, 125.7, 124.7, 124.1, 61.2, 14.3.

HRMS (ESI): m/z calculated for [$C_{19}H_{16}O_2S$] [M+Na$^+$]: 331.0763, found: 331.0762.

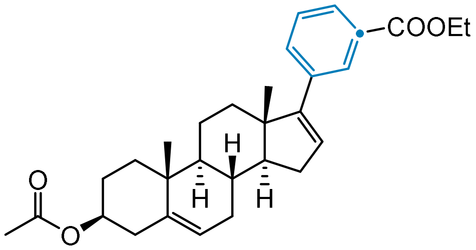

Ethyl 3-((3S,8R,9S,10R,13S,14S)-3-acetoxy-10,13-dimethyl-2,3,4,7,8,9,10,11,12,13,14,15-dodecahydro-1*H*-cyclopenta[a]phenanthren-17-yl)benzoate (4s)

Prepared according to general procedure using abiraterone acetate (78.2 mg, 0.2 mmol), dimethyl (2-oxopropyl)phosphonate (55 uL, 2.0 equiv.) were used. Purification by column chromatography using pre-basified silica with pentane/EtOAc (10:1 v/v, Rf = 0.3) as eluent afforded **4s** as white solid (51.0 mg, 55% yield).

^{1}H NMR (400 MHz, CDCl$_3$) δ 8.16 (d, *J* = 15.8 Hz, 1H), 7.33 (d, *J* = 7.8 Hz, 1H), 7.23 (d, *J* = 7.2 Hz, 1H), 7.12 (t, *J* = 7.6 Hz, 1H), 6.29 (d, *J* = 15.8 Hz, 1H), 5.42 (d, *J* = 4.9 Hz, 1H), 4.62 (ddd, *J* = 11.5, 8.5, 3.5 Hz, 1H), 4.26 (q, *J* = 7.1 Hz, 2H), 2.74 (dd, *J* = 14.7, 6.4 Hz, 1H), 2.56 – 2.47 (m, 2H), 2.40 – 2.32 (m, 2H), 2.13 – 2.07 (m, 1H), 2.04 (s, 3H), 1.92 – 1.86 (m, 2H), 1.81 – 1.62 (m, 7H), 1.34 (t, *J* = 7.1 Hz, 3H), 1.18 – 1.11 (m, 2H), 1.10 (s, 3H), 1.06 (s, 3H).

^{13}C NMR (101 MHz, CDCl$_3$) δ 170.5, 167.1, 152.7, 143.8, 142.6, 140.0, 130.5, 126.6, 126.4, 124.4, 122.1, 119.0, 73.8, 60.4, 57.0, 50.0, 47.0, 38.1, 37.1, 36.8, 36.7, 32.3, 31.6, 30.7, 27.7, 21.4, 21.0, 19.3, 17.7, 14.3.

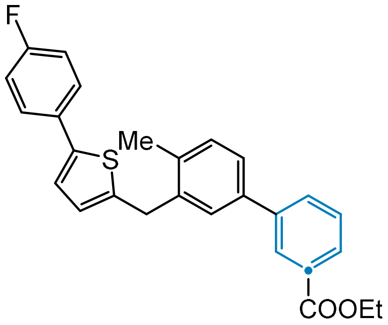

Ethyl 3'-((5-(4-fluorophenyl)thiophen-2-yl)methyl)-4'-methyl-[1,1'-biphenyl]-3-carboxylate (4t)

Prepared according to general procedure using 3-(3-((5-(4-fluorophenyl)thiophen-2-yl)methyl)-4-methylphenyl)pyridine (71.8 mg, 0.2 mmol), dimethyl (2-oxopropyl)phosphonate (55 uL, 2.0 equiv.)

were used. Purification by column chromatography using pre-basified silica with pentane/EtOAc (20:1 v/v, Rf = 0.3) as eluent afforded **4t** as colorless oil (30.1 mg, 42% yield).

^{1}H NMR (400 MHz, CDCl$_3$) δ 8.27 (t, *J* = 1.8 Hz, 1H), 8.00 (dt, *J* = 7.7, 1.4 Hz, 1H), 7.77 (ddd, *J* = 7.7, 1.9, 1.2 Hz, 1H), 7.52 – 7.43 (m, 5H), 7.28 (d, *J* = 7.9 Hz, 1H), 7.06 – 6.97 (m, 3H), 6.73 – 6.68 (m, 1H), 4.40 (q, *J* = 7.1 Hz, 2H), 4.21 (s, 2H), 2.38 (s, 3H), 1.40 (t, *J* = 7.1 Hz, 3H).

^{13}C NMR (101 MHz, CDCl$_3$) δ 166.6, 162.1 (d, *J* = 246.7 Hz), 143.2, 141.6, 141.2, 138.7, 138.1, 136.1, 131.3, 131.1, 131.0, 130.8 (d, *J* = 3.4 Hz), 128.8, 128.3, 128.1, 128.0, 127.1 (d, *J* = 8.0 Hz), 126.0, 125.7, 122.7, 115.7 (d, *J* = 21.8 Hz), 61.1, 34.3, 19.2, 14.3.

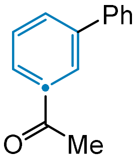

1-([1,1'-Biphenyl]-3-yl)ethan-1-one (5a)

Prepared according to general procedure using 3-phenylpyridine (29 uL, 0.2 mmol), dimethyl (2-oxopropyl)phosphonate (55 uL, 2.0 equiv.) were used. Purification by column chromatography using pre-basified silica with pentane/EtOAc (50:1 v/v, Rf = 0.4) as eluent afforded **5a** as yellow oil (17.4 mg, 44% yield).

^{1}H NMR (400 MHz, CDCl$_3$) δ 8.19 (td, *J* = 1.9, 0.5 Hz, 1H), 7.94 (ddd, *J* = 7.8, 1.8, 1.1 Hz, 1H), 7.80 (ddd, *J* = 7.7, 1.9, 1.1 Hz, 1H), 7.65 – 7.59 (m, 2H), 7.54 (td, *J* = 7.7, 0.6 Hz, 1H), 7.51 – 7.43 (m, 2H), 7.42 – 7.36 (m, 1H), 2.66 (s, 3H).

^{13}C NMR (101 MHz, CDCl$_3$) δ 198.1, 141.7, 140.2, 137.6, 131.7, 129.0, 128.9, 127.8, 127.2, 127.2, 127.0, 26.7.

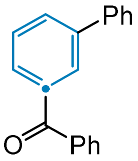

[1,1'-Biphenyl]-3-yl(phenyl)methanone (5b)

Prepared according to general procedure using 3-phenylpyridine (29 uL, 0.2 mmol), diethyl (2-oxo-2-phenylethyl)phosphonate (87 uL, 2.0 equiv.) were used. Purification by column chromatography using pre-basified silica with pentane/EtOAc (50:1 v/v, Rf = 0.4) as eluent afforded **5b** as colorless oil (20.8 mg, 40% yield).

^{1}H NMR (400 MHz, CDCl$_3$) δ 8.06 – 8.01 (m, 1H), 7.90 – 7.84 (m, 2H), 7.82 (ddd, *J* = 7.7, 1.9, 1.2 Hz, 1H), 7.77 (dt, *J* = 7.8, 1.4 Hz, 1H), 7.61 (ddt, *J* = 8.2, 6.7, 1.4 Hz, 3H), 7.56 (t, *J* = 7.7 Hz, 1H), 7.53 – 7.44 (m, 4H), 7.41 – 7.36 (m, 1H).

^{13}C NMR (101 MHz, CDCl$_3$) δ 196.7, 141.4, 140.2, 138.2, 137.6, 132.5, 131.0, 130.1, 128.9, 128.7, 128.6, 128.3, 127.8, 127.2.

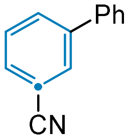

[1,1'-Biphenyl]-3-carbonitrile (5c)

Prepared according to general procedure using 3-phenylpyridine (29 uL, 0.2 mmol), diethyl (cyanomethyl)phosphonate (65 uL, 2.0 equiv.) were used. Purification by column chromatography using pre-basified silica with pentane/EtOAc (50:1 v/v, Rf = 0.4) as eluent afforded **5c** as yellow oil (22.1 mg, 62% yield).

^{1}H NMR (400 MHz, CDCl$_3$) δ 7.89 – 7.84 (m, 1H), 7.82 (ddd, *J* = 7.9, 1.9, 1.3 Hz, 1H), 7.63 (dt, *J* = 7.7, 1.4 Hz, 1H), 7.59 – 7.52 (m, 3H), 7.48 (ddd, *J* = 8.2, 7.0, 1.0 Hz, 2H), 7.45 – 7.39 (m, 1H).

^{13}C NMR (101 MHz, CDCl$_3$) δ 142.4, 138.9, 131.5, 130.7, 130.7, 129.6, 129.1, 128.4, 127.1, 118.8, 112.9.

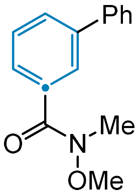

***N*-Methoxy-*N*-methyl-[1,1'-biphenyl]-3-carboxamide (5d)**

Prepared according to general procedure using 3-phenylpyridine (29 uL, 0.2 mmol), diethyl (2-(methoxy(methyl)amino)-2-oxoethyl)phosphonate (82 uL, 2.0 equiv.) were used. Purification by column chromatography using pre-basified silica with pentane/EtOAc (2:1 v/v, Rf = 0.4) as eluent afforded **5d** as yellow oil (26.8 mg, 56% yield).

^{1}H NMR (400 MHz, CDCl$_3$) δ 7.90 (t, *J* = 1.8 Hz, 1H), 7.69 (ddd, *J* = 7.7, 1.9, 1.2 Hz, 1H), 7.66 – 7.58 (m, 3H), 7.51 – 7.43 (m, 3H), 7.39 – 7.33 (m, 1H), 3.59 (s, 3H), 3.39 (s, 3H).

^{13}C NMR (101 MHz, CDCl$_3$) δ 169.9, 141.0, 140.4, 134.7, 129.2, 128.8, 128.5, 127.6, 127.1, 126.9, 126.9, 61.1, 33.8.

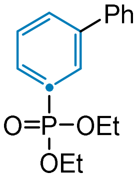

Diethyl [1,1'-biphenyl]-3-ylphosphonate (5e)

Prepared according to general procedure using 3-phenylpyridine (29 uL, 0.2 mmol), tetraethyl methylenebis(phosphonate) (99 uL, 2.0 equiv.) were used. Purification by column chromatography

using pre-basified silica with EtOAc (Rf = 0.4) as eluent afforded **5e** as yellow oil (22.0 mg, 38% yield).

^{1}H NMR (400 MHz, CDCl$_3$) δ 8.04 (dt, *J* = 14.2, 1.8 Hz, 1H), 7.83 – 7.75 (m, 2H), 7.63 – 7.58 (m, 2H), 7.54 (td, *J* = 7.7, 4.4 Hz, 1H), 7.49 – 7.42 (m, 2H), 7.40 – 7.36 (m, 1H), 4.25 – 4.06 (m, 4H), 1.34 (t, *J* = 7.1 Hz, 6H).

^{13}C NMR (101 MHz, CDCl$_3$) δ 141.5 (d, *J* = 15.0 Hz), 140.0, 140.0, 131.0 (d, *J* = 3.2 Hz), 130.5 (d, *J* = 5.9 Hz), 130.4 (d, *J* = 6.8 Hz), 128.9 (d, *J* = 15.7 Hz), 128.9, 127.8, 127.2, 62.2, 62.1, 16.4, 16.3.

^{31}P NMR (162 MHz, CDCl$_3$) δ 18.71.

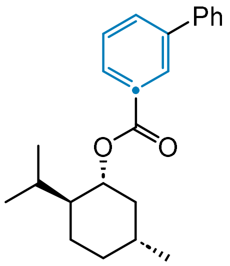

(1R,2S,5R)-2-Isopropyl-5-methylcyclohexyl [1,1'-biphenyl]-3-carboxylate (5f)

Prepared according to general procedure using 3-phenylpyridine (29 uL, 0.2 mmol), (1R,2S,5R)-2-isopropyl-5-methylcyclohexyl 2-(diethoxyphosphoryl)acetate (133.6 mg, 2.0 equiv.) were used. Purification by column chromatography using pre-basified silica with pentane/EtOAc (50:1 v/v, Rf = 0.4) as eluent afforded **5f** as colorless oil (31.2 mg, 47% yield).

^{1}H NMR (400 MHz, CDCl$_3$) δ 8.28 (t, *J* = 1.8 Hz, 1H), 8.04 (dt, *J* = 7.8, 1.4 Hz, 1H), 7.78 (ddd, *J* = 7.7, 2.0, 1.2 Hz, 1H), 7.66 – 7.61 (m, 2H), 7.54 – 7.45 (m, 3H), 7.42 – 7.37 (m, 1H), 4.99 (td, *J* = 10.9, 4.4 Hz, 1H), 2.15 (dtd, *J* = 12.0, 4.0, 1.7 Hz, 1H), 1.98 (ddt, *J* = 14.0, 7.0, 3.5 Hz, 1H), 1.78 – 1.71 (m, 2H), 1.62 – 1.55 (m, 2H), 1.19 – 1.08 (m, 2H), 0.94 (dd, *J* = 6.8, 5.5 Hz, 7H), 0.82 (d, *J* = 6.9 Hz, 3H).

^{13}C NMR (101 MHz, CDCl$_3$) δ 166.0, 141.5, 140.3, 131.4, 131.4, 128.9, 128.8, 128.3, 128.2, 127.7, 127.2, 75.0, 47.3, 41.0, 34.3, 31.5, 26.5, 23.7, 22.0, 20.8, 16.6.

4. Limitations

4.1 limitation of pyridines

i) No imine or low-yielding imine formation

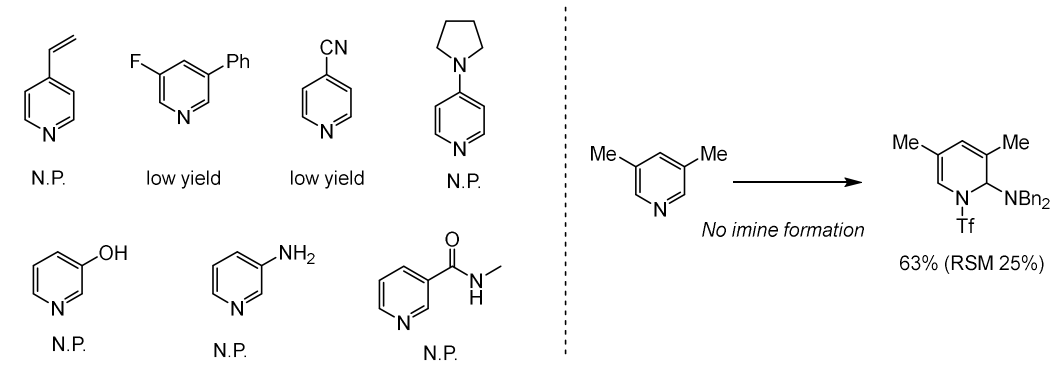

ii) Problematic hydrolysis step

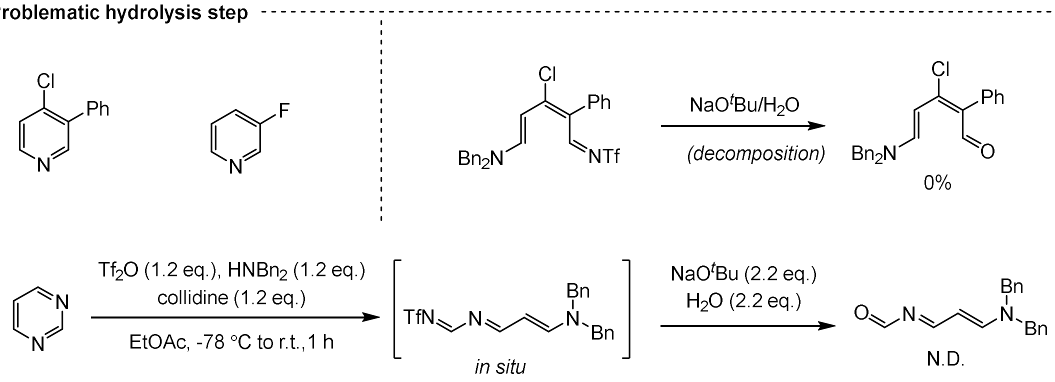

iii) Low yielding alkene foramtion during olefination step

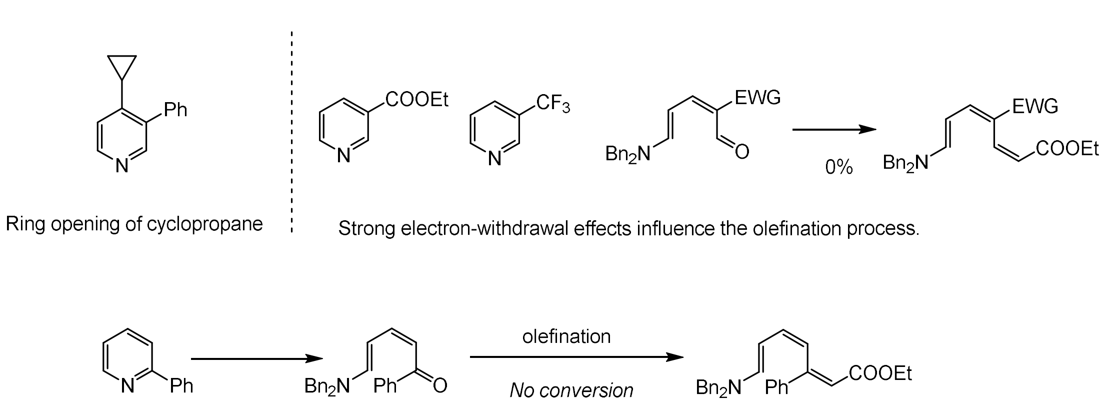

iv) Problematic ring closure step

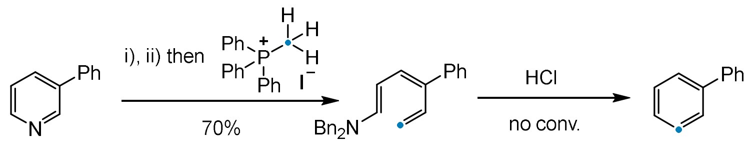

4.2 limitation of phosphine reagents

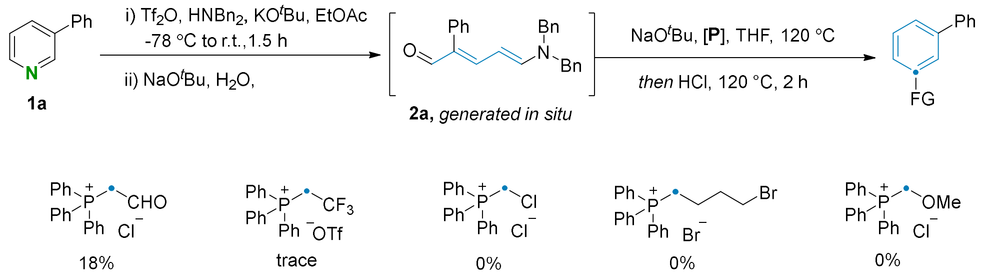

5. X-Ray analysis

X-Ray diffraction: Data sets for compound **2a** were collected with a Bruker D8 Venture Photon III Diffractometer. Programs used: data collection: *APEX4* Version 2021.4-0 [1] (Bruker AXS Inc., **2021**); cell refinement: *SAINT* Version 8.40B (Bruker AXS Inc., **2021**); data reduction: *SAINT* Version 8.40B (Bruker AXS Inc., **2021**); absorption correction, *SADABS* Version 2016/2 (Bruker AXS Inc., **2021**); structure solution *SHELXT*-Version 2018-3 [2] (Sheldrick, G. M. *Acta Cryst.,* **2015**, *A71*, 3-8); structure refinement *SHELXL*-Version 2018-3 [3] (Sheldrick, G. M. *Acta Cryst.,* **2015**, *C71* (1), 3-8) and graphics, *XP* [4] (Version 5.1, Bruker AXS Inc., Madison, Wisconsin, USA, **1998**). *R*-values are given for observed reflections, and wR^2 values are given for all reflections.

X-ray crystal structure analysis of 2a: A orange-yellow, block-like specimen of $C_{25}H_{23}NO$, approximate dimensions 0.074 mm x 0.090 mm x 0.110 mm, was used for the X-ray crystallographic analysis. The X-ray intensity data were measured on a single crystal diffractometer Bruker D8 Venture Photon III system equipped with a micro focus tube Cu ImS (CuKα, λ = 1.54178 Å) and a MX mirror monochromator. A total of 1432 frames were collected. The total exposure time was 22.40 hours. The frames were integrated with the Bruker SAINT software package using a wide-frame algorithm. The integration of the data using a monoclinic unit cell yielded a total of 29807 reflections to a maximum θ angle of 66.68° (0.84 Å resolution), of which 3305 were independent (average redundancy 9.019, completeness = 99.4%, R_{int} = 9.09%, R_{sig} = 3.84%) and 2617 (79.18%) were greater than $2\sigma(F^2)$. The final cell constants of a = 16.5300(3) Å, b = 5.94310(10) Å, c = 20.4998(4) Å, β = 110.4490(10)°, volume = 1886.98(6) Å^3, are based upon the refinement of the XYZ-centroids of 8534 reflections above 20 σ(I) with 5.948° < 2θ < 132.7°. Data were corrected for absorption effects using the multi-scan method (SADABS). The ratio of minimum to maximum apparent transmission was 0.918. The calculated minimum and maximum transmission coefficients (based on crystal size) are 0.9390 and 0.9580. The structure was solved and refined using the Bruker SHELXTL Software Package, using the space group $P2_1/n$, with Z = 4 for the formula unit, $C_{25}H_{23}NO$. The final anisotropic full-matrix least-squares refinement on F^2 with 244 variables converged at R1 = 4.18%, for the observed data and wR2 = 10.30% for all data. The goodness-of-fit was 1.049. The largest peak in the

final difference electron density synthesis was 0.247 $e^-/Å^3$ and the largest hole was -0.181 $e^-/Å^3$ with an RMS deviation of 0.039 $e^-/Å^3$. On the basis of the final model, the calculated density was 1.244 g/cm^3 and F(000), 752 e^-. CCDC Nr.: 2334460.

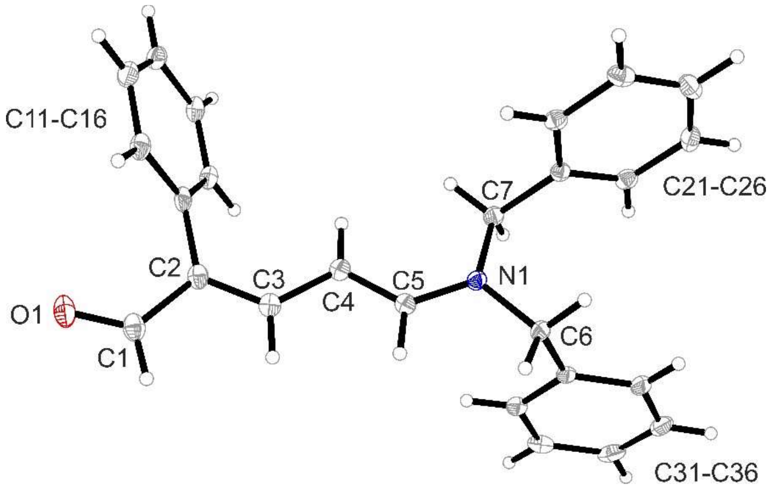

Figure S1. Crystal structure of compound **2a**.
Thermal ellipsoids are shown at 30% probability.

6. DFT calculation

Computational Details

All geometry optimizations of intermediates and transition states were achieved using spin-unrestricted UB3LYP[5]-D3[6]/def2-SVP[7] method, in tetrahydrofuran solvent using the CPCM solvent model[8] with “opt=noeigen” and “guess=mix” keywords as implemented in Gaussian16[9]. Frequency calculations were also conducted at the same level of theory to obtain vibrational frequencies to determine the identity of stationary points as intermediates (no imaginary frequencies) or transition states (only one imaginary frequency), as well as obtaining the thermochemistry: enthalpy (ΔH) and free energy (ΔG) at the temperature of 298 K. Single point energy calculations were done using the following methods:

UB3LYP-D3/def2TZVPP-CPCM(THF)//UB3LYP-D3/def2svp-CPCM(THF)
UB3LYP-D3/aug-CC-PVTZ-CPCM(THF)//UB3LYP-D3/def2svp-CPCM(THF)
UPBEPBE-D3/aug-CC-PVTZ-CPCM(THF)//UB3LYP-D3/def2svp-CPCM(THF)
UM06/aug-CC-PVTZ-CPCM(THF)//UB3LYP-D3/def2svp-CPCM(THF).

Extensive conformational analysis was done for all key transition states and stationary points and the minimum energy structures were chosen for the minimum energy profiles. All structural figures were generated with CYLview.[10] Distances in structural figures are shown in Å and energies are in kcal/mol.

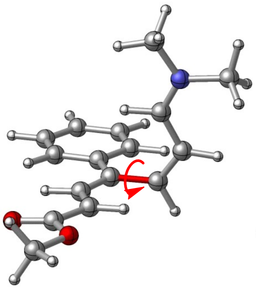

TS-3a-r
0.0 (0.0) [0.0]

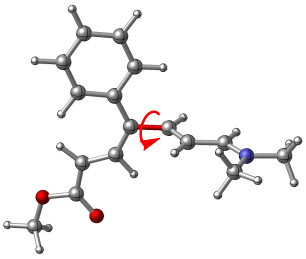

TS-3a-r-conf1
5.6 (5.9) [6.4]

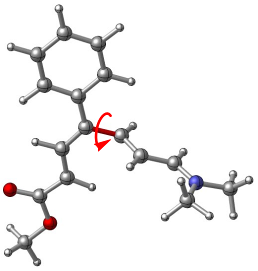

TS-3a-r-conf2
1.9 (2.1) [2.3]

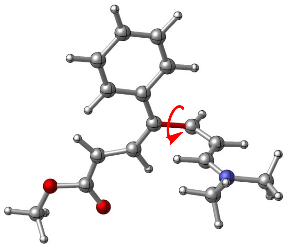

TS-3a-r-conf3
4.2 (4.5) [5.0]

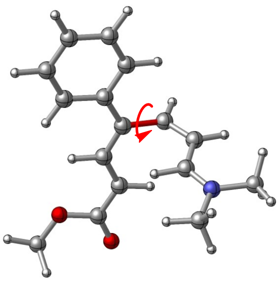

TS-3a-r-conf4
0.8 (0.9) [0.6]

Figure S2. Different conformations for the C_γ-C_δ rotation transition state for substrate **3a** calculated at the theoretical level: UB3LYP-d3/def2-svp-CPCM(THF). Calculated energies, enthalpy (parentheses) and free Gibbs energies [brackets] are given in kcal mol^{-1}.

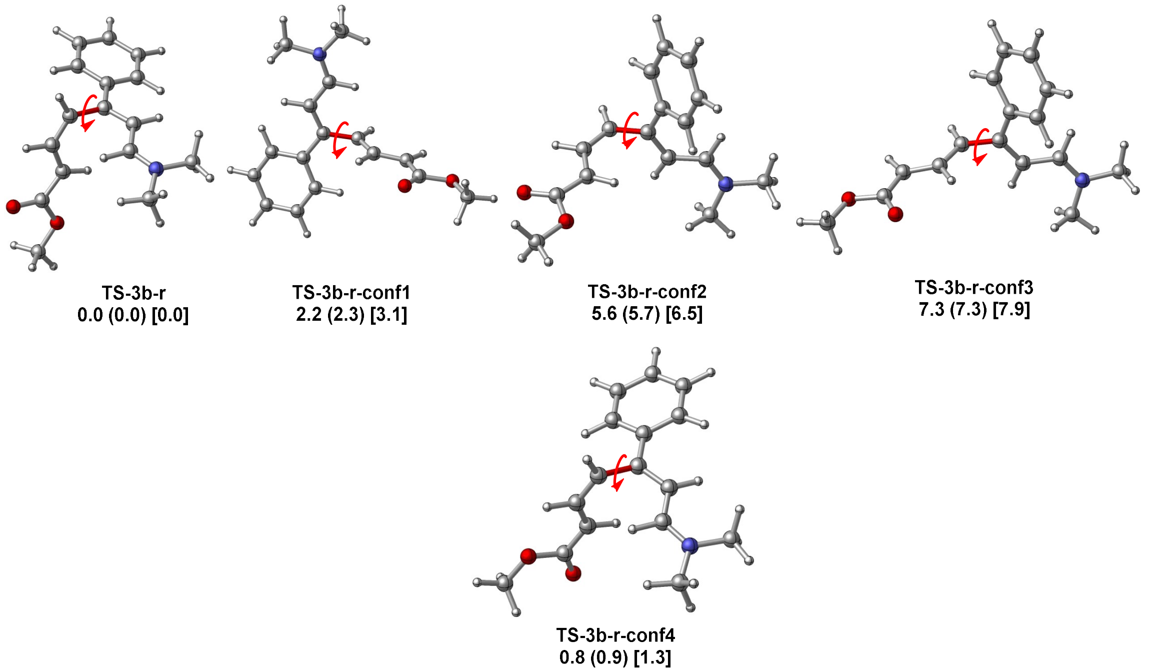

Figure S3. Different conformations for the C_{γ}-C_{δ} rotation transition state for substrate **3b** calculated at the theoretical level: UB3LYP-d3/def2-svp-CPCM(THF). Calculated energies, enthalpy (parentheses) and free Gibbs energies [brackets] are given in kcal mol^{-1}.

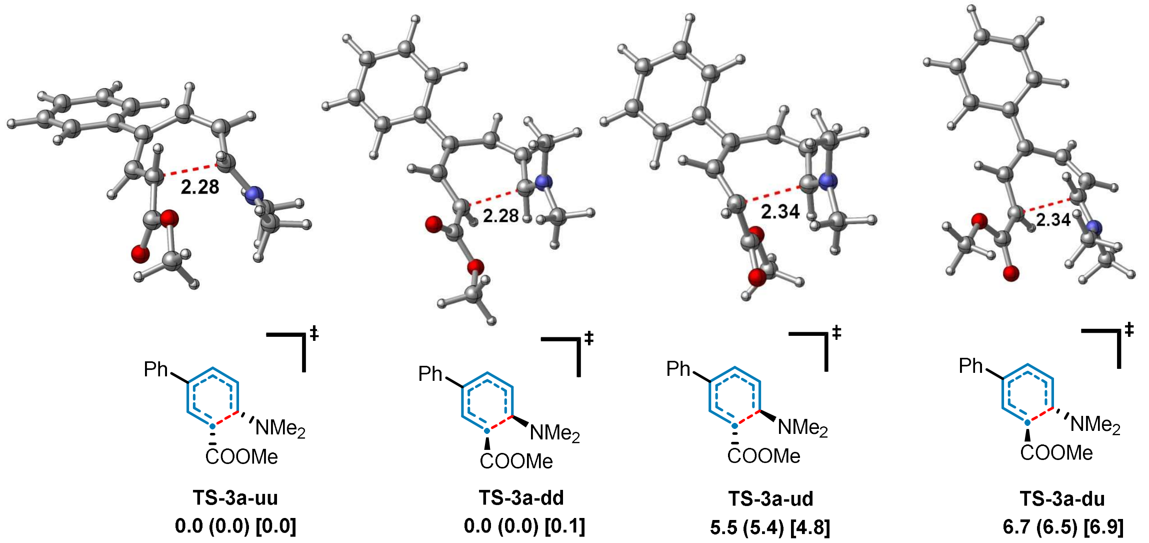

Figure S4. Different conformations for the electrocyclization transition state for substrate **3a** calculated at the theoretical level: UB3LYP-d3/def2-svp-CPCM(THF). Calculated energies, enthalpy (parentheses) and free Gibbs energies [brackets] are given in kcal mol^{-1}.

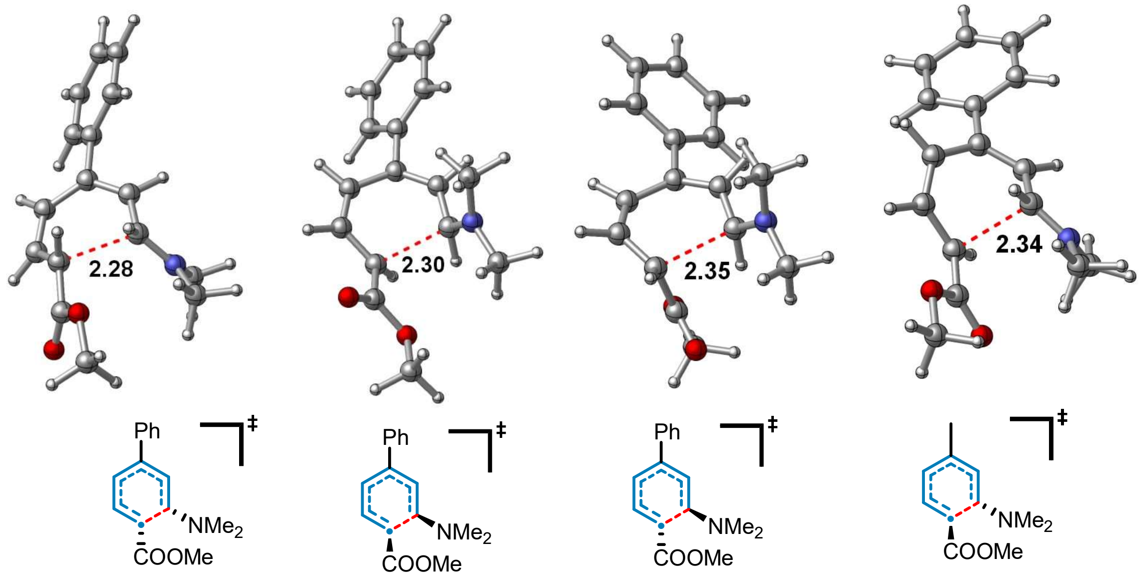

TS-3b-uu 0.0 (0.0) [0.0] | TS-3b-dd 0.3 (0.3) [0.3] | TS-3b-ud 4.8 (4.7) [4.6] | TS-3b-du 5.2 (5.1) [5.4]

Figure S5. Different conformations for the electrocyclization transition state for substrate **3b** calculated at the theoretical level: UB3LYP-d3/def2-svp-CPCM(THF). Calculated energies, enthalpy (parentheses) and free Gibbs energies [brackets] are given in kcal mol^{-1}.

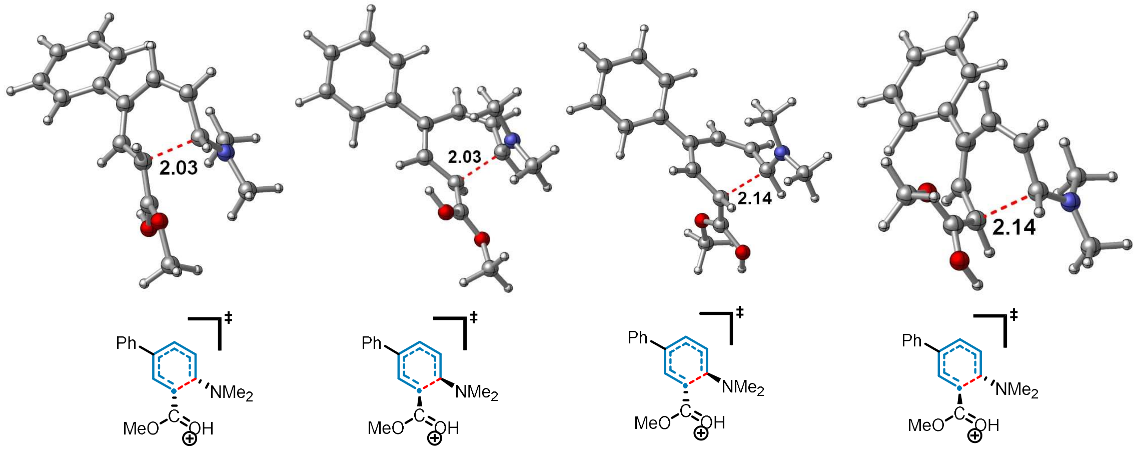

TS-3a-OH-uu' 0.0 (0.0) [0.0] | TS-3a-OH-dd' 0.1 (0.1) [0.1] | TS-3a-OH-ud' 4.5 (4.0) [3.0] | TS-3a-OH-du' 1.7 (1.5) [0.9]

Figure S6. Different conformations for the electrocyclization transition state for the protonated substrate **3a-OH** (without chloride as counter-ion) calculated at the theoretical level: UB3LYP-d3/def2-svp-CPCM(THF). Calculated energies, enthalpy (parentheses) and free Gibbs energies [brackets] are given in kcal mol^{-1}.

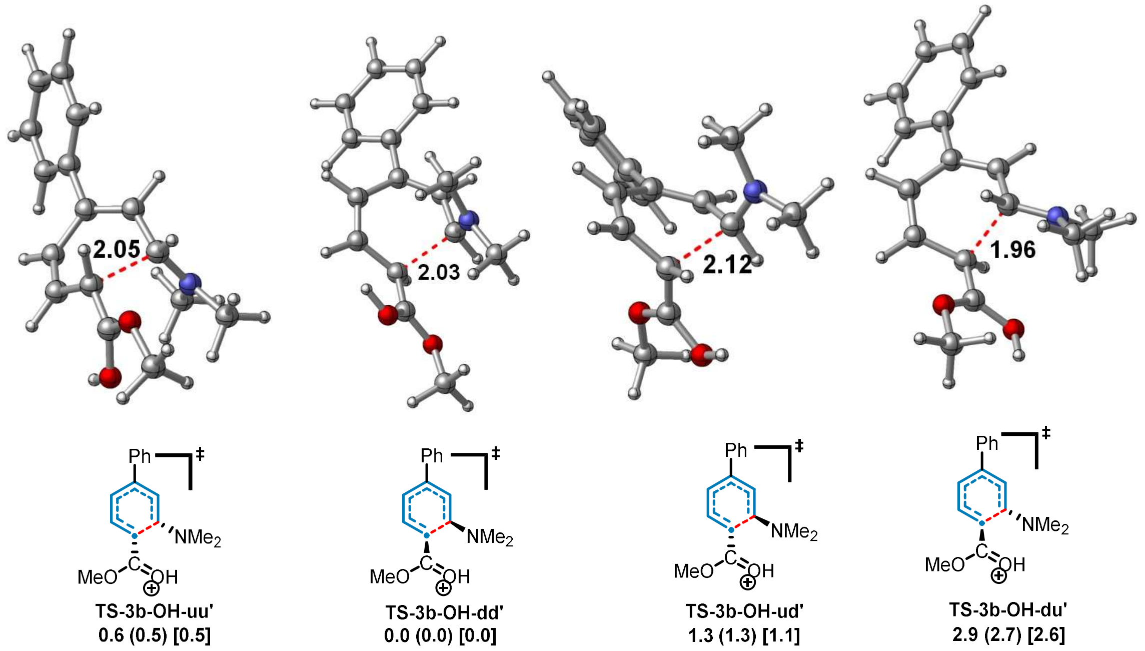

Figure S7. Different conformations for the electrocyclization transition state for the protonated substrate **3b-OH** (without chloride as counter-ion) calculated at the theoretical level: UB3LYP-d3/def2-svp-CPCM(THF). Calculated energies, enthalpy (parentheses) and free Gibbs energies [brackets] are given in kcal mol^{-1}.

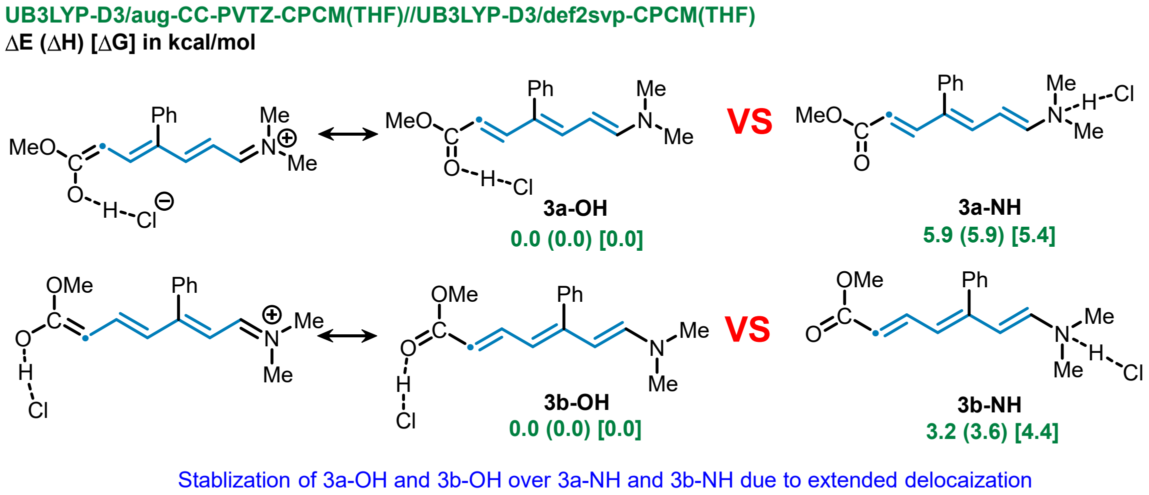

Figure S8. Energy preferences for protonation at the carbonyl oxygen over the nitrogen due to extended delocalization calculated at the theoretical level: UB3LYP-D3/aug-CC-PVTZ-CPCM(THF)//UB3LYP-d3/def2-svp-CPCM(THF). Calculated energies, enthalpy (parentheses) and free Gibbs energies [brackets] are given in kcal mol^{-1}.

- Under acidic conditions, Carbonyl oxygen gets protonated rather than N.

- Thermodynamically, protonation of carbonyl O is favored over N.
- If carbonyl oxygen gets protonated, the positive charge can delocalize, and this also helps in lowering the barrier of rotation whereas that is not possible if N gets protonated.

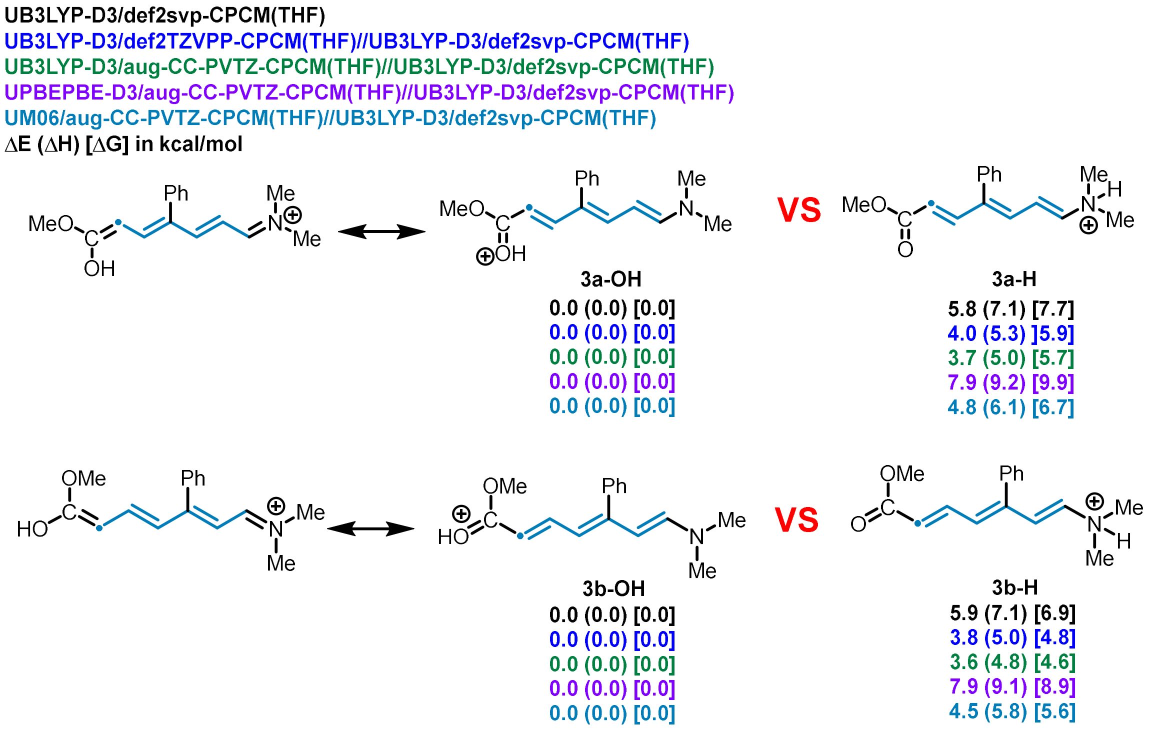

Figure S9. Energy preferences for protonation at the carbonyl oxygen over the nitrogen due to extended delocalization calculated at different methods without the chloride as counter-ion. Calculated energies, enthalpy (parentheses) and free Gibbs energies [brackets] are given in kcal mol^{-1}.

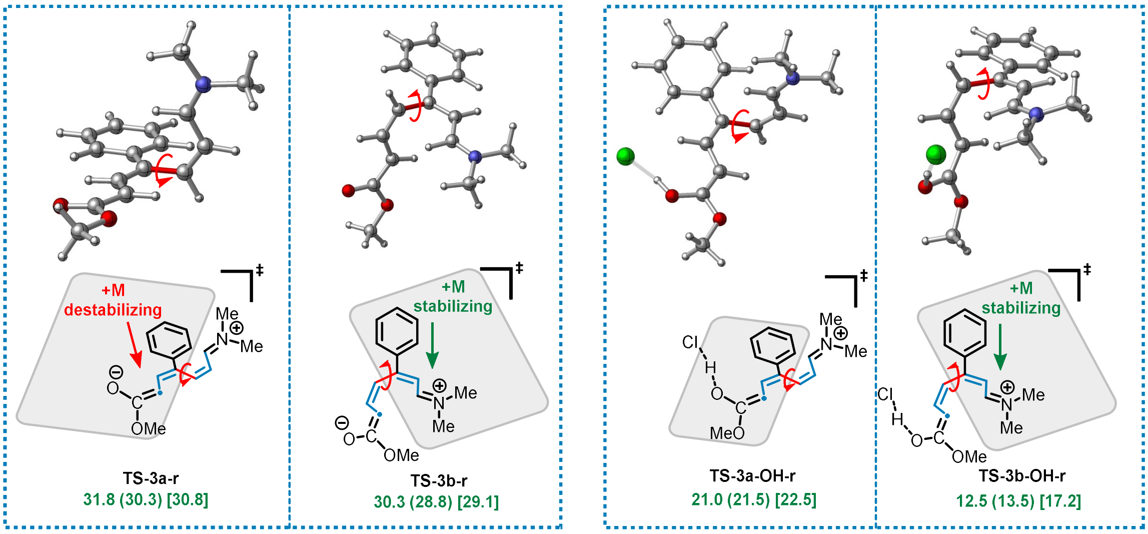

Figure S10. Key C_γ-C_δ rotation transition states for substrates **3a** and **3b** under basic (left) and acidic (right) conditions, highlighting the effect of protonation in lowering the rotation barrier calculated at the theoretical level: UB3LYP-D3/aug-CC-PVTZ-CPCM(THF)//UB3LYP-d3/def2-svp-CPCM(THF). Calculated energies, enthalpy (parentheses) and free Gibbs energies [brackets] are given in kcal mol^{-1}.

Under basic conditions, the barrier for double bond rotation for the 3-substituted substrate (**3a**) is around 1.7 kcal higher as compared to the 4-substituted substrate (**3b**) consistent with experiments. Under acidic conditions that barrier becomes much lower (below 20 kcal) presumably because of the resonance structure shown above in which the double bond in question has reduced π-bond character.

TS-3b-r has lower barrier because in that TS, the phenyl ring is in the same plane as the NMe2 group thus stabilizing the positive charge on N via +M effect. The Opposite is true for **TS-3a-r** where phenyl ring destabilizes the negative charge on O.

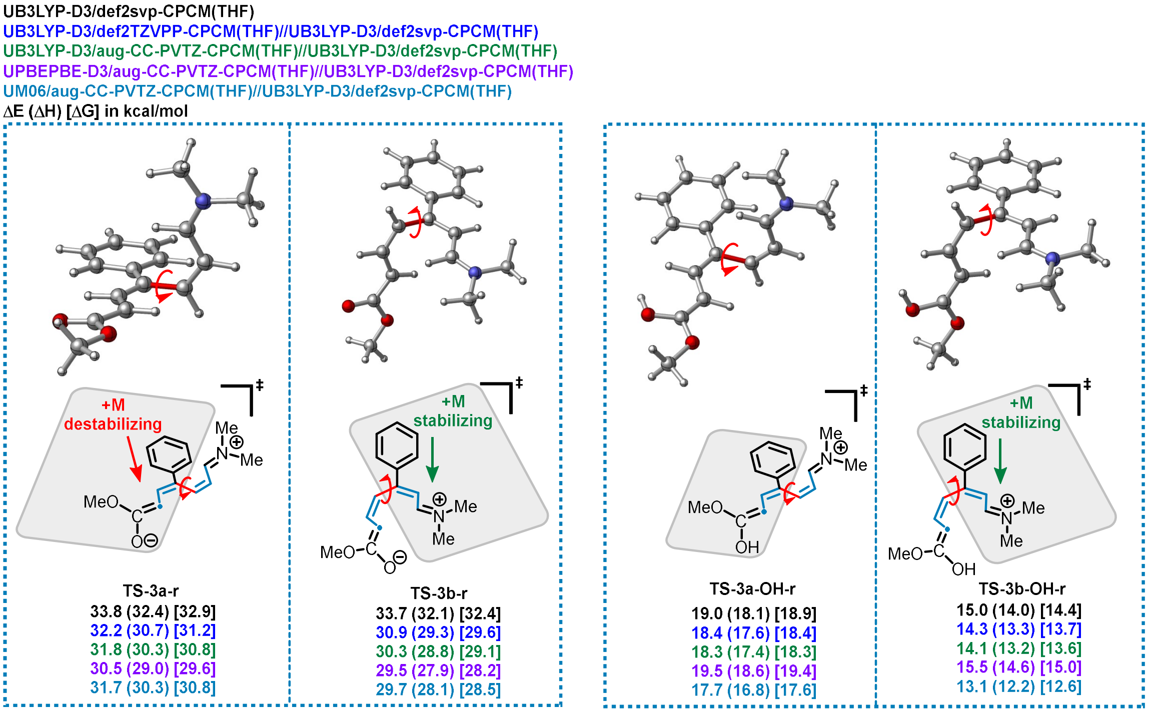

Figure S11. Key C_γ-C_δ rotation transition states for substrates **3a** and **3b** under basic (left) and acidic (right) conditions without the chloride as counter-ion, highlighting the effect of protonation in lowering the rotation barrier calculated at different methods. Calculated energies, enthalpy (parentheses) and free Gibbs energies [brackets] are given in kcal mol^{-1}.

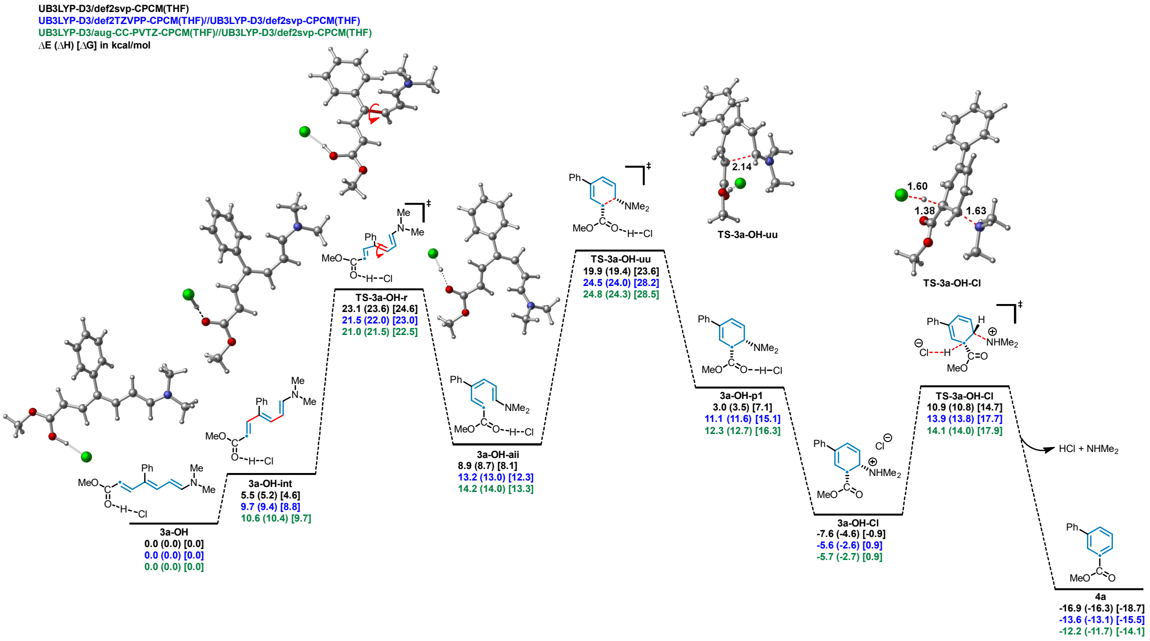

Figure S12. Full minimum energy pathway for substrate **3a** under acidic conditions calculated at different methods.

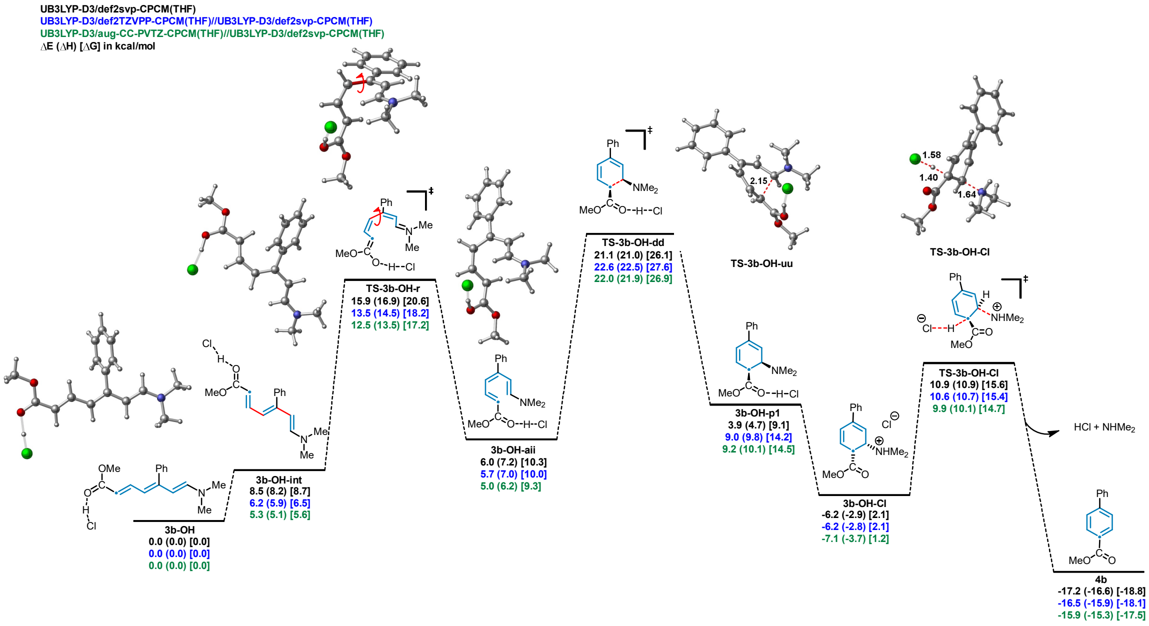

Figure S13. Full minimum energy pathway for substrate **3b** under acidic conditions calculated at different methods.

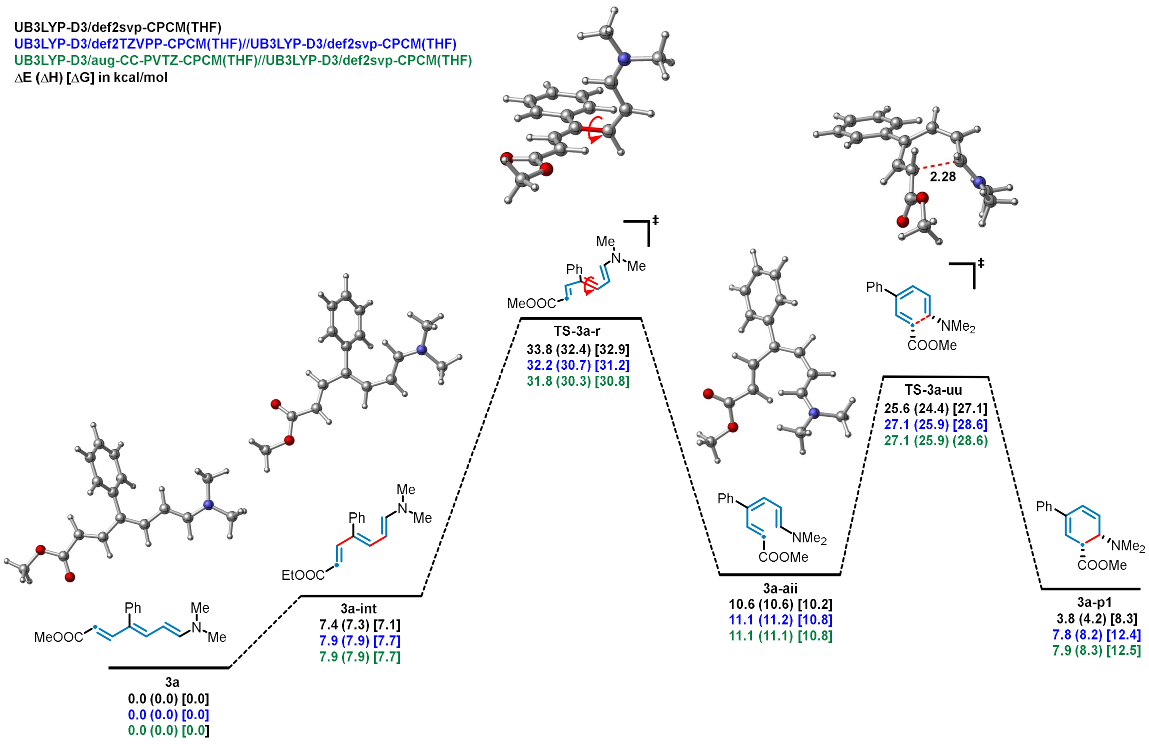

Figure S14. Minimum energy pathway for substrate **3a** under basic conditions till electrocyclization step calculated at different methods.

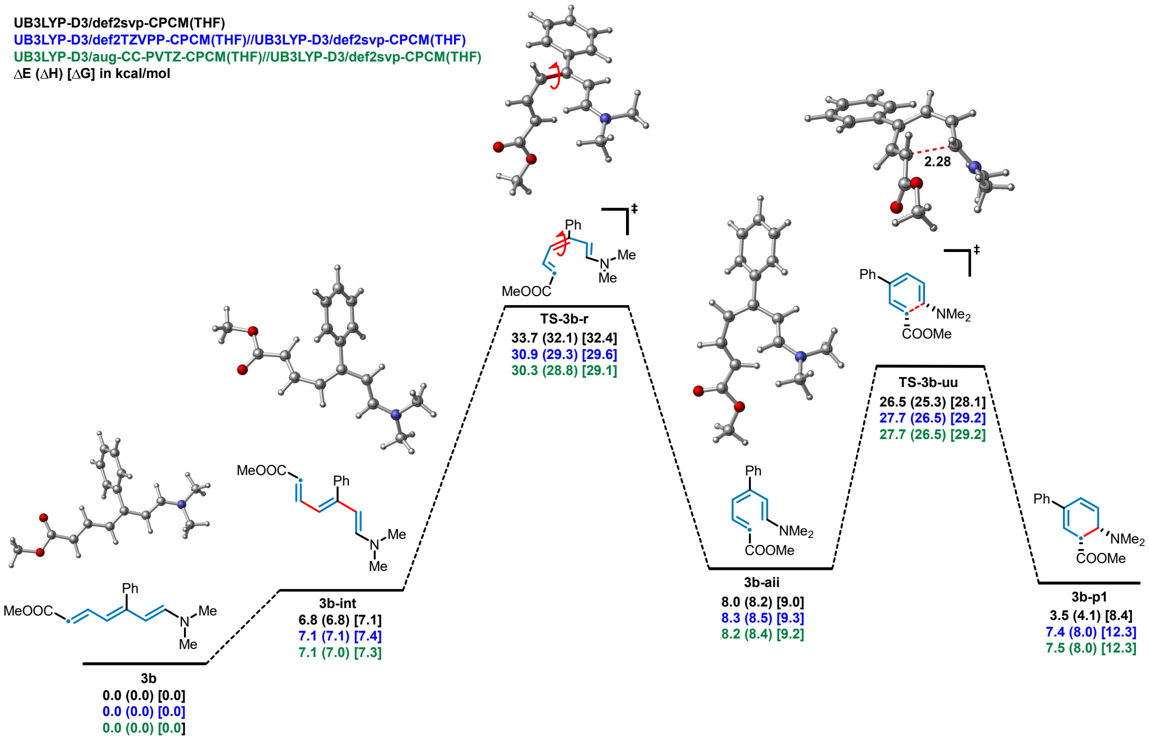

Figure S15. Minimum energy pathway for substrate **3b** under basic conditions till electrocyclization step calculated at different methods.

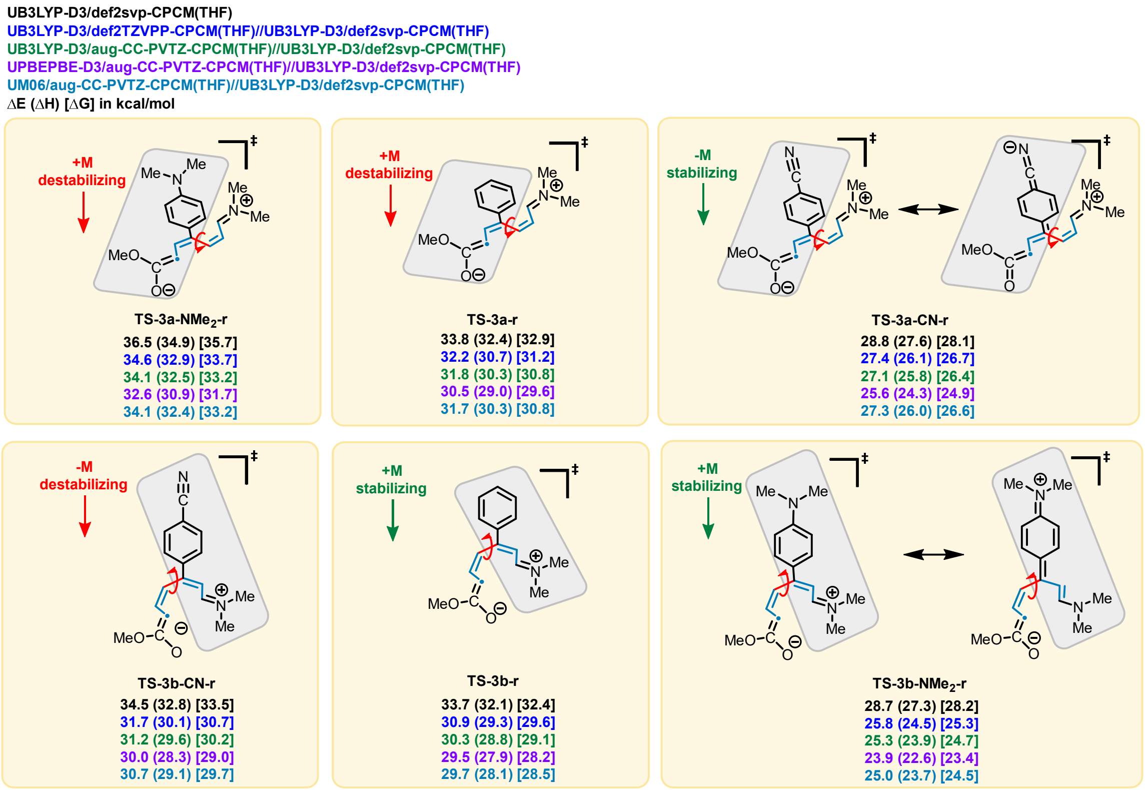

Figure S16. Probing the effect of electron donating and withdrawing groups in the key C_γ-C_δ rotation transition states for substrates **3a** and **3b** under basic conditions.

Increasing +M effect of Phenyl ring should hypothetically have destabilizing effect on **TS-3a-r** and stabilizing effect on **TS-3b-r**. Whereas increasing –M effect should stabilize **TS-3a-r** and destabilize **TS-3b-r**. A proof of concept is shown here where +M effect of phenyl ring is increased by introducing NMe2 and –M effect is increased by introducing CN group. Predicted barriers are consistent with the hypothesis.

Table 7. Cartesian coordinates (xyz format) and energies of all the structures involved in each reaction mechanism studied calculated at the UB3LYP-d3/def2-svp/CPCM(THF) level of theory.

3a

E(scf) = -825.763525522 a.u.

ν_{min} = 28.2 cm^{-1}

C	-0.152966	2.218025	0.394035	C	-4.641762	0.636588	-0.624015
C	-1.490052	2.570486	0.384228	O	-4.267301	-0.487377	-0.917740
C	-2.456947	1.591783	-0.039532	O	-5.947263	1.000132	-0.694817
H	0.080337	1.199584	0.057535	C	2.242256	2.516746	0.757333
H	-2.059395	0.604221	-0.303555	H	2.401160	1.495797	0.393730
C	-3.805383	1.745323	-0.165897	H	0.768336	4.023288	1.166674
H	-4.306323	2.687206	0.062688	C	-6.860571	-0.002968	-1.132403
C	0.955235	3.010912	0.805123	H	-6.609026	-0.354195	-2.145835

H	-6.850837	-0.871536	-0.454762	C	-1.940947	3.934210	0.795148
H	-7.854899	0.461429	-1.132355	C	-2.796052	4.109873	1.898887
N	3.368452	3.165006	1.114677	C	-1.523225	5.076809	0.088606
C	3.303884	4.527297	1.616489	C	-3.216047	5.385647	2.286404
H	2.829114	5.197374	0.879800	H	-3.130997	3.233037	2.458437
H	2.720122	4.581688	2.552127	C	-1.940907	6.354151	0.474970
H	4.318512	4.893450	1.816619	H	-0.864054	4.956593	-0.774746
C	4.669740	2.519381	1.062739	C	-2.789558	6.513378	1.575825
H	5.365404	3.087957	0.422529	H	-3.877534	5.499955	3.149330
H	5.115420	2.442831	2.069793	H	-1.605407	7.228425	-0.089322
H	4.570038	1.506288	0.651074	H	-3.117985	7.511144	1.878120

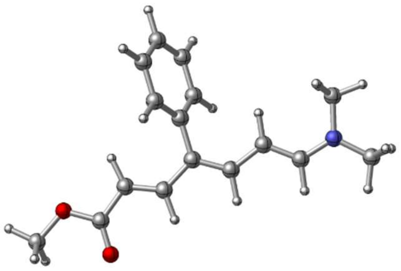

Zero-point correction= 0.316565 (Hartree/Particle)
Thermal correction to Energy= 0.336293
Thermal correction to Enthalpy= 0.337238
Thermal correction to Gibbs Free Energy= 0.265520
Sum of electronic and zero-point Energies= -825.446961
Sum of electronic and thermal Energies= -825.427232
Sum of electronic and thermal Enthalpies= -825.426288
Sum of electronic and thermal Free Energies= -825.498005

3a-aii

E(scf) = -825.746690573 a.u.

ν_{min} = 11.5 cm^{-1}

C	-0.173573	4.200421	0.503584	C	0.960023	3.439053	0.940974
C	-1.548347	4.024016	0.564765	C	-2.813644	0.412288	1.076044
C	-2.264934	2.794627	0.877222	O	-3.953522	0.530887	1.490965
H	0.127159	5.143281	0.031168	O	-2.250826	-0.798045	0.844285
H	-3.308775	2.928601	1.185443	C	0.961033	2.449742	1.900833
C	-1.877206	1.497853	0.764405	H	0.019630	2.166545	2.375464
H	-0.883914	1.204256	0.423492	H	1.918814	3.768163	0.533200

C	-3.068940	-1.937302	1.105146	H	0.853973	0.678827	3.707754
H	-3.377242	-1.968869	2.162146	C	-2.413120	5.190694	0.219312
H	-3.976324	-1.923716	0.480901	C	-3.627484	5.018645	-0.480920
H	-2.459957	-2.817670	0.864295	C	-2.052775	6.508280	0.576788
N	2.033346	1.778318	2.362742	C	-4.435294	6.108633	-0.814622
C	3.361990	2.048774	1.840049	H	-3.933338	4.017017	-0.792912
H	4.086510	1.373317	2.311507	C	-2.854988	7.599974	0.235489
H	3.395820	1.892797	0.748518	H	-1.141429	6.676070	1.155664
H	3.667092	3.089601	2.045807	C	-4.053002	7.407574	-0.461570
C	1.906809	0.780720	3.413352	H	-5.365731	5.941857	-1.364187
H	2.267529	-0.202571	3.066718	H	-2.549405	8.607259	0.531549
H	2.493034	1.067348	4.303361	H	-4.684612	8.260496	-0.722559

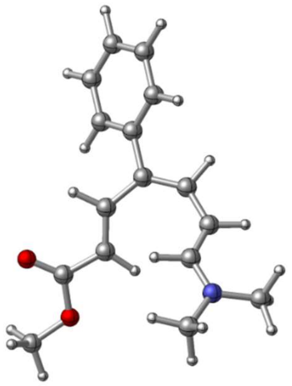

Zero-point correction= 0.316582 (Hartree/Particle)
Thermal correction to Energy= 0.336314
Thermal correction to Enthalpy= 0.337258
Thermal correction to Gibbs Free Energy= 0.265004
Sum of electronic and zero-point Energies= -825.430108
Sum of electronic and thermal Energies= -825.410377
Sum of electronic and thermal Enthalpies= -825.409433
Sum of electronic and thermal Free Energies= -825.481687

3a-int

E(scf) = -825.751749528 a.u.

ν_{min} = 22.2 cm^{-1}

C	-0.208691	1.951213	0.683882	C	-1.502571	2.411250	0.518978

C	-2.546271	1.551966	-0.002603	C	3.362785	3.671937	2.473028
H	-0.016470	0.953852	0.272126	H	4.105260	4.330641	2.940791
H	-3.484816	2.055159	-0.263723	H	3.484029	2.655750	2.888749
C	-2.535066	0.204599	-0.205788	H	3.568786	3.625159	1.391108
H	-1.684423	-0.426992	0.058137	C	1.886624	5.259527	3.696638
C	0.944934	2.534220	1.299456	H	2.194610	4.925232	4.703081
C	-3.697786	-0.469914	-0.784409	H	2.509019	6.125733	3.417486
O	-4.746369	0.049152	-1.130241	H	0.839132	5.585657	3.743963
O	-3.484899	-1.805308	-0.897897	C	-1.911580	3.814914	0.832223
C	0.949300	3.626556	2.144711	C	-2.926104	4.081778	1.768172
H	0.003430	4.102011	2.409300	C	-1.311877	4.904581	0.170989
H	1.887189	2.013876	1.113684	C	-3.316738	5.396049	2.051416
C	-4.553828	-2.571624	-1.447952	H	-3.406265	3.247667	2.286893
H	-5.463642	-2.481913	-0.833368	C	-1.698748	6.215852	0.451948
H	-4.794726	-2.239440	-2.470331	H	-0.525730	4.711135	-0.563037
H	-4.212232	-3.614398	-1.463017	C	-2.701365	6.467943	1.398207
N	2.028263	4.190658	2.720639	H	-4.103691	5.581294	2.787386

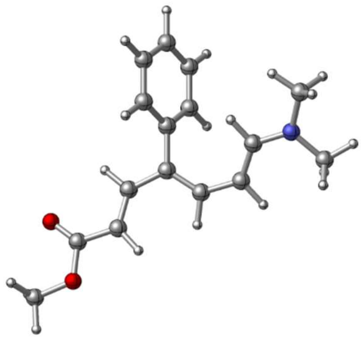

Zero-point correction= 0.316368 (Hartree/Particle)
Thermal correction to Energy= 0.336168
Thermal correction to Enthalpy= 0.337112
Thermal correction to Gibbs Free Energy= 0.265146
Sum of electronic and zero-point Energies= -825.435382
Sum of electronic and thermal Energies= -825.415582
Sum of electronic and thermal Enthalpies= -825.414638
Sum of electronic and thermal Free Energies= -825.486603

3a-p1
E(scf) = -825.757528762 a.u.
ν_{min} = 40.6 cm^{-1}

C	-1.393976	4.525141	0.936676	C	-4.407689	4.215568	2.619988
C	-1.135614	3.383502	1.834733	H	-4.368591	3.223687	3.119013
C	-1.956124	2.308280	1.760330	H	-3.440195	4.711323	2.780212
H	-1.875856	1.477710	2.464381	H	-5.183500	4.815580	3.123267
C	-3.002673	2.224344	0.690579	C	-6.059206	3.668059	0.933114
H	-2.500846	1.932722	-0.252712	H	-6.293247	2.671945	1.365487
C	-2.540430	4.642472	0.239966	H	-6.784429	4.380431	1.361849
C	-4.046104	1.160177	0.956417	H	-6.236728	3.614509	-0.151073
O	-4.233764	0.596435	2.011343	H	-0.638006	5.312421	0.872592
O	-4.771776	0.917132	-0.146590	C	-0.036344	3.471254	2.829688
C	-3.652305	3.617290	0.339384	C	0.686906	2.325449	3.218169
H	-4.102810	3.494877	-0.655778	C	0.306160	4.701825	3.425110
H	-2.744894	5.535085	-0.357438	C	1.701867	2.404159	4.174072
C	-5.835105	-0.033260	-0.023577	H	0.462370	1.365402	2.747836
H	-5.448508	-1.010570	0.301420	C	1.322665	4.781300	4.381219
H	-6.581077	0.313213	0.708212	H	-0.243564	5.606335	3.154530
H	-6.290318	-0.114029	-1.017716	C	2.025290	3.633075	4.761184
N	-4.706660	4.123644	1.202855	H	2.251740	1.501835	4.454331

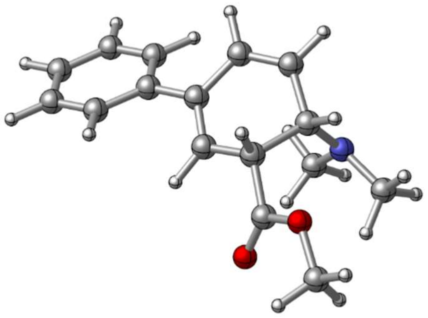

Zero-point correction= 0.319044 (Hartree/Particle)
Thermal correction to Energy= 0.336971
Thermal correction to Enthalpy= 0.337915
Thermal correction to Gibbs Free Energy= 0.272811
Sum of electronic and zero-point Energies= -825.438484
Sum of electronic and thermal Energies= -825.420558
Sum of electronic and thermal Enthalpies= -825.419614
Sum of electronic and thermal Free Energies= -825.484718

3b
E(scf) = -825.761853779 a.u.
ν_{min} = 24.1 cm^{-1}

C	0.190017	2.624312	-0.750587	C	-1.179429	2.482494	-0.881058

C	-2.041593	1.823772	0.046585	H	1.755939	5.302180	-3.786216
H	-1.593803	1.372441	0.938195	H	3.312359	4.890994	-4.561406
C	-3.394212	1.698709	-0.081977	C	4.472162	4.440949	-2.372995
H	-3.931797	2.119976	-0.935339	H	5.130767	3.991926	-3.135549
C	0.962979	3.252476	-1.788425	H	4.633445	5.533495	-2.381674
C	-4.181011	0.992023	0.927062	H	4.770622	4.056411	-1.388606
O	-3.755298	0.461102	1.940405	H	-1.645467	2.892543	-1.784683
O	-5.500972	0.984057	0.611554	C	0.888398	2.127049	0.475976
C	2.307231	3.534850	-1.688491	C	0.649722	2.719329	1.727984
H	2.836852	3.280652	-0.766646	C	1.798170	1.057486	0.398299
H	0.417654	3.537808	-2.691213	C	1.301425	2.253101	2.873915
C	-6.366137	0.320607	1.529821	H	-0.053281	3.552747	1.798071
H	-6.095651	-0.742351	1.633228	C	2.445164	0.586907	1.544413
H	-6.318715	0.786084	2.527242	H	1.991235	0.589722	-0.570155
H	-7.380678	0.411451	1.121367	C	2.199737	1.184473	2.785897
N	3.080614	4.111830	-2.632763	H	1.105351	2.726647	3.839326
C	2.515817	4.509932	-3.909989	H	3.142423	-0.251415	1.467825
H	2.035313	3.653281	-4.412041	H	2.706963	0.817791	3.681898

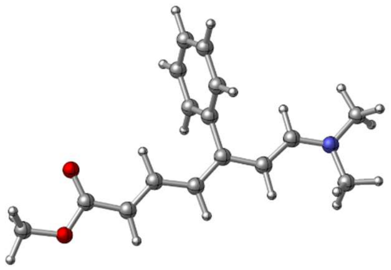

Zero-point correction= 0.316297 (Hartree/Particle)
Thermal correction to Energy= 0.336109
Thermal correction to Enthalpy= 0.337054
Thermal correction to Gibbs Free Energy= 0.265006
Sum of electronic and zero-point Energies= -825.445557
Sum of electronic and thermal Energies= -825.425744
Sum of electronic and thermal Enthalpies= -825.424800
Sum of electronic and thermal Free Energies= -825.496847

3b-aii

E(scf) = -825.749051404 a.u.

ν_{min} = 29.7 cm^{-1}

C	-0.893350	4.880610	1.559728	C	-0.244305	3.654799	1.535693

C	-0.629296	2.347506	2.006757	H	-5.075655	6.382819	1.668084
H	0.132145	1.578129	1.826626	H	-6.243244	5.229887	2.378995
C	-1.706302	1.895917	2.712260	C	-5.514035	3.473435	0.694347
H	-2.556848	2.522218	2.977363	H	-6.281530	3.047412	1.360437
C	-2.311965	5.096148	1.750487	H	-6.027272	4.038181	-0.105027
C	-1.752211	0.508277	3.177325	H	-4.958230	2.646786	0.231867
O	-0.928430	-0.365670	2.963310	H	0.784000	3.680957	1.163819
O	-2.872969	0.275753	3.907142	C	-0.070455	6.109585	1.352357
C	-3.268205	4.231033	1.278961	C	1.240074	6.211014	1.862884
H	-2.945473	3.360653	0.701230	C	-0.590659	7.212497	0.644634
H	-2.615765	6.045877	2.196277	C	2.004057	7.362816	1.662208
C	-3.032815	-1.045928	4.417037	H	1.652859	5.385111	2.446306
H	-3.079180	-1.783383	3.599925	C	0.173879	8.363482	0.440195
H	-2.196795	-1.319161	5.080323	H	-1.600139	7.153424	0.231756
H	-3.975031	-1.049592	4.979646	C	1.475428	8.444002	0.947842
N	-4.603918	4.322722	1.445746	H	3.014434	7420798	2.075367
C	-5.176520	5.420930	2.203688	H	-0.247266	9.200632	-0.122682
H	-4.673472	5.518198	3.178842	H	2.072691	9.346039	0.792131

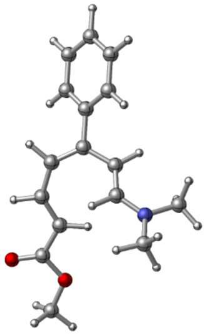

Zero-point correction= 0.316777 (Hartree/Particle)
Thermal correction to Energy= 0.336358
Thermal correction to Enthalpy= 0.337302
Thermal correction to Gibbs Free Energy= 0.266572
Sum of electronic and zero-point Energies= -825.432274
Sum of electronic and thermal Energies= -825.412693
Sum of electronic and thermal Enthalpies= -825.411749
Sum of electronic and thermal Free Energies= -825.482479

3b-int

E(scf) = -825.750949451 a.u.

ν_{min} = 22.7 cm^{-1}

C	-2.274478	4.252784	1.252468	H	-5.138204	7.389518	-0.079179
C	-2.303673	2.869232	1.261230	H	-4.243597	6.683457	-1.454852
C	-1.542640	1.934934	2.048135	H	-5.999755	6.973647	-1.589657
H	-1.671765	0.885403	1.755750	C	-6.849552	4.855055	-0.473211
C	-0.725874	2.123651	3.124142	H	-7.501797	5.451686	0.190439
H	-0.525858	3.104964	3.552306	H	-7.243931	4.930788	-1.499324
C	-3.272043	5.068091	0.597612	H	-6.904144	3.804882	-0.155872
C	-0.080697	0.980908	3.772509	H	-3.030453	2.394134	0.595686
O	-0.165626	-0.191952	3.446631	C	-1.165246	5.018815	1.897900
O	0.663666	1.378639	4.835589	C	0.168394	4.817812	1.499763
C	-4.532476	4.629417	0.260066	C	-1.437272	5.981413	2.884716
H	-4.847895	3.627570	0.568140	C	1.204148	5.547764	2.085217
H	-3.000248	6.108761	0.410250	H	0.385655	4.073902	0.729887
C	1.341704	0.350719	5.553362	C	-0.398896	6.703924	3.481881
H	0.629281	-0.380298	5.967795	H	-2.471711	6.152162	3.193227
H	2.048112	-0.188310	4.902136	C	0.924675	6.488867	3.084281
H	1.885580	0.847292	6.366977	H	2.235228	5.381896	1.762350
N	-5.472276	5.318232	-0.422924	H	-0.625533	7.438767	4.258767
C	-5.198979	6.659426	-0.907358	H	1.736402	7.055961	3.547048

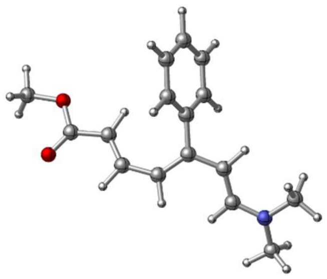

Zero-point correction= 0.316329 (Hartree/Particle)
Thermal correction to Energy= 0.336064
Thermal correction to Enthalpy= 0.337008
Thermal correction to Gibbs Free Energy= 0.265442
Sum of electronic and zero-point Energies= -825.434621
Sum of electronic and thermal Energies= -825.414885
Sum of electronic and thermal Enthalpies= -825.413941
Sum of electronic and thermal Free Energies= -825.485507

3b-p1

E(scf) = -825.756268226 a.u.

ν_{min} = 38.5 cm^{-1}

C	-1.523942	4.619074	1.229361	H	-4.774392	3.400492	3.086952
C	-1.404978	3.505725	2.188748	H	-3.813136	4.872823	2.773163
C	-2.181687	2.412497	2.091594	H	-5.587091	4.979609	2.874491
H	-2.126205	1.604340	2.824277	C	-6.160297	3.686182	0.666010
C	-3.104150	2.253044	0.919154	H	-6.457654	2.722792	1.132140
H	-2.497492	1.892149	0.066396	H	-6.934460	4.423946	0.937976
C	-2.589203	4.658372	0.390439	H	-6.186597	3.553957	-0.425676
C	-4.183112	1.213954	1.135595	H	-0.670606	3.583683	2.993291
O	-4.515027	0.746942	2.202141	C	-0.508539	5.707188	1.242294
O	-4.757420	0.871329	-0.028550	C	0.046313	6.174716	2.449737
C	-3.691750	3.620603	0.404546	C	-0.084402	6.309952	0.041632
H	-4.006656	3.439313	-0.633407	C	0.977320	7.217193	2.457043
H	-2.763250	5.529932	-0.245691	H	-0.269728	5.737359	3.399444
C	-5.835894	-0.067828	0.034893	C	0.847490	7.350524	0.048197
H	-5.505447	-1.009883	0.497014	H	-0.478267	5.942716	-0.908895
H	-6.671261	0.341154	0.623901	C	1.382707	7.810277	1.256750
H	-6.152824	-0.240779	-1.000346	H	1.385546	7.569963	3.407766
N	-4.856020	4.166746	1.086130	H	1.165155	7.798037	-0.897244
C	-4.749614	4.356004	2.520845	H	2.114649	8.621879	1.262465

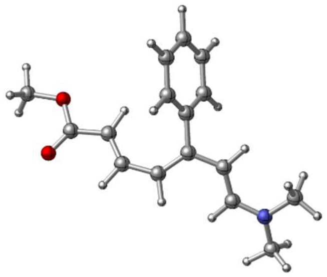

Zero-point correction= 0.319005 (Hartree/Particle)
Thermal correction to Energy= 0.336951
Thermal correction to Enthalpy= 0.337895
Thermal correction to Gibbs Free Energy= 0.272782
Sum of electronic and zero-point Energies= -825.437264
Sum of electronic and thermal Energies= -825.419317
Sum of electronic and thermal Enthalpies= -825.418373
Sum of electronic and thermal Free Energies= -825.483487

TS-3a-r
E(scf) = -825.709631147 a.u.
ν_{min} = -511.0 cm^{-1}

C	-0.287755	3.316329	-0.435546	C	2.970572	2.677126	2.277928
C	-1.682626	3.437751	0.053349	H	3.571048	2.645601	3.193524
C	-2.398208	2.251729	0.208041	H	3.190725	1.785728	1.672448
H	-0.099146	3.379086	-1.519121	H	3.231575	3.579620	1.709489
H	-3.451115	2.332174	0.499715	C	1.223219	2.448001	4.047623
C	-1.941654	0.937062	0.058786	H	1.620718	1.464782	4.340663
H	-0.909018	0.712049	-0.214272	H	1.688989	3.222578	4.674698
C	0.800413	3.094336	0.353161	H	0.136627	2.461453	4.191336
C	-2.823761	-0.171263	0.274827	C	-2.214084	4.776571	0.288969
O	-4.012428	-0.141433	0.596283	C	-3.537507	5.011326	0.755364
O	-2.186576	-1.380036	0.086764	C	-1.418607	5.932678	0.059454
C	0.607738	2.901765	1.756962	C	-4.019213	6.299926	0.972494
H	-0.425546	2.910500	2.118745	H	-4.198869	4.166364	0.956390
H	1.796190	3.012669	-0.085989	C	-1.907212	7.221672	0.280707
C	-2.980370	-2.539022	0.280120	H	-0.392109	5.816882	-0.298599
H	-3.376056	-2.596416	1.308330	C	-3.213344	7.425019	0.739744
H	-3.840138	-2.564826	-0.410497	H	-5.044060	6.429951	1.333354
H	-2.328309	-3.402599	0.088229	H	-1.255258	8.079347	0.088863
N	1.546115	2.699784	2.640754	H	-3.596579	8.433498	0.913079

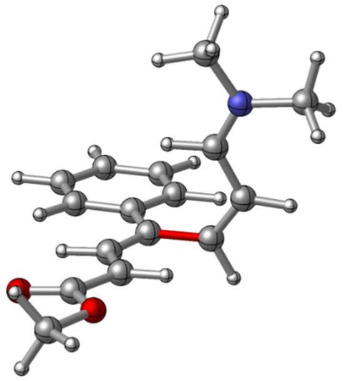

Zero-point correction= 0.314604 (Hartree/Particle)
Thermal correction to Energy= 0.333973
Thermal correction to Enthalpy= 0.334917
Thermal correction to Gibbs Free Energy= 0.263999
Sum of electronic and zero-point Energies= -825.395027

Sum of electronic and thermal Energies= -825.375658
Sum of electronic and thermal Enthalpies= -825.374714
Sum of electronic and thermal Free Energies= -825.445632

TS-3a-uu

E(scf) = -825.722678629 a.u.

ν_{min} =-268.29 cm^{-1}

C	-1.495684	4.504547	1.301991	H	-5.808306	4.237914	3.086542
C	-1.038586	3.316276	1.974658	H	-4.243093	5.064986	2.813453
C	-1.834935	2.176127	1.964462	H	-5.764527	5.811701	2.242369
H	-1.787844	1.474242	2.805855	C	-6.293145	3.561027	0.576351
C	-2.781768	1.943295	0.946054	H	-6.718103	2.833805	1.290341
H	-2.471224	2.141071	-0.078954	H	-7.052515	4.338402	0.388437
C	-2.751051	4.792529	0.815485	H	-6.073633	3.039463	-0.363287
C	-3.840507	0.955502	1.121064	H	-0.824250	5.366975	1.357438
O	-4.151745	0.402581	2.162566	C	0.153994	3.384376	2.855392
O	-4.522771	0.745285	-0.038814	C	0.918313	2.222200	3.100279
C	-3.876259	3.905138	0.563797	C	0.562704	4.575613	3.494737
H	-3.943018	3.381362	-0.386366	C	2.027199	2.245119	3.949708
H	-3.004777	5.858451	0.744064	H	0.647200	1.291792	2.595630
C	-5.635566	-0.142548	0.033334	C	1.679004	4.602197	4.333831
H	-5.326772	-1.139679	0.383378	H	-0.016102	5.491611	3.357828
H	-6.404394	0.243234	0.722390	C	2.417921	3.436926	4.569684
H	-6.045012	-0.209981	-0.982516	H	2.598696	1.327924	4.116343
N	-5.079640	4.154375	1.115348	H	1.966274	5.539379	4.818422
C	-5.229293	4.856743	2.379736	H	3.289991	3.458680	5.228135

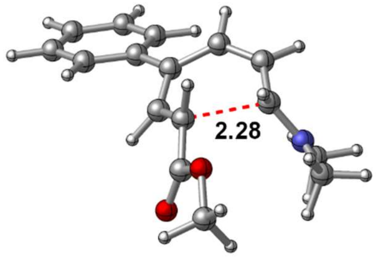

Zero-point correction= 0.315729 (Hartree/Particle)
Thermal correction to Energy= 0.334345
Thermal correction to Enthalpy= 0.335289
Thermal correction to Gibbs Free Energy= 0.267895
Sum of electronic and zero-point Energies= -825.406950

Sum of electronic and thermal Energies= -825.388333
Sum of electronic and thermal Enthalpies= -825.387389
Sum of electronic and thermal Free Energies= -825.454784

TS-3b-r
E(scf) = -825.708129187 a.u.
ν_{min} = -616.9 cm^{-1}

C	-1.535890	4.847069	0.936048	H	-5.225962	6.626015	0.332797
C	-1.126704	3.523600	0.402169	H	-6.795428	5.822143	0.610691
C	-1.148623	2.380361	1.164573	H	-5.631284	5.985947	1.957883
H	-0.809618	1.455633	0.680123	C	-6.094965	3.454261	0.158518
C	-1.587200	2.227105	2.499703	H	-6.777039	3.272597	1.003378
H	-1.953104	3.082146	3.073254	H	-6.689029	3.755518	-0.717544
C	-2.866816	5.223912	0.942148	H	-5.548196	2.531588	-0.069332
C	-1.571211	0.955959	3.135626	H	-0.804497	3.498913	-0.643250
O	-1.207496	-0.131315	2.673297	C	-0.502374	5.751852	1.484249
O	-2.043321	1.019419	4.441174	C	0.748398	5.221167	1.869589
C	-3.859818	4.298685	0.536290	C	-0.710378	7.142552	1.632738
H	-3.517871	3.303837	0.235835	C	1.737774	6.040296	2.411953
H	-3.173225	6.190755	1.341508	H	0.918737	4.150649	1.747472
C	-2.061311	-0.203626	5.151874	C	0.289190	7.963782	2.152097
H	-2.706637	-0.955342	4.664866	H	-1.649768	7.596397	1.312468
H	-1.053097	-0.644020	5.241884	C	1.514653	7.415442	2.551349
H	-2.453214	0.018729	6.155061	H	2.691659	5.606156	2.721174
N	-5.154270	4.518749	0.508082	H	0.114504	9.038624	2.242670
C	-5.731927	5.815455	0.875055	H	2.295595	8.060233	2.962186

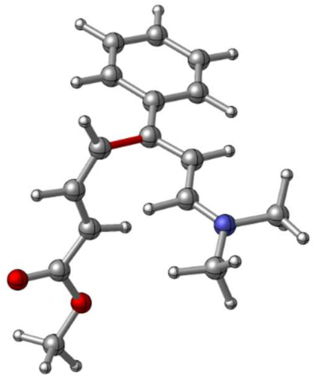

Zero-point correction= 0.314159 (Hartree/Particle)
Thermal correction to Energy= 0.333604
Thermal correction to Enthalpy= 0.334548
Thermal correction to Gibbs Free Energy= 0.263025
Sum of electronic and zero-point Energies= -825.393970
Sum of electronic and thermal Energies= -825.374525
Sum of electronic and thermal Enthalpies= -825.373581
Sum of electronic and thermal Free Energies= -825.445104

TS-3b-uu

E(scf) = -825.719525678 a.u.

ν_{min} = -292.22 cm^{-1}

C	-1.422207	4.469523	1.262841	H	-5.858497	4.350218	2.953609
C	-1.050753	3.254791	1.934769	H	-4.237883	5.082347	2.744770
C	-1.856240	2.138812	1.988745	H	-5.684457	5.904458	2.090813
H	-1.771928	1.447589	2.836894	C	-6.254479	3.631609	0.447019
C	-2.838093	1.891962	1.000525	H	-6.743678	2.944641	1.160183
H	-2.533585	2.014737	-0.037058	H	-6.971824	4.432910	0.200716
C	-2.694107	4.757677	0.778512	H	-6.016730	3.071576	-0.465742
C	-3.927736	0.954629	1.242733	H	-0.162511	3.296482	2.569903
O	-4.238969	0.465902	2.316173	C	-0.456403	5.612467	1.302562
O	-4.641882	0.715042	0.107875	C	0.325462	5.896350	2.439683
C	-3.824932	3.889596	0.518610	C	-0.319333	6.453417	0.180951
H	-3.879914	3.343473	-0.419172	C	1.207850	6.979433	2.453586
H	-2.952787	5.823688	0.751424	H	0.225524	5.279466	3.335231
C	-5.791171	-0.115824	0.247735	C	0.565620	7.535508	0.192317
H	-5.519698	-1.108513	0.639110	H	-0.904805	6.240031	-0.716466
H	-6.524687	0.337449	0.934333	C	1.334122	7.803656	1.329472
H	-6.228258	-0.212651	-0.754161	H	1.795374	7.185346	3.352183
N	-5.042100	4.189452	1.022262	H	0.658853	8.167788	-0.694602
C	-5.212285	4.922239	2.265601	H	2.027205	8.648700	1.340639

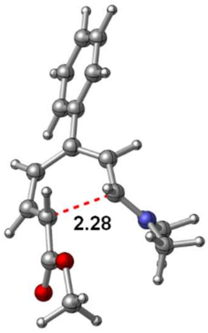

Zero-point correction= 0.315467 (Hartree/Particle)
Thermal correction to Energy= 0.334150
Thermal correction to Enthalpy= 0.335094
Thermal correction to Gibbs Free Energy= 0.267470
Sum of electronic and zero-point Energies= -825.404059
Sum of electronic and thermal Energies= -825.385376
Sum of electronic and thermal Enthalpies= -825.384431
Sum of electronic and thermal Free Energies= -825.452055

3a-H
E(scf) = -826.181186664 a.u.
ν_{min} = 28.74 cm^{-1}

C	-0.100630	2.121677	0.156164	C	3.186094	4.749639	1.227306
C	-1.414551	2.480055	0.270974	H	2.600026	5.291182	0.476625
C	-2.430593	1.511614	-0.126944	H	2.633451	4.675952	2.170438
H	0.131264	1.132252	-0.250337	H	4.143917	5.254345	1.395240
H	-2.073149	0.522098	-0.431991	C	4.334006	2.596645	1.692239
C	-3.762562	1.726257	-0.172166	H	5.252871	3.166998	1.875443
H	-4.211911	2.682400	0.100990	H	3.765201	2.465830	2.620092
C	1.021744	2.948595	0.545371	H	4.575716	1.621853	1.252566
C	-4.679299	0.649587	-0.612296	C	-1.832527	3.824730	0.764388
O	-4.347135	-0.470899	-0.943401	C	-2.597964	3.950874	1.937778
O	-5.954827	1.070811	-0.602892	C	-1.468357	4.987801	0.062812
C	2.292326	2.573980	0.328944	C	-2.977676	5.211914	2.403817
H	2.569680	1.635441	-0.154477	H	-2.888780	3.054312	2.490004
H	0.792890	3.899527	1.029379	C	-1.854033	6.248721	0.527589
C	-6.944789	0.121905	-1.012593	H	-0.887706	4.898542	-0.858593
H	-6.761951	-0.209383	-2.046272	C	-2.607596	6.363818	1.700023
H	-6.934520	-0.760015	-0.354012	H	-3.565275	5.295470	3.321328
H	-7.910491	0.636512	-0.943665	H	-1.567707	7.143081	-0.031248
N	3.472675	3.367606	0.722312	H	-2.909134	7.349071	2.063941

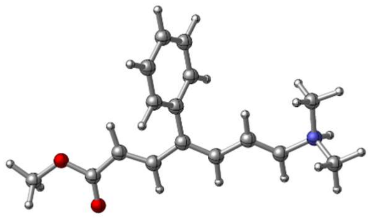

Zero-point correction= 0.332185 (Hartree/Particle)
Thermal correction to Energy= 0.351576
Thermal correction to Enthalpy= 0.352520

Thermal correction to Gibbs Free Energy= 0.282131
Sum of electronic and zero-point Energies= -825.849002
Sum of electronic and thermal Energies= -825.829611
Sum of electronic and thermal Enthalpies= -825.828667
Sum of electronic and thermal Free Energies= -825.899056

3a-H-aii

E(scf) = -826.167461541 a.u.

ν_{min} = 27.35 cm^{-1}

C	-0.169656	3.157630	0.683134	C	2.591542	-0.208678	0.981846
C	-1.530105	3.184716	0.707329	H	3.137063	-1.134706	1.195941
C	-2.376305	1.974035	0.790506	H	2.183226	-0.237112	-0.034334
H	0.348627	4.100267	0.482271	H	3.256852	0.652395	1.109304
H	-3.178044	1.978814	1.538900	C	1.966547	-0.128698	3.382416
C	-2.296536	0.913914	-0.029881	H	2.553914	-1.042914	3.529268
H	-1.555628	0.856258	-0.830461	H	2.586594	0.761492	3.539010
C	0.733851	2.022620	0.880179	H	1.107138	-0.117384	4.062852
C	-3.222270	-0.234198	0.126597	C	-2.268404	4.473869	0.624005
O	-4.092128	-0.329264	0.967654	C	-3.532964	4.526097	0.003983
O	-2.974592	-1.179862	-0.794216	C	-1.737321	5.664124	1.161635
C	0.540043	1.033728	1.759272	C	-4.232091	5.731222	-0.095828
H	-0.329198	0.970603	2.414444	H	-3.965339	3.617801	-0.421876
H	1.652286	2.057728	0.287697	C	-2.437914	6.866980	1.062885
C	-3.808223	-2.343447	-0.755950	H	-0.779020	5.643170	1.685116
H	-3.711068	-2.859952	0.211230	C	-3.686809	6.906356	0.431330
H	-4.863996	-2.069890	-0.903705	H	-5.206871	5.751914	-0.589191
H	-3.466317	-2.993015	-1.570342	H	-2.012076	7.777330	1.491969
N	1.462656	-0.101344	1.961091	H	-4.235682	7.848373	0.357971

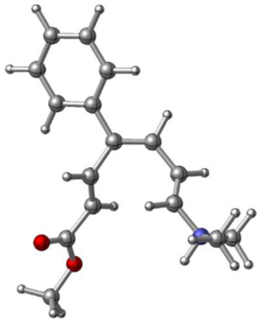

Zero-point correction= 0.331809 (Hartree/Particle)
Thermal correction to Energy= 0.351290
Thermal correction to Enthalpy= 0.352234
Thermal correction to Gibbs Free Energy= 0.281721
Sum of electronic and zero-point Energies= -825.835652
Sum of electronic and thermal Energies= -825.816172
Sum of electronic and thermal Enthalpies= -825.815228
Sum of electronic and thermal Free Energies= -825.885740

3a-H-int

E(scf) = -826.170479003 a.u.

ν_{min} = 30.83 cm^{-1}

C	-0.534773	0.856537	0.206425
C	-1.654864	1.626429	0.115023
C	-2.811524	1.189627	-0.680483
H	-0.476105	-0.035915	-0.423725
H	-3.446712	1.979792	-1.095557
C	-3.185731	-0.085242	-0.905978
H	-2.652502	-0.938037	-0.479702
C	0.646855	1.089526	1.037210
C	-4.377687	-0.384796	-1.735342
O	-5.095241	0.438960	-2.264671
O	-4.575823	-1.709944	-1.828823
C	0.610801	1.642941	2.255234
H	-0.308854	1.972566	2.738890
H	1.592796	0.735721	0.618358
C	-5.701852	-2.135054	-2.603771
H	-6.637151	-1.738272	-2.180284
H	-5.611679	-1.787197	-3.644157
H	-5.703078	-3.230877	-2.567999
N	1.798231	1.946723	3.076098
C	3.092720	1.362459	2.599365
H	3.862818	1.590510	3.345139
H	2.978108	0.277768	2.495821
H	3.357391	1.818292	1.638765
C	1.908521	3.438546	3.283248
H	2.723267	3.634892	3.990500
H	2.113568	3.901102	2.310849
H	0.957952	3.811128	3.682609
C	-1.786067	2.967560	0.759943
C	-2.864848	3.237055	1.618798
C	-0.846284	3.983763	0.509324
C	-2.982174	4.484075	2.241925
H	-3.608165	2.459206	1.811592
C	-0.968111	5.230319	1.125410
H	-0.014696	3.787453	-0.171293
C	-2.032566	5.481806	2.000527
H	-3.820123	4.675961	2.916529
H	-0.232204	6.011500	0.918925
H	-2.126107	6.457126	2.484328

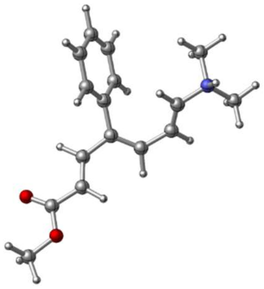

Zero-point correction= 0.331925 (Hartree/Particle)
Thermal correction to Energy= 0.351354
Thermal correction to Enthalpy= 0.352298
Thermal correction to Gibbs Free Energy= 0.282042
Sum of electronic and zero-point Energies= -825.838554
Sum of electronic and thermal Energies= -825.819125
Sum of electronic and thermal Enthalpies= -825.818181
Sum of electronic and thermal Free Energies= -825.888437

3a-H-p1

E(scf) = -826.202040660 a.u.

ν_{min} = 40.68 cm^{-1}

C	-1.379254	4.532644	0.920408	H	-4.357931	3.172361	3.009820
C	-1.158093	3.400937	1.839799	H	-3.430730	4.703947	2.818643
C	-1.998861	2.344747	1.756659	H	-5.184249	4.757602	3.202198
H	-1.923383	1.494842	2.436279	C	-6.092193	3.643709	0.959184
C	-3.048624	2.280473	0.680948	H	-6.151146	2.652108	1.417586
H	-2.545163	1.954861	-0.250211	H	-6.832499	4.300629	1.430943
C	-2.502768	4.669919	0.190984	H	-6.275134	3.583522	-0.119292
C	-4.124483	1.236054	0.948204	H	-0.597241	5.291000	0.837825
O	-4.392712	0.784603	2.038035	C	-0.055618	3.472097	2.831509
O	-4.758497	0.911089	-0.178075	C	0.640486	2.313107	3.226838
C	-3.615018	3.656961	0.276868	C	0.308237	4.701741	3.413949
H	-4.119088	3.557582	-0.692086	C	1.656887	2.380256	4.181535
H	-2.650939	5.532101	-0.464124	H	0.398057	1.353585	2.764631
C	-5.821918	-0.052654	-0.078827	C	1.324788	4.767877	4.370584
H	-5.445491	-0.993210	0.347374	H	-0.220972	5.616288	3.135456
H	-6.631448	0.332101	0.559276	C	2.002792	3.607809	4.758404
H	-6.183743	-0.208716	-1.100927	H	2.189489	1.470358	4.469137
N	-4.729268	4.223204	1.204469	H	1.585693	5.730796	4.816598
C	-4.396669	4.212375	2.668023	H	2.801187	3.660131	5.502620

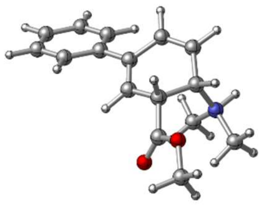

Zero-point correction= 0.335181 (Hartree/Particle)
Thermal correction to Energy= 0.353021
Thermal correction to Enthalpy= 0.353965
Thermal correction to Gibbs Free Energy= 0.289026
Sum of electronic and zero-point Energies= -825.866860
Sum of electronic and thermal Energies= -825.849020
Sum of electronic and thermal Enthalpies= -825.848076
Sum of electronic and thermal Free Energies= -825.913015

3a-OH'

E(scf) = -826.190534011 a.u.

ν_{min} = 28.85 cm^{-1}

C	-0.116021	2.185927	0.381171	C	3.253286	4.535185	1.659827
C	-1.482376	2.537235	0.356773	H	2.805412	5.201375	0.906399
C	-2.407354	1.574156	-0.067462	H	2.638736	4.568517	2.572915
H	0.131063	1.173181	0.039960	H	4.260009	4.896836	1.897619
H	-1.975334	0.601252	-0.334595	C	4.667124	2.562146	1.078972
C	-3.790810	1.743338	-0.187946	H	5.320680	3.158901	0.423369
H	-4.257010	2.698500	0.053099	H	5.118380	2.523875	2.082961
C	0.941585	2.990215	0.802755	H	4.595515	1.542659	0.680179
C	-4.666864	0.746410	-0.615679	C	-1.928897	3.901350	0.773813
O	-4.353689	-0.491954	-0.951866	C	-2.709815	4.078292	1.929242
O	-5.942651	1.031575	-0.703604	C	-1.572692	5.032775	0.018924
C	2.255390	2.507069	0.771388	C	-3.124635	5.354912	2.320214
H	2.431810	1.490804	0.404460	H	-2.990263	3.206374	2.525469
H	0.734524	3.999718	1.159633	C	-1.986555	6.309486	0.410110
C	-6.882979	0.031132	-1.139476	H	-0.968524	4.907123	-0.883096
H	-6.641297	-0.303569	-2.158223	C	-2.763723	6.473915	1.561927
H	-6.873119	-0.828426	-0.454475	H	-3.729685	5.475937	3.222298
H	-7.860862	0.523889	-1.120826	H	-1.703698	7.178662	-0.189108
N	3.340495	3.167755	1.148860	H	-3.087734	7.471850	1.867620

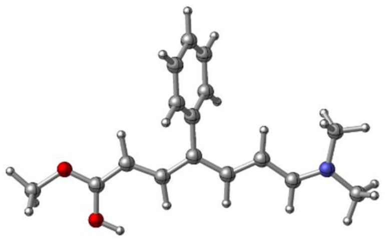

Zero-point correction= 0.329657 (Hartree/Particle)
Thermal correction to Energy= 0.349534
Thermal correction to Enthalpy= 0.350478
Thermal correction to Gibbs Free Energy= 0.279080
Sum of electronic and zero-point Energies= -825.860877
Sum of electronic and thermal Energies= -825.841000
Sum of electronic and thermal Enthalpies= -825.840056
Sum of electronic and thermal Free Energies= -825.911454

3a-OH-aii'
E(scf) = -826.172422162 a.u.
ν_{min} = 33.33 cm^{-1}

C	-0.198834	4.045906	0.175964	N	1.796037	1.874445	2.393536
C	-1.600991	3.874786	0.415434	C	3.098936	1.752120	1.735781
C	-2.233391	2.650286	0.671641	H	3.666953	0.945002	2.212432
H	0.049414	4.854900	-0.521777	H	2.967204	1.515939	0.670880
H	-3.307884	2.752181	0.866713	H	3.666296	2.691528	1.830157
C	-1.742310	1.334357	0.604799	C	1.642913	1.209115	3.686517
H	-0.709626	1.122694	0.329902	H	1.810706	0.127821	3.569828
C	0.896963	3.377823	0.705933	H	2.382451	1.606211	4.399183
C	-2.524045	0.209085	0.828533	H	0.633837	1.380609	4.080254
O	-3.789811	0.182961	1.217552	C	-2.446315	5.092529	0.254710
O	-1.982958	-0.977522	0.654189	C	-3.726976	5.043295	-0.334602
C	0.822121	2.593100	1.873640	C	-1.968419	6.346247	0.689087
H	-0.115517	2.588503	2.435866	C	-4.501662	6.197208	-0.469822
H	1.873021	3.571038	0.257211	H	-4.112415	4.096248	-0.719922
C	-2.756145	-2.166521	0.894340	C	-2.739272	7.502610	0.544556
H	-3.098677	-2.200464	1.938605	H	-0.989007	6.411975	1.169988
H	-3.623879	-2.205540	0.220420	C	-4.011560	7.433050	-0.032100
H	-2.077951	-3.001923	0.688871	H	-5.488752	6.133112	-0.934756

H	-2.347555	8.460900	0.895013	H	-4.616957	8.336001	-0.142536

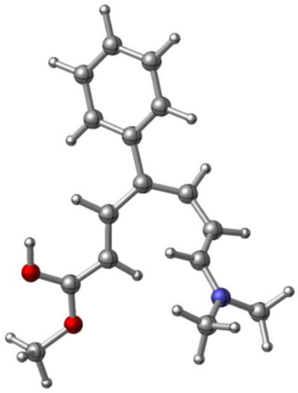

Zero-point correction= 0.329684 (Hartree/Particle)
Thermal correction to Energy= 0.349435
Thermal correction to Enthalpy= 0.350379
Thermal correction to Gibbs Free Energy= 0.279735
Sum of electronic and zero-point Energies= -825.842738
Sum of electronic and thermal Energies= -825.822987
Sum of electronic and thermal Enthalpies= -825.822043
Sum of electronic and thermal Free Energies= -825.892687

3a-OH-int'
E(scf) = -826.179621676 a.u.
ν_{min} = 23.47 cm^{-1}

C	-0.214486	1.905558	0.879927	C	-4.446254	-2.707820	-1.275786
C	-1.538248	2.363624	0.678244	H	-5.299492	-2.694822	-0.582927
C	-2.537570	1.495119	0.210803	H	-4.763785	-2.338410	-2.261260
H	-0.017088	0.891226	0.520369	H	-4.038960	-3.720979	-1.360718
H	-3.507112	1.986106	0.058935	N	1.992990	4.311948	2.667695
C	-2.468143	0.122357	-0.064265	C	3.317096	3.694342	2.592290
H	-1.558403	-0.454724	0.100113	H	3.323525	2.729078	3.121849
C	0.906643	2.510494	1.455622	H	3.604984	3.527279	1.543499
C	-3.540444	-0.620673	-0.552220	H	4.052003	4.359113	3.059857
O	-4.751589	-0.174756	-0.836138	C	1.890501	5.585973	3.375939
O	-3.367726	-1.904309	-0.761168	H	2.205375	5.461133	4.423814
C	0.926679	3.744875	2.128097	H	2.543375	6.333498	2.899139
H	-0.001011	4.303108	2.253599	H	0.854589	5.946059	3.350634
H	1.833263	1.936655	1.405103	C	-1.932953	3.785550	0.930660

C	-2.784506	4.120214	1.996527	H	-0.791754	4.563044	-0.735060
C	-1.452980	4.813840	0.098285	C	-2.641727	6.468952	1.411623
C	-3.135835	5.453820	2.236501	H	-3.794694	5.699099	3.073259
H	-3.161348	3.329311	2.650158	H	-1.421358	6.932351	-0.316524
C	-1.802295	6.144486	0.338166	H	-2.912749	7.510586	1.600588

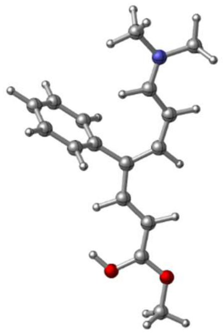

Zero-point correction= 0.329804 (Hartree/Particle)
Thermal correction to Energy= 0.349659
Thermal correction to Enthalpy= 0.350603
Thermal correction to Gibbs Free Energy= 0.279311
Sum of electronic and zero-point Energies= -825.849818
Sum of electronic and thermal Energies= -825.829962
Sum of electronic and thermal Enthalpies= -825.829018
Sum of electronic and thermal Free Energies= -825.900311

3a-OH-p1'

E(scf) = -826.155911126 a.u.

ν_{min} = 43.13 cm^{-1}

C	-1.428226	4.500650	0.968406	H	-2.770742	5.492232	-0.349635
C	-1.170423	3.370435	1.878433	C	-5.728290	-0.270340	0.033311
C	-1.969548	2.278483	1.802739	H	-5.378387	-1.152151	0.583864
H	-1.812461	1.433524	2.479697	H	-6.556815	0.217737	0.563594
C	-2.997270	2.180393	0.712981	H	-6.007107	-0.522122	-0.993459
H	-2.510766	1.932863	-0.247309	N	-4.732008	4.091713	1.244354
C	-2.570684	4.613262	0.268041	C	-4.419968	4.342172	2.638296
C	-4.026781	1.147389	0.922633	H	-4.355500	3.414414	3.247406
O	-4.395442	0.715819	2.082665	H	-3.462681	4.870097	2.736254
O	-4.630020	0.683120	-0.099923	H	-5.205070	4.971065	3.085197
C	-3.699331	3.611982	0.378489	C	-6.079606	3.604522	1.008597
H	-4.146347	3.470112	-0.615193	H	-6.283163	2.614994	1.472396

H	-6.811607	4.306795	1.437473	H	0.496368	1.379820	2.741873
H	-6.276626	3.525202	-0.070458	C	1.259839	4.798962	4.422107
H	-0.667621	5.280824	0.891276	H	-0.334574	5.596016	3.213092
C	-0.064866	3.470536	2.863137	C	1.997589	3.665391	4.779364
C	0.690985	2.338911	3.227256	H	2.291228	1.547716	4.438821
C	0.242174	4.704400	3.469667	H	1.475613	5.763200	4.888811
C	1.710366	2.435442	4.176558	H	2.797723	3.741116	5.519692

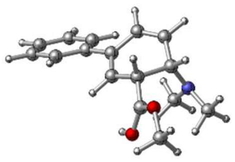

Zero-point correction= 0.331189 (Hartree/Particle)
Thermal correction to Energy= 0.349514
Thermal correction to Enthalpy= 0.350458
Thermal correction to Gibbs Free Energy= 0.284743
Sum of electronic and zero-point Energies= -825.824722
Sum of electronic and thermal Energies= -825.806397
Sum of electronic and thermal Enthalpies= -825.805453
Sum of electronic and thermal Free Energies= -825.871168

TS-3a-H-r
E(scf) = -826.125523135 a.u.
ν_{min} = -132.02 cm^{-1}

C	-0.434502	2.333880	-0.429767	O	-3.250659	-1.753329	-1.368559
C	-1.821048	2.622315	0.015081	C	0.310504	1.403319	1.695879
C	-2.798828	1.619030	-0.160562	H	-0.640931	1.573625	2.201153
H	-0.161427	2.581064	-1.461922	H	1.522787	1.578758	-0.065358
H	-3.827967	1.823422	0.144799	C	-4.238748	-2.770528	-1.551110
C	-2.572075	0.367200	-0.692824	H	-4.684000	-3.061241	-0.587022
H	-1.584629	0.048491	-1.031590	H	-5.043150	-2.419299	-2.215743
C	0.541791	1.752701	0.382495	H	-3.720826	-3.625313	-2.002491
C	-3.673579	-0.596860	-0.821559	N	1.342709	0.854161	2.589285
O	-4.827881	-0.402902	-0.485093	C	2.461669	0.119061	1.909631

H	3.064663	-0.370560	2.683071	C	-1.071176	4.875133	0.779461
H	2.033627	-0.625269	1.228980	C	-3.659392	5.553321	1.576100
H	3.081150	0.834413	1.358598	H	-4.253825	3.614095	0.896561
C	1.865730	1.919089	3.526362	C	-1.319730	6.125422	1.340954
H	2.547281	1.449091	4.246360	H	-0.054499	4.621088	0.473657
H	2.390400	2.671361	2.926015	C	-2.614650	6.473856	1.744230
H	1.015039	2.374945	4.046085	H	-4.673767	5.816067	1.885894
C	-2.112197	3.926445	0.600178	H	-0.497771	6.834594	1.465390
C	-3.417489	4.303443	1.014925	H	-2.810348	7.454131	2.185062

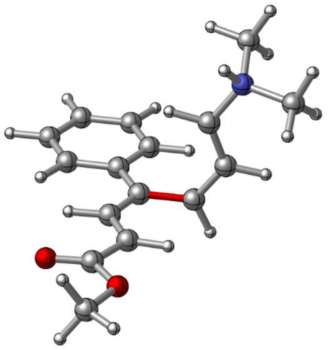

Zero-point correction= 0.328153 (Hartree/Particle)
Thermal correction to Energy= 0.347297
Thermal correction to Enthalpy= 0.348241
Thermal correction to Gibbs Free Energy= 0.278984
Sum of electronic and zero-point Energies= -825.797370
Sum of electronic and thermal Energies= -825.778227
Sum of electronic and thermal Enthalpies= -825.777282
Sum of electronic and thermal Free Energies= -825.846539

TS-3a-uu

E(scf) = -826.141145497 a.u.

ν_{min} = -461.34 cm^{-1}

C	-1.475648	4.685812	1.460235	C	-3.733904	4.105369	0.541553
C	-1.053997	3.468102	2.053679	H	-3.623007	3.629552	-0.425361
C	-1.938927	2.379327	2.131220	H	-3.093857	6.025537	1.254038
H	-1.950017	1.787563	3.052562	C	-5.681033	-0.242253	0.578587
C	-2.904344	2.102290	1.159788	H	-5.331785	-1.132904	1.120843
H	-2.596550	2.112460	0.118067	H	-6.490983	0.230533	1.154140
C	-2.771869	4.994599	1.045469	H	-6.030843	-0.516069	-0.423161
C	-4.005231	1.156088	1.489633	N	-5.165238	4.489258	0.773012
O	-4.362270	0.878574	2.615246	C	-5.559350	4.290918	2.211412
O	-4.597113	0.680350	0.382787	H	-5.484443	3.223938	2.450983

H	-4.868503	4.859647	2.843873	C	0.708234	4.562024	3.526205
H	-6.586115	4.650326	2.347477	C	2.079858	2.133738	3.713201
C	-6.089182	3.775512	-0.166988	H	0.532713	1.333035	2.440489
H	-5.937323	2.697448	-0.044846	C	1.886047	4.479078	4.270845
H	-7.122749	4.050413	0.071615	H	0.168905	5.510731	3.492668
H	-5.844677	4.073126	-1.193362	C	2.577408	3.265790	4.366534
H	-0.815413	5.543925	1.609022	H	2.614689	1.183053	3.777662
C	0.196760	3.431712	2.857317	H	2.260593	5.365260	4.788973
C	0.902232	2.216218	2.966964	H	3.499404	3.203679	4.949693

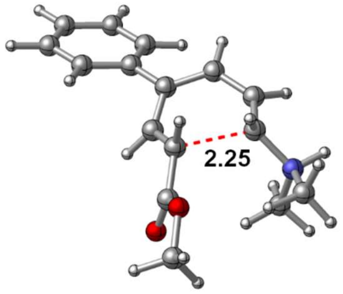

Zero-point correction= 0.331492 (Hartree/Particle)
Thermal correction to Energy= 0.349737
Thermal correction to Enthalpy= 0.350681
Thermal correction to Gibbs Free Energy= 0.284361
Sum of electronic and zero-point Energies= -825.809654
Sum of electronic and thermal Energies= -825.791409
Sum of electronic and thermal Enthalpies= -825.790464
Sum of electronic and thermal Free Energies= -825.856784

TS-3a-OH-r'
E(scf) =-826.160268552 a.u.
ν_{min} = -51.81 cm^{-1}

C	-0.347471	3.008542	-0.402235	H	-0.286280	3.114006	2.204415
C	-1.724816	3.196185	0.125307	H	1.748686	2.803897	-0.160688
C	-2.500261	2.081484	0.298695	C	-3.314776	-2.716775	0.124653
H	-0.237521	2.907535	-1.490452	H	-3.710803	-2.815871	1.146397
H	-3.512549	2.249816	0.680925	H	-4.153965	-2.643502	-0.583497
C	-2.109069	0.726830	0.056135	H	-2.693258	-3.585918	-0.120900
H	-1.097380	0.494356	-0.278534	N	1.704127	3.047002	2.613996
C	0.788552	2.938155	0.337953	C	3.121097	2.965782	2.245784
C	-2.928876	-0.360272	0.230698	H	3.544050	2.042293	2.667382
O	-4.209502	-0.345227	0.612390	H	3.256548	2.970089	1.161470
O	-2.449206	-1.580747	0.015997	H	3.645690	3.827856	2.681586
C	0.714909	3.039058	1.767624	C	1.456436	3.137032	4.058321

H	1.888808	2.254398	4.551658	H	-4.313412	4.096056	0.452630
H	1.945848	4.039946	4.451424	C	-1.713704	6.961159	0.721439
H	0.379617	3.182378	4.255911	H	-0.211685	5.483922	0.323706
C	-2.190504	4.579987	0.380231	C	-3.071121	7.233862	0.904133
C	-3.562959	4.882910	0.546009	H	-5.060479	6.377639	0.935673
C	-1.279631	5.657747	0.461583	H	-0.978808	7.768258	0.779847
C	-3.992268	6.181809	0.811017	H	-3.411463	8.252056	1.106655

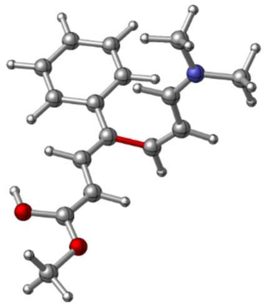

Zero-point correction= 0.328820 (Hartree/Particle)
Thermal correction to Energy= 0.348145
Thermal correction to Enthalpy= 0.349089
Thermal correction to Gibbs Free Energy= 0.279007
Sum of electronic and zero-point Energies= -825.831449
Sum of electronic and thermal Energies= -825.812123
Sum of electronic and thermal Enthalpies= -825.811179
Sum of electronic and thermal Free Energies= -825.881261

3a-OH-uu’
E(scf) = -826.149706076 a.u.
ν_{min} = -307.35 cm^{-1}

C	-1.410479	4.391634	0.930068	H	-2.750047	5.513512	-0.268308
C	-1.131636	3.269447	1.840314	C	-5.733195	-0.222165	-0.101876
C	-1.896159	2.147435	1.769402	H	-5.464166	-1.159402	0.402954
H	-1.759256	1.360121	2.519653	H	-6.516898	0.299789	0.465379
C	-2.873745	1.954270	0.688554	H	-6.062293	-0.413579	-1.128000
H	-2.482058	1.992298	-0.334129	N	-4.752900	4.067847	1.341893
C	-2.593691	4.605233	0.323901	C	-4.435762	4.619765	2.653592
C	-3.922527	1.022625	0.829705	H	-4.479057	3.832294	3.426267
O	-4.379963	0.577554	1.978186	H	-3.433614	5.061558	2.653596
O	-4.568301	0.626326	-0.228130	H	-5.169617	5.397876	2.912693
C	-3.807749	3.740262	0.435456	C	-6.100542	3.527729	1.206745
H	-4.233226	3.441481	-0.527685	H	-6.245364	2.656703	1.869383

H	-6.839080	4.293838	1.487106	H	0.552248	1.341322	2.801637
H	-6.286268	3.224056	0.168204	C	1.215055	4.822256	4.393863
H	-0.620041	5.132773	0.787070	H	-0.365658	5.553442	3.128074
C	-0.052128	3.423053	2.846335	C	1.963268	3.713694	4.804225
C	0.716579	2.317714	3.263118	H	2.299352	1.591921	4.536638
C	0.220578	4.679886	3.423142	H	1.403538	5.804419	4.834460
C	1.710193	2.461113	4.233664	H	2.744473	3.826346	5.559910

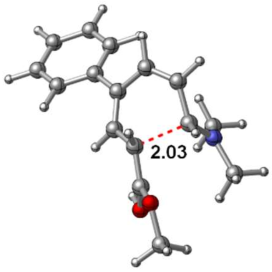

Zero-point correction= 0.329782 (Hartree/Particle)

Thermal correction to Energy= 0.348140

Thermal correction to Enthalpy= 0.349084

Thermal correction to Gibbs Free Energy= 0.283185
Sum of electronic and zero-point Energies= -825.819924

Sum of electronic and thermal Energies= -825.801566

Sum of electronic and thermal Enthalpies= -825.800622

Sum of electronic and thermal Free Energies= -825.866521

3b-H
E(scf) = -826.180724363 a.u.
ν_{min} = 25.09 cm^{-1}

C	0.332391	2.414253	-0.693750	C	-1.953744	1.775272	0.088560
C	-1.024131	2.357082	-0.849714	H	-1.561597	1.300354	0.992724

C	-3.293738	1.777918	-0.091249	H	3.222451	4.502675	-4.800937
H	-3.759731	2.237459	-0.966659	C	3.855459	5.137328	-2.275290
C	1.120692	3.014896	-1.769242	H	4.526443	5.532677	-3.047337
C	-4.190919	1.152805	0.905834	H	3.049780	5.848175	-2.058170
O	-3.834174	0.605482	1.930011	H	4.418260	4.911599	-1.362189
O	-5.477618	1.265791	0.535794	H	-1.456083	2.773579	-1.766262
C	2.446320	3.211271	-1.719133	C	1.021804	1.920399	0.534011
H	3.068115	2.935532	-0.866986	C	0.771571	2.520792	1.780377
H	0.550208	3.324210	-2.647588	C	1.933015	0.850786	0.465166
C	-6.449521	0.699219	1.420302	C	1.414304	2.059456	2.932654
H	-6.282799	-0.382154	1.540687	H	0.070705	3.356634	1.841729
H	-6.397910	1.175074	2.411576	C	2.571439	0.388234	1.618814
H	-7.427704	0.883966	0.960698	H	2.131716	0.372066	-0.496851
N	3.228493	3.863692	-2.787437	C	2.314745	0.992102	2.854939
C	2.505437	4.094861	-4.079681	H	1.210280	2.536707	3.894222
H	2.106381	3.141101	-4.442062	H	3.269088	-0.450223	1.552703
H	1.697369	4.815494	-3.912164	H	2.815591	0.630422	3.756242

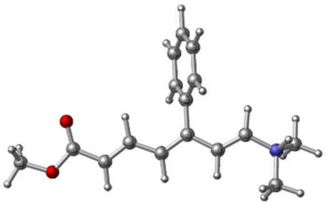

Zero-point correction= 0.332100 (Hartree/Particle)

Thermal correction to Energy= 0.351548

Thermal correction to Enthalpy= 0.352492

Thermal correction to Gibbs Free Energy= 0.281583

Sum of electronic and zero-point Energies= -825.848624

Sum of electronic and thermal Energies= -825.829177

Sum of electronic and thermal Enthalpies= -825.828232

Sum of electronic and thermal Free Energies= -825.899141

3b-H-aii
E(scf) = -826.172613092 a.u.
ν_{min} = 25.06 cm^{-1}

C	-0.828043	4.762772	1.789883	C	-0.228275	3.544576	1.669800

C	-0.810890	2.222600	1.869980	H	-5.281110	4.774830	-0.481637
H	-0.395412	1.414054	1.256814	H	-6.452164	5.315794	0.774038
C	-1.775681	1.887138	2.751162	C	-5.223559	3.104404	1.702011
H	-2.208397	2.614072	3.440203	H	-6.273336	3.205094	2.002291
C	-2.284545	4.900849	2.039713	H	-5.152960	2.574917	0.744264
C	-2.336810	0.521543	2.793905	H	-4.650961	2.569859	2.469510
O	-1.912756	-0.448900	2.202613	H	0.821240	3.536174	1.359809
O	-3.441407	0.492036	3.569733	C	-0.070726	6.032828	1.666950
C	-3.208730	4.412710	1.214946	C	1.294850	6.112801	2.011999
H	-2.990856	3.875785	0.289960	C	-0.712179	7.200841	1.206451
H	-2.586752	5.455822	2.937389	C	1.995218	7.311757	1.879475
C	-4.109535	-0.769679	3.683365	H	1.807156	5.235342	2.411916
H	-4.447113	-1.121293	2.696545	C	-0.009018	8.400440	1.074056
H	-3.437475	-1.525965	4.116094	H	-1.768208	7.166736	0.929226
H	-4.969286	-0.603190	4.343033	C	1.347608	8.460397	1.407800
N	-4.643497	4.484195	1.521273	H	3.051063	7.353754	2.157739
C	-5.394301	5.276968	0.486822	H	-0.523573	9.291626	0.706587
H	-4.966200	6.285082	0.445970	H	1.897486	9.399406	1.308788

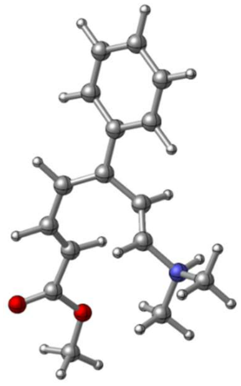

Zero-point correction= 0.331139 (Hartree/Particle)
Thermal correction to Energy= 0.350753
Thermal correction to Enthalpy= 0.351697
Thermal correction to Gibbs Free Energy= 0.280886
Sum of electronic and zero-point Energies= -825.841474
Sum of electronic and thermal Energies= -825.821860
Sum of electronic and thermal Enthalpies= -825.820916
Sum of electronic and thermal Free Energies= -825.891728

3b-H-int

E(scf) = -826.171415585 a.u.

ν_{min} = 27.03 cm^{-1}

C	-2.279705	4.324355	1.223813	H	-5.098865	5.179150	-2.181811
C	-2.423652	2.983933	1.018054	H	-6.563217	4.588018	-1.317077
C	-1.708860	1.873475	1.633082	H	-6.547224	6.242074	-2.026630
H	-1.730018	0.929300	1.077227	C	-6.494214	6.481055	0.667532
C	-1.091226	1.848992	2.833321	H	-7.099440	7.228330	0.140197
H	-1.041882	2.719092	3.488892	H	-7.111482	5.616795	0.941123
C	-3.219592	5.272990	0.601853	H	-6.032435	6.917700	1.560330
C	-0.451495	0.605915	3.321594	H	-3.173217	2.675970	0.281944
O	-0.405020	-0.450762	2.724511	C	-1.184213	4.950652	2.017365
O	0.087976	0.792186	4.538540	C	0.163862	4.666829	1.732345
C	-4.522997	5.035112	0.414741	C	-1.484511	5.869515	3.037915
H	-5.047554	4.132146	0.730923	C	1.184966	5.271724	2.465862
H	-2.806460	6.247519	0.313717	H	0.405244	3.963819	0.932248
C	0.742739	-0.335753	5.127182	C	-0.460367	6.468426	3.778444
H	0.039341	-1.173327	5.251402	H	-2.528039	6.103791	3.264348
H	1.580878	-0.671539	4.497492	C	0.875953	6.169795	3.495427
H	1.111892	-0.001460	6.104026	H	2.227925	5.044063	2.232249
N	-5.398710	6.009565	-0.250507	H	-0.708402	7.170900	4.577874
C	-5.943329	5.463884	-1.543991	H	1.676877	6.639952	4.071217

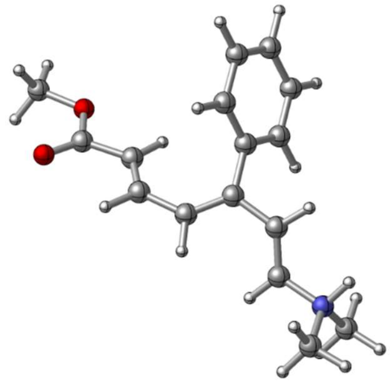

Zero-point correction= 0.331591 (Hartree/Particle)
Thermal correction to Energy= 0.351162
Thermal correction to Enthalpy= 0.352106
Thermal correction to Gibbs Free Energy= 0.281454

Sum of electronic and zero-point Energies= -825.839825
Sum of electronic and thermal Energies= -825.820253
Sum of electronic and thermal Enthalpies= -825.819309
Sum of electronic and thermal Free Energies= -825.889962

3b-H-p1

E(scf) = -826.201947898 a.u.

ν_{min} = 38.99 cm^{-1}

C	-1.548042	4.666207	1.249676	H	-4.812717	3.338393	2.973126
C	-1.474174	3.568962	2.231554	H	-3.879811	4.870573	2.818085
C	-2.253673	2.482116	2.116393	H	-5.671518	4.918474	2.934476
H	-2.203508	1.660590	2.833043	C	-6.221772	3.651138	0.652050
C	-3.158487	2.324973	0.924486	H	-6.345587	2.687795	1.155510
H	-2.531600	1.939060	0.097700	H	-7.031207	4.322572	0.962640
C	-2.578675	4.704902	0.366121	H	-6.234422	3.523573	-0.436073
C	-4.254370	1.286473	1.121287	H	-0.752482	3.640138	3.046515
O	-4.671984	0.920023	2.196000	C	-0.499488	5.718157	1.255942
O	-4.718091	0.857948	-0.052262	C	0.042067	6.191111	2.466890
C	-3.663605	3.665374	0.354208	C	-0.034793	6.276080	0.048920
H	-4.022057	3.496066	-0.668692	C	1.002935	7.205172	2.470091
H	-2.688454	5.539935	-0.329953	H	-0.306459	5.785793	3.419173
C	-5.774864	-0.117722	-0.024375	C	0.931274	7.283932	0.053668
H	-5.449070	-1.016701	0.517646	H	-0.415959	5.898287	-0.902416
H	-6.667545	0.294846	0.469061	C	1.451778	7.754433	1.264511
H	-5.993249	-0.355552	-1.071145	H	1.401995	7.568166	3.420323
N	-4.917831	4.265047	1.067412	H	1.286191	7.697559	-0.893439
C	-4.809153	4.353183	2.560591	H	2.209028	8.542176	1.267931

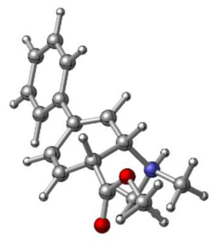

Zero-point correction= 0.335033 (Hartree/Particle)
Thermal correction to Energy= 0.352931
Thermal correction to Enthalpy= 0.353875
Thermal correction to Gibbs Free Energy= 0.288798
Sum of electronic and zero-point Energies= -825.866915

Sum of electronic and thermal Energies= -825.849017
Sum of electronic and thermal Enthalpies= -825.848073
Sum of electronic and thermal Free Energies= -825.913150

3b-OH'
E(scf) = -826.190169381 a.u.
ν_{min} = 33.25 cm^{-1}

C	0.214746	2.655577	-0.721687	H	2.020445	3.601694	-4.392702
C	-1.188385	2.523146	-0.844714	H	1.724544	5.263367	-3.794829
C	-2.008696	1.849038	0.052053	H	3.283258	4.852471	-4.564959
H	-1.515011	1.379072	0.910624	C	4.468010	4.404497	-2.400960
C	-3.398435	1.724091	-0.075574	H	5.084160	3.876741	-3.145887
H	-3.920098	2.175365	-0.921130	H	4.648585	5.486636	-2.500342
C	0.946725	3.267251	-1.756567	H	4.768795	4.083817	-1.395830
C	-4.206634	1.038899	0.828824	H	-1.656935	2.968421	-1.728348
O	-3.812479	0.425281	1.931041	C	0.909073	2.143969	0.496661
O	-5.495711	0.975817	0.597886	C	0.630457	2.692726	1.761646
C	2.313364	3.550266	-1.670508	C	1.849445	1.102503	0.397750
H	2.846693	3.310814	-0.747254	C	1.282468	2.213080	2.900615
H	0.394864	3.560926	-2.651326	H	-0.093290	3.506548	1.846298
C	-6.364610	0.285043	1.515386	C	2.492098	0.617580	1.539964
H	-6.084936	-0.775842	1.583843	H	2.065848	0.665412	-0.579793
H	-6.315187	0.746227	2.511993	C	2.212115	1.172893	2.793114
H	-7.371163	0.388134	1.096156	H	1.064033	2.655066	3.875757
N	3.055029	4.108992	-2.618512	H	3.213530	-0.198104	1.449895
C	2.486193	4.476353	-3.913562	H	2.718195	0.795827	3.685127

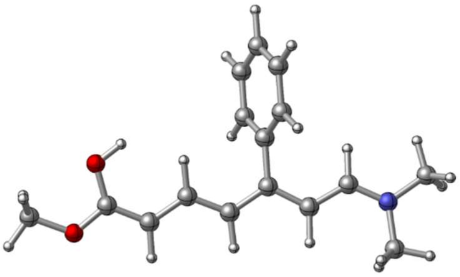

Zero-point correction= 0.329828 (Hartree/Particle)
Thermal correction to Energy= 0.349583

Thermal correction to Enthalpy= 0.350527
Thermal correction to Gibbs Free Energy= 0.279930
Sum of electronic and zero-point Energies= -825.860341
Sum of electronic and thermal Energies= -825.840586
Sum of electronic and thermal Enthalpies= -825.839642
Sum of electronic and thermal Free Energies= -825.910240

3b-OH-aii'
E(scf) = -826.177479145 a.u.
ν_{min} = 29.15 cm^{-1}

C	-0.995445	4.896648	1.327035	H	-4.684618	5.413233	3.055454
C	-0.378009	3.615300	1.139893	H	-5.176639	6.241907	1.544905
C	-0.704090	2.386430	1.701285	H	-6.270028	5.054728	2.312823
H	-0.036539	1.579253	1.371669	C	-5.543223	3.226330	0.743296
C	-1.660814	2.046053	2.681006	H	-6.117191	2.734967	1.543728
H	-2.323957	2.795533	3.111047	H	-6.248322	3.724912	0.059699
C	-2.375052	5.095031	1.503773	H	-4.978131	2.468802	0.186590
C	-1.810310	0.772137	3.205912	H	0.547526	3.624880	0.558467
O	-1.159152	-0.323906	2.843365	C	-0.109833	6.085781	1.315844
O	-2.694077	0.585260	4.164521	C	1.210819	6.001056	1.805401
C	-3.326588	4.147439	1.101919	C	-0.563102	7.327173	0.820291
H	-2.988405	3.275641	0.535830	C	2.044819	7.119724	1.807332
H	-2.715506	6.058171	1.885025	H	1.573986	5.055994	2.214295
C	-2.887832	-0.725521	4.722694	C	0.274860	8.442316	0.815383
H	-3.216067	-1.432300	3.946949	H	-1.569700	7.409650	0.405230
H	-1.959343	-1.091176	5.184288	C	1.580362	8.343229	1.311272
H	-3.667850	-0.606772	5.482793	H	3.060075	7.038378	2.202697
N	-4.628165	4.208363	1.320719	H	-0.088778	9.391753	0.415321
C	-5.220971	5.293143	2.103242	H	2.235104	9.218081	1.309284

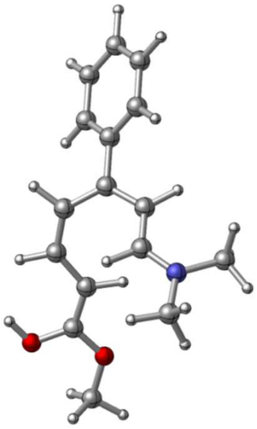

Zero-point correction= 0.329980 (Hartree/Particle)

Thermal correction to Energy= 0.349619

Thermal correction to Enthalpy= 0.350563

Thermal correction to Gibbs Free Energy= 0.280402

Sum of electronic and zero-point Energies= -825.847499

Sum of electronic and thermal Energies= -825.827860

Sum of electronic and thermal Enthalpies= -825.826916

Sum of electronic and thermal Free Energies= -825.897077

3b-OH-int'

E(scf) = -826.179928102 a.u.

ν_{min} =23.16 cm^{-1}

C	-2.231001	4.304843	1.258038	H	-0.594605	3.182484	3.575930
C	-2.190905	2.883299	1.183787	C	-3.261578	5.077825	0.687847
C	-1.486307	1.984971	1.982679	C	-0.080373	1.169741	3.833185
H	-1.617820	0.942197	1.664807	O	-0.039670	-0.110980	3.502955
C	-0.714971	2.193702	3.138848	O	0.560010	1.467226	4.941002

C	-4.462630	4.549008	0.198376	H	-6.848024	4.740827	-1.864967
H	-4.663491	3.480190	0.307943	H	-6.650054	3.528421	-0.561455
H	-3.112668	6.157510	0.660611	H	-2.826506	2.415251	0.429207
C	1.248651	0.441128	5.678097	C	-1.125470	5.064888	1.901511
H	0.541174	-0.326569	6.022605	C	0.212211	4.813019	1.541442
H	2.025400	-0.025356	5.055472	C	-1.398488	6.068770	2.848534
H	1.702308	0.952092	6.534244	C	1.249908	5.544259	2.118787
N	-5.432128	5.238162	-0.384799	H	0.429627	4.039018	0.802474
C	-5.313853	6.677400	-0.615364	C	-0.355738	6.786715	3.440461
H	-5.290183	7.222972	0.341379	H	-2.432744	6.267966	3.138526
H	-4.394395	6.902005	-1.176097	C	0.969626	6.528003	3.075736
H	-6.176591	7.020924	-1.197241	H	2.282972	5.346158	1.823115
C	-6.684224	4.601775	-0.785119	H	-0.579850	7.551007	4.188438
H	-7.527965	5.054961	-0.241064	H	1.784324	7.093640	3.534401

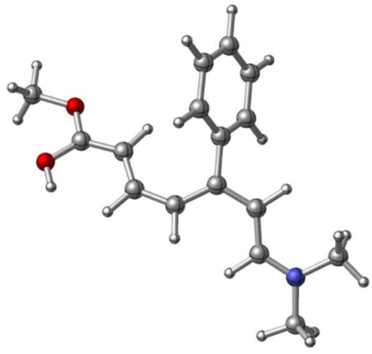

Zero-point correction= 0.329591 (Hartree/Particle)
Thermal correction to Energy= 0.349466
Thermal correction to Enthalpy= 0.350410
Thermal correction to Gibbs Free Energy= 0.278964
Sum of electronic and zero-point Energies= -825.850337
Sum of electronic and thermal Energies= -825.830462
Sum of electronic and thermal Enthalpies= -825.829518
Sum of electronic and thermal Free Energies= -825.900964

3b-OH-p1'

E(scf) = -826.154802508a.u.

ν_{min} =32.34 cm^{-1}

C	-1.675113	4.305861	0.236390	H	-1.312150	0.930306	-0.444398
C	-1.084074	3.019681	-0.171756	C	-3.161018	1.871074	0.457299
C	-1.779910	1.869941	-0.135268	H	-3.092169	1.892343	1.559134

C	-3.016600	4.388530	0.406049	C	-5.914798	2.813795	-1.315864
C	-3.986661	0.696111	0.127010	H	-5.900658	1.730039	-1.562719
O	-3.846874	-0.012640	-0.943558	H	-6.513858	2.950211	-0.403713
O	-4.931998	0.382248	0.922102	H	-6.438350	3.315442	-2.144973
C	-3.969225	3.245461	0.134652	H	-0.054035	3.015372	-0.534420
H	-4.769677	3.270520	0.886700	C	-0.780471	5.481220	0.408604
H	-3.494114	5.335921	0.665490	C	-1.198660	6.775026	0.042383
C	-5.846350	-0.715706	0.617825	C	0.509589	5.323853	0.950393
H	-6.391093	-0.477925	-0.305442	C	-0.359453	7.876172	0.224491
H	-5.271000	-1.642655	0.503125	H	-2.182184	6.916592	-0.411387
H	-6.520850	-0.765185	1.476807	C	1.348031	6.426404	1.135281
N	-4.593287	3.390710	-1.145953	H	0.856367	4.332182	1.251199
C	-3.767505	3.365227	-2.336865	C	0.916833	7.706800	0.773423
H	-3.447791	2.340676	-2.628411	H	-0.700068	8.870921	-0.073567
H	-4.329401	3.783204	-3.186149	H	2.342024	6.283835	1.566510
H	-2.861379	3.968618	-2.196395	H	1.574219	8.568475	0.912967

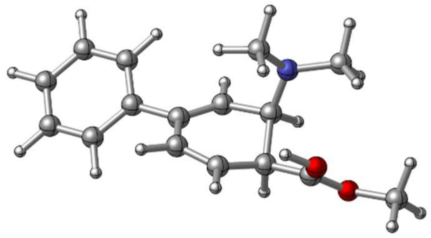

Zero-point correction= 0.331013 (Hartree/Particle)
Thermal correction to Energy= 0.349411
Thermal correction to Enthalpy= 0.350355
Thermal correction to Gibbs Free Energy= 0.284292
Sum of electronic and zero-point Energies= -825.823790
Sum of electronic and thermal Energies= -825.805392
Sum of electronic and thermal Enthalpies= -825.804447
Sum of electronic and thermal Free Energies= -825.870511

TS-3b-H-r

E(scf) = -826.128660070 a.u.

ν_{min} = -129.7 cm^{-1}

C	-1.549431	4.874941	1.103530	C	-1.223326	3.591111	0.433038

C	-1.220695	2.359343	1.069616	H	-5.932033	4.317911	-1.246367
H	-0.978402	1.469015	0.480852	H	-6.910215	5.695606	-0.625387
C	-1.510221	2.164218	2.419655	C	-6.187470	4.175685	1.482948
H	-1.758505	3.001762	3.075296	H	-7.193547	4.609737	1.534896
C	-2.906212	5.296204	1.102523	H	-6.224421	3.186671	1.010851
C	-1.490240	0.819209	3.007516	H	-5.743993	4.103942	2.482543
O	-1.235006	-0.208331	2.405926	H	-0.991403	3.627252	-0.638448
O	-1.795551	0.851900	4.318591	C	-0.486612	5.651786	1.721137
C	-3.937692	4.574037	0.576020	C	0.846939	5.161124	1.707404
H	-3.847549	3.597747	0.099034	C	-0.714571	6.903277	2.356086
H	-3.148296	6.256981	1.565725	C	1.887066	5.877065	2.293641
C	-1.803933	-0.404413	5.001921	H	1.059456	4.202015	1.232187
H	-2.541687	-1.087977	4.553978	C	0.329183	7.613322	2.939986
H	-0.812381	-0.880613	4.955517	H	-1.716840	7.331325	2.397311
H	-2.071408	-0.186612	6.042822	C	1.637214	7.107516	2.914496
N	-5.316550	5.070924	0.640757	H	2.901806	5.472863	2.267722
C	-5.898393	5.285519	-0.731274	H	0.123867	8.572302	3.421726
H	-5.252879	5.984097	-1.275434	H	2.453257	7.668931	3.375272

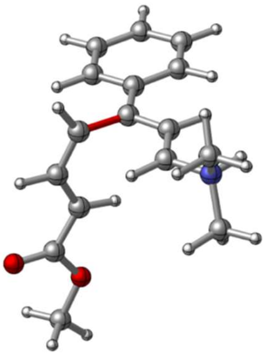

Zero-point correction= 0.327867 (Hartree/Particle)
Thermal correction to Energy= 0.347094
Thermal correction to Enthalpy= 0.348038
Thermal correction to Gibbs Free Energy= 0.278394
Sum of electronic and zero-point Energies= -825.800793
Sum of electronic and thermal Energies= -825.781566
Sum of electronic and thermal Enthalpies= -825.780622
Sum of electronic and thermal Free Energies= -825.850266

TS-3b-H-uu

E(scf) = -826.140727266 a.u.

ν_{min} = -464.38 cm^{-1}

C	-1.353401	4.564919	1.216503	C	-1.040355	3.291752	1.756961

C	-1.943536	2.248399	1.914730	H	-4.716652	4.940939	2.713834
H	-1.861837	1.631196	2.817001	H	-6.454168	4.777667	2.275141
C	-3.019076	2.001600	1.045274	C	-6.068609	3.773385	-0.209413
H	-2.807227	1.940590	-0.017788	H	-5.959078	2.696960	-0.038446
C	-2.679872	4.911489	0.882976	H	-7.082025	4.105109	0.043294
C	-4.124844	1.124751	1.515251	H	-5.842069	4.012023	-1.255348
O	-4.387671	0.910368	2.680380	H	-0.101473	3.226835	2.312665
O	-4.839454	0.632379	0.489586	C	-0.366005	5.675056	1.307745
C	-3.679381	4.059572	0.403441	C	0.600903	5.755842	2.329982
H	-3.607284	3.587530	-0.569198	C	-0.397057	6.696380	0.336858
H	-2.977803	5.928005	1.174238	C	1.504145	6.818819	2.374937
C	-5.940719	-0.226048	0.826572	H	0.633795	5.000613	3.117411
H	-5.581998	-1.112436	1.369755	C	0.508377	7.759190	0.381918
H	-6.667946	0.307721	1.456582	H	-1.123698	6.640388	-0.477268
H	-6.398985	-0.519466	-0.124658	C	1.463135	7.823859	1.401228
N	-5.090750	4.493171	0.670013	H	2.239215	6.867928	3.181904
C	-5.446280	4.372862	2.126156	H	0.471940	8.534678	-0.387023
H	-5.404860	3.315544	2.410808	H	2.173014	8.653711	1.438391

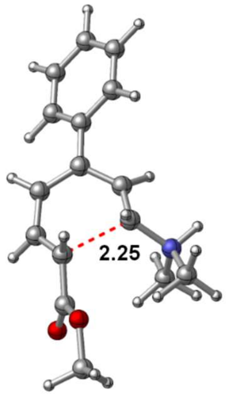

Zero-point correction= 0.331649 (Hartree/Particle)

Thermal correction to Energy= 0.349785

Thermal correction to Enthalpy= 0.350730

Thermal correction to Gibbs Free Energy= 0.284833

Sum of electronic and zero-point Energies= -825.809078

Sum of electronic and thermal Energies= -825.790942

Sum of electronic and thermal Enthalpies= -825.789998

Sum of electronic and thermal Free Energies= -825.855894

TS-3b-OH-dd’
E(scf) = -826.148431900 a.u.
ν_{min} = -318.87 cm^{-1}

C	-1.699789	4.302640	0.139861	H	-3.569880	2.688138	-2.996599
C	-1.091762	3.030920	-0.283235	H	-4.520623	4.188121	-3.188866
C	-1.721128	1.843961	-0.172074	H	-2.988131	4.216815	-2.269526
H	-1.241709	0.945121	-0.577580	C	-5.885780	2.629528	-1.472174
C	-3.013648	1.734280	0.527101	H	-5.718495	1.619665	-1.885053
H	-3.024769	2.060323	1.572894	H	-6.424133	2.543579	-0.519245
C	-3.046778	4.425905	0.244539	H	-6.514664	3.186728	-2.182902
C	-3.872497	0.644535	0.289048	H	-0.098786	3.074541	-0.736382
O	-3.844009	-0.112661	-0.785347	C	-0.798194	5.460155	0.388088
O	-4.838894	0.399626	1.126169	C	-1.176965	6.773992	0.051749
C	-4.067117	3.375099	-0.046140	C	0.465428	5.256840	0.975006
H	-4.806163	3.242002	0.750120	C	-0.325678	7.850547	0.308575
H	-3.491307	5.387725	0.519597	H	-2.136719	6.952937	-0.438230
C	-5.811016	-0.632992	0.841989	C	1.315084	6.334898	1.235914
H	-6.361025	-0.387536	-0.077556	H	0.779911	4.246853	1.248919
H	-5.309307	-1.603526	0.732178	C	0.922582	7.635737	0.904164
H	-6.484936	-0.639175	1.704376	H	-0.634326	8.861917	0.032691
N	-4.622396	3.330175	-1.276274	H	2.287533	6.157135	1.701657
C	-3.879693	3.622203	-2.495912	H	1.589170	8.478597	1.102487

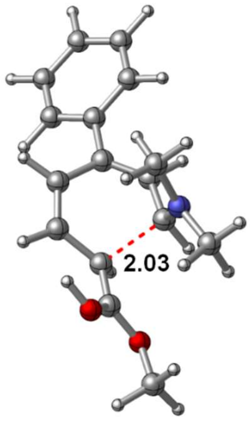

Zero-point correction= 0.329477 (Hartree/Particle)

Thermal correction to Energy= 0.347983

Thermal correction to Enthalpy= 0.348927

Thermal correction to Gibbs Free Energy= 0.282336

Sum of electronic and zero-point Energies= -825.818955

Sum of electronic and thermal Energies= -825.800449

Sum of electronic and thermal Enthalpies= -825.799505

Sum of electronic and thermal Free Energies= -825.866096

TS-3b-OH-r'

E(scf) = -826.166183776 a.u.

ν_{min} = -48.47 cm^{-1}

C	-1.487231	4.778150	1.005915	H	-5.438345	6.019048	1.956327
C	-1.075725	3.433971	0.512495	H	-5.224478	6.582941	0.266508
C	-0.974126	2.337195	1.302535	H	-6.744654	5.784489	0.760026
H	-0.650427	1.417800	0.798498	C	-6.073571	3.462968	0.119935
C	-1.261350	2.273514	2.709762	H	-6.789717	3.283259	0.935849
H	-1.620222	3.162553	3.230740	H	-6.624926	3.812860	-0.765682
C	-2.819243	5.141745	0.989869	H	-5.550812	2.529389	-0.119114
C	-1.126461	1.151760	3.481685	H	-0.833209	3.362867	-0.552358
O	-0.685717	-0.048031	3.084143	C	-0.438547	5.693337	1.487686
O	-1.457378	1.196385	4.770407	C	0.916136	5.292528	1.431134
C	-3.826133	4.231475	0.579425	C	-0.729606	6.973933	2.018203
H	-3.521421	3.224170	0.282510	C	1.934678	6.132783	1.878887
H	-3.118295	6.138153	1.309960	H	1.162357	4.308418	1.031120
C	-1.257002	0.045437	5.597101	C	0.288203	7.810003	2.465963
H	-1.860293	-0.805449	5.246308	H	-1.759117	7.324916	2.093707
H	-0.196576	-0.248535	5.618383	C	1.625523	7.394179	2.397278
H	-1.581426	0.342677	6.601501	H	2.974038	5.801457	1.823846
N	-5.111212	4.486161	0.533226	H	0.040889	8.792398	2.874426
C	-5.658749	5.797886	0.901797	H	2.422190	8.053593	2.749986

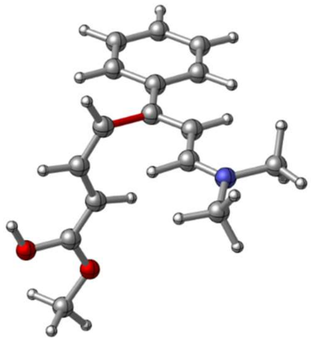

Zero-point correction= 0.328793 (Hartree/Particle)
Thermal correction to Energy= 0.348045
Thermal correction to Enthalpy= 0.348989
Thermal correction to Gibbs Free Energy= 0.279018
Sum of electronic and zero-point Energies= -825.837391
Sum of electronic and thermal Energies= -825.818139
Sum of electronic and thermal Enthalpies= -825.817194
Sum of electronic and thermal Free Energies= -825.887166

TS-3a-dd
E(scf) = -825.722679153 a.u.
ν_{min} = -271.06 cm^{-1}

C	-1.547054	4.106997	0.269649
C	-0.823768	2.863735	0.199963
C	-1.527881	1.668159	0.107577
H	-0.931139	5.009511	0.331151
H	-1.080183	0.814194	-0.414938
C	-2.850816	1.548967	0.581097
H	-3.075396	1.991090	1.550823
C	-2.880299	4.344852	0.022586
C	-3.693194	0.431215	0.170332
O	-3.460844	-0.360195	-0.728004
O	-4.852664	0.392207	0.883719
C	-3.988156	3.407018	-0.079620
H	-4.508882	3.093769	0.821668
H	-3.135298	5.365426	-0.292144
C	-5.792044	-0.612710	0.510099
H	-6.127548	-0.472052	-0.530238
H	-5.354365	-1.619034	0.598963
H	-6.644026	-0.510704	1.194103
N	-4.773633	3.394766	-1.173988
C	-4.291287	3.794043	-2.486307
H	-4.465525	2.982333	-3.213256
H	-4.817342	4.694787	-2.845951
H	-3.215981	4.008468	-2.442312
C	-6.099912	2.798925	-1.142756
H	-6.852846	3.511498	-1.519268
H	-6.134182	1.897497	-1.779443

H	-6.360119	2.511018	-0.116774	C	2.700809	3.927304	-0.825118
C	0.646584	2.880512	-0.001007	H	0.746895	4.801668	-1.011317
C	1.431007	1.788002	0.430165	C	3.458695	2.831002	-0.397132
C	1.317247	3.949686	-0.635024	H	3.391518	0.898786	0.576597
C	2.813113	1.759016	0.228637	H	3.189475	4.768541	-1.324304
H	0.946812	0.959418	0.952481	H	4.540994	2.813845	-0.548329

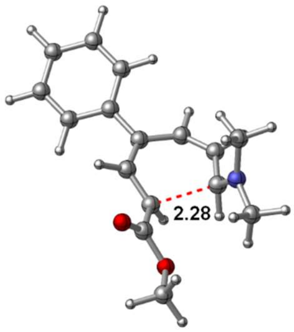

Zero-point correction= 0.315739 (Hartree/Particle)

Thermal correction to Energy= 0.334345

Thermal correction to Enthalpy= 0.335290

Thermal correction to Gibbs Free Energy= 0.267966

Sum of electronic and zero-point Energies= -825.406940

Sum of electronic and thermal Energies= -825.388334

Sum of electronic and thermal Enthalpies= -825.387389

Sum of electronic and thermal Free Energies= -825.454713

TS-3a-du
E(scf) = -825.712056002 a.u.
ν_{min} = -560.69 cm^{-1}

C	-1.825050	4.186135	0.848739	H	-1.664171	0.982769	-0.401062
C	-1.251318	2.961139	0.268977	C	-3.336650	1.678047	0.778941
C	-2.038841	1.825142	0.191413	H	-3.558946	2.170960	1.726443
H	-1.152864	4.869300	1.379900	C	-3.132471	4.527591	0.765824

C	-4.316408	0.720803	0.379879	C	-6.253746	3.140284	-0.901967
O	-5.422861	0.559859	0.905017	H	-6.667735	2.201740	-0.507673
O	-3.966396	0.010665	-0.743264	H	-7.073152	3.834686	-1.142405
C	-4.071772	3.738376	-0.053605	H	-5.679950	2.925772	-1.811718
H	-3.722556	3.443866	-1.049290	C	0.135574	2.995644	-0.228194
H	-3.508344	5.431781	1.249486	C	0.921841	1.823424	-0.336370
C	-4.936512	-0.897438	-1.243924	C	0.739064	4.212486	-0.626010
H	-5.234683	-1.635257	-0.481839	C	2.223736	1.863951	-0.837280
H	-5.845186	-0.370380	-1.580875	H	0.513007	0.868716	0.001940
H	-4.472627	-1.410673	-2.097135	C	2.047554	4.253367	-1.112933
N	-5.381921	3.748677	0.104032	H	0.164383	5.140826	-0.577723
C	-6.006217	3.980550	1.402850	C	2.800415	3.079282	-1.227619
H	-6.981305	4.466787	1.261530	H	2.801817	0.938065	-0.906024
H	-6.150648	3.003214	1.893068	H	2.479054	5.211748	-1.415349
H	-5.371885	4.609845	2.036886	H	3.823955	3.110800	-1.609348

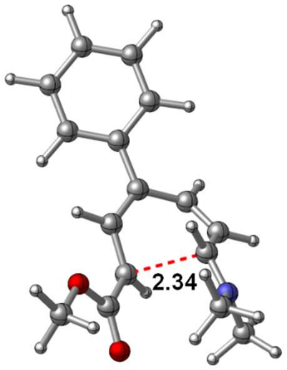

Zero-point correction= 0.315493 (Hartree/Particle)
Thermal correction to Energy= 0.334036
Thermal correction to Enthalpy= 0.334980
Thermal correction to Gibbs Free Energy= 0.268160
Sum of electronic and zero-point Energies= -825.396563
Sum of electronic and thermal Energies= -825.378020
Sum of electronic and thermal Enthalpies= -825.377076
Sum of electronic and thermal Free Energies= -825.443896

TS-3a-H-dd
E(scf) = -826.140311565 a.u.
ν_{min} = -442.01 cm^{-1}

C	-1.516028	4.084252	0.318859
C	-0.767430	2.890849	0.132568
C	-1.421780	1.714408	-0.252648
H	-0.934600	4.994690	0.484253
H	-0.921446	1.020291	-0.934918
C	-2.747952	1.458518	0.111420
H	-3.022356	1.662803	1.143611
C	-2.846245	4.291098	-0.041220
C	-3.469700	0.316372	-0.513866
O	-3.133209	-0.243257	-1.534784
O	-4.574470	0.003851	0.181177
C	-3.887717	3.344593	-0.147054
H	-4.393237	2.914357	0.712410
H	-3.061125	5.301441	-0.419815
C	-5.360364	-1.089933	-0.315527
H	-5.723451	-0.876298	-1.332334
H	-4.761833	-2.012235	-0.339799
H	-6.202991	-1.198140	0.376706
N	-4.925172	3.687030	-1.178886
C	-5.450945	2.459764	-1.862617
H	-5.909751	1.813804	-1.106395
H	-6.195533	2.765484	-2.606984
H	-4.615878	1.942130	-2.347174
C	-6.036732	4.520246	-0.603020
H	-6.726816	4.796712	-1.409735
H	-6.557814	3.922556	0.154574
H	-5.603821	5.417553	-0.146231
C	0.717014	2.949379	0.080468
C	1.462473	1.817069	0.466870
C	1.417482	4.093184	-0.352381
C	2.858328	1.828559	0.426720
H	0.939470	0.926945	0.824404
C	2.813060	4.104250	-0.390761
H	0.870769	4.975113	-0.692245
C	3.539212	2.973193	-0.000730
H	3.416249	0.942183	0.738340
H	3.336498	4.998396	-0.738033
H	4.631431	2.984171	-0.031491

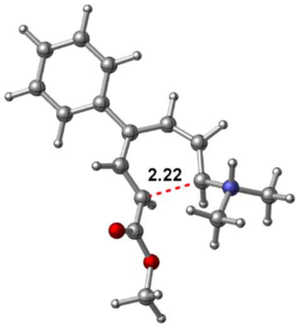

Zero-point correction= 0.331674 (Hartree/Particle)
Thermal correction to Energy= 0.349856
Thermal correction to Enthalpy= 0.350800
Thermal correction to Gibbs Free Energy= 0.284574
Sum of electronic and zero-point Energies= -825.808637
Sum of electronic and thermal Energies= -825.790456
Sum of electronic and thermal Enthalpies= -825.789511
Sum of electronic and thermal Free Energies= -825.855738

TS-3a-H-du

E(scf) = -826.134641835 a.u.

ν_{min} = -452.13 cm^{-1}

C	-2.251304	3.857057	-0.942429
C	-1.430928	2.949775	-0.226219
C	-1.904531	2.347457	0.954335
H	-1.731135	4.522908	-1.636005
H	-1.202432	2.343545	1.796821
C	-3.215026	1.922469	1.221071

H	-3.504185	1.861826	2.275294	H	-5.414545	3.291625	2.595663
C	-3.557865	4.216293	-0.620375	C	-6.785416	4.198198	-0.396705
C	-3.872888	0.822429	0.459858	H	-7.116979	3.213035	-0.746137
O	-4.821014	0.197812	0.890590	H	-7.614193	4.730739	0.085765
O	-3.330648	0.605685	-0.743427	H	-6.389916	4.783847	-1.234150
C	-4.487431	3.365352	-0.010044	C	0.029935	2.897248	-0.498107
H	-4.791928	2.462373	-0.529165	C	0.712813	1.686270	-0.267108
H	-3.834345	5.265574	-0.792054	C	0.766506	4.000497	-0.973099
C	-3.914552	-0.434187	-1.542219	C	2.084925	1.579030	-0.505861
H	-3.823445	-1.405463	-1.034513	H	0.150977	0.815352	0.079070
H	-4.978508	-0.227063	-1.730211	C	2.137747	3.892680	-1.210630
H	-3.353752	-0.441328	-2.483768	H	0.275150	4.962591	-1.131500
N	-5.685731	3.995118	0.611841	C	2.802081	2.682173	-0.979415
C	-6.166560	3.213006	1.803851	H	2.593454	0.628322	-0.328206
H	-7.119663	3.637337	2.139612	H	2.693738	4.762541	-1.568761
H	-6.285571	2.163751	1.508156	H	3.875439	2.600804	-1.167370

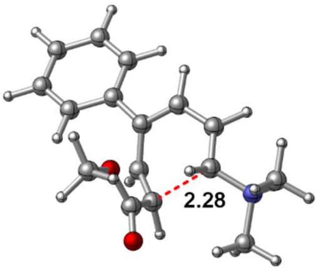

Zero-point correction= 0.331309 (Hartree/Particle)
Thermal correction to Energy= 0.349503
Thermal correction to Enthalpy= 0.350447
Thermal correction to Gibbs Free Energy= 0.284320
Sum of electronic and zero-point Energies= -825.803333
Sum of electronic and thermal Energies= -825.785139
Sum of electronic and thermal Enthalpies= -825.784195
Sum of electronic and thermal Free Energies= -825.850322

TS-3a-H-ud
E(scf) = -826.134517421 a.u.
ν_{min} = -459.25 cm^{-1}

C	-1.334476	4.583610	-0.201692	C	-2.636249	4.948586	-0.547216
C	-0.814222	3.267200	-0.185375	C	-3.547089	1.825510	1.016000
C	-1.614786	2.178600	-0.593190	O	-4.614507	1.286580	1.226347
H	-0.593958	5.387788	-0.205431	O	-2.773713	2.335140	1.981812
H	-1.135787	1.485125	-1.295285	C	-3.759211	4.164524	-0.276412
C	-2.980453	1.986640	-0.352405	H	-3.979789	3.916320	0.754934
H	-3.516018	1.333948	-1.050926	H	-2.751804	5.870644	-1.132872

C	-3.252950	2.213489	3.329541
H	-4.225004	2.716376	3.441481
H	-3.364281	1.154291	3.603508
H	-2.498239	2.694623	3.961961
N	-5.014124	4.436328	-1.036501
C	-4.863924	4.129288	-2.499243
H	-4.686363	3.052894	-2.601579
H	-5.785130	4.421726	-3.016745
H	-4.010381	4.688877	-2.897223
C	-6.181962	3.706633	-0.438563
H	-7.071240	3.911232	-1.044831
H	-5.952995	2.634756	-0.425874
H	-6.331197	4.062554	0.587364
C	0.653681	3.050027	-0.093515
C	1.118913	1.855214	0.491628
C	1.603408	3.978809	-0.563601
C	2.486901	1.597965	0.607988
H	0.394885	1.135766	0.881719
C	2.970131	3.720448	-0.447769
H	1.277456	4.899213	-1.052295
C	3.417411	2.530619	0.139306
H	2.826257	0.669208	1.073015
H	3.691269	4.448136	-0.827830
H	4.488149	2.332028	0.229500

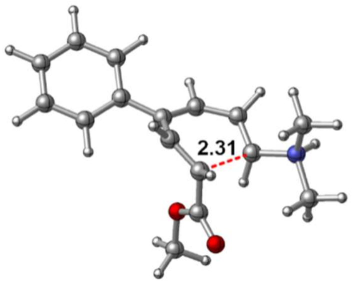

Zero-point correction= 0.331229 (Hartree/Particle)
Thermal correction to Energy= 0.349445
Thermal correction to Enthalpy= 0.350389
Thermal correction to Gibbs Free Energy= 0.284004
Sum of electronic and zero-point Energies= -825.803288
Sum of electronic and thermal Energies= -825.785072
Sum of electronic and thermal Enthalpies= -825.784128
Sum of electronic and thermal Free Energies= -825.850513

TS-3a-H-uu

E(scf) = -826.141145497 a.u.

ν_{min} = -461.34 cm^{-1}

C	-1.475648	4.685812	1.460235
C	-1.053997	3.468102	2.053679
C	-1.938927	2.379327	2.131220
H	-1.950017	1.787563	3.052562
C	-2.904344	2.102290	1.159788
H	-2.596550	2.112460	0.118067
C	-2.771869	4.994599	1.045469
C	-4.005231	1.156088	1.489633
O	-4.362270	0.878574	2.615246
O	-4.597113	0.680350	0.382787
C	-3.733904	4.105369	0.541553
H	-3.623007	3.629552	-0.425361
H	-3.093857	6.025537	1.254038
C	-5.681033	-0.242253	0.578587
H	-5.331785	-1.132904	1.120843
H	-6.490983	0.230533	1.154140
H	-6.030843	-0.516069	-0.423161
N	-5.165238	4.489258	0.773012
C	-5.559350	4.290918	2.211412
H	-5.484443	3.223938	2.450983
H	-4.868503	4.859647	2.843873
H	-6.586115	4.650326	2.347477

C	-6.089182	3.775512	-0.166988	C	2.079858	2.133738	3.713201
H	-5.937323	2.697448	-0.044846	H	0.532713	1.333035	2.440489
H	-7.122749	4.050413	0.071615	C	1.886047	4.479078	4.270845
H	-5.844677	4.073126	-1.193362	H	0.168905	5.510731	3.492668
H	-0.815413	5.543925	1.609022	C	2.577408	3.265790	4.366534
C	0.196760	3.431712	2.857317	H	2.614689	1.183053	3.777662
C	0.902232	2.216218	2.966964	H	2.260593	5.365260	4.788973
C	0.708234	4.562024	3.526205	H	3.499404	3.203679	4.949693

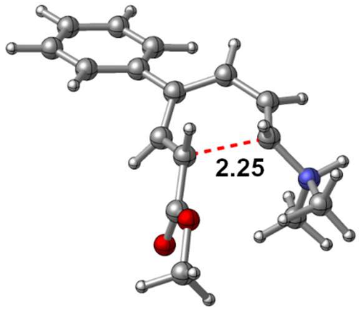

Zero-point correction= 0.331492 (Hartree/Particle)
Thermal correction to Energy= 0.349737
Thermal correction to Enthalpy= 0.350681
Thermal correction to Gibbs Free Energy= 0.284361
Sum of electronic and zero-point Energies= -825.809654
Sum of electronic and thermal Energies= -825.791409
Sum of electronic and thermal Enthalpies= -825.790464
Sum of electronic and thermal Free Energies= -825.856784

TS-3a-OH-dd'
E(scf) = -826.149667240 a.u.
ν_{min} = -310.59 cm^{-1}

C	-1.662240	4.069067	0.722936	H	-6.354432	-0.394191	-0.420138
C	-0.981974	2.830133	0.313241	H	-5.454925	-1.681965	0.458950
C	-1.690788	1.671685	0.249872	H	-6.729256	-0.730326	1.312123
H	-1.037712	4.898589	1.064028	N	-4.385559	3.341465	-1.148271
H	-1.213460	0.776162	-0.164184	C	-3.468327	3.660805	-2.235829
C	-3.069096	1.587754	0.753893	H	-3.153339	2.741143	-2.759104
H	-3.220507	1.856871	1.805213	H	-3.972292	4.315669	-2.962806
C	-2.990218	4.273132	0.633549	H	-2.579515	4.172677	-1.851977
C	-3.917945	0.542153	0.336730	C	-5.637042	2.715813	-1.558137
O	-3.761082	-0.153767	-0.766506	H	-6.134838	3.340354	-2.315206
O	-4.993530	0.282630	1.021162	H	-5.455209	1.722114	-2.003276
C	-4.001512	3.286337	0.144693	H	-6.308423	2.607365	-0.696344
H	-4.843314	3.134979	0.827730	C	0.448803	2.898521	-0.072849
H	-3.411120	5.255709	0.873198	C	1.316299	1.810822	0.153239
C	-5.942987	-0.702671	0.551269	C	0.972850	4.050363	-0.693696

C	2.653853	1.866367	-0.243591	C	3.157397	3.013311	-0.867764
H	0.945313	0.922629	0.669741	H	3.310252	1.013734	-0.052364
C	2.311494	4.105212	-1.089482	H	2.694246	5.005088	-1.577355
H	0.322999	4.906009	-0.891859	H	4.205528	3.058200	-1.173433

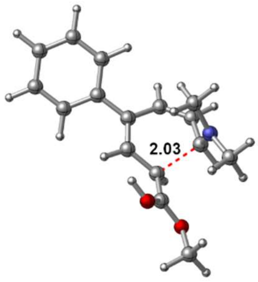

Zero-point correction= 0.329801 (Hartree/Particle)

Thermal correction to Energy= 0.348166

Thermal correction to Enthalpy= 0.349110

Thermal correction to Gibbs Free Energy= 0.283215

Sum of electronic and zero-point Energies= -825.819866

Sum of electronic and thermal Energies= -825.801501

Sum of electronic and thermal Enthalpies= -825.800557

Sum of electronic and thermal Free Energies= -825.866453

TS-3a-OH-du’

E(scf) = -826.147067192 a.u.

ν_{min} = -231.47 cm^{-1}

C	-2.636233	2.672434	-1.571319	O	-5.131036	-0.429183	0.598073
C	-1.803647	2.527064	-0.362140	O	-3.310978	-0.252713	-0.652851
C	-2.327505	2.029299	0.786192	C	-4.848419	2.734444	-0.368193
H	-2.117115	2.732684	-2.531390	H	-5.688157	2.036503	-0.445310
H	-1.728972	2.057330	1.702414	H	-4.515823	2.935267	-2.512116
C	-3.687408	1.466057	0.906532	C	-3.739929	-1.440074	-1.353192
H	-4.205857	1.651023	1.853337	H	-3.815105	-2.285907	-0.656320
C	-3.974967	2.789612	-1.570782	H	-4.710772	-1.264199	-1.836334
C	-4.025236	0.238552	0.319771	H	-2.965417	-1.627217	-2.104081

N	-5.143362	3.842061	0.324944	C	0.623093	2.367700	0.302412
C	-6.228460	3.814873	1.298989	C	-0.055173	4.121682	-1.217788
H	-5.831379	3.643808	2.315450	C	1.936357	2.840738	0.261219
H	-6.755677	4.779822	1.292565	H	0.389424	1.476639	0.889436
H	-6.940542	3.016437	1.053511	C	1.258829	4.594710	-1.258259
C	-4.203460	4.946715	0.479001	H	-0.826878	4.641726	-1.790663
H	-4.760064	5.893940	0.518510	C	2.260125	3.957266	-0.518024
H	-3.633991	4.832038	1.417882	H	2.714267	2.326709	0.831438
H	-3.501733	4.974765	-0.361274	H	1.500411	5.468049	-1.869273
C	-0.398627	3.005178	-0.429268	H	3.288879	4.323998	-0.553715

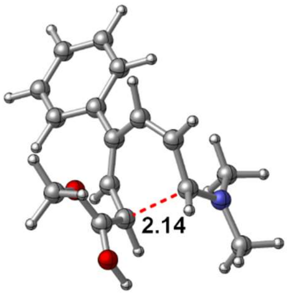

Zero-point correction= 0.329273 (Hartree/Particle)

Thermal correction to Energy= 0.347898

Thermal correction to Enthalpy= 0.348842

Thermal correction to Gibbs Free Energy= 0.282046

Sum of electronic and zero-point Energies= -825.817794

Sum of electronic and thermal Energies= -825.799169

Sum of electronic and thermal Enthalpies= -825.798225

Sum of electronic and thermal Free Energies= -825.865022

TS-3a-OH-ud’
E(scf) = -826.142578945 a.u.
ν_{min} = -228.28 cm^{-1}

C	-1.666837	4.290178	0.619375	C	-2.948304	4.680521	0.507320
C	-1.128279	3.011390	0.116103	C	-3.737781	1.851723	1.686997
C	-1.940161	1.938831	-0.062327	O	-5.002732	1.597448	1.974162
H	-0.957633	4.985501	1.076063	O	-2.880929	2.175383	2.625068
H	-1.545444	1.046594	-0.559039	C	-4.047657	3.871750	-0.084582
C	-3.358814	1.895461	0.341996	H	-4.942815	3.778049	0.538341
H	-4.050604	1.351269	-0.306890	H	-3.257949	5.666245	0.871182

C	-3.278670	2.364071	3.994336	H	-6.310818	3.307896	-1.089389
H	-4.017365	3.176395	4.075583	C	0.312142	2.962428	-0.244392
H	-3.668264	1.427113	4.422856	C	1.064176	1.782268	-0.078401
H	-2.365990	2.647984	4.528736	C	0.960716	4.094985	-0.776365
N	-4.320319	3.929630	-1.394629	C	2.409888	1.731473	-0.447876
C	-3.292338	4.145459	-2.406361	H	0.594605	0.903031	0.368341
H	-2.939545	3.176776	-2.801228	C	2.307218	4.043638	-1.145804
H	-3.716437	4.729597	-3.235721	H	0.402533	5.022732	-0.924260
H	-2.441160	4.687575	-1.981029	C	3.037497	2.861492	-0.984427
C	-5.566789	3.360672	-1.894464	H	2.975366	0.807268	-0.304235
H	-5.959716	3.987496	-2.708150	H	2.787021	4.931655	-1.564903
H	-5.394702	2.344404	-2.291484	H	4.091948	2.822690	-1.268572

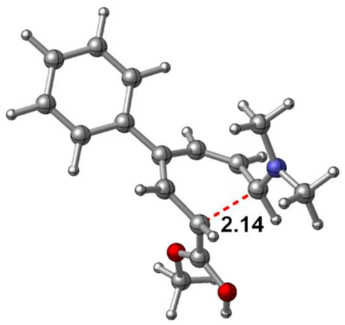

Zero-point correction= 0.328540 (Hartree/Particle)

Thermal correction to Energy= 0.347434

Thermal correction to Enthalpy= 0.348379

Thermal correction to Gibbs Free Energy= 0.280772

Sum of electronic and zero-point Energies= -825.814039

Sum of electronic and thermal Energies= -825.795145

Sum of electronic and thermal Enthalpies= -825.794200

Sum of electronic and thermal Free Energies= -825.861807

TS-3a-OH-uu’

E(scf) = -826.149706076 a.u.

ν_{min} = -307.35 cm^{-1}

C	-1.410479	4.391634	0.930068	C	-2.593691	4.605233	0.323901
C	-1.131636	3.269447	1.840314	C	-3.922527	1.022625	0.829705
C	-1.896159	2.147435	1.769402	O	-4.379963	0.577554	1.978186
H	-1.759256	1.360121	2.519653	O	-4.568301	0.626326	-0.228130
C	-2.873745	1.954270	0.688554	C	-3.807749	3.740262	0.435456
H	-2.482058	1.992298	-0.334129	H	-4.233226	3.441481	-0.527685

H	-2.750047	5.513512	-0.268308	H	-6.286268	3.224056	0.168204
C	-5.733195	-0.222165	-0.101876	H	-0.620041	5.132773	0.787070
H	-5.464166	-1.159402	0.402954	C	-0.052128	3.423053	2.846335
H	-6.516898	0.299789	0.465379	C	0.716579	2.317714	3.263118
H	-6.062293	-0.413579	-1.128000	C	0.220578	4.679886	3.423142
N	-4.752900	4.067847	1.341893	C	1.710193	2.461113	4.233664
C	-4.435762	4.619765	2.653592	H	0.552248	1.341322	2.801637
H	-4.479057	3.832294	3.426267	C	1.215055	4.822256	4.393863
H	-3.433614	5.061558	2.653596	H	-0.365658	5.553442	3.128074
H	-5.169617	5.397876	2.912693	C	1.963268	3.713694	4.804225
C	-6.100542	3.527729	1.206745	H	2.299352	1.591921	4.536638
H	-6.245364	2.656703	1.869383	H	1.403538	5.804419	4.834460
H	-6.839080	4.293838	1.487106	H	2.744473	3.826346	5.559910

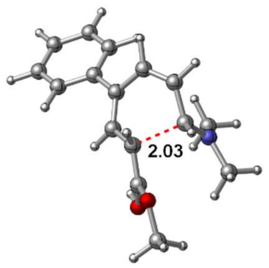

Zero-point correction= 0.329782 (Hartree/Particle)

Thermal correction to Energy= 0.348140

Thermal correction to Enthalpy= 0.349084

Thermal correction to Gibbs Free Energy= 0.283185

Sum of electronic and zero-point Energies= -825.819924

Sum of electronic and thermal Energies= -825.801566

Sum of electronic and thermal Enthalpies= -825.800622

Sum of electronic and thermal Free Energies= -825.866521

TS-3a-ud
E(scf) = -825.713904076 a.u.
ν_{min} = -318.43 cm^{-1}

C	-1.426196	4.395015	0.002069	H	-1.163486	1.152685	-0.884617
C	-0.849189	3.078609	-0.045118	C	-3.022700	1.833525	-0.032254
C	-1.636196	1.966301	-0.316833	H	-3.589038	1.131648	-0.654120
H	-0.706888	5.217415	0.060694	C	-2.715314	4.780640	-0.266952

C	-3.568043	1.840546	1.325680	C	-6.194646	3.545291	-0.987137
O	-4.646174	1.354093	1.637436	H	-6.976911	4.268877	-1.269099
O	-2.792119	2.480689	2.232462	H	-6.331609	2.635394	-1.599691
C	-3.919992	3.977615	-0.258089	H	-6.319655	3.269573	0.068007
H	-4.323273	3.721235	0.717479	C	0.630771	2.937575	-0.083206
H	-2.861194	5.818993	-0.590361	C	1.231711	1.769870	0.433551
C	-3.288433	2.536803	3.567820	C	1.482367	3.918552	-0.635429
H	-4.260647	3.052784	3.609050	C	2.616360	1.584815	0.392700
H	-3.414647	1.526680	3.988016	H	0.598375	1.009050	0.896107
H	-2.542933	3.094380	4.148889	C	2.866940	3.738950	-0.668380
N	-4.873689	4.112328	-1.201866	H	1.057111	4.824940	-1.071776
C	-4.567186	4.491921	-2.570349	C	3.443213	2.570274	-0.156141
H	-4.806866	3.661704	-3.259204	H	3.052134	0.670601	0.804730
H	-5.159114	5.370856	-2.875387	H	3.500554	4.513358	-1.109185
H	-3.501658	4.733490	-2.669428	H	4.526963	2.431230	-0.183096

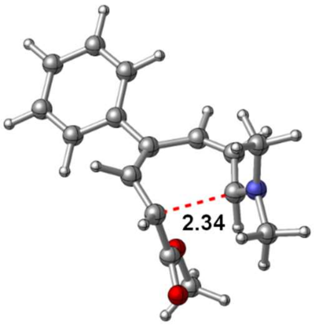

Zero-point correction= 0.315350 (Hartree/Particle)

Thermal correction to Energy= 0.334113

Thermal correction to Enthalpy= 0.335057

Thermal correction to Gibbs Free Energy= 0.266729

Sum of electronic and zero-point Energies= -825.398554

Sum of electronic and thermal Energies= -825.379791

Sum of electronic and thermal Enthalpies= -825.378847

Sum of electronic and thermal Free Energies= -825.447175

TS-3a-uu
E(scf) = -825.722678629 a.u.
ν_{min} = -268.29 cm^{-1}

C	-1.475648	4.685812	1.460235	H	-1.950017	1.787563	3.052562
C	-1.053997	3.468102	2.053679	C	-2.904344	2.102290	1.159788
C	-1.938927	2.379327	2.131220	H	-2.596550	2.112460	0.118067

C	-2.771869	4.994599	1.045469	C	-6.089182	3.775512	-0.166988
C	-4.005231	1.156088	1.489633	H	-5.937323	2.697448	-0.044846
O	-4.362270	0.878574	2.615246	H	-7.122749	4.050413	0.071615
O	-4.597113	0.680350	0.382787	H	-5.844677	4.073126	-1.193362
C	-3.733904	4.105369	0.541553	H	-0.815413	5.543925	1.609022
H	-3.623007	3.629552	-0.425361	C	0.196760	3.431712	2.857317
H	-3.093857	6.025537	1.254038	C	0.902232	2.216218	2.966964
C	-5.681033	-0.242253	0.578587	C	0.708234	4.562024	3.526205
H	-5.331785	-1.132904	1.120843	C	2.079858	2.133738	3.713201
H	-6.490983	0.230533	1.154140	H	0.532713	1.333035	2.440489
H	-6.030843	-0.516069	-0.423161	C	1.886047	4.479078	4.270845
N	-5.165238	4.489258	0.773012	H	0.168905	5.510731	3.492668
C	-5.559350	4.290918	2.211412	C	2.577408	3.265790	4.366534
H	-5.484443	3.223938	2.450983	H	2.614689	1.183053	3.777662
H	-4.868503	4.859647	2.843873	H	2.260593	5.365260	4.788973
H	-6.586115	4.650326	2.347477	H	3.499404	3.203679	4.949693

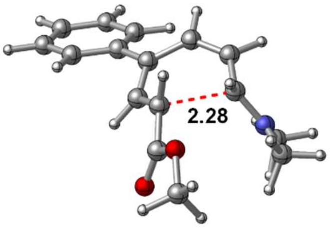

Zero-point correction= 0.315729 (Hartree/Particle)

Thermal correction to Energy= 0.334345

Thermal correction to Enthalpy= 0.335289

Thermal correction to Gibbs Free Energy= 0.267895

Sum of electronic and zero-point Energies= -825.406950

Sum of electronic and thermal Energies= -825.388333

Sum of electronic and thermal Enthalpies= -825.387389

Sum of electronic and thermal Free Energies= -825.454784

TS-3b-dd
E(scf) = -825.719040934 a.u.
ν_{min} = -276.49 cm^{-1}

C	-1.547054	4.106997	0.269649	C	-2.850816	1.548967	0.581097
C	-0.823768	2.863735	0.199963	H	-3.075396	1.991090	1.550823
C	-1.527881	1.668159	0.107577	C	-2.880299	4.344852	0.022586
H	-0.931139	5.009511	0.331151	C	-3.693194	0.431215	0.170332
H	-1.080183	0.814194	-0.414938	O	-3.460844	-0.360195	-0.728004

O	-4.852664	0.392207	0.883719	C	-6.099912	2.798925	-1.142756
C	-3.988156	3.407018	-0.079620	H	-6.852846	3.511498	-1.519268
H	-4.508882	3.093769	0.821668	H	-6.134182	1.897497	-1.779443
H	-3.135298	5.365426	-0.292144	H	-6.360119	2.511018	-0.116774
C	-5.792044	-0.612710	0.510099	C	0.646584	2.880512	-0.001007
H	-6.127548	-0.472052	-0.530238	C	1.431007	1.788002	0.430165
H	-5.354365	-1.619034	0.598963	C	1.317247	3.949686	-0.635024
H	-6.644026	-0.510704	1.194103	C	2.813113	1.759016	0.228637
N	-4.773633	3.394766	-1.173988	H	0.946812	0.959418	0.952481
C	-4.291287	3.794043	-2.486307	C	2.700809	3.927304	-0.825118
H	-4.465525	2.982333	-3.213256	H	0.746895	4.801668	-1.011317
H	-4.817342	4.694787	-2.845951	C	3.458695	2.831002	-0.397132
H	-3.215981	4.008468	-2.442312	H	3.391518	0.898786	0.576597

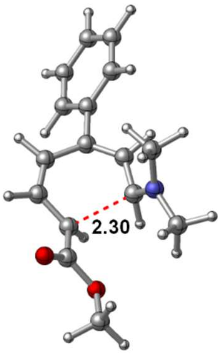

Zero-point correction= 0.315421 (Hartree/Particle)

Thermal correction to Energy= 0.334124

Thermal correction to Enthalpy= 0.335069

Thermal correction to Gibbs Free Energy= 0.267411

Sum of electronic and zero-point Energies= -825.403620

Sum of electronic and thermal Energies= -825.384916

Sum of electronic and thermal Enthalpies= -825.383972

Sum of electronic and thermal Free Energies= -825.451630

TS-3b-du

E(scf) = -825.711208173 a.u.

ν_{min} = -604.51 cm^{-1}

C	-1.825050	4.186135	0.848739	H	-1.664171	0.982769	-0.401062
C	-1.251318	2.961139	0.268977	C	-3.336650	1.678047	0.778941
C	-2.038841	1.825142	0.191413	H	-3.558946	2.170960	1.726443
H	-1.152864	4.869300	1.379900	C	-3.132471	4.527591	0.765824

C	-4.316408	0.720803	0.379879	H	-5.371885	4.609845	2.036886
O	-5.422861	0.559859	0.905017	C	-6.253746	3.140284	-0.901967
O	-3.966396	0.010665	-0.743264	H	-6.667735	2.201740	-0.507673
C	-4.071772	3.738376	-0.053605	H	-7.073152	3.834686	-1.142405
H	-3.722556	3.443866	-1.049290	H	-5.679950	2.925772	-1.811718
H	-3.508344	5.431781	1.249486	C	0.135574	2.995644	-0.228194
C	-4.936512	-0.897438	-1.243924	C	0.921841	1.823424	-0.336370
H	-5.234683	-1.635257	-0.481839	C	0.739064	4.212486	-0.626010
H	-5.845186	-0.370380	-1.580875	C	2.223736	1.863951	-0.837280
H	-4.472627	-1.410673	-2.097135	H	0.513007	0.868716	0.001940
N	-5.381921	3.748677	0.104032	C	2.047554	4.253367	-1.112933
C	-6.006217	3.980550	1.402850	H	0.164383	5.140826	-0.577723
H	-6.981305	4.466787	1.261530	C	2.800415	3.079282	-1.227619
H	-6.150648	3.003214	1.893068	H	2.801817	0.938065	-0.906024

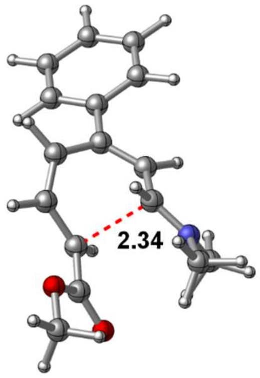

Zero-point correction= 0.315298 (Hartree/Particle)

Thermal correction to Energy= 0.333914

Thermal correction to Enthalpy= 0.334858

Thermal correction to Gibbs Free Energy= 0.267777

Sum of electronic and zero-point Energies= -825.395910

Sum of electronic and thermal Energies= -825.377295

Sum of electronic and thermal Enthalpies= -825.376350

Sum of electronic and thermal Free Energies= -825.443432

TS-3b-H-dd

E(scf) = -826.137686527 a.u.

ν_{min} = -452.60cm^{-1}

C	-1.542681	4.262377	0.310911	C	-0.874921	3.007683	0.383310

C	-1.471530	1.787860	0.113027	C	-5.287790	2.310449	-1.982795
H	-0.881364	1.000890	-0.368424	H	-5.798090	1.764595	-1.181939
C	-2.839293	1.562487	0.335683	H	-5.975491	2.503304	-2.814502
H	-3.239160	1.882026	1.294466	H	-4.416895	1.744427	-2.330597
C	-2.830358	4.385578	-0.236228	C	-5.973409	4.517357	-1.069011
C	-3.476508	0.343916	-0.229660	H	-6.589103	4.682431	-1.961783
O	-3.018428	-0.325067	-1.131150	H	-6.562052	4.020487	-0.288353
O	-4.656627	0.093712	0.360511	H	-5.578430	5.469355	-0.696420
C	-3.870753	3.440168	-0.279673	H	0.217350	3.056880	0.390095
H	-4.451894	3.157078	0.591954	C	-0.715786	5.489015	0.515662
H	-3.000280	5.304159	-0.812521	C	-0.789216	6.610163	-0.332783
C	-5.375732	-1.058231	-0.103828	C	0.176443	5.531679	1.603947
H	-5.615752	-0.959312	-1.173493	C	0.003770	7.735095	-0.098913
H	-4.777087	-1.969264	0.041661	H	-1.448506	6.602555	-1.203517
H	-6.294519	-1.104259	0.491746	C	0.963369	6.661340	1.842484
N	-4.818921	3.624636	-1.432352	H	0.239785	4.677531	2.282552

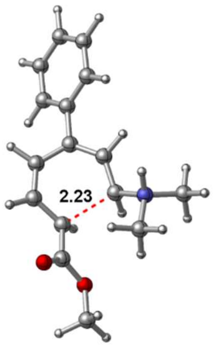

Zero-point correction= 0.331508 (Hartree/Particle)

Thermal correction to Energy= 0.349798

Thermal correction to Enthalpy= 0.350742

Thermal correction to Gibbs Free Energy= 0.283867

Sum of electronic and zero-point Energies= -825.806179

Sum of electronic and thermal Energies= -825.787888

Sum of electronic and thermal Enthalpies= -825.786944

Sum of electronic and thermal Free Energies= -825.853820

TS-3b-H-du
E(scf) = -826.134270537 a.u.
ν_{min} = -450.87 cm^{-1}

C	-2.136646	4.175484	0.329866	H	-1.672606	1.882950	2.730891
C	-1.532104	3.077089	0.993351	C	-3.518147	1.700045	1.661624
C	-2.197709	2.171720	1.810779	H	-4.019818	1.369542	2.577599

C	-3.521395	4.416248	0.422986	H	-7.593665	3.130657	1.937942
C	-3.937402	0.825403	0.532638	H	-6.544435	1.864136	1.191369
O	-4.915059	0.106085	0.584506	C	-6.687233	4.303585	-0.219850
O	-3.151156	0.921455	-0.546534	H	-6.850729	3.434273	-0.868175
C	-4.500761	3.427356	0.522556	H	-7.645238	4.690306	0.148171
H	-4.592492	2.695516	-0.273477	H	-6.140674	5.084014	-0.761072
H	-3.822111	5.466200	0.533646	H	-0.446832	3.135613	1.110763
C	-3.502421	0.110324	-1.677541	C	-1.286803	5.272067	-0.207883
H	-3.465299	-0.956235	-1.412060	C	-1.746929	6.010543	-1.317022
H	-4.515110	0.355731	-2.030556	C	-0.036472	5.611437	0.347166
H	-2.761485	0.337240	-2.452550	C	-0.982614	7.047527	-1.857049
N	-5.857924	3.861422	0.956494	H	-2.703074	5.748540	-1.776456
C	-6.563499	2.802454	1.757843	C	0.726007	6.648314	-0.192680
H	-6.032550	2.675073	2.706729	H	0.335193	5.083492	1.227455

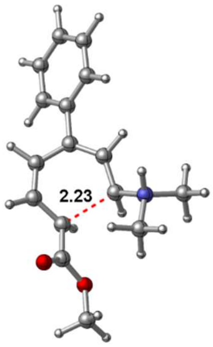

Zero-point correction= 0.331511 (Hartree/Particle)

Thermal correction to Energy= 0.349630

Thermal correction to Enthalpy= 0.350574

Thermal correction to Gibbs Free Energy= 0.284589

Sum of electronic and zero-point Energies= -825.802759

Sum of electronic and thermal Energies= -825.784640

Sum of electronic and thermal Enthalpies= -825.783696

Sum of electronic and thermal Free Energies= -825.849681

TS-3b-H-ud

E(scf) = -826.134117172 a.u.

ν_{min} = -462.23 cm^{-1}

C	-1.362173	4.544251	0.214540	C	-3.042853	1.925962	-0.281644
C	-0.918540	3.201603	0.131098	H	-3.502552	1.318830	-1.070064
C	-1.656180	2.138666	-0.385703	C	-2.658972	4.912112	-0.203955
H	-1.102247	1.453162	-1.041107	C	-3.729876	1.651844	1.009200

O	-4.803121	1.086472	1.074443	H	-3.850665	4.761668	-2.657240
O	-3.063256	2.091858	2.083862	C	-6.206082	3.711110	-0.405271
C	-3.788419	4.113753	-0.060185	H	-7.050610	3.974928	-1.051512
H	-4.074066	3.783556	0.931384	H	-6.003930	2.634606	-0.444611
H	-2.735691	5.843527	-0.778818	H	-6.411412	4.011710	0.628975
C	-3.668568	1.853699	3.363662	H	0.164136	3.059533	0.186411
H	-4.658543	2.330486	3.418901	C	-0.378867	5.639028	0.433321
H	-3.781243	0.774125	3.540360	C	-0.807141	6.821817	1.069683
H	-2.990355	2.293327	4.103854	C	0.967388	5.551123	0.025355
N	-4.987393	4.443347	-0.886211	C	0.078984	7.877482	1.296322
C	-4.747524	4.212229	-2.351001	H	-1.841219	6.901648	1.413522
H	-4.599552	3.137474	-2.503753	C	1.851801	6.606846	0.251618
H	-5.621085	4.567481	-2.910081	H	1.325183	4.664495	-0.501494

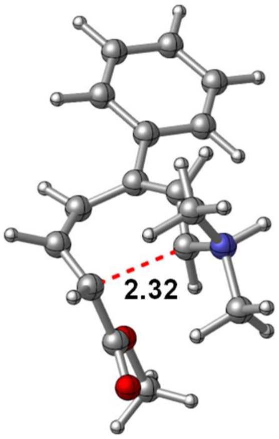

Zero-point correction= 0.331202 (Hartree/Particle)

Thermal correction to Energy= 0.349383

Thermal correction to Enthalpy= 0.350328

Thermal correction to Gibbs Free Energy= 0.284409

Sum of electronic and zero-point Energies= -825.802915

Sum of electronic and thermal Energies= -825.784734

Sum of electronic and thermal Enthalpies= -825.783790

Sum of electronic and thermal Free Energies= -825.849708

TS-3b-H-uu
E(scf) = -826.140727266 a.u.
ν_{min} = -464.38 cm^{-1}

C	-1.353401	4.564919	1.216503	N	-5.090750	4.493171	0.670013
C	-1.040355	3.291752	1.756961	C	-5.446280	4.372862	2.126156
C	-1.943536	2.248399	1.914730	H	-5.404860	3.315544	2.410808
H	-1.861837	1.631196	2.817001	H	-4.716652	4.940939	2.713834
C	-3.019076	2.001600	1.045274	H	-6.454168	4.777667	2.275141
H	-2.807227	1.940590	-0.017788	C	-6.068609	3.773385	-0.209413
C	-2.679872	4.911489	0.882976	H	-5.959078	2.696960	-0.038446
C	-4.124844	1.124751	1.515251	H	-7.082025	4.105109	0.043294
O	-4.387671	0.910368	2.680380	H	-5.842069	4.012023	-1.255348
O	-4.839454	0.632379	0.489586	H	-0.101473	3.226835	2.312665
C	-3.679381	4.059572	0.403441	C	-0.366005	5.675056	1.307745
H	-3.607284	3.587530	-0.569198	C	0.600903	5.755842	2.329982
H	-2.977803	5.928005	1.174238	C	-0.397057	6.696380	0.336858
C	-5.940719	-0.226048	0.826572	C	1.504145	6.818819	2.374937
H	-5.581998	-1.112436	1.369755	H	0.633795	5.000613	3.117411
H	-6.667946	0.307721	1.456582	C	0.508377	7.759190	0.381918
H	-6.398985	-0.519466	-0.124658	H	-1.123698	6.640388	-0.477268

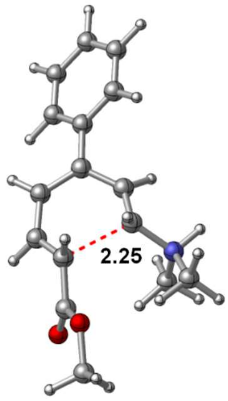

Zero-point correction= 0.331649 (Hartree/Particle)

Thermal correction to Energy= 0.349785

Thermal correction to Enthalpy= 0.350730

Thermal correction to Gibbs Free Energy= 0.284833

Sum of electronic and zero-point Energies= -825.809078

Sum of electronic and thermal Energies= -825.790942

Sum of electronic and thermal Enthalpies= -825.789998

Sum of electronic and thermal Free Energies= -825.855894

TS-3b-OH-dd'

E(scf) = -826.148431900 a.u.

ν_{min} = -318.87 cm^{-1}

C	-1.699789	4.302640	0.139861	H	-4.520623	4.188121	-3.188866
C	-1.091762	3.030920	-0.283235	H	-2.988131	4.216815	-2.269526
C	-1.721128	1.843961	-0.172074	C	-5.885780	2.629528	-1.472174
H	-1.241709	0.945121	-0.577580	H	-5.718495	1.619665	-1.885053
C	-3.013648	1.734280	0.527101	H	-6.424133	2.543579	-0.519245
H	-3.024769	2.060323	1.572894	H	-6.514664	3.186728	-2.182902
C	-3.046778	4.425905	0.244539	H	-0.098786	3.074541	-0.736382
C	-3.872497	0.644535	0.289048	C	-0.798194	5.460155	0.388088
O	-3.844009	-0.112661	-0.785347	C	-1.176965	6.773992	0.051749
O	-4.838894	0.399626	1.126169	C	0.465428	5.256840	0.975006
C	-4.067117	3.375099	-0.046140	C	-0.325678	7.850547	0.308575
H	-4.806163	3.242002	0.750120	H	-2.136719	6.952937	-0.438230
H	-3.491307	5.387725	0.519597	C	1.315084	6.334898	1.235914
C	-5.811016	-0.632992	0.841989	H	0.779911	4.246853	1.248919
H	-6.361025	-0.387536	-0.077556	C	0.922582	7.635737	0.904164
H	-5.309307	-1.603526	0.732178	H	-0.634326	8.861917	0.032691
H	-6.484936	-0.639175	1.704376	H	2.287533	6.157135	1.701657
N	-4.622396	3.330175	-1.276274	H	1.589170	8.478597	1.102487
C	-3.879693	3.622203	-2.495912	H	-3.095270	0.099882	-1.368546
H	-3.569880	2.688138	-2.996599				

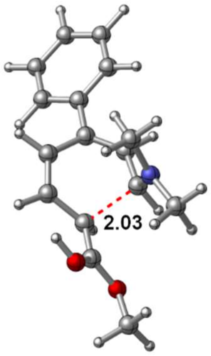

Zero-point correction= 0.329477 (Hartree/Particle)

Thermal correction to Energy= 0.347983

Thermal correction to Enthalpy= 0.348927

Thermal correction to Gibbs Free Energy= 0.282336

Sum of electronic and zero-point Energies= -825.818955

Sum of electronic and thermal Energies= -825.800449

Sum of electronic and thermal Enthalpies= -825.799505

Sum of electronic and thermal Free Energies= -825.866096

TS-3b-OH-du'

E(scf) = -826.143737584 a.u.

ν_{min} = -393.10cm^{-1}

C	-2.067592	4.467961	-0.313668	N	-5.567331	3.297520	-0.109783
C	-1.341620	3.192514	-0.166296	C	-6.475816	2.284428	-0.628968
C	-1.888905	2.117522	0.431114	H	-5.958601	1.649293	-1.360385
H	-1.326542	1.182295	0.498635	H	-6.876294	1.648460	0.177150
C	-3.253867	2.166905	1.021257	H	-7.328478	2.770576	-1.130549
H	-3.398942	2.858959	1.859512	C	-6.108125	4.132030	0.953197
C	-3.424073	4.456501	-0.311295	H	-5.441424	4.975622	1.169199
C	-3.969697	0.968647	1.185302	H	-7.090283	4.526326	0.652453
O	-5.018703	0.968295	1.980038	H	-6.235800	3.546637	1.881599
O	-3.639915	-0.078650	0.475169	H	-0.318352	3.135365	-0.544292
C	-4.229355	3.201152	-0.328361	C	-1.290976	5.725211	-0.446003
H	-3.983520	2.534701	-1.167354	C	-1.779042	6.813360	-1.196947
H	-3.985359	5.390706	-0.366365	C	-0.040935	5.859585	0.190027
C	-4.381895	-1.313994	0.527767	C	-1.049505	7.999221	-1.294571
H	-4.349400	-1.738281	1.542943	H	-2.727150	6.721009	-1.731330
H	-5.418927	-1.160257	0.191487	C	0.686694	7.048350	0.094983
H	-3.872057	-1.990601	-0.165654	H	0.357944	5.034184	0.784204

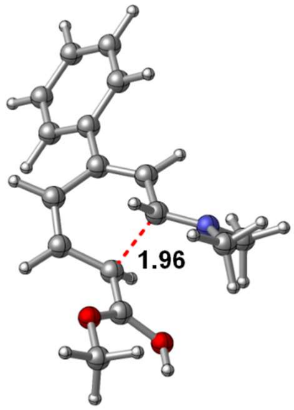

Zero-point correction= 0.328909 (Hartree/Particle)

Thermal correction to Energy= 0.347614

Thermal correction to Enthalpy= 0.348558

Thermal correction to Gibbs Free Energy= 0.281824

Sum of electronic and zero-point Energies= -825.814828

Sum of electronic and thermal Energies= -825.796123

Sum of electronic and thermal Enthalpies= -825.795179

Sum of electronic and thermal Free Energies= -825.861913

TS-3b-OH-ud’

E(scf) = -826.146344756 a.u.

ν_{min} = -239.51cm^{-1}

C	-1.797971	4.521436	0.022530	N	-4.820461	3.951592	-1.357924
C	-1.372698	3.250986	-0.592003	C	-4.055883	4.193955	-2.574873
C	-2.112009	2.127090	-0.575851	H	-3.739306	3.236982	-3.025640
H	-1.753267	1.239488	-1.107680	H	-4.686633	4.731014	-3.297965
C	-3.404385	2.010119	0.137570	H	-3.166660	4.794903	-2.354729
H	-4.171392	1.392169	-0.340635	C	-6.115703	3.313970	-1.558424
C	-3.108674	4.815566	0.190253	H	-6.707467	3.895611	-2.280286
C	-3.453306	1.979940	1.539135	H	-5.989202	2.292123	-1.959256
O	-4.522646	1.631087	2.230918	H	-6.663767	3.258761	-0.608760
O	-2.463114	2.445555	2.245747	H	-0.397235	3.231109	-1.083181
C	-4.254944	3.929976	-0.139643	C	-0.750539	5.486921	0.451120
H	-4.989968	3.825987	0.665729	C	-0.867169	6.155659	1.684785
H	-3.404710	5.787620	0.598382	C	0.374563	5.751768	-0.352800
C	-2.576699	2.577257	3.678915	C	0.103314	7.072546	2.095421
H	-3.408858	3.248250	3.931674	H	-1.715532	5.934264	2.336501
H	-2.734454	1.592353	4.139118	C	1.340828	6.674203	0.055995
H	-1.623909	3.007993	4.003528	H	0.487030	5.254776	-1.318910

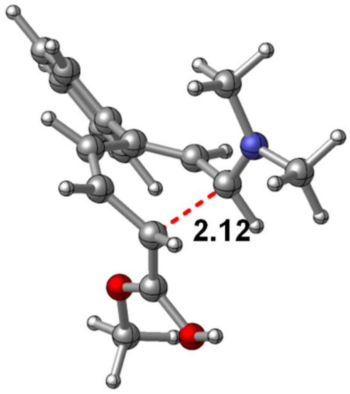

Zero-point correction= 0.329256 (Hartree/Particle)

Thermal correction to Energy= 0.347878

Thermal correction to Enthalpy= 0.348822

Thermal correction to Gibbs Free Energy= 0.282088

Sum of electronic and zero-point Energies= -825.817089

Sum of electronic and thermal Energies= -825.798467

Sum of electronic and thermal Enthalpies= -825.797523

Sum of electronic and thermal Free Energies= -825.864256

TS-3b-OH-uu'

E(scf) = -826.147456903 a.u.

ν_{min} = -288.49cm^{-1}

C	-1.616855	4.639521	1.350444	N	-4.978779	4.135521	1.261724
C	-1.478307	3.525118	2.298753	C	-4.892741	4.734812	2.587851
C	-2.142760	2.359656	2.161237	H	-5.012728	3.963974	3.369094
H	-2.034119	1.590149	2.934848	H	-3.925692	5.231381	2.722207
C	-2.942046	2.080760	0.956225	H	-5.696456	5.476805	2.708508
H	-2.405371	2.118008	0.002110	C	-6.263445	3.526007	0.939670
C	-2.719310	4.762877	0.568929	H	-6.472704	2.673198	1.608222
C	-3.960846	1.111230	0.969747	H	-7.069372	4.264283	1.068359
O	-4.568081	0.674207	2.051470	H	-6.268145	3.176868	-0.101020
O	-4.427038	0.658931	-0.159314	H	-0.802361	3.655434	3.145889
C	-3.893651	3.844743	0.516631	C	-0.550247	5.678464	1.304546
H	-4.153462	3.500759	-0.489580	C	0.060431	6.149212	2.482608
H	-2.853290	5.662355	-0.041762	C	-0.137218	6.220747	0.072627
C	-5.553848	-0.247228	-0.176663	C	1.039640	7.144352	2.429891
H	-5.314322	-1.158418	0.387474	H	-0.247139	5.757301	3.454517
H	-6.436806	0.242543	0.257905	C	0.846212	7.211295	0.020743
H	-5.721890	-0.477702	-1.233376	H	-0.576360	5.845001	-0.854405

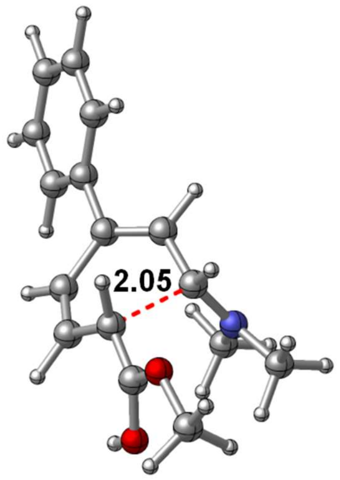

Zero-point correction= 0.329313 (Hartree/Particle)

Thermal correction to Energy= 0.347853

Thermal correction to Enthalpy= 0.348797

Thermal correction to Gibbs Free Energy= 0.282244

Sum of electronic and zero-point Energies= -825.818144

Sum of electronic and thermal Energies= -825.799604

Sum of electronic and thermal Enthalpies= -825.798660

Sum of electronic and thermal Free Energies= -825.865213

TS-3b-ud

E(scf) = -825.711936346 a.u.

ν_{min} = -334.00cm^{-1}

C	-1.425807	4.442958	0.218764	N	-4.907847	4.194016	-0.997581
C	-0.905098	3.107922	0.101912	C	-4.630176	4.595298	-2.366625
C	-1.628663	2.002677	-0.279339	H	-4.703113	3.724358	-3.045779
H	-1.082590	1.218903	-0.824736	H	-5.357664	5.352222	-2.700303
C	-3.032504	1.815431	-0.105978	H	-3.621434	5.016586	-2.450226
H	-3.533491	1.159932	-0.827620	C	-6.181264	3.520553	-0.802909
C	-2.727855	4.810935	-0.077850	H	-7.007442	4.159017	-1.153167
C	-3.662647	1.672518	1.204480	H	-6.213473	2.570488	-1.369154
O	-4.747699	1.141414	1.395391	H	-6.329537	3.285947	0.259115
O	-2.956645	2.224140	2.224145	H	0.185054	3.023783	0.090651
C	-3.916527	3.997083	-0.097974	C	-0.446218	5.554970	0.422498
H	-4.279644	3.662893	0.869207	C	-0.792796	6.647639	1.241459
H	-2.876843	5.844144	-0.413075	C	0.823681	5.564112	-0.187822
C	-3.541761	2.136852	3.521319	C	0.094382	7.708428	1.445069
H	-4.522467	2.637646	3.549760	H	-1.765861	6.650410	1.738259
H	-3.679934	1.087610	3.825959	C	1.710382	6.624840	0.012954
H	-2.845570	2.637642	4.206112	H	1.113904	4.745124	-0.849381

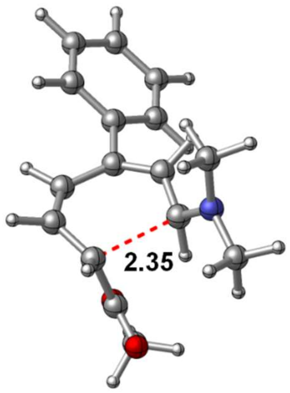

Zero-point correction= 0.315371 (Hartree/Particle)

Thermal correction to Energy= 0.334058

Thermal correction to Enthalpy= 0.335002

Thermal correction to Gibbs Free Energy= 0.267122

Sum of electronic and zero-point Energies= -825.396565

Sum of electronic and thermal Energies= -825.377879

Sum of electronic and thermal Enthalpies= -825.376934

Sum of electronic and thermal Free Energies= -825.444814

TS-3b-uu
E(scf) = -825.719525678 a.u.
ν_{min} = -292.22cm^{-1}

C	-1.422207	4.469523	1.262841	N	-5.042100	4.189452	1.022262
C	-1.050753	3.254791	1.934769	C	-5.212285	4.922239	2.265601
C	-1.856240	2.138812	1.988745	H	-5.858497	4.350218	2.953609
H	-1.771928	1.447589	2.836894	H	-4.237883	5.082347	2.744770
C	-2.838093	1.891962	1.000525	H	-5.684457	5.904458	2.090813
H	-2.533585	2.014737	-0.037058	C	-6.254479	3.631609	0.447019
C	-2.694107	4.757677	0.778512	H	-6.743678	2.944641	1.160183
C	-3.927736	0.954629	1.242733	H	-6.971824	4.432910	0.200716
O	-4.238969	0.465902	2.316173	H	-6.016730	3.071576	-0.465742
O	-4.641882	0.715042	0.107875	H	-0.162511	3.296482	2.569903
C	-3.824932	3.889596	0.518610	C	-0.456403	5.612467	1.302562
H	-3.879914	3.343473	-0.419172	C	0.325462	5.896350	2.439683
H	-2.952787	5.823688	0.751424	C	-0.319333	6.453417	0.180951
C	-5.791171	-0.115824	0.247735	C	1.207850	6.979433	2.453586
H	-5.519698	-1.108513	0.639110	H	0.225524	5.279466	3.335231
H	-6.524687	0.337449	0.934333	C	0.565620	7.535508	0.192317
H	-6.228258	-0.212651	-0.754161	H	-0.904805	6.240031	-0.716466

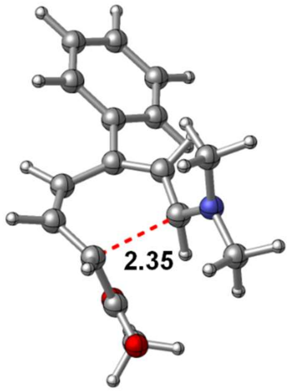

Zero-point correction= 0.315467 (Hartree/Particle)

Thermal correction to Energy= 0.334150

Thermal correction to Enthalpy= 0.335094

Thermal correction to Gibbs Free Energy= 0.267470

Sum of electronic and zero-point Energies= -825.404059

Sum of electronic and thermal Energies= -825.385376

Sum of electronic and thermal Enthalpies= -825.384431

Sum of electronic and thermal Free Energies= -825.452055

3a-CN
E(scf) = -917.942063716 a.u.
ν_{min} = 30.77 cm^{-1}

C	-0.161761	2.243007	0.424137	H	-6.846675	-0.808015	-0.658667
C	-1.497747	2.604919	0.383825	H	-7.812986	0.528538	-1.382774
C	-2.461018	1.635273	-0.071618	N	3.352013	3.114793	1.243359
H	0.066752	1.225352	0.082948	C	3.301319	4.470753	1.766198
H	-2.060506	0.645789	-0.322820	H	2.868757	5.163729	1.024866
C	-3.802148	1.795434	-0.248720	H	2.686839	4.521756	2.681600
H	-4.310927	2.738940	-0.045631	H	4.316441	4.808717	2.008175
C	0.945400	3.011830	0.873973	C	4.646467	2.453855	1.191531
C	-4.624368	0.687955	-0.738605	H	5.351039	3.020123	0.559207
O	-4.241373	-0.437606	-1.012599	H	5.084642	2.365641	2.200417
O	-5.922641	1.057609	-0.865020	H	4.536728	1.445268	0.771750
C	2.225941	2.494731	0.847026	C	-1.947876	3.964108	0.796233
H	2.374586	1.478105	0.467539	C	-2.889063	4.130762	1.831222
H	0.770653	4.020119	1.252125	C	-1.445002	5.116723	0.161978
C	-6.822561	0.058148	-1.338866	C	-3.310131	5.397320	2.226970
H	-6.529146	-0.297101	-2.339437	H	-3.290026	3.249693	2.336012

C	-1.856180	6.390641	0.545710	H	-4.034792	5.508516	3.035851
H	-0.723001	5.006805	-0.649634	H	-1.458840	7.272941	0.040219
C	-2.794444	6.540349	1.585105	C	-3.223029	7.849033	1.987253

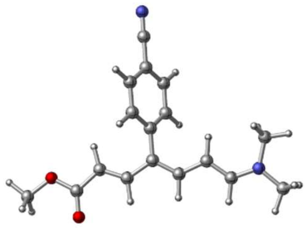

Zero-point correction= 0.315475 (Hartree/Particle)

Thermal correction to Energy= 0.336941

Thermal correction to Enthalpy= 0.337885

Thermal correction to Gibbs Free Energy= 0.262229

Sum of electronic and zero-point Energies= -917.626589

Sum of electronic and thermal Energies= -917.605123

Sum of electronic and thermal Enthalpies= -917.604179

Sum of electronic and thermal Free Energies= -917.6798

3a-NMe₂

E(scf) = -959.644321276 a.u.

ν_{min} = 22.16 cm^{-1}

C	-0.158732	2.216616	0.411813	C	3.289891	4.469058	1.761959
C	-1.491391	2.586337	0.377554	H	2.815251	5.155933	1.040924
C	-2.450783	1.615659	-0.083908	H	2.704351	4.501788	2.698020
H	0.073385	1.203802	0.057546	H	4.303769	4.832186	1.971933
H	-2.052807	0.625593	-0.338278	C	4.650113	2.457413	1.211995
C	-3.793529	1.778813	-0.254769	H	5.382274	3.037032	0.624644
H	-4.293786	2.724149	-0.039976	H	5.049550	2.337816	2.234621
C	0.948946	2.988883	0.865667	H	4.555911	1.460948	0.760167
C	-4.622103	0.679048	-0.745427	C	-1.941073	3.947700	0.790564
O	-4.248608	-0.448254	-1.028614	C	-2.864438	4.135538	1.834499
O	-5.921769	1.054322	-0.862850	C	-1.463447	5.107298	0.155408
C	2.232465	2.487316	0.832241	C	-3.287096	5.402390	2.232263
H	2.392912	1.474513	0.446944	H	-3.263462	3.261676	2.356353
H	0.760831	3.992671	1.249714	C	-1.871677	6.384726	0.535733
C	-6.826517	0.060303	-1.336393	H	-0.750603	5.006863	-0.667519
H	-6.540565	-0.291461	-2.340522	C	-2.799562	6.573137	1.593498
H	-6.849943	-0.809862	-0.661051	H	-4.001348	5.476148	3.051530
H	-7.816243	0.533191	-1.372526	H	-1.465341	7.239988	-0.003075
N	3.357169	3.120376	1.226766	N	-3.209272	7.832704	1.981396

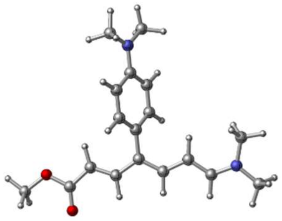

Zero-point correction= 0.388933 (Hartree/Particle)

Thermal correction to Energy= 0.413152

Thermal correction to Enthalpy= 0.414096

Thermal correction to Gibbs Free Energy= 0.331359

Sum of electronic and zero-point Energies= -959.255389

Sum of electronic and thermal Energies= -959.231170

Sum of electronic and thermal Enthalpies= -959.230225

Sum of electronic and thermal Free Energies= -959.312963

3a-OH-Cl

E(scf) = -1286.46489884 a.u.

ν_{min} = 21.77 cm^{-1}

C	-1.372853	4.504752	0.954385	H	-4.333828	3.257382	3.105109
C	-1.134815	3.384607	1.885571	H	-3.455857	4.787617	2.755838
C	-1.957787	2.313829	1.818970	H	-5.205979	4.832072	3.111116
H	-1.878377	1.483026	2.521678	C	-6.051326	3.491792	0.985713
C	-2.996139	2.214186	0.735142	H	-6.154639	2.575687	1.579330
H	-2.481492	1.884017	-0.188118	H	-6.807510	4.223127	1.300416
C	-2.502835	4.617756	0.233765	H	-6.187991	3.271840	-0.079027
C	-4.052175	1.157427	1.015747	H	-0.610059	5.283248	0.877113
O	-4.297338	0.687948	2.103652	C	-0.044503	3.489659	2.888298
O	-4.702696	0.826424	-0.103878	C	0.673385	2.350724	3.303419
C	-3.598584	3.579940	0.322953	C	0.288384	4.732118	3.462701
H	-4.067125	3.461787	-0.662564	C	1.678489	2.448632	4.267714
H	-2.712757	5.498194	-0.375772	H	0.454761	1.381928	2.848587
C	-5.758676	-0.138570	0.015891	C	1.293693	4.829630	4.428670
H	-5.376629	-1.075686	0.445626	H	-0.258240	5.631447	3.169233
H	-6.562865	0.248214	0.659867	C	1.992945	3.688752	4.835666
H	-6.133701	-0.305464	-1.000112	H	2.227031	1.552821	4.569924
N	-4.718708	4.117585	1.197225	H	1.528843	5.802627	4.867478
C	-4.402796	4.249666	2.641221	H	2.782454	3.765718	5.587365

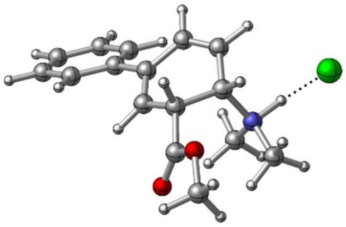

Zero-point correction= 0.332894 (Hartree/Particle)

Thermal correction to Energy= 0.352940

Thermal correction to Enthalpy= 0.353884

Thermal correction to Gibbs Free Energy= 0.282362

Sum of electronic and zero-point Energies= -1286.132005

Sum of electronic and thermal Energies= -1286.111959

Sum of electronic and thermal Enthalpies= -1286.111015

Sum of electronic and thermal Free Energies= -1286.182537

3b-CN

E(scf) = -917.939234334 a.u.

ν_{min} =24.62 cm^{-1}

C	0.185701	2.630576	-0.753421	H	2.062141	3.669819	-4.409342
C	-1.181832	2.480275	-0.882145	H	1.754284	5.306091	-3.763267
C	-2.040451	1.822030	0.050622	H	3.322194	4.928255	-4.531384
H	-1.593188	1.381252	0.947956	C	4.485580	4.421290	-2.359545
C	-3.390697	1.686075	-0.080266	H	5.133373	3.947760	-3.116616
H	-3.929204	2.093473	-0.939659	H	4.669961	5.509564	-2.382751
C	0.964884	3.259949	-1.784484	H	4.775833	4.043073	-1.370334
C	-4.174646	0.983896	0.936290	H	-1.648944	2.881911	-1.788630
O	-3.744399	0.469441	1.955627	C	0.881603	2.131869	0.473249
O	-5.492261	0.961137	0.618027	C	0.665063	2.744417	1.719580
C	2.310073	3.538808	-1.677742	C	1.766937	1.041753	0.397873
H	2.838112	3.278775	-0.756483	C	1.310515	2.283548	2.865311
H	0.423162	3.548819	-2.688010	H	-0.018499	3.592999	1.787290
C	-6.354923	0.300888	1.541864	C	2.415146	0.566816	1.535401
H	-6.075348	-0.758314	1.657850	H	1.942063	0.559122	-0.565744
H	-6.313750	0.778732	2.533583	C	2.190807	1.188714	2.778885
H	-7.368764	0.378701	1.129289	H	1.136066	2.767741	3.827828
N	3.086233	4.121224	-2.614671	H	3.093787	-0.285348	1.467310
C	2.526061	4.526760	-3.892097	C	2.859532	0.707820	3.954635

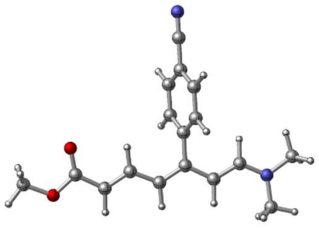

Zero-point correction= 0.315037 (Hartree/Particle)
Thermal correction to Energy= 0.336667
Thermal correction to Enthalpy= 0.337611
Thermal correction to Gibbs Free Energy= 0.261096
Sum of electronic and zero-point Energies= -917.624197
Sum of electronic and thermal Energies= -917.602568
Sum of electronic and thermal Enthalpies= -917.601624
Sum of electronic and thermal Free Energies= -917.678138

3b-NMe$_2$
E(scf) = -959.643747074 a.u.
ν_{min} = 24.82cm^{-1}

C	0.209894	2.616378	-0.723447	H	2.028433	3.612953	-4.372030
C	-1.162244	2.474488	-0.864137	H	1.719448	5.292951	-3.851963
C	-2.029130	1.772109	0.023441	H	3.288307	4.857374	-4.589304
H	-1.584276	1.260107	0.882974	C	4.394185	4.669188	-2.327076
C	-3.384706	1.665810	-0.109323	H	5.112853	4.299106	-3.077126
H	-3.921027	2.149566	-0.929781	H	4.438741	5.773456	-2.326426
C	0.969217	3.254509	-1.770141	H	4.711944	4.311740	-1.338310
C	-4.173996	0.898761	0.849564	H	-1.625045	2.917161	-1.754190
O	-3.752383	0.292361	1.822320	C	0.909747	2.124630	0.496607
O	-5.496902	0.926779	0.540724	C	0.546530	2.565108	1.782138
C	2.292836	3.614357	-1.661824	C	1.962435	1.193884	0.418149
H	2.817753	3.431431	-0.720694	C	1.190031	2.108720	2.929331
H	0.423356	3.490848	-2.686861	H	-0.259054	3.296335	1.885171
C	-6.364241	0.209485	1.414350	C	2.611612	0.715468	1.552730
H	-6.107962	-0.861837	1.438096	H	2.271635	0.821145	-0.561667
H	-6.304500	0.599500	2.443087	C	2.246145	1.162064	2.851352
H	-7.380436	0.342667	1.021606	H	0.865852	2.498450	3.893310
N	3.053645	4.190878	-2.619479	H	3.406894	-0.017403	1.423112
C	2.492237	4.505529	-3.920549	N	2.886080	0.699835	3.980063

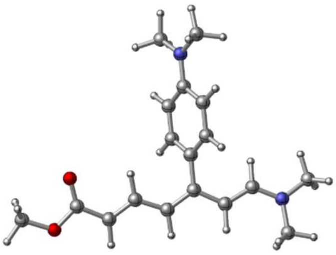

Zero-point correction= 0.388869 (Hartree/Particle)

Thermal correction to Energy= 0.413028

Thermal correction to Enthalpy= 0.413972

Thermal correction to Gibbs Free Energy= 0.332185

Sum of electronic and zero-point Energies= -959.254878

Sum of electronic and thermal Energies= -959.230719

Sum of electronic and thermal Enthalpies= -959.229775

Sum of electronic and thermal Free Energies= -959.311562

3b-OH-Cl

E(scf) = 31.48 a.u.
ν_{min} = -1286.46292721 cm^{-1}

C	1.011009	1.182557	-0.015485	H	-2.378610	-2.077609	-1.880585
C	1.801852	0.018226	-0.452527	H	-1.725310	-1.263848	-3.348487
C	1.320004	-1.229685	-0.324429	H	-0.611320	-2.077929	-2.233695
H	1.887573	-2.113578	-0.619643	C	-2.577593	0.645305	-1.764960
C	-0.043214	-1.421031	0.283554	H	-2.653432	0.879266	-2.833624
H	0.070688	-1.290423	1.381687	H	-3.476605	0.123333	-1.410110
C	-0.311745	1.035248	0.237076	H	-2.443855	1.582780	-1.213916
C	-0.583701	-2.839644	0.167758	H	2.791753	0.196597	-0.876582
O	0.071625	-3.799671	-0.160513	C	1.683238	2.499346	0.103733
O	-1.870811	-2.903027	0.527638	C	1.089035	3.649420	-0.447311
C	-0.995601	-0.276155	-0.088428	C	2.930427	2.619815	0.742971
H	-1.932303	-0.400101	0.469005	C	1.714006	4.893774	-0.334735
H	-0.916661	1.881250	0.567052	H	0.158293	3.545038	-1.008147
C	-2.483064	-4.201855	0.514951	C	3.551940	3.865933	0.859539
H	-2.457113	-4.627796	-0.499068	H	3.408468	1.732341	1.165923
H	-1.957474	-4.880905	1.201919	C	2.943697	5.007149	0.323983
H	-3.518648	-4.052129	0.840676	H	1.245595	5.777556	-0.775550
N	-1.381038	-0.217117	-1.570729	H	4.515111	3.946485	1.369897
C	-1.541243	-1.502646	-2.293326	H	3.432875	5.980786	0.409793

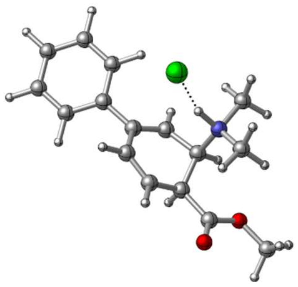

Zero-point correction= 0.333022 (Hartree/Particle)

Thermal correction to Energy= 0.353024

Thermal correction to Enthalpy= 0.353968

Thermal correction to Gibbs Free Energy= 0.283172

Sum of electronic and zero-point Energies= -1286.129905

Sum of electronic and thermal Energies= -1286.109903

Sum of electronic and thermal Enthalpies= -1286.108959

Sum of electronic and thermal Free Energies= -1286.179755

4a

E(scf) = -1286.47968161 a.u.

ν_{min} = 15.31 cm^{-1}

C	-1.256223	4.749386	2.080816	C	-7.302272	4.126061	1.464088
C	-0.744132	3.493826	2.464015	H	-6.895158	3.190676	1.880717
C	-1.603699	2.384756	2.398778	H	-7.303877	4.876318	2.270439
H	-1.257871	1.398102	2.711601	H	-8.358872	3.933868	1.172331
C	-2.928657	2.514566	1.948373	C	-6.486292	3.693328	-0.787727
H	-1.080946	1.982612	0.261665	H	-6.024802	2.728040	-0.524001
C	-2.575009	4.881507	1.641095	H	-7.509234	3.484186	-1.172736
C	-3.753396	1.269644	1.887057	H	-5.896989	4.130291	-1.609215
O	-3.339275	0.164065	2.166292	H	-0.604488	5.625712	2.105012
O	-5.012678	1.494302	1.480814	C	0.665456	3.338241	2.909122
C	-3.421897	3.771985	1.565353	C	1.426300	2.224284	2.504967
H	-4.446585	3.902135	1.205035	C	1.273552	4.301326	3.736175
H	-2.949310	5.862399	1.337607	C	2.752203	2.077070	2.919902
C	-5.867285	0.350168	1.379178	H	0.987110	1.485822	1.830757
H	-5.459848	-0.374782	0.658820	C	2.598951	4.151945	4.152275
H	-5.973480	-0.142301	2.357264	H	0.695923	5.164684	4.075121
H	-6.837433	0.726403	1.034144	C	3.343367	3.038747	3.746632
N	-6.459160	4.575437	0.367944	H	3.329055	1.210134	2.587836

H	3.050295	4.906008	4.802138	H	4.380291	2.922452	4.071641

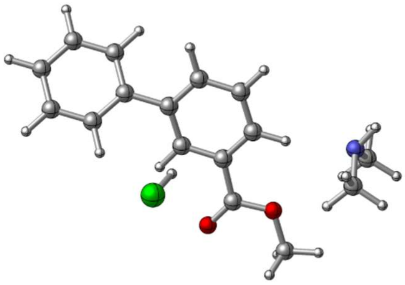

Zero-point correction= 0.326046 (Hartree/Particle)

Thermal correction to Energy= 0.349029

Thermal correction to Enthalpy= 0.349973

Thermal correction to Gibbs Free Energy= 0.268850

Sum of electronic and zero-point Energies= -1286.153636

Sum of electronic and thermal Energies= -1286.130653

Sum of electronic and thermal Enthalpies= -1286.129708

Sum of electronic and thermal Free Energies= -1286.210832

4b

E(scf) = -1286.48029470 a.u.

ν_{min} = 18.56 cm^{-1}

C	1.132613	1.193749	0.259997	C	-2.533643	-1.726717	-3.755208
C	1.591007	0.082162	-0.473992	H	-3.315999	-2.460414	-3.495312
C	0.774365	-1.026882	-0.677695	H	-2.861372	-1.215706	-4.688166
H	1.132272	-1.886432	-1.247118	H	-1.615248	-2.292395	-3.976999
C	-0.527850	-1.059288	-0.158219	C	-3.454203	-0.049612	-2.238126
H	1.036006	-0.271263	2.322907	H	-3.881016	0.581917	-3.049213
C	-0.178988	1.153584	0.776278	H	-4.258802	-0.716633	-1.883777
C	-1.362039	-2.271851	-0.407474	H	-3.181281	0.610297	-1.399743
O	-0.974464	-3.258192	-0.998733	H	2.599757	0.092392	-0.891794
O	-2.579078	-2.181493	0.153194	C	2.020369	2.353520	0.524889
C	-0.998637	0.041451	0.573379	C	1.531367	3.672070	0.464314
H	-2.005844	0.019626	0.989845	C	3.372525	2.152901	0.862946
H	-0.550759	1.992924	1.368001	C	2.371582	4.757688	0.723955
C	-3.471107	-3.267585	-0.093512	H	0.488327	3.848480	0.190581
H	-3.670014	-3.362507	-1.171888	C	4.210894	3.238886	1.126046
H	-3.049353	-4.216084	0.272183	H	3.757985	1.134872	0.953560
H	-4.398951	-3.032314	0.441836	C	3.714576	4.545342	1.055046
N	-2.306363	-0.837172	-2.634921	H	1.976622	5.774942	0.662229

H	5.255212	3.063244	1.396485	H	4.371079	5.394736	1.259731

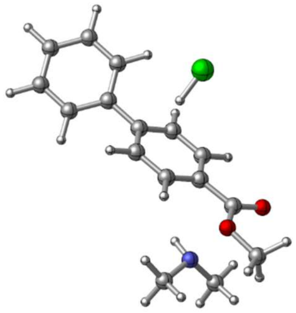

Zero-point correction= 0.325271 (Hartree/Particle)
Thermal correction to Energy= 0.348527
Thermal correction to Enthalpy= 0.349472
Thermal correction to Gibbs Free Energy= 0.267233
Sum of electronic and zero-point Energies= -1286.155024
Sum of electronic and thermal Energies= -1286.131767
Sum of electronic and thermal Enthalpies= -1286.130823
Sum of electronic and thermal Free Energies= -1286.213062

TS-3a-CN-r
E(scf) = -917.896137009 a.u.
ν_{min} = -347.98cm^{-1}

C	-0.291692	3.292653	-0.428516	C	2.987182	2.706835	2.269227
C	-1.687240	3.422865	0.059912	H	3.598014	2.670730	3.177511
C	-2.417743	2.232063	0.206587	H	3.177927	1.804401	1.670689
H	-0.109374	3.337087	-1.513321	H	3.261036	3.597540	1.688158
H	-3.470400	2.317592	0.495666	C	1.262122	2.587408	4.072582
C	-1.970409	0.928105	0.041933	H	1.645873	1.611232	4.404385
H	-0.939698	0.697822	-0.233937	H	1.753924	3.381056	4.654089
C	0.798603	3.085756	0.359571	H	0.178527	2.629945	4.232122
C	-2.865148	-0.184153	0.245305	C	-2.212299	4.743529	0.298338
O	-4.049550	-0.141210	0.566569	C	-3.550079	4.983348	0.743884
O	-2.236741	-1.385777	0.040842	C	-1.405062	5.906756	0.099234
C	0.620905	2.947606	1.772851	C	-4.030170	6.259713	0.971040
H	-0.406206	2.985866	2.150042	H	-4.222226	4.142266	0.918433
H	1.790626	2.991717	-0.085006	C	-1.882578	7.187114	0.325363
C	-3.034937	-2.547652	0.217874	H	-0.373053	5.792251	-0.239790
H	-3.428438	-2.615422	1.245675	C	-3.208431	7.396720	0.768612
H	-3.893587	-2.557421	-0.473632	H	-5.058853	6.395359	1.313565
H	-2.384377	-3.408619	0.012876	H	-1.227710	8.045854	0.159058
N	1.567700	2.766957	2.650259	C	-3.703335	8.711732	1.005951

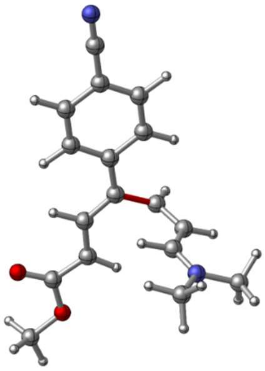

Zero-point correction= 0.313888 (Hartree/Particle)

Thermal correction to Energy= 0.334971

Thermal correction to Enthalpy= 0.335915

Thermal correction to Gibbs Free Energy= 0.261130

Sum of electronic and zero-point Energies= -917.582249

Sum of electronic and thermal Energies= -917.561166

Sum of electronic and thermal Enthalpies= -917.560222

Sum of electronic and thermal Free Energies= -917.635007

TS-3a-NMe$_2$-r

E(scf) = -959.586084050 a.u.

ν_{min} = - 704.70cm^{-1}

C	-0.236286	3.392531	-0.395582	H	-2.215536	-3.353484	0.057720
C	-1.643163	3.495060	0.063439	N	1.491244	2.538851	2.685597
C	-2.350990	2.308058	0.188946	C	2.927284	2.541855	2.374894
H	-0.016502	3.514199	-1.468318	H	3.494466	2.462289	3.308854
H	-3.409549	2.378004	0.463824	H	3.174910	1.685853	1.729775
C	-1.877613	0.990478	0.042748	H	3.204355	3.474884	1.866835
H	-0.838564	0.778278	-0.215026	C	1.116478	2.164102	4.051132
C	0.826404	3.116841	0.410604	H	1.505205	1.158749	4.272147
C	-2.748331	-0.124080	0.239000	H	1.555537	2.879997	4.761646
O	-3.945138	-0.113105	0.539488	H	0.025041	2.164061	4.153749
O	-2.093364	-1.330164	0.060589	C	-2.179808	4.836064	0.321391
C	0.584213	2.814545	1.787554	C	-3.488161	5.068310	0.812540
H	-0.460888	2.795890	2.111560	C	-1.411754	6.001770	0.088438
H	1.835484	3.055676	-0.001233	C	-3.984333	6.346151	1.052697
C	-2.878269	-2.495534	0.239921	H	-4.147325	4.222088	1.018610
H	-3.290642	-2.559939	1.261531	C	-1.895091	7.288891	0.328745
H	-3.727513	-2.530794	-0.463686	H	-0.393802	5.907783	-0.300837

C	-3.198849	7.507109	0.832744	H	-1.236341	8.130678	0.115329
H	-5.004217	6.434388	1.427183	N	-3.683629	8.785070	1.103247

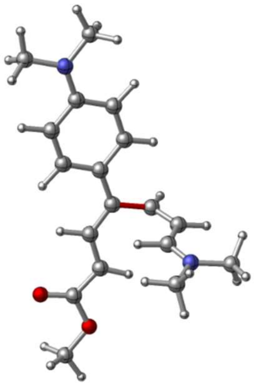

Zero-point correction= 0.386698 (Hartree/Particle)

Thermal correction to Energy= 0.410541

Thermal correction to Enthalpy= 0.411485

Thermal correction to Gibbs Free Energy= 0.329982

Sum of electronic and zero-point Energies= -959.199386

Sum of electronic and thermal Energies= -959.175543

Sum of electronic and thermal Enthalpies= -959.174599

Sum of electronic and thermal Free Energies= -959.256102

TS-3a-OH-Cl

E(scf) = -1286.43540909a.u.

ν_{min} = - 885.32cm^{-1}

C	-1.450874	4.494621	1.026714	H	-2.678819	5.322503	-0.504939
C	-1.194426	3.426580	1.984129	C	-5.750828	-0.134073	0.042769
C	-2.034968	2.347064	1.942994	H	-5.121557	-0.953189	-0.337693
H	-1.885773	1.489523	2.601792	H	-6.180901	-0.437485	1.009087
C	-3.107752	2.239525	0.967320	H	-6.549721	0.091669	-0.674200
H	-2.432613	1.817224	-0.160911	N	-4.834652	4.222485	1.194707
C	-2.549844	4.528121	0.235089	C	-4.596274	4.216520	2.663345
C	-3.953123	1.030600	1.052307	H	-4.652535	3.183488	3.025245
O	-3.728095	0.061885	1.751850	H	-3.599700	4.625084	2.862705
O	-4.984223	1.064420	0.176076	H	-5.363372	4.827916	3.155955
C	-3.634537	3.506021	0.361588	C	-6.204083	3.751653	0.845363
H	-4.126384	3.302072	-0.597541	H	-6.333183	2.728059	1.206945

H	-6.939667	4.418723	1.313691	H	0.361621	1.393950	2.961020
H	-6.321247	3.769695	-0.244793	C	1.434605	4.864612	4.329036
H	-0.698775	5.279688	0.910357	H	-0.157706	5.679534	3.131493
C	-0.054042	3.521520	2.926878	C	2.107997	3.713687	4.750783
C	0.642398	2.372561	3.357030	H	2.228537	1.559307	4.570680
C	0.368198	4.769373	3.430306	H	1.736082	5.845341	4.706778
C	1.703774	2.466465	4.259165	H	2.942733	3.787117	5.452373

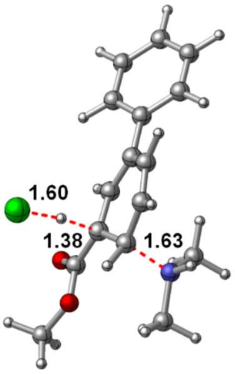

Zero-point correction= 0.328032 (Hartree/Particle)

Thermal correction to Energy= 0.348040

Thermal correction to Enthalpy= 0.348984

Thermal correction to Gibbs Free Energy= 0.277889

Sum of electronic and zero-point Energies= -1286.107377

Sum of electronic and thermal Energies= -1286.087369

Sum of electronic and thermal Enthalpies= -1286.086425

Sum of electronic and thermal Free Energies= -1286.157520

TS-3a-r-conf1

E(scf) = -825.700714105 a.u.

ν_{min} = - 691.73cm^{-1}

C	-0.218775	3.261164	-0.197115	O	-5.504354	0.339477	-0.008659
C	-1.663779	3.387888	0.119614	C	2.108962	2.864214	0.302364
C	-2.380893	2.192648	-0.002102	H	2.331374	2.968384	-0.764678
H	0.110283	3.366681	-1.246325	H	0.482146	2.897271	1.774569
H	-1.759297	1.319569	-0.247801	C	-6.004812	-0.974681	-0.185217
C	-3.733008	1.847412	0.101247	H	-5.742480	-1.382380	-1.176154
H	-4.529156	2.563573	0.291529	H	-5.609957	-1.668405	0.576535
C	0.752135	3.003103	0.721945	H	-7.097990	-0.910399	-0.091683
C	-4.134698	0.485267	-0.098433	N	3.129001	2.617200	1.078571
O	-3.423961	-0.495901	-0.323568	C	2.975531	2.455221	2.532591

H	2.532249	3.361015	2.968889	C	-1.299865	5.861110	0.268602
H	2.331183	1.591654	2.749852	C	-3.861704	6.290318	1.254903
H	3.961967	2.288210	2.977691	H	-4.124807	4.168858	1.153220
C	4.485012	2.482474	0.539832	C	-1.725355	7.154811	0.566199
H	5.135466	3.244140	0.994750	H	-0.285144	5.724647	-0.114906
H	4.877343	1.484797	0.786259	C	-3.015286	7.389244	1.060351
H	4.471930	2.613042	-0.548673	H	-4.869046	6.443123	1.654213
C	-2.144149	4.726427	0.442325	H	-1.039477	7.992642	0.408046
C	-3.443654	4.993085	0.956170	H	-3.350367	8.403036	1.292990

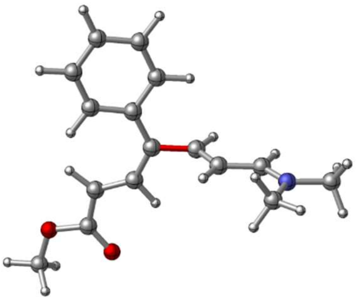

Zero-point correction= 0.315181 (Hartree/Particle)

Thermal correction to Energy= 0.334363

Thermal correction to Enthalpy= 0.335307

Thermal correction to Gibbs Free Energy= 0.265268

Sum of electronic and zero-point Energies= -825.385533

Sum of electronic and thermal Energies= -825.366352

Sum of electronic and thermal Enthalpies= -825.365407

Sum of electronic and thermal Free Energies= -825.435446

TS-3a-r-conf2

E(scf) = -825.706538428a.u.

ν_{min} = - 736.33cm^{-1}

C	-0.212193	3.156534	-0.117305	O	-2.229624	-0.929883	2.156554
C	-1.565908	3.520073	0.348265	C	2.173484	2.862275	0.089853
C	-2.295548	2.515447	0.983486	H	2.184526	2.446284	-0.922970
H	-0.100725	2.717312	-1.124626	H	0.860834	3.711365	1.629623
H	-3.319567	2.757922	1.288787	C	-3.049182	-1.891990	2.798156
C	-1.902583	1.207837	1.287428	H	-3.367293	-1.556236	3.799858
H	-0.903484	0.835474	1.050500	H	-3.960110	-2.108108	2.214366
C	0.921368	3.287785	0.625237	H	-2.447126	-2.806827	2.891351
C	-2.803418	0.307678	1.940415	N	3.326126	2.919301	0.699744
O	-3.961523	0.522926	2.302747	C	3.455147	3.452203	2.064851

H	3.141465	4.505308	2.090022	C	-1.263571	5.795289	-0.665721
H	2.833605	2.868658	2.758192	C	-3.810586	6.586253	0.119856
H	4.502602	3.381828	2.376336	H	-4.023644	4.635341	0.975358
C	4.557291	2.453998	0.055749	C	-1.723244	7.077821	-0.970539
H	5.273962	3.286475	-0.003363	H	-0.256122	5.512569	-0.982755
H	4.998894	1.644981	0.656248	C	-3.001950	7.493287	-0.582746
H	4.339137	2.085153	-0.953346	H	-4.814531	6.884951	0.436724
C	-2.063356	4.856897	0.044095	H	-1.069195	7.762004	-1.519836
C	-3.359465	5.304822	0.425470	H	-3.361940	8.497453	-0.819405

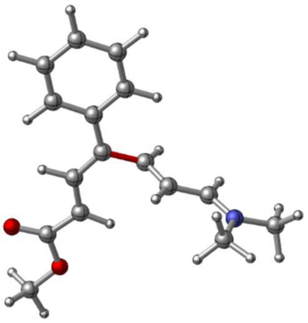

Zero-point correction= 0.314826 (Hartree/Particle)

Thermal correction to Energy= 0.334121

Thermal correction to Enthalpy= 0.335065

Thermal correction to Gibbs Free Energy= 0.264539

Sum of electronic and zero-point Energies= -825.391712

Sum of electronic and thermal Energies= -825.372418

Sum of electronic and thermal Enthalpies= -825.371474

Sum of electronic and thermal Free Energies= -825.442000

TS-3a-r-conf3

E(scf) = -825.702957391 a.u.

ν_{min} = - 685.20 cm^{-1}

C	-0.076305	3.719483	-0.369196	C	-3.643279	0.503490	-0.205740
C	-1.500912	3.654439	0.061342	O	-2.844632	-0.371015	-0.544314
C	-2.090254	2.394846	-0.103198	O	-4.976763	0.193707	-0.037381
H	0.152242	3.904039	-1.431472	C	0.801700	3.331417	1.841852
H	-1.394669	1.617941	-0.451533	H	-0.235550	3.262793	2.185632
C	-3.383325	1.889155	0.062629	H	2.015543	3.618268	0.037156
H	-4.239649	2.493880	0.352069	C	-5.338324	-1.156153	-0.276003
C	1.005279	3.562147	0.446265	H	-5.115965	-1.461357	-1.312522

H	-4.803988	-1.847285	0.397689	C	-2.100345	4.898877	0.531630
H	-6.419928	-1.228782	-0.095445	C	-3.378971	4.970241	1.151608
N	1.735278	3.192663	2.741673	C	-1.399454	6.133752	0.411119
C	3.164414	3.263343	2.401032	C	-3.910188	6.180028	1.600219
H	3.423577	2.462272	1.694444	H	-3.951527	4.060298	1.312937
H	3.397503	4.238404	1.951081	C	-1.939370	7.339741	0.855399
H	3.755198	3.139996	3.314924	H	-0.407172	6.147498	-0.047011
C	1.405610	2.954187	4.149638	C	-3.205113	7.381351	1.454570
H	1.852094	2.001625	4.471270	H	-4.894531	6.180958	2.078320
H	1.819395	3.768008	4.763442	H	-1.362243	8.261069	0.731320
H	0.317834	2.912940	4.278416	H	-3.628874	8.326079	1.803927

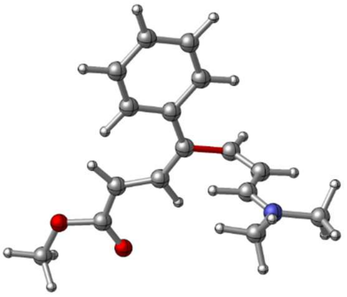

Zero-point correction= 0.315119 (Hartree/Particle)

Thermal correction to Energy= 0.334312

Thermal correction to Enthalpy= 0.335256

Thermal correction to Gibbs Free Energy= 0.265154

Sum of electronic and zero-point Energies= -825.387838

Sum of electronic and thermal Energies= -825.368645

Sum of electronic and thermal Enthalpies= -825.367701

Sum of electronic and thermal Free Energies= -825.437804

TS-3a-r-conf4

E(scf) = -825.708282959 a.u.

ν_{min} = - 497.22cm^{-1}

C	-0.308194	3.339176	-0.434531	C	0.771511	3.089006	0.357050
C	-1.700721	3.497695	0.050770	C	-2.837022	-0.147587	0.282920
C	-2.448272	2.331371	0.213778	O	-2.477814	-1.321578	0.193783
H	-0.115869	3.393325	-1.517881	O	-4.145795	0.150040	0.605433
H	-3.499096	2.451150	0.492729	C	0.568478	2.892909	1.759045
C	-2.010822	1.006793	0.086712	H	-0.464921	2.931758	2.118213
H	-0.974374	0.774790	-0.167488	H	1.765937	2.981076	-0.079498

C	-5.002168	-0.957978	0.825144	H	0.076770	2.449298	4.188442
H	-5.079215	-1.597568	-0.070145	C	-2.198929	4.850929	0.274847
H	-4.648165	-1.588818	1.657920	C	-3.511102	5.123025	0.753085
H	-5.991312	-0.546374	1.069712	C	-1.379693	5.985410	0.021114
N	1.497504	2.652921	2.643371	C	-3.960934	6.425399	0.956346
C	2.921155	2.585695	2.282961	H	-4.189082	4.296921	0.976275
H	3.517864	2.515523	3.198853	C	-1.835803	7.288169	0.229939
H	3.110502	1.698418	1.661289	H	-0.360455	5.841032	-0.347208
H	3.215967	3.488626	1.731904	C	-3.132120	7.528212	0.699271
C	1.162590	2.399022	4.046993	H	-4.978308	6.584074	1.326815
H	1.524362	1.399577	4.331353	H	-1.166277	8.127730	0.019397
H	1.653774	3.150537	4.682686	H	-3.490089	8.547598	0.862479

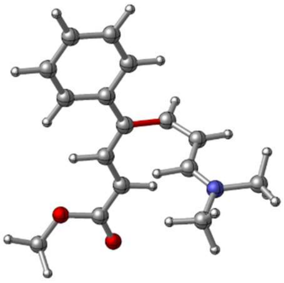

Zero-point correction= 0.314631 (Hartree/Particle)

Thermal correction to Energy= 0.334012

Thermal correction to Enthalpy= 0.334956

Thermal correction to Gibbs Free Energy= 0.263534

Sum of electronic and zero-point Energies= -825.393652

Sum of electronic and thermal Energies= -825.374271

Sum of electronic and thermal Enthalpies= -825.373327

Sum of electronic and thermal Free Energies= -825.444749

TS-3b-CN-r

E(scf) = -917.884245502 a.u.

ν_{min} = -669.7cm^{-1}

C	-1.494279	4.874621	0.938251	C	-2.828391	5.217791	0.963948
C	-1.038761	3.573569	0.391315	C	-1.597395	0.961029	3.060380
C	-1.075837	2.418041	1.135439	O	-1.199657	-0.114269	2.601466
H	-0.693005	1.506665	0.659097	O	-2.141327	1.003947	4.335451
C	-1.588561	2.242915	2.440624	C	-3.801130	4.261736	0.560343
H	-1.992994	3.085998	3.005358	H	-3.431194	3.282465	0.243411

H	-3.153946	6.169377	1.385555	H	-5.440044	2.444411	-0.023741
C	-2.190575	-0.228696	5.029845	H	-0.656832	3.579602	-0.633647
H	-2.801573	-0.977713	4.497076	C	-0.483977	5.802940	1.496959
H	-1.185495	-0.662091	5.171360	C	0.715779	5.283742	2.029106
H	-2.640583	-0.020807	6.011272	C	-0.669894	7.202750	1.496492
N	-5.099084	4.441436	0.556641	C	1.679098	6.123299	2.575222
C	-5.715588	5.714213	0.947836	H	0.868987	4.203895	2.020779
H	-6.775565	5.698035	0.670312	C	0.300377	8.054383	2.014331
H	-5.632012	5.857915	2.035847	H	-1.569315	7.637430	1.057388
H	-5.225759	6.550437	0.431321	C	1.480507	7.519416	2.565743
C	-6.011527	3.349260	0.214233	H	2.592006	5.706586	3.004157
H	-6.675881	3.146455	1.068059	H	0.151254	9.135282	1.991351
H	-6.625142	3.637852	-0.652286	C	2.480790	8.393916	3.106724

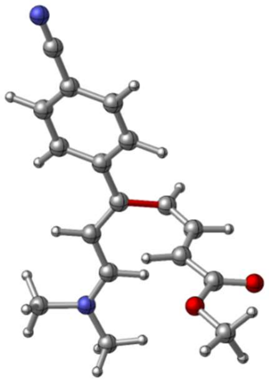

Zero-point correction= 0.312797 (Hartree/Particle)

Thermal correction to Energy= 0.334049

Thermal correction to Enthalpy= 0.334993

Thermal correction to Gibbs Free Energy= 0.259484

Sum of electronic and zero-point Energies= -917.571449

Sum of electronic and thermal Energies= -917.550196

Sum of electronic and thermal Enthalpies= -917.549252

Sum of electronic and thermal Free Energies= -917.624762

TS-3b-NMe$_2$-r

E(scf) = -959.598007767 a.u.

ν_{min} = -415.07 cm^{-1}

C	-1.404495	4.861013	0.947500	C	-1.300937	2.302805	2.632971
C	-0.984037	3.519787	0.457288	H	-1.656972	3.172682	3.190439
C	-0.941238	2.412414	1.268824	C	-2.763027	5.189881	0.980830
H	-0.607748	1.474422	0.805624	C	-1.220813	1.062114	3.316433

O	-0.855250	-0.038195	2.883566	H	-6.609950	3.114634	1.247510
O	-1.626784	1.169547	4.644719	H	-6.600475	3.575066	-0.480967
C	-3.735181	4.228743	0.662051	H	-5.395899	2.404083	0.141083
H	-3.381528	3.231377	0.386079	H	-0.712292	3.446485	-0.600460
H	-3.086433	6.178461	1.305392	C	-0.389721	5.800588	1.395466
C	-1.583364	-0.023193	5.402029	C	0.982718	5.443964	1.344426
H	-2.234742	-0.807703	4.978397	C	-0.679247	7.095884	1.904869
H	-0.562910	-0.440636	5.460577	C	1.988938	6.294086	1.762958
H	-1.930904	0.230736	6.414314	H	1.239395	4.455038	0.960616
N	-5.045623	4.408775	0.675727	C	0.310072	7.962294	2.325436
C	-5.638518	5.699164	1.025527	H	-1.712080	7.438732	1.982244
H	-5.405517	5.962482	2.069233	C	1.689560	7.592800	2.270497
H	-5.254573	6.491384	0.365393	H	3.020192	5.951385	1.696639
H	-6.726988	5.640623	0.910586	H	0.016508	8.938811	2.706815
C	-5.963190	3.312476	0.378048	N	2.670758	8.443433	2.684474

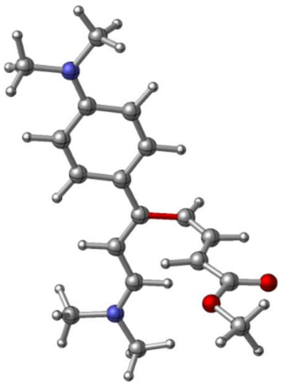

Zero-point correction= 0.387196 (Hartree/Particle)

Thermal correction to Energy= 0.410895

Thermal correction to Enthalpy= 0.411840

Thermal correction to Gibbs Free Energy= 0.331357

Sum of electronic and zero-point Energies= -959.210812

Sum of electronic and thermal Energies= -959.187112

Sum of electronic and thermal Enthalpies= -959.186168

Sum of electronic and thermal Free Energies= -959.266650

TS-3b-OH-Cl

E(scf) = -1286.43544267 a.u.

ν_{min} = -915.45cm^{-1}

C	-1.682883	4.294776	0.162714	C	-1.016430	3.118641	-0.385518

C	-1.660245	1.918433	-0.386379	H	-5.157071	2.455062	-3.261238
H	-1.150353	1.000689	-0.688467	H	-4.101953	1.374152	-2.303203
C	-3.023724	1.813281	0.110296	C	-5.205514	4.514875	-1.618312
H	-2.829201	1.811840	1.494099	H	-5.404836	4.763251	-2.668654
C	-3.024254	4.249637	0.421118	H	-6.144959	4.257868	-1.111701
C	-3.668286	0.488326	0.072600	H	-4.736091	5.371098	-1.121553
O	-3.101615	-0.563543	-0.156552	H	0.016987	3.198685	-0.726329
O	-4.979766	0.544788	0.406933	C	-0.880620	5.503241	0.487659
C	-3.829557	3.064010	-0.001479	C	-1.409851	6.796428	0.310373
H	-4.788519	2.982094	0.522311	C	0.428518	5.383381	0.993532
H	-3.525693	5.076377	0.925835	C	-0.661220	7.929773	0.636389
C	-5.659763	-0.705429	0.529111	H	-2.411785	6.913541	-0.108733
H	-5.629911	-1.264171	-0.418894	C	1.176574	6.516703	1.322577
H	-5.200077	-1.322782	1.315919	H	0.855588	4.391010	1.154172
H	-6.696835	-0.466277	0.794744	C	0.635364	7.794680	1.145243
N	-4.293440	3.343549	-1.545116	H	-1.089352	8.923910	0.484048
C	-4.860857	2.161473	-2.245703	H	2.186460	6.400246	1.724150
H	-5.728826	1.799866	-1.683806	H	1.222246	8.681093	1.398845

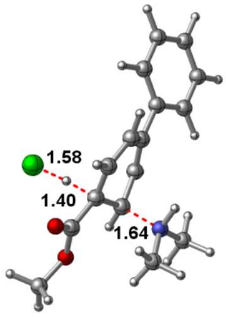

Zero-point correction= 0.327642 (Hartree/Particle)
Thermal correction to Energy= 0.347737
Thermal correction to Enthalpy= 0.348681
Thermal correction to Gibbs Free Energy= 0.277272
Sum of electronic and zero-point Energies= -1286.107801
Sum of electronic and thermal Energies= -1286.087706

Sum of electronic and thermal Enthalpies= -1286.086761
Sum of electronic and thermal Free Energies= -1286.158171

TS-3b-r-conf1
E(scf) = -825.704568836 a.u.
ν_{min} = -996.86 cm^{-1}

C	0.393082	2.657997	-0.556041	H	3.237376	4.169979	-3.230698
C	-1.075527	2.829134	-0.666143	H	3.179383	2.486837	-3.845552
C	-1.938498	1.817752	-1.017553	H	3.169821	3.860682	-4.988431
H	-1.487882	0.841931	-1.251539	C	0.678157	3.893596	-5.230248
C	-3.344957	1.844353	-1.134517	H	0.987135	4.897802	-5.558291
H	-3.907956	2.757149	-0.921063	H	0.954305	3.166480	-6.009078
C	1.222700	2.907041	-1.638629	H	-0.408680	3.875248	-5.086543
C	-4.065382	0.690155	-1.540426	H	-1.455248	3.825869	-0.406384
O	-3.627449	-0.428965	-1.836229	C	0.976627	2.236112	0.738800
O	-5.436492	0.920284	-1.589560	C	0.133516	1.696468	1.735585
C	0.675499	3.281116	-2.888285	C	2.356170	2.357400	1.033484
H	-0.415081	3.338943	-2.955891	C	0.647909	1.269593	2.960164
H	2.301888	2.784942	-1.552690	H	-0.932840	1.609686	1.525291
C	-6.234271	-0.178326	-1.984144	C	2.865392	1.947426	2.263105
H	-5.987488	-0.525029	-3.003031	H	3.040635	2.799037	0.307835
H	-6.116275	-1.041134	-1.305635	C	2.014410	1.395257	3.230394
H	-7.279253	0.163926	-1.959986	H	-0.022434	0.842816	3.710050
N	1.355451	3.553947	-3.978404	H	3.931492	2.061991	2.473284
C	2.821484	3.514394	-4.009009	H	2.417023	1.071827	4.193580

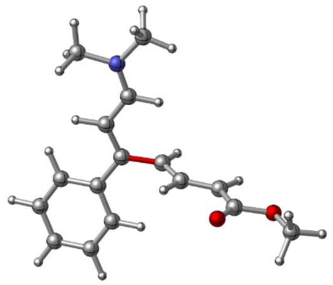

Zero-point correction= 0.314559 (Hartree/Particle)

Thermal correction to Energy= 0.333814

Thermal correction to Enthalpy= 0.334758

Thermal correction to Gibbs Free Energy= 0.264384

Sum of electronic and zero-point Energies= -825.390010

Sum of electronic and thermal Energies= -825.370755

Sum of electronic and thermal Enthalpies= -825.369811

Sum of electronic and thermal Free Energies= -825.440185

TS-3b-r-conf2

E(scf) = -825.706538428 a.u.

ν_{min} = - 736.33 cm^{-1}

C	-0.212193	3.156534	-0.117305	C	3.455147	3.452203	2.064851
C	-1.565908	3.520073	0.348265	H	3.141465	4.505308	2.090022
C	-2.295548	2.515447	0.983486	H	2.833605	2.868658	2.758192
H	-0.100725	2.717312	-1.124626	H	4.502602	3.381828	2.376336
H	-3.319567	2.757922	1.288787	C	4.557291	2.453998	0.055749
C	-1.902583	1.207837	1.287428	H	5.273962	3.286475	-0.003363
H	-0.903484	0.835474	1.050500	H	4.998894	1.644981	0.656248
C	0.921368	3.287785	0.625237	H	4.339137	2.085153	-0.953346
C	-2.803418	0.307678	1.940415	C	-2.063356	4.856897	0.044095
O	-3.961523	0.522926	2.302747	C	-3.359465	5.304822	0.425470
O	-2.229624	-0.929883	2.156554	C	-1.263571	5.795289	-0.665721
C	2.173484	2.862275	0.089853	C	-3.810586	6.586253	0.119856
H	2.184526	2.446284	-0.922970	H	-4.023644	4.635341	0.975358
H	0.860834	3.711365	1.629623	C	-1.723244	7.077821	-0.970539
C	-3.049182	-1.891990	2.798156	H	-0.256122	5.512569	-0.982755
H	-3.367293	-1.556236	3.799858	C	-3.001950	7.493287	-0.582746
H	-3.960110	-2.108108	2.214366	H	-4.814531	6.884951	0.436724
H	-2.447126	-2.806827	2.891351	H	-1.069195	7.762004	-1.519836
N	3.326126	2.919301	0.699744	H	-3.361940	8.497453	-0.819405

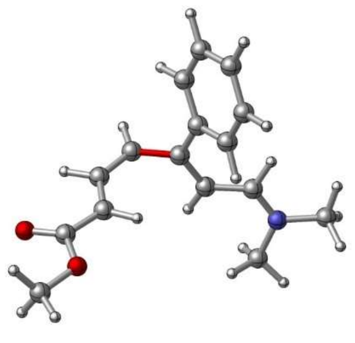

Zero-point correction= 0.314826 (Hartree/Particle)

Thermal correction to Energy= 0.334121

Thermal correction to Enthalpy= 0.335065

Thermal correction to Gibbs Free Energy= 0.264539

Sum of electronic and zero-point Energies= -825.391712

Sum of electronic and thermal Energies= -825.372418

Sum of electronic and thermal Enthalpies= -825.371474

Sum of electronic and thermal Free Energies= -825.442000

TS-3b-r-conf3

E(scf) = -825.696465517 a.u.

ν_{min} = - 1251.88cm^{-1}

C	0.337279	2.999965	0.070093	H	1.080722	2.778858	-3.905328
C	-1.104091	3.149053	0.375464	H	1.168032	4.569299	-3.952486
C	-2.016556	2.140739	0.178494	H	2.376839	3.587779	-4.831698
H	-1.628184	1.193702	-0.224475	C	4.056649	3.888756	-2.989395
C	-3.409541	2.136318	0.411694	H	4.463889	3.119666	-3.662597
H	-3.914159	3.020136	0.811762	H	4.175938	4.874513	-3.464289
C	0.798910	3.307081	-1.196309	H	4.607005	3.874191	-2.041148
C	-4.191962	0.987590	0.127396	H	-1.411234	4.118909	0.789237
O	-3.826453	-0.104384	-0.327807	C	1.235389	2.510338	1.140004
O	-5.538618	1.184941	0.422189	C	1.052104	2.960358	2.466268
C	2.170569	3.436387	-1.539468	C	2.241909	1.552589	0.888215
H	2.920171	3.425879	-0.743673	C	1.886511	2.515238	3.488499
H	0.044956	3.549499	-1.946969	H	0.253302	3.672910	2.679278
C	-6.392396	0.086730	0.171751	C	3.057225	1.084604	1.922706
H	-6.376026	-0.218127	-0.889318	H	2.348904	1.131426	-0.113599
H	-6.116619	-0.798412	0.771636	C	2.891119	1.573412	3.221125
H	-7.409176	0.406787	0.443183	H	1.748023	2.892801	4.504473
N	2.636064	3.630073	-2.751001	H	3.816109	0.326902	1.713283
C	1.759788	3.641452	-3.927717	H	3.529922	1.209883	4.029781

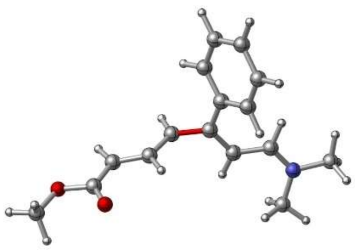

Zero-point correction= 0.314281 (Hartree/Particle)

Thermal correction to Energy= 0.333610

Thermal correction to Enthalpy= 0.334554

Thermal correction to Gibbs Free Energy= 0.264018

Sum of electronic and zero-point Energies= -825.382185

Sum of electronic and thermal Energies= -825.362855

Sum of electronic and thermal Enthalpies= -825.361911

Sum of electronic and thermal Free Energies= -825.432448

TS-3b-r-conf4
E(scf) = -825.706768383 a.u.
ν_{min} = -596.15 cm^{-1}

C	-1.380630	4.882008	0.933473	H	-5.197073	6.496009	0.593556
C	-0.950422	3.581567	0.360638	H	-6.691377	5.610085	1.005907
C	-0.884295	2.429183	1.108258	H	-5.420163	5.829978	2.243304
H	-0.535131	1.530191	0.587498	C	-5.914384	3.292670	0.441164
C	-1.252805	2.258102	2.463635	H	-6.527487	3.066022	1.326932
H	-1.622976	3.107828	3.043742	H	-6.580557	3.579472	-0.386637
C	-2.723216	5.200684	1.026512	H	-5.343622	2.400020	0.158828
C	-1.194763	1.032034	3.181162	H	-0.693827	3.579098	-0.703003
O	-1.515388	0.844376	4.359372	C	-0.354379	5.824515	1.429244
O	-0.724396	-0.037922	2.427050	C	0.939384	5.344604	1.729820
C	-3.699099	4.233920	0.679819	C	-0.611551	7.203391	1.608361
H	-3.334331	3.259072	0.343517	C	1.924662	6.199768	2.221548
H	-3.044826	6.150194	1.454295	H	1.147486	4.283990	1.582286
C	-0.657454	-1.283794	3.093106	C	0.382533	8.061468	2.076244
H	0.010640	-1.247361	3.970833	H	-1.587364	7.619934	1.352516
H	-1.648624	-1.617574	3.446253	C	1.652430	7.562469	2.392649
H	-0.265841	-2.009941	2.365966	H	2.913424	5.803967	2.465911
N	-5.001062	4.394218	0.745079	H	0.168209	9.126722	2.191628
C	-5.609389	5.658062	1.172669	H	2.429119	8.235990	2.763491

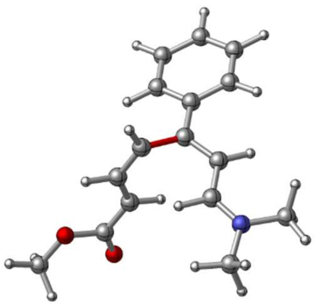

Zero-point correction= 0.314328 (Hartree/Particle)
Thermal correction to Energy= 0.333698
Thermal correction to Enthalpy= 0.334642
Thermal correction to Gibbs Free Energy= 0.263809
Sum of electronic and zero-point Energies= -825.392440
Sum of electronic and thermal Energies= -825.373071
Sum of electronic and thermal Enthalpies= -825.372127
Sum of electronic and thermal Free Energies= -825.442959

3a-OH
E(scf) = -1286.45284976 a.u.
ν_{min} = 15.90 cm^{-1}

C	-0.143938	2.242736	0.406707	C	3.308691	4.522460	1.639671
C	-1.494442	2.600841	0.391816	H	2.864487	5.199459	0.891874
C	-2.419022	1.612506	-0.032599	H	2.705834	4.579600	2.560993
H	0.082032	1.223241	0.069500	H	4.325636	4.865954	1.863554
H	-1.982564	0.642556	-0.294295	C	4.662873	2.511692	1.056053
C	-3.789079	1.744556	-0.161554	H	5.334775	3.079902	0.391981
H	-4.299142	2.679460	0.073453	H	5.128172	2.454870	2.053969
C	0.948858	3.030563	0.816861	H	4.555680	1.493709	0.660020
C	-4.633313	0.693766	-0.606576	C	-1.948072	3.963638	0.800193
O	-4.320899	-0.498049	-0.947952	C	-2.809328	4.136486	1.899095
O	-5.919929	1.004922	-0.670555	C	-1.527465	5.105641	0.094478
C	2.240162	2.520977	0.772515	C	-3.235868	5.411647	2.281867
H	2.387969	1.499326	0.407209	H	-3.142959	3.259384	2.459098
H	0.767689	4.045059	1.174556	C	-1.951590	6.381761	0.476986
C	-6.852395	0.008700	-1.113137	H	-0.863380	4.986772	-0.765306
H	-6.614468	-0.318146	-2.135929	C	-2.807972	6.539141	1.572246
H	-6.834842	-0.863680	-0.443728	H	-3.902977	5.525226	3.140402
H	-7.836165	0.491089	-1.085679	H	-1.615300	7.256497	-0.085894
N	3.358835	3.155816	1.135666	H	-3.141094	7.536291	1.871194

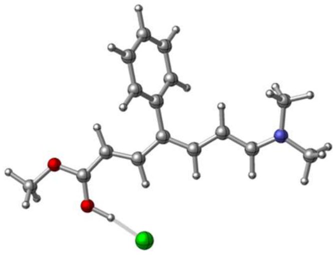

Zero-point correction= 0.326410 (Hartree/Particle)

Thermal correction to Energy= 0.348189

Thermal correction to Enthalpy= 0.349133

Thermal correction to Gibbs Free Energy= 0.271823

Sum of electronic and zero-point Energies= -1286.126440

Sum of electronic and thermal Energies= -1286.104661

Sum of electronic and thermal Enthalpies= -1286.103717

Sum of electronic and thermal Free Energies= -1286.181027

3a-OH-aii

E(scf) = -1286.43850809 a.u.
ν_{min} = 14.97 cm^{-1}

C	-0.161400	4.153602	0.523372	N	2.110959	1.762012	2.310653
C	-1.530733	3.972769	0.703432	C	3.380857	1.881090	1.611805
C	-2.210624	2.730197	1.014316	H	4.081324	1.128480	1.994585
H	0.099994	5.085922	0.007947	H	3.245486	1.717188	0.530736
H	-3.256799	2.847744	1.312820	H	3.825654	2.881394	1.756035
C	-1.787720	1.437116	0.902868	C	2.071780	0.929523	3.503640
H	-0.776913	1.172483	0.594579	H	2.374150	-0.103328	3.265018
C	0.989281	3.392289	0.889719	H	2.754081	1.318329	4.279297
C	-2.673068	0.320000	1.181034	H	1.053360	0.909637	3.913019
O	-3.863847	0.366575	1.507624	C	-2.413780	5.154085	0.467627
O	-2.047485	-0.861930	1.057174	C	-3.689565	5.016357	-0.121268
C	1.041359	2.475050	1.921088	C	-1.999713	6.456504	0.822743
H	0.142999	2.299454	2.517249	C	-4.506384	6.126368	-0.348489
H	1.917362	3.664795	0.381612	H	-4.054125	4.029436	-0.411316
C	-2.827300	-2.038365	1.286677	C	-2.810952	7.568633	0.583368
H	-3.215363	-2.056145	2.316534	H	-1.036926	6.596661	1.320133
H	-3.678456	-2.086008	0.590397	C	-4.071238	7.410775	-0.003439
H	-2.153522	-2.887661	1.120954	H	-5.489412	5.984005	-0.805565

H	-2.462302	8.563394	0.874053	H	-4.710592	8.278866	-0.183101

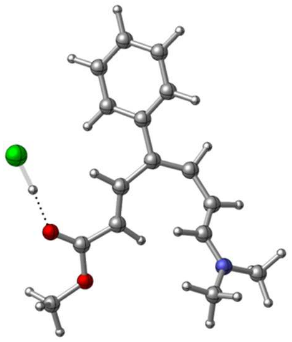

Zero-point correction= 0.325664 (Hartree/Particle)

Thermal correction to Energy= 0.347798

Thermal correction to Enthalpy= 0.348743

Thermal correction to Gibbs Free Energy= 0.270368

Sum of electronic and zero-point Energies= -1286.112844

Sum of electronic and thermal Energies= -1286.090710

Sum of electronic and thermal Enthalpies= -1286.089765

Sum of electronic and thermal Free Energies= -1286.168140

3a-OH-int

E(scf) = -1286.44398626 a.u.

ν_{min} = 15.89 cm^{-1}

C	-0.193651	2.033997	0.572156	H	-4.688564	-1.873835	-3.120105
C	-1.396229	2.577373	0.140360	H	-4.068700	-3.315461	-2.234776
C	-2.407442	1.752232	-0.471608	N	1.845592	4.234701	2.831948
H	-0.026673	0.992182	0.277033	C	3.134917	3.564710	2.903093
H	-3.286009	2.297318	-0.828263	H	3.045066	2.576874	3.387250
C	-2.437336	0.395975	-0.656694	H	3.554472	3.416551	1.894688
H	-1.621714	-0.262043	-0.351782	H	3.833941	4.177108	3.485801
C	0.878193	2.564612	1.348220	C	1.657483	5.428543	3.643026
C	-3.570199	-0.246978	-1.287342	H	1.754493	5.195295	4.717278
O	-4.655050	0.275832	-1.581664	H	2.409257	6.193202	3.387051
O	-3.352676	-1.547000	-1.543455	H	0.658880	5.848324	3.464669
C	0.844605	3.732386	2.091821	C	-1.729477	4.029910	0.264647
H	-0.072699	4.322218	2.119860	C	-2.905176	4.450403	0.911872
H	1.773368	1.941111	1.397647	C	-0.893179	5.011745	-0.302575
C	-4.426944	-2.283885	-2.132703	C	-3.222533	5.810192	1.011218
H	-5.321610	-2.252966	-1.492228	H	-3.583519	3.703326	1.330583

C	-1.207008	6.367828	-0.202249	H	-4.140749	6.114745	1.520510
H	0.018913	4.699143	-0.817031	H	-0.543959	7.112945	-0.650078
C	-2.372646	6.774235	0.461414	H	-2.619232	7.836170	0.540264

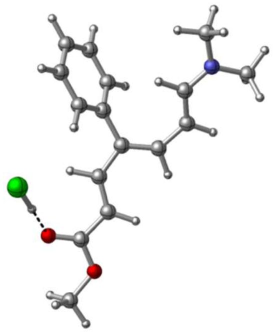

Zero-point correction= 0.325593 (Hartree/Particle)

Thermal correction to Energy= 0.347742

Thermal correction to Enthalpy= 0.348686

Thermal correction to Gibbs Free Energy= 0.270400

Sum of electronic and zero-point Energies= -1286.118393

Sum of electronic and thermal Energies= -1286.096244

Sum of electronic and thermal Enthalpies= -1286.095300

Sum of electronic and thermal Free Energies= -1286.173586

3a-OH-p1

E(scf) = -1286.44788814a.u.

ν_{min} = 31.09 cm^{-1}

C	-1.414723	4.544623	0.920456	H	-5.343047	-1.082934	0.300550
C	-1.123515	3.402600	1.807424	H	-6.511466	0.241537	0.601250
C	-1.914268	2.306102	1.728245	H	-6.132126	-0.248620	-1.091735
H	-1.790733	1.475006	2.422677	N	-4.710370	4.071159	1.239919
C	-2.971750	2.212244	0.670262	C	-4.379939	4.220369	2.646048
H	-2.479655	1.951828	-0.286393	H	-4.286993	3.253637	3.181825
C	-2.572276	4.644312	0.240126	H	-3.429954	4.759348	2.765359
C	-3.994697	1.131904	0.917300	H	-5.166766	4.807108	3.147799
O	-4.227048	0.585639	1.984141	C	-6.048124	3.550423	1.018641
O	-4.671008	0.836803	-0.188754	H	-6.225907	2.558031	1.485446
C	-3.663750	3.599577	0.352603	H	-6.793280	4.240705	1.448919
H	-4.130430	3.476135	-0.635314	H	-6.251810	3.459978	-0.058601
H	-2.802198	5.534915	-0.350772	H	-0.673732	5.345256	0.849557
C	-5.729804	-0.126790	-0.079805	C	-0.025746	3.508629	2.801910

C	0.714512	2.375194	3.194350	H	-0.272280	5.639767	3.126233
C	0.291989	4.744924	3.398790	C	2.021654	3.705795	4.744187
C	1.722766	2.471506	4.155354	H	2.286037	1.578663	4.439132
H	0.509338	1.410917	2.724030	H	1.523036	5.810702	4.815654
C	1.301633	4.841813	4.360501	H	2.813705	3.781803	5.493393

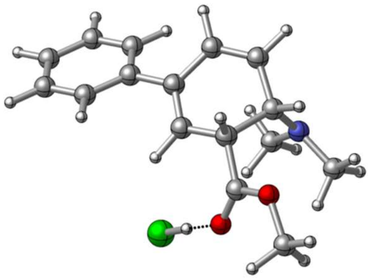

Zero-point correction= 0.328495 (Hartree/Particle)

Thermal correction to Energy= 0.348947

Thermal correction to Enthalpy= 0.349891

Thermal correction to Gibbs Free Energy= 0.278219

Sum of electronic and zero-point Energies= -1286.119393

Sum of electronic and thermal Energies= -1286.098941

Sum of electronic and thermal Enthalpies= -1286.097997

Sum of electronic and thermal Free Energies= -1286.169669

3b-OH

E(scf) = -1286.45285742 a.u.

ν_{min} = 16.79 cm^{-1}

C	-0.043420	1.823157	0.275282	C	-5.451282	-1.337960	-1.492057
C	-1.392970	2.145945	0.285908	H	-6.152053	-1.584759	-0.680049
C	-2.448312	1.296747	-0.143282	H	-6.019204	-0.896051	-2.324863
H	-2.183753	0.309058	-0.531538	H	-4.927515	-2.241058	-1.827935
C	-3.778796	1.627508	-0.113270	N	3.247730	3.392059	1.143625
H	-4.113071	2.595438	0.266118	C	2.924441	4.779561	1.433385
C	0.937890	2.792727	0.662935	H	2.423466	5.254910	0.573437
C	-4.835782	0.751223	-0.566383	H	2.255086	4.860228	2.307472
O	-6.044101	1.036546	-0.543270	H	3.846531	5.333526	1.648650
O	-4.437149	-0.440168	-1.034205	C	4.631432	2.971638	1.298055
C	2.285199	2.520329	0.790197	H	5.295166	3.562545	0.644713
H	2.643146	1.506190	0.594972	H	4.970418	3.101784	2.340298
H	0.566294	3.793806	0.893182	H	4.733524	1.911806	1.029934

H	-6.450156	2.422086	0.003588
C			
l	-6.762470	3.660422	0.496131
H	-1.669802	3.147488	0.634256
C	0.402426	0.457016	-0.140708
C	1.160472	0.276928	-1.311394
C	0.072366	-0.670801	0.630536
C	1.570553	-1.000012	-1.704923
H	1.421548	1.147157	-1.918356
C	0.488583	-1.947377	0.240626
H	-0.513339	-0.540175	1.543509
C	1.237102	-2.115917	-0.929040
H	2.151559	-1.123941	-2.622389

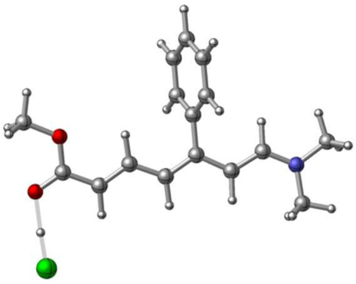

Zero-point correction= 0.325404 (Hartree/Particle)

Thermal correction to Energy= 0.347567

Thermal correction to Enthalpy= 0.348511

Thermal correction to Gibbs Free Energy= 0.269734

Sum of electronic and zero-point Energies= -1286.127453

Sum of electronic and thermal Energies= -1286.105290

Sum of electronic and thermal Enthalpies= -1286.104346

Sum of electronic and thermal Free Energies= -1286.183123

3b-OH-aii

E(scf) = -1286.44319050 a.u.

ν_{min} = 29.09 cm^{-1}

C	-0.827698	4.921687	1.445837
C	-0.173604	3.672910	1.294216
C	-0.566874	2.421346	1.793076
H	-0.077190	1.560543	1.326249
C	-1.411243	2.128594	2.873408
H	-1.787845	2.916929	3.526634
C	-2.228524	5.038975	1.621708
C	-1.821334	0.825508	3.154186
O	-1.725516	-0.184614	2.341113
O	-2.375522	0.591972	4.339519
C	-3.096946	4.019426	1.222969
H	-2.706427	3.190125	0.622161
H	-2.630581	5.970978	2.022271
C	-2.950412	-0.696715	4.589158
H	-3.705901	-0.943243	3.828275
H	-2.175518	-1.477541	4.590507
H	-3.416570	-0.628371	5.579223
N	-4.392072	3.935510	1.499688
C	-5.058396	4.933455	2.328932
H	-4.482745	5.111233	3.249527
H	-5.161711	5.889615	1.788954
H	-6.058356	4.573249	2.599440
C	-5.216141	2.884633	0.905089
H	-5.801740	2.385232	1.692356
H	-5.917683	3.315105	0.170869
H	-4.570399	2.143285	0.413315
H	0.755556	3.679506	0.716144
C	-0.015729	6.162006	1.362012

C	1.318901	6.177540	1.817125	H	-1.575533	7.355804	0.444803
C	-0.553487	7.352144	0.829556	C	1.538020	8.513740	1.213870
C	2.086774	7.341075	1.745593	H	3.116172	7.335081	2.112408
H	1.745954	5.271983	2.253450	H	-0.215913	9.423815	0.328974
C	0.215531	8.514547	0.754896	H	2.139479	9.424457	1.156421

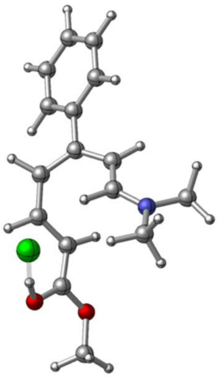

Zero-point correction= 0.328361 (Hartree/Particle)

Thermal correction to Energy= 0.349587

Thermal correction to Enthalpy= 0.350532

Thermal correction to Gibbs Free Energy= 0.276487

Sum of electronic and zero-point Energies= -1286.114830

Sum of electronic and thermal Energies= -1286.093603

Sum of electronic and thermal Enthalpies= -1286.092659

Sum of electronic and thermal Free Energies= -1286.166703

3b-OH-int

E(scf) = -1286.43931515 a.u.

ν_{min} = 12.76 cm^{-1}

C	-2.216660	4.271121	1.244500	H	-2.965816	6.113473	0.426335
C	-2.251703	2.872135	1.300643	C	1.276414	0.668334	5.845691
C	-1.532383	1.977136	2.131183	H	0.561701	-0.043376	6.284511
H	-1.700352	0.922282	1.889194	H	2.021455	0.111181	5.258901
C	-0.700574	2.224301	3.209929	H	1.768684	1.248524	6.634807
H	-0.499247	3.231204	3.570762	N	-5.373818	5.268295	-0.498530
C	-3.204959	5.060330	0.580519	C	-5.135754	6.642152	-0.920057
C	-0.076747	1.194479	3.964399	H	-5.077405	7.320272	-0.051504
O	-0.085495	-0.064408	3.768485	H	-4.191780	6.715659	-1.482236
O	0.597816	1.627133	5.024443	H	-5.957023	6.972080	-1.567883
C	-4.442508	4.583097	0.174627	C	-6.695868	4.709287	-0.747352
H	-4.729129	3.558032	0.425785	H	-7.469487	5.284433	-0.211086

H	-6.927667	4.736945	-1.824187	H	0.432463	3.968983	0.741604
H	-6.733907	3.667285	-0.404688	C	-0.267730	6.805769	3.306383
H	-2.962137	2.376628	0.633700	H	-2.356784	6.300398	3.056820
C	-1.086813	5.042974	1.839800	C	1.047541	6.522643	2.924213
C	0.239419	4.770424	1.458072	H	2.321981	5.288107	1.682213
C	-1.329233	6.078615	2.758584	H	-0.469767	7.597169	4.032578
C	1.297127	5.507306	1.992154	H	1.877166	7.094010	3.348129

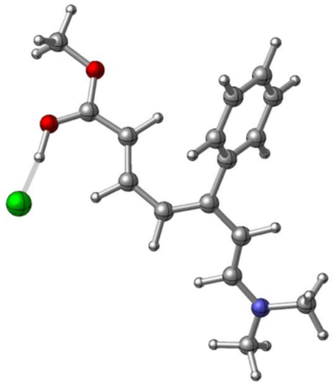

Zero-point correction= 0.325259 (Hartree/Particle)

Thermal correction to Energy= 0.347171

Thermal correction to Enthalpy= 0.348115

Thermal correction to Gibbs Free Energy= 0.270258

Sum of electronic and zero-point Energies= -1286.114056

Sum of electronic and thermal Energies= -1286.092144

Sum of electronic and thermal Enthalpies= -1286.091200

Sum of electronic and thermal Free Energies= -1286.169057

3b-OH-p1

E(scf) = -1286.44664323 a.u.

ν_{min} = 30.13 cm^{-1}

C	-1.659998	4.335996	0.261976	O	-4.982844	0.521511	0.873653
C	-1.053188	3.063834	-0.172852	C	-3.925813	3.228877	0.173240
C	-1.747422	1.912521	-0.165593	H	-4.724235	3.240232	0.929042
H	-1.314820	0.990482	-0.556799	H	-3.488844	5.339251	0.711391
C	-3.126963	1.888007	0.424779	C	-5.892436	-0.553209	0.592427
H	-3.022233	1.855467	1.525773	H	-6.366615	-0.403993	-0.389110
C	-3.002168	4.398528	0.443195	H	-5.362762	-1.516503	0.593071
C	-3.947258	0.678540	0.054565	H	-6.645637	-0.526767	1.387856
O	-3.742275	-0.077431	-0.881857	N	-4.584315	3.411558	-1.108101

C	-3.761386	3.336267	-2.301698	C	-1.221687	6.818749	0.105894
H	-3.444268	2.303632	-2.553971	C	0.519170	5.379365	0.962528
H	-4.324757	3.733121	-3.162098	C	-0.396864	7.929681	0.295690
H	-2.853495	3.943755	-2.183509	H	-2.213820	6.952851	-0.331130
C	-5.892647	2.803385	-1.273593	C	1.344539	6.490871	1.154636
H	-5.855880	1.729191	-1.555149	H	0.884290	4.387032	1.238156
H	-6.476719	2.890452	-0.345465	C	0.890202	7.771446	0.822724
H	-6.446832	3.321614	-2.074422	H	-0.757305	8.924201	0.020054
H	-0.030174	3.083682	-0.556064	H	2.347314	6.354995	1.567999
C	-0.781994	5.523546	0.442864	H	1.536846	8.640514	0.967764

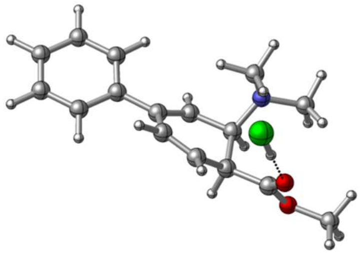

Zero-point correction= 0.328410 (Hartree/Particle)
Thermal correction to Energy= 0.348892
Thermal correction to Enthalpy= 0.349836
Thermal correction to Gibbs Free Energy= 0.278044
Sum of electronic and zero-point Energies= -1286.118234
Sum of electronic and thermal Energies= -1286.097751
Sum of electronic and thermal Enthalpies= -1286.096807
Sum of electronic and thermal Free Energies= -1286.168599

TS-3a-OH-r

E(scf) = -1286.41596930 a.u.

ν_{min} = -127.07 cm^{-1}

C	-0.347149	3.054713	-0.412744	H	-3.599301	-2.807476	1.201933
C	-1.715600	3.236899	0.133040	H	-4.055910	-2.648894	-0.520079
C	-2.488720	2.118916	0.332977	H	-2.562222	-3.544985	-0.069204
H	-0.236763	2.977835	-1.503954	N	1.689090	2.988959	2.609753
H	-3.498130	2.270040	0.726463	C	3.107823	2.909681	2.251161
C	-2.100834	0.768936	0.093997	H	3.504462	1.934848	2.571836
H	-1.094431	0.528688	-0.254239	H	3.256534	3.031440	1.175106
C	0.792874	2.957164	0.322844	H	3.648810	3.704884	2.783130
C	-2.925799	-0.325220	0.283679	C	1.425602	3.021375	4.052862
O	-4.177191	-0.360228	0.662092	H	1.865330	2.127273	4.518637
O	-2.381143	-1.534461	0.067388	H	1.895726	3.916077	4.486681
C	0.708558	3.015949	1.751322	H	0.346098	3.041783	4.240171
H	-0.298944	3.083357	2.175477	C	-2.187099	4.616724	0.377408
H	1.753743	2.825318	-0.175595	C	-3.560994	4.904097	0.581476
C	-3.206872	-2.692503	0.179697	C	-1.288168	5.709052	0.407321

C	-3.992657	6.205564	0.830606	C	-3.084981	7.274073	0.869823
H	-4.304176	4.103597	0.536186	H	-5.059283	6.390037	0.987739
C	-1.730372	7.013445	0.650282	H	-1.003838	7.830727	0.667701
H	-0.221696	5.543202	0.243834	H	-3.432510	8.292487	1.060598

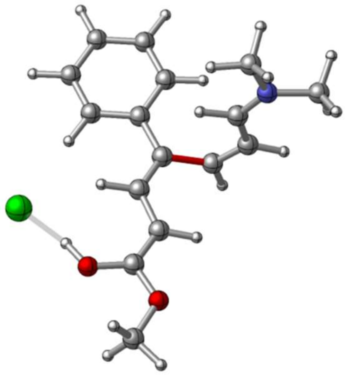

Zero-point correction= 0.327698 (Hartree/Particle)

Thermal correction to Energy= 0.348937

Thermal correction to Enthalpy= 0.349881

Thermal correction to Gibbs Free Energy= 0.274255

Sum of electronic and zero-point Energies= -1286.088272

Sum of electronic and thermal Energies= -1286.067032

Sum of electronic and thermal Enthalpies= -1286.066088

Sum of electronic and thermal Free Energies= -1286.141714

TS-3a-OH-uu

E(scf) = -1286.42107884 a.u.

ν_{min} = -278.11 cm^{-1}

C	-1.407851	4.304596	0.825019	H	-6.400790	0.075452	0.155430
C	-1.140352	3.216241	1.777606	H	-5.842509	-0.455813	-1.474377
C	-1.932800	2.104397	1.763304	N	-4.650645	4.102066	1.564220
H	-1.883995	1.395179	2.592888	C	-4.178782	4.761263	2.780991
C	-2.888188	1.874351	0.692405	H	-4.072169	4.009838	3.580130
H	-2.528546	2.036049	-0.327875	H	-3.216251	5.250774	2.602532
C	-2.616883	4.568881	0.285579	H	-4.917588	5.518517	3.086034
C	-3.912615	0.905018	0.755093	C	-5.968886	3.488028	1.689749
O	-4.441069	0.552660	-0.411802	H	-5.940975	2.733852	2.493210
C	-3.857052	3.779872	0.539210	H	-6.716019	4.254610	1.947638
H	-4.388034	3.416363	-0.344745	H	-6.258730	3.011535	0.744393
H	-2.760153	5.458929	-0.336868	H	-0.589231	4.993676	0.597246
C	-5.562538	-0.342972	-0.420758	C	-0.080862	3.407652	2.795434
H	-5.283357	-1.316985	0.006274	C	0.611718	2.307769	3.344856

C	0.255506	4.695317	3.263927	C	1.900816	3.770984	4.787996
C	1.583176	2.486259	4.331424	H	2.105891	1.616225	4.737618
H	0.398953	1.301674	2.976611	H	1.467717	5.882989	4.596522
C	1.232759	4.874284	4.246580	H	2.666090	3.910668	5.555591
H	-0.270878	5.569430	2.873179	O	-4.424569	0.367114	1.801174

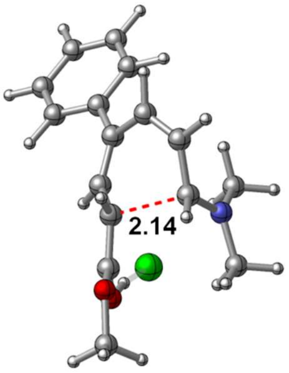

Zero-point correction= 0.327169 (Hartree/Particle)

Thermal correction to Energy= 0.347299

Thermal correction to Enthalpy= 0.348243

Thermal correction to Gibbs Free Energy= 0.277666

Sum of electronic and zero-point Energies= -1286.093910

Sum of electronic and thermal Energies= -1286.073780

Sum of electronic and thermal Enthalpies= -1286.072836

Sum of electronic and thermal Free Energies= -1286.143413

TS-3b-OH-dd

E(scf) = -1286.41917396 a.u.

ν_{min} = -282.80 cm^{-1}

C	-1.752619	4.273347	0.078809	C	-5.738720	-0.566431	1.391029
C	-1.152643	3.008123	-0.370749	H	-6.367915	-0.450739	0.496156
C	-1.782687	1.815148	-0.258512	H	-5.267908	-1.560001	1.372986
H	-1.374370	0.936069	-0.763593	H	-6.342144	-0.448706	2.298470
C	-3.018957	1.694485	0.507376	N	-4.535126	3.289232	-1.482198
H	-3.026503	2.174554	1.490233	C	-3.743862	3.693159	-2.642683
C	-3.102683	4.425459	0.146097	H	-3.303579	2.797314	-3.109405
C	-3.885292	0.586650	0.442235	H	-4.404851	4.194932	-3.366549
O	-3.984057	-0.285634	-0.496527	H	-2.950582	4.384792	-2.341473
O	-4.743328	0.464926	1.451182	C	-5.678804	2.440505	-1.801578
C	-4.112356	3.394147	-0.219620	H	-5.320986	1.527755	-2.305319
H	-4.883722	3.190940	0.528032	H	-6.218507	2.169712	-0.884898
H	-3.537962	5.385052	0.442722	H	-6.365184	2.974830	-2.476829

H	-0.183480	3.066683	-0.872476	C	1.266511	6.219631	1.314167
C	-0.842211	5.409560	0.389993	H	0.697273	4.139827	1.230679
C	-1.191568	6.741792	0.095451	C	0.901498	7.538605	1.024743
C	0.406181	5.166028	0.994198	H	-0.617560	8.820532	0.166527
C	-0.330171	7.794693	0.410962	H	2.226428	6.008614	1.792337
H	-2.137538	6.953814	-0.407984	H	1.576274	8.362875	1.269020

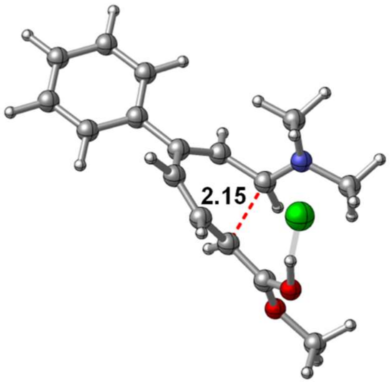

Zero-point correction= 0.327239 (Hartree/Particle)

Thermal correction to Energy= 0.347407

Thermal correction to Enthalpy= 0.348352

Thermal correction to Gibbs Free Energy= 0.277637

Sum of electronic and zero-point Energies= -1286.091935

Sum of electronic and thermal Energies= -1286.071767

Sum of electronic and thermal Enthalpies= -1286.070822

Sum of electronic and thermal Free Energies= -1286.141537

TS-3b-OH-r

E(scf) = -1286.42747508 a.u.

ν_{min} = -44.10cm^{-1}

C	-1.240707	4.832446	1.005103	H	-3.124387	2.966760	0.568642
C	-0.717817	3.534045	0.506742	H	-3.005069	6.026253	1.254998
C	-0.590299	2.435978	1.290215	C	-1.585836	0.048852	5.384748
H	-0.370594	1.491362	0.784523	H	-2.617558	-0.164380	5.062351
C	-0.801511	2.374530	2.712043	H	-0.970628	-0.846951	5.208565
H	-0.779967	3.284079	3.315849	H	-1.575398	0.298950	6.453497
C	-2.603376	5.047881	0.991208	N	-4.815472	4.111351	0.653879
C	-1.055821	1.196777	3.365152	C	-5.490120	5.404500	0.802662
O	-1.303154	0.030362	2.794341	H	-5.444540	5.741334	1.849831
O	-1.054018	1.188093	4.710595	H	-5.020346	6.159841	0.158418
C	-3.510954	3.985773	0.712439	H	-6.540459	5.293752	0.510401

C	-5.675282	2.936178	0.479976	C	2.011267	6.629073	1.704012
H	-6.395623	2.893916	1.311544	H	1.424795	4.800283	0.715944
H	-6.234403	3.028471	-0.464011	C	0.209498	7.938076	2.657360
H	-5.061878	2.023749	0.473178	H	-1.770377	7.146975	2.444329
H	-0.594891	3.444663	-0.577158	C	1.576621	7.759840	2.402981
C	-0.295990	5.851636	1.494933	H	3.075502	6.481894	1.505728
C	1.086461	5.684325	1.258938	H	-0.133648	8.812338	3.215535
C	-0.715224	6.997928	2.210602	H	2.299749	8.499338	2.755748

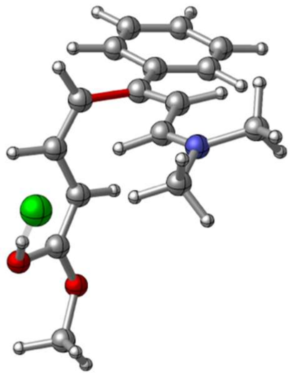

Zero-point correction= 0.328407 (Hartree/Particle)
Thermal correction to Energy= 0.349179
Thermal correction to Enthalpy= 0.350123
Thermal correction to Gibbs Free Energy= 0.277134
Sum of electronic and zero-point Energies= -1286.099068
Sum of electronic and thermal Energies= -1286.078296
Sum of electronic and thermal Enthalpies= -1286.077352
Sum of electronic and thermal Free Energies= -1286.150341

3a-NH

E(scf) = -1286.44749053 a.u.

ν_{min} = 18.3 cm^{-1}

C	-0.188786	2.158861	0.407572	C	2.206852	2.504743	0.731128
C	-1.513973	2.519762	0.424530	H	2.393302	1.490922	0.361894
C	-2.502574	1.542062	0.018421	H	0.725627	3.968210	1.192989
H	0.044329	1.143383	0.065258	C	-6.954069	0.030274	-0.985906
H	-2.122319	0.548020	-0.244567	H	-6.727951	-0.330724	-2.001502
C	-3.843302	1.723370	-0.084418	H	-6.949626	-0.832898	-0.301936
H	-4.321384	2.676499	0.146765	H	-7.936069	0.519071	-0.969436
C	0.928313	2.966001	0.812530	N	3.363630	3.212690	1.021834
C	-4.714742	0.623710	-0.522329	C	3.228058	4.520825	1.661396
O	-4.361669	-0.505330	-0.812532	H	2.592914	5.174358	1.047210
O	-6.007553	1.015189	-0.571318	H	2.785599	4.436448	2.670060

H	4.219809	4.984602	1.741197	H	-3.085878	3.202254	2.545396
C	4.523046	2.427881	1.460588	C	-1.957657	6.301691	0.490202
H	5.440399	3.010805	1.296253	H	-0.925710	4.889150	-0.781311
H	4.451917	2.162498	2.530229	C	-2.767216	6.472606	1.618173
H	4.589579	1.504037	0.869340	H	-3.802493	5.478352	3.238823
C	-1.951397	3.887742	0.836317	H	-1.640185	7.169543	-0.093711
C	-2.768759	4.072817	1.966108	H	-3.083126	7.473944	1.921678
C	-1.554856	5.020028	0.102606	H	3.934342	3.753789	-0.542463
C	-3.171615	5.353537	2.354900	Cl	4.475642	4.303770	-1.635015

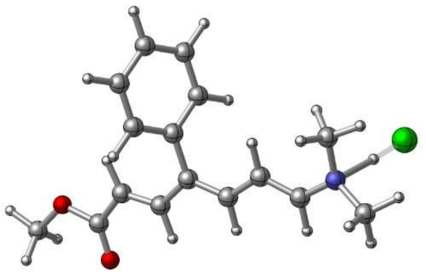

Zero-point correction= 0.326200 (Hartree/Particle)

Thermal correction to Energy= 0.348200

Thermal correction to Enthalpy= 0.349144

Thermal correction to Gibbs Free Energy= 0.271055

Sum of electronic and zero-point Energies= -1286.121291

Sum of electronic and thermal Energies= -1286.099291

Sum of electronic and thermal Enthalpies= -1286.098346

Sum of electronic and thermal Free Energies= -1286.176436

3b-NH

E(scf) = -1286.44790932 a.u.

ν_{min} = 18.5 cm^{-1}

C	0.216220	2.671545	-0.687398	O	-5.506681	1.078548	0.560321
C	-1.146048	2.540807	-0.826426	C	2.300904	3.625867	-1.645401
C	-2.027474	1.856318	0.075288	H	2.826783	3.480528	-0.698724
H	-1.595975	1.350130	0.944496	H	0.445004	3.503502	-2.675319
C	-3.375722	1.774606	-0.074673	C	-6.404718	0.389629	1.429892
H	-3.892979	2.254678	-0.909279	H	-6.163909	-0.684120	1.475104
C	0.984866	3.300620	-1.747630	H	-6.356555	0.798862	2.451275
C	-4.196257	1.032431	0.890816	H	-7.409532	0.533093	1.013499
O	-3.790250	0.439536	1.874679	N	3.128401	4.054815	-2.671965

C	2.528583	4.379632	-3.963213	C	1.961837	1.203047	0.380533
H	1.962833	3.515073	-4.339346	C	1.260489	2.106597	2.931583
H	1.848788	5.247872	-3.893175	H	-0.191315	3.351985	1.923210
H	3.326039	4.608443	-4.682267	C	2.620830	0.702960	1.505980
C	4.287253	4.867762	-2.294235	H	2.248898	0.859128	-0.616186
H	5.043718	4.820199	-3.090365	C	2.272585	1.152665	2.785127
H	4.007953	5.923956	-2.129918	H	0.987653	2.469199	3.925862
H	4.730141	4.472023	-1.369470	H	3.411630	-0.041458	1.382995
H	-1.605663	2.972730	-1.722722	H	2.791575	0.762987	3.664493
C	0.932016	2.154872	0.516777	H	3.776907	2.431261	-2.999884
C	0.594937	2.602677	1.806583	Cl	4.081342	1.153209	-3.238208

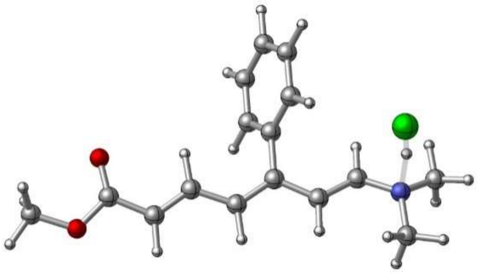

Zero-point correction= 0.326183 (Hartree/Particle)

Thermal correction to Energy= 0.348161

Thermal correction to Enthalpy= 0.349105

Thermal correction to Gibbs Free Energy= 0.271647

Sum of electronic and zero-point Energies= -1286.121727

Sum of electronic and thermal Energies= -1286.099748

Sum of electronic and thermal Enthalpies= -1286.098804

Sum of electronic and thermal Free Energies= -1286.176263

7. Reference

1. Bruker AXS (**2021**) *APEX4 Version 2021.4-0*, *SAINT Version 8.40B* and *SADABS Bruker AXS area detector scaling and absorption correction Version 2016/2*, Bruker AXS Inc., Madison, Wisconsin, USA.

2. Sheldrick, G. M., *SHELXT – Integrated space-group and crystal-structure determination*, *Acta Cryst.,* **2015**, *A71*, 3-8.

3. Sheldrick, G.M., *Crystal structure refinement with SHELXL*, *Acta Cryst.*, **2015**, *C71 (1)*, 3-8.

4. Bruker AXS (**1998**) *XP – Interactive molecular graphics, Version 5.1,* Bruker AXS Inc., Madison, Wisconsin, USA.

5. (a) Lee, C.; Yang, W.; Parr, R. G., Development of the Colle-Salvetti Correlation-Energy Formula into a Functional of the Electron Density. *Phys. Rev. B* **1988**, *37*, 785–789. (b) Becke, A. D., Density-Functional Thermochemistry. III. The Role of Exact Exchange. *J. Chem. Phys.* **1993**, *98*, 5648–5652.

6. (a) Grimme, S. Accurate description of van der Waals complexes by density functional theory including empirical corrections. *J. Comput. Chem.* **2004**, *25*, 1463-1473. (b) Grimme, S.; Antony, J.; Ehrlich, S.; Krieg, H. A consistent and accurate ab initio parametrization of density functional dispersion correction (DFT-D) for the 94 elements H-Pu. *J. Chem. Phys.* **2010**, *132*, 154104. (c) Grimme, S. Density functional theory with London dispersion corrections. *WIRES Comput. Mol. Sci.* **2011**, *1*, 211-228. (d) Ehrlich, S.; Moellmann, J.; Grimme, S. Dispersion-Corrected Density Functional Theory for Aromatic Interactions in Complex Systems. *Acc. Chem. Res.* **2012**, *46*, 916-926.

7. (a) Weigend, F.; Ahlrichs, R. Balanced basis sets of split valence, triple zeta valence and quadruple zeta valence quality for H to Rn: Design and assessment of accuracy. *Phys. Chem. Chem. Phys.* **2005**, *7*, 3297-3305. (b) Weigend, F. Accurate Coulomb-fitting basis sets for H to Rn. *Phys. Chem. Chem. Phys.* **2006**, *8*, 1057-1065.

8. (a) Klamt, A.; Schüürmann, G. COSMO: a new approach to dielectric screening in solvents with explicit expressions for the screening energy and its gradient. *J. Chem. Soc. Perkin Trans. 2* **1993**, *0*, 799-805. (b) Tomasi, J.; Persico, M. Molecular Interactions in Solution: An Overview of Methods Based on Continuous Distributions of the Solvent. *Chem. Rev.* **1994**, *94*, 2027-2094. (c) Andzelm, J.; Kölmel, C.; Klamt, A. Incorporation of solvent effects into density functional calculations of molecular energies and geometries. *J. Chem. Phys.* **1995**, *103*, 9312-9320. (d) Barone, V.; Cossi, M. Quantum Calculation of Molecular Energies and Energy Gradients in Solution by a Conductor Solvent Model. *J. Phys. Chem. A* **1998**, *102*, 1995-2001. (e) Cossi, M.; Rega, N.; Scalmani, G.; Barone, V. Energies, structures, and electronic properties of molecules in solution with the C-PCM solvation model. *J. Comput. Chem.* **2003**, *24*, 669-681.

9. Gaussian 16, Revision C.01, Frisch, M. J.; Trucks, G. W.; Schlegel, H. B.; Scuseria, G. E.; Robb, M. A.; Cheeseman, J. R.; Scalmani, G.; Barone, V.; Petersson, G. A.; Nakatsuji, H.; Li, X.; Caricato, M.; Marenich,

A. V.; Bloino, J.; Janesko, B. G.; Gomperts, R.; Mennucci, B.; Hratchian, H. P.; Ortiz, J. V.; Izmaylov, A. F.; Sonnenberg, J. L.; Williams-Young, D.; Ding, F.; Lipparini, F.; Egidi, F.; Goings, J.; Peng, B.; Petrone, A.; Henderson, T.; Ranasinghe, D.; Zakrzewski, V. G.; Gao, J.; Rega, N.; Zheng, G.; Liang, W.; Hada, M.; Ehara, M.; Toyota, K.; Fukuda, R.; Hasegawa, J.; Ishida, M.; Nakajima, T.; Honda, Y.; Kitao, O.; Nakai, H.; Vreven, T.; Throssell, K.; Montgomery, J. A., Jr.; Peralta, J. E.; Ogliaro, F.; Bearpark, M. J.; Heyd, J. J.; Brothers, E. N.; Kudin, K. N.; Staroverov, V. N.; Keith, T. A.; Kobayashi, R.; Normand, J.; Raghavachari, K.; Rendell, A. P.; Burant, J. C.; Iyengar, S. S.; Tomasi, J.; Cossi, M.; Millam, J. M.; Klene, M.; Adamo, C.; Cammi, R.; Ochterski, J. W.; Martin, R. L.; Morokuma, K.; Farkas, O.; Foresman, J. B.; Fox, D. J. Gaussian, Inc., Wallingford CT, 2016.

10. Legault, C. Y. (2009) CYLview, 1.0b, Université de Sherbrooke: Sherbrooke, Canada, http://www.cylview.org.

8. Copies of ^{1}H, ^{13}C and ^{19}F NMR spectra

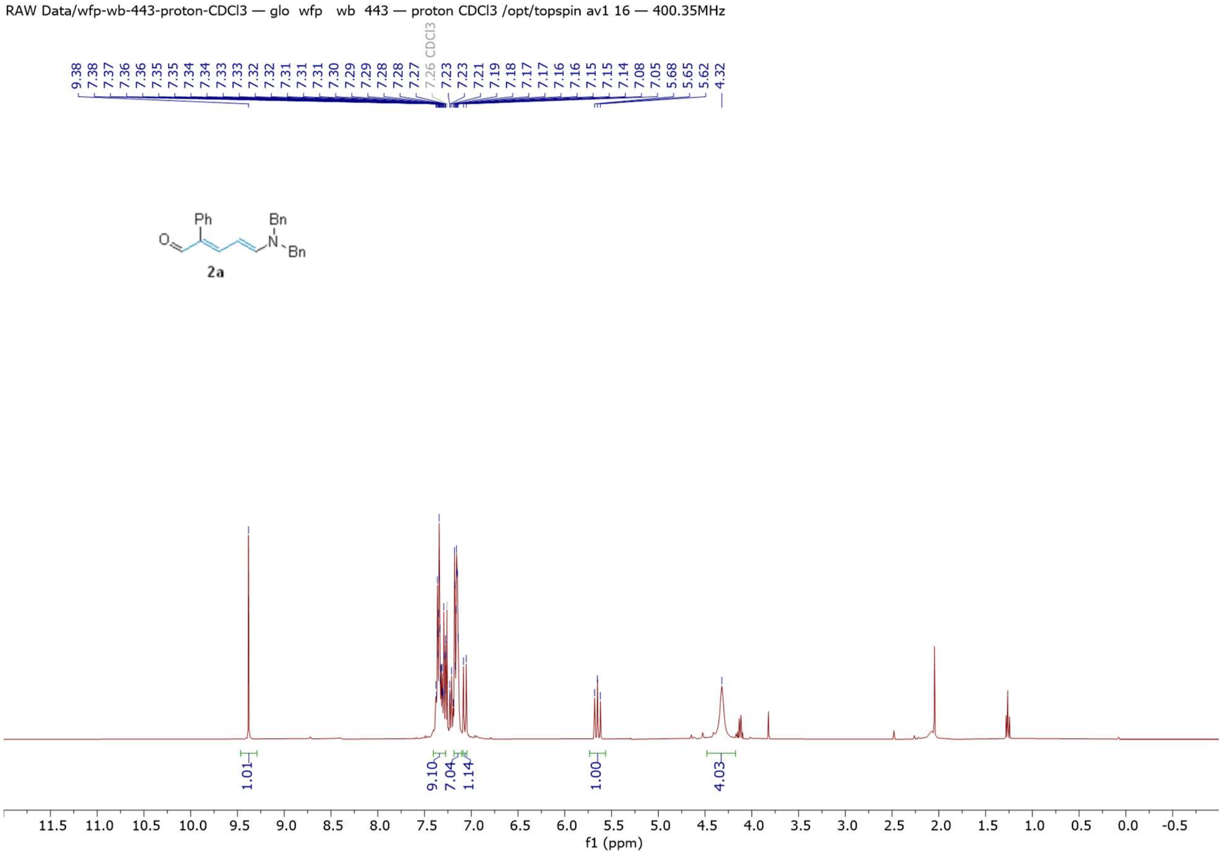

RAW Data/wfp-wb-443-carbon_256-CDCl3 — glo wfp wb 443 — carbon_256 CDCl3 /opt/topspin av1 16 — 100.68MHz

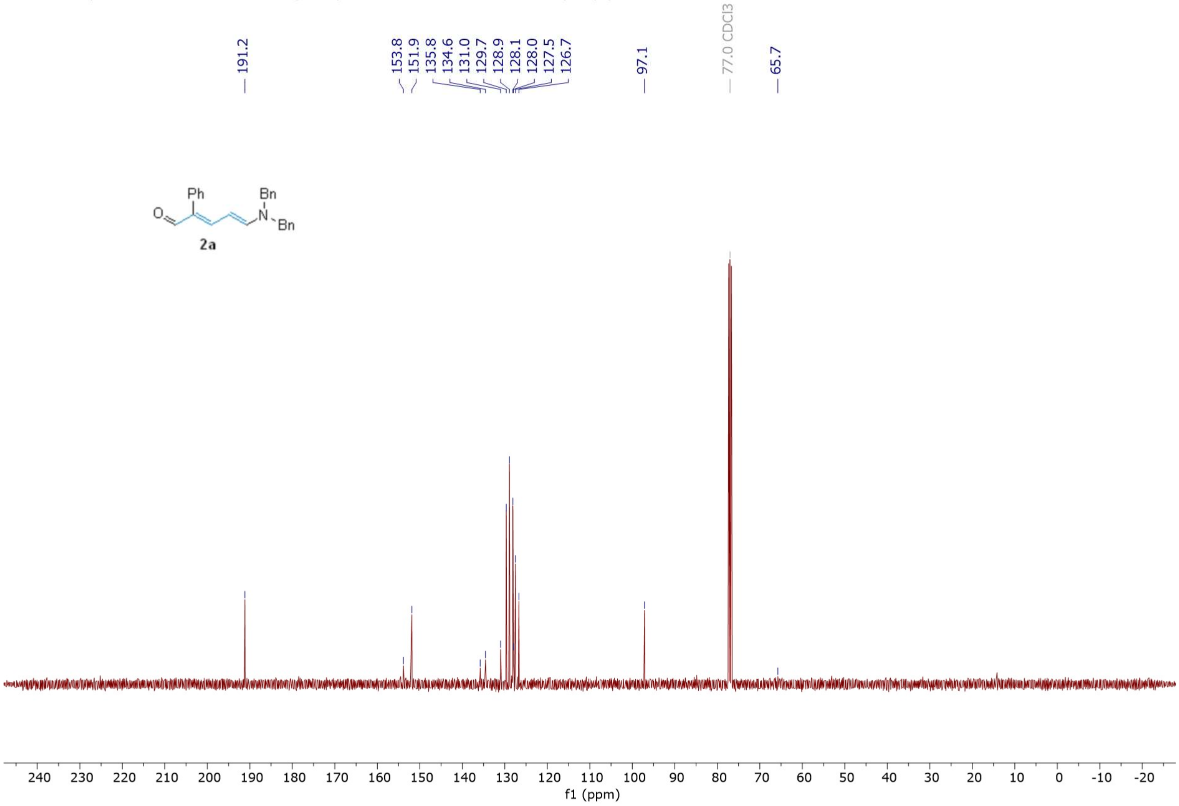

472/wfp-wb-472-proton-CDCl3-2 — glo wfp wb 472 — proton CDCl3 /opt/topspin av1 2 — 400.35MHz

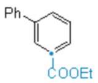

4a

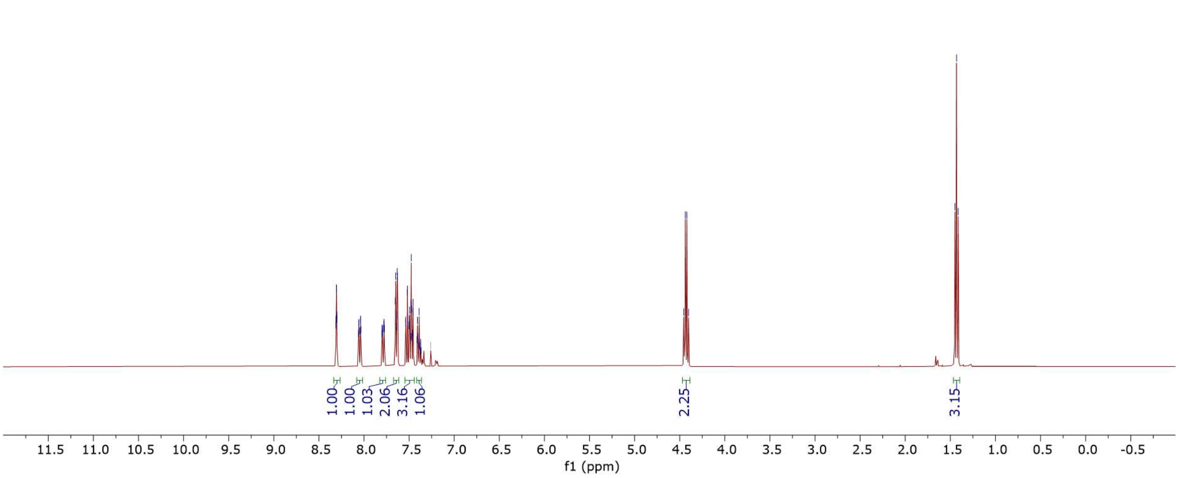

472/wfp-wb-472-carbon_256-CDCl3 — glo wfp wb 472 — carbon_256 CDCl3 /opt/topspin av1 2 — 100.68MHz

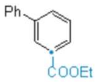

4a

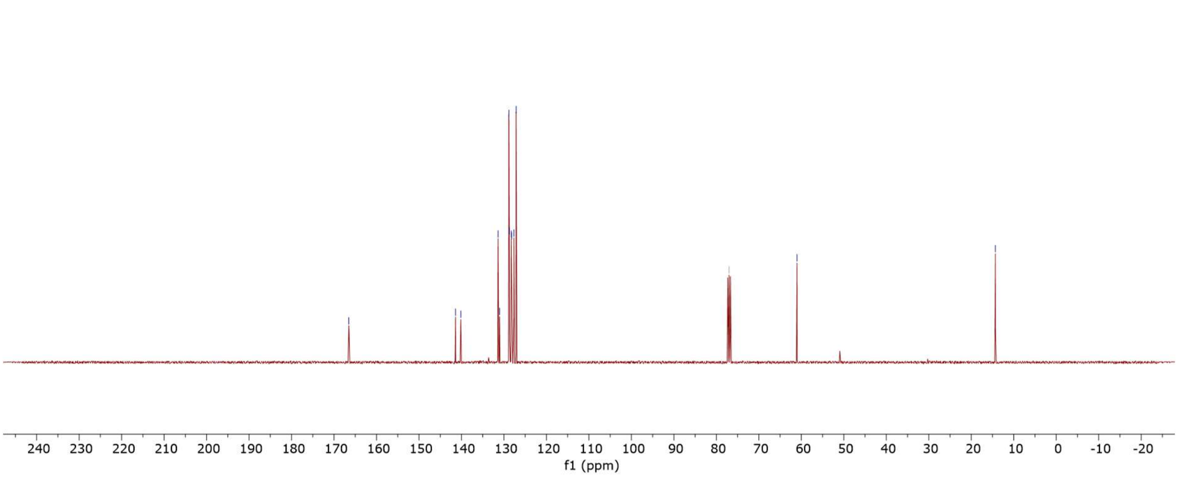

RAW Data/wfp-wb-491-proton-CDCl3-3 — glo wfp wb 491 — proton CDCl3 /opt/topspin av1 17 — 400.13MHz

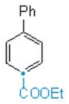

4b

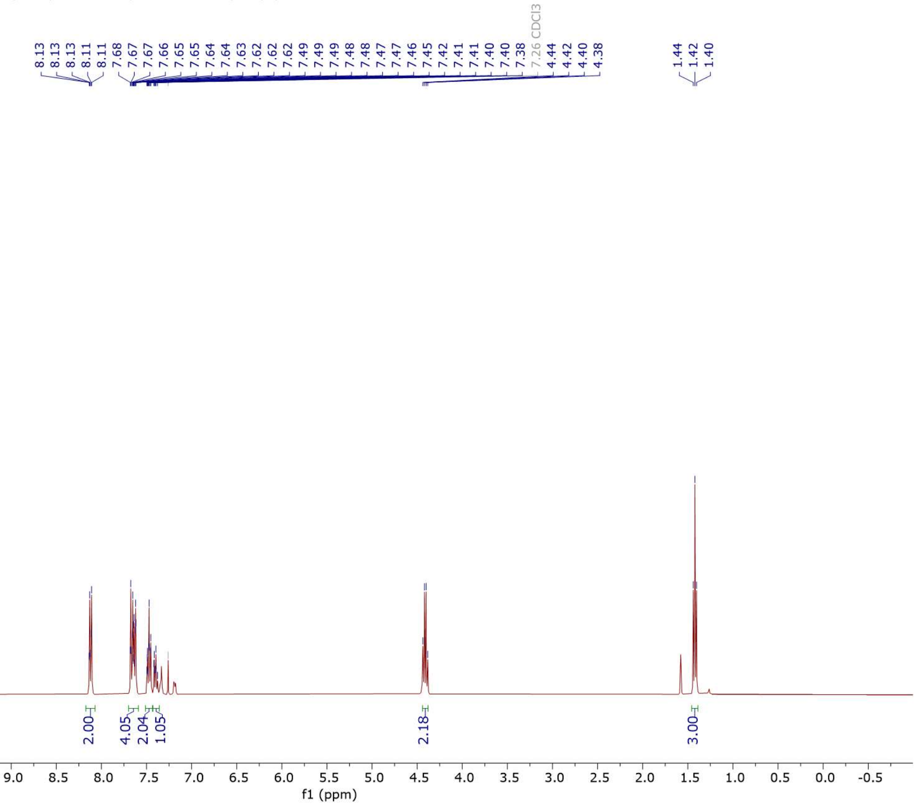

RAW Data/wfp-wb-491-carbon_256-CDCl3-2 — glo wfp wb 491 — carbon_256 CDCl3 /opt/topspin av1 17 — 100.62MHz

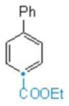

4b

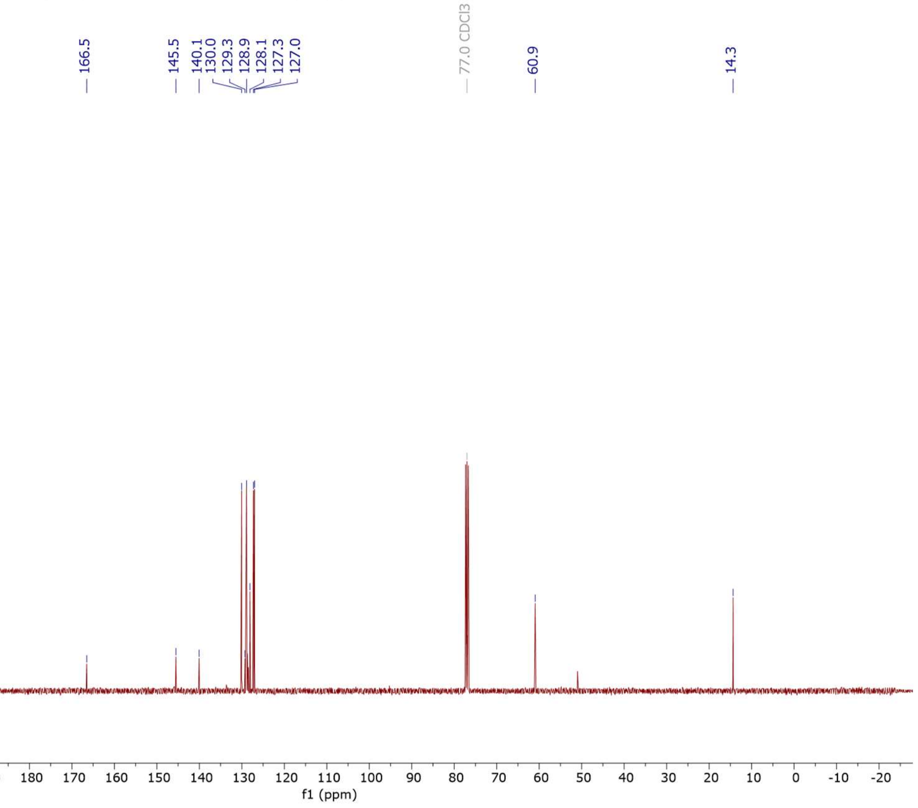

504/wfp-wb-503-2-proton-CDCl3 — glo wfp wb 503 2 — proton CDCl3 /opt/topspin av1 12 — 400.35MHz

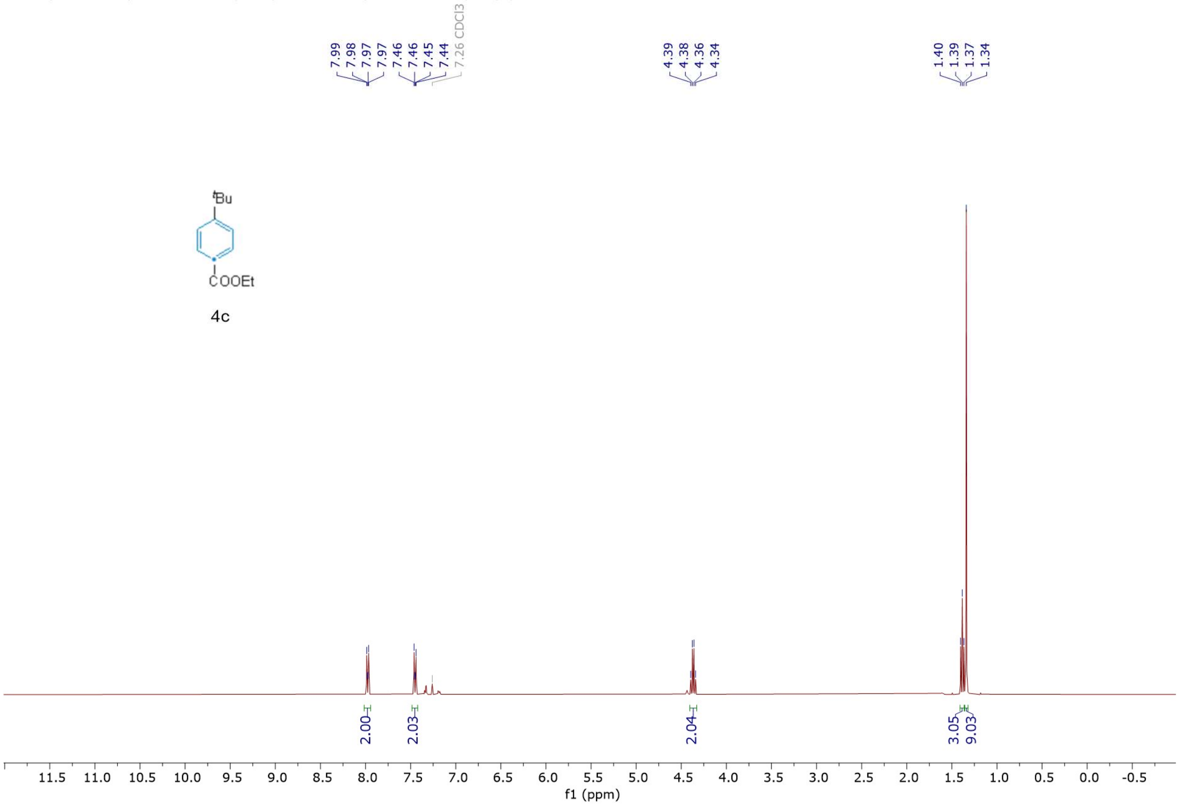

504/wfp-wb-503-2-carbon_256-CDCl3 — glo wfp wb 503 2 — carbon_256 CDCl3 /opt/topspin av1 12 — 100.68MHz

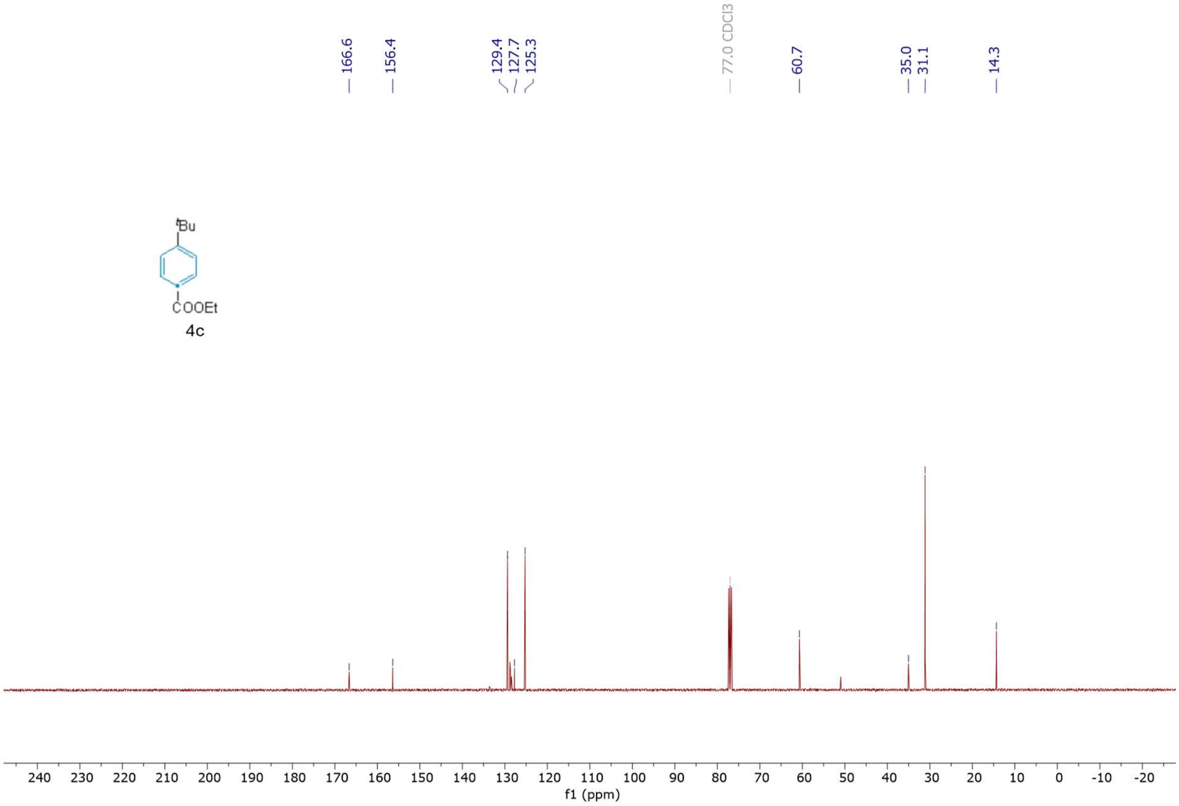

515/wfp-wb-515-proton-CDCl3 — glo wfp wb 513 2 — proton CDCl3 /opt/topspin av1 2 — 400.35MHz

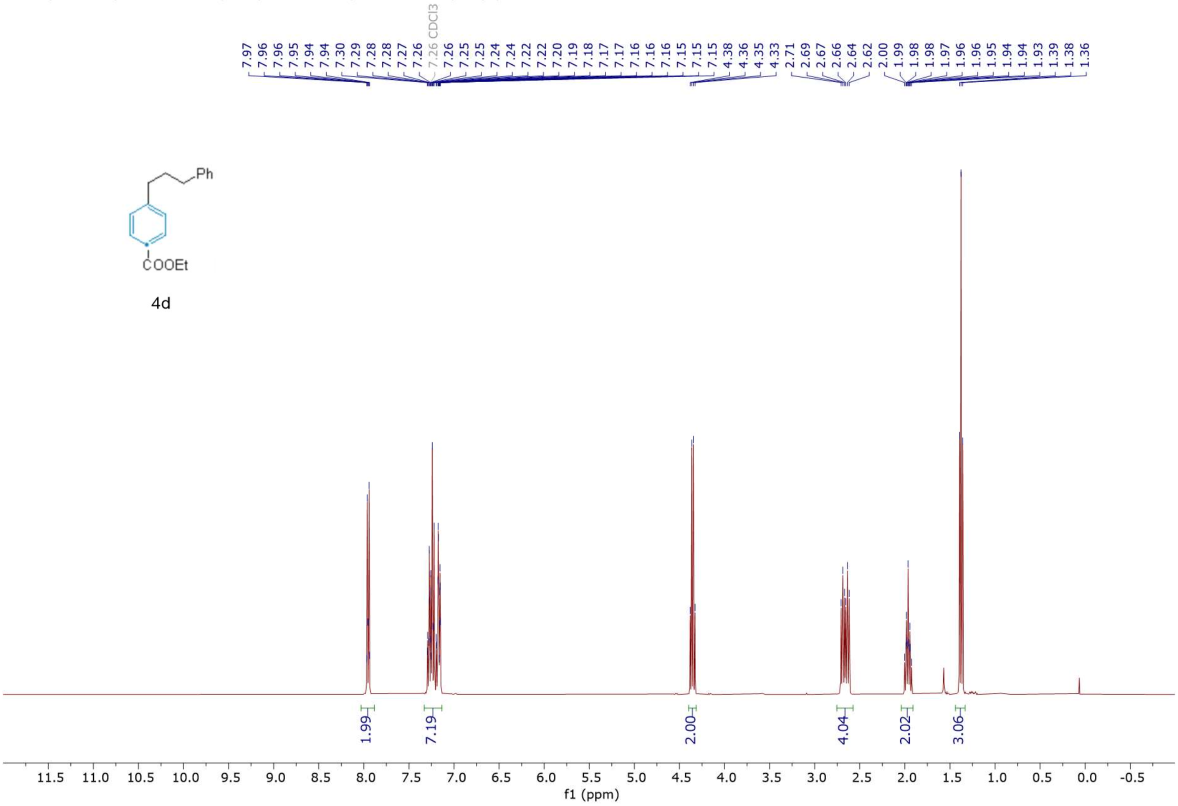

515/wfp-wb-515-carbon_256-CDCl3 — glo wfp wb 513 2 — carbon_256 CDCl3 /opt/topspin av1 2 — 100.68MHz

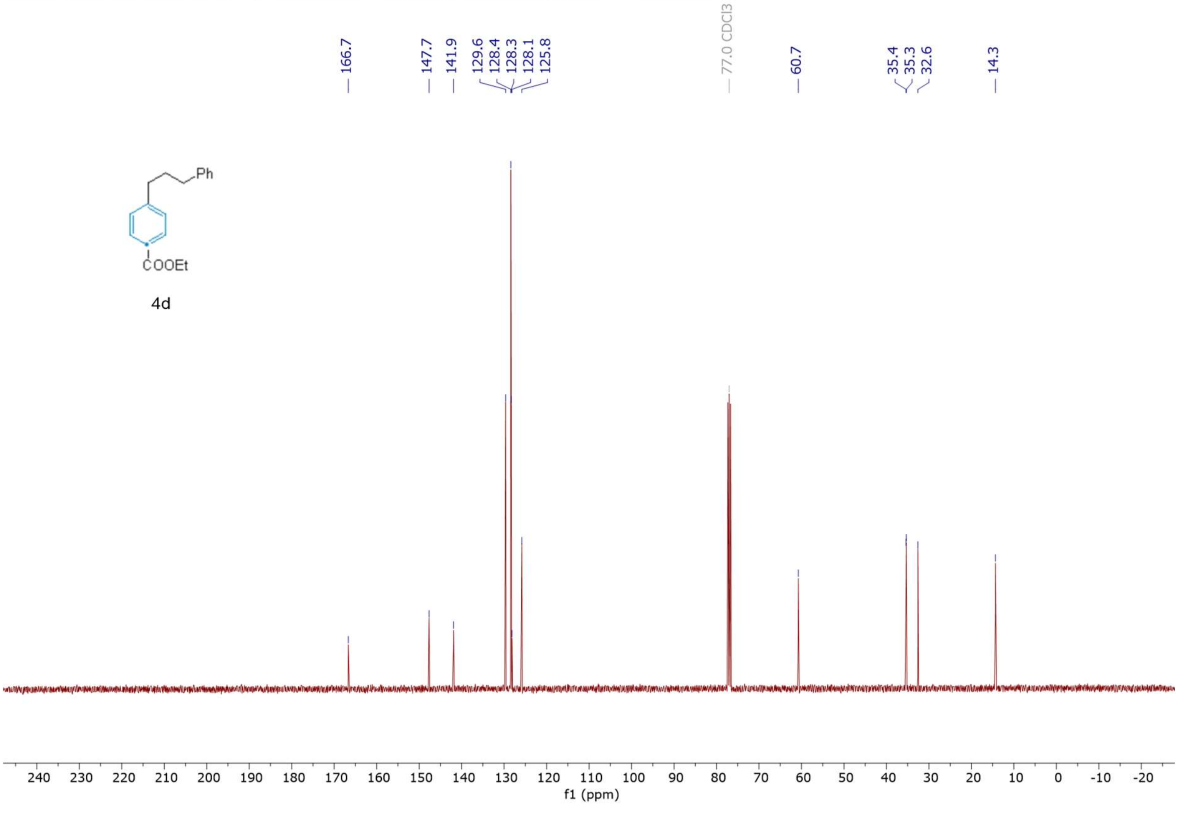

509/wfp-wb-507-3-proton-CDCl3 — glo wfp wb 507 3 — proton CDCl3 /opt/topspin av1 13 — 400.35MHz

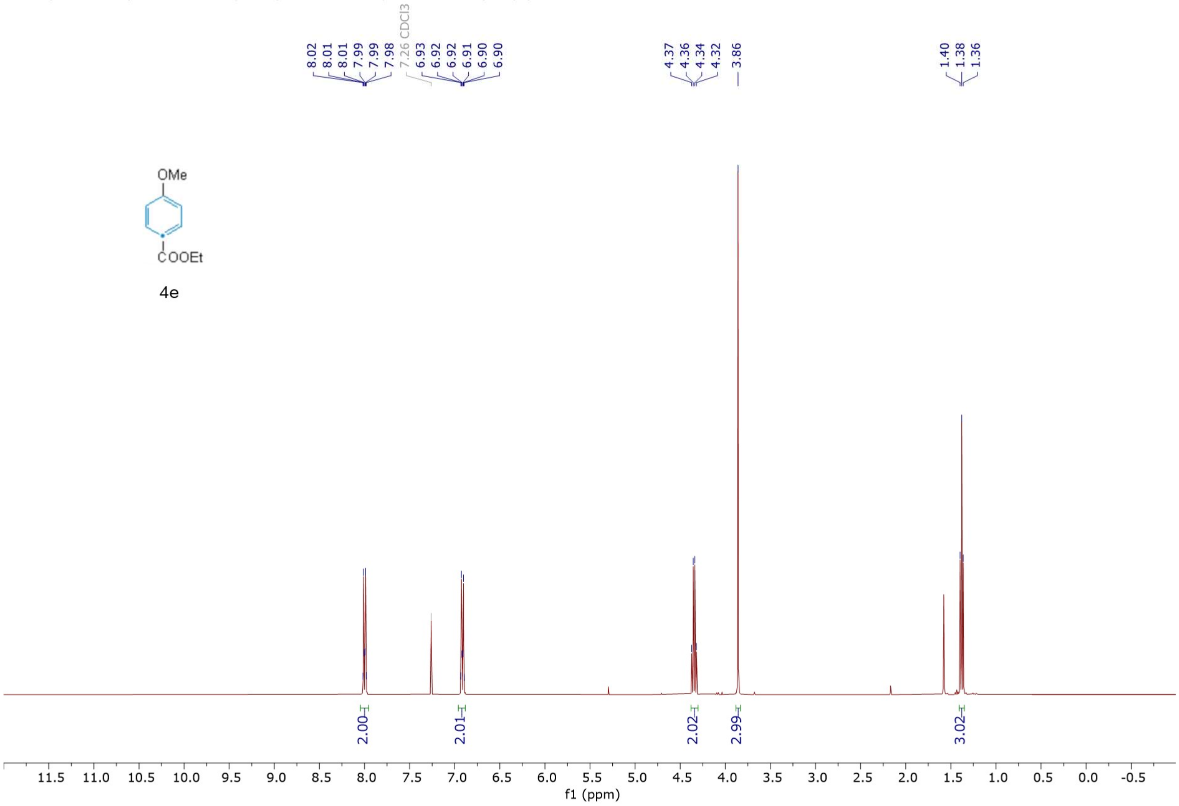

509/wfp-wb-507-3-carbon_256-CDCl3 — glo wfp wb 507 3 — carbon_256 CDCl3 /opt/topspin av1 13 — 100.68MHz

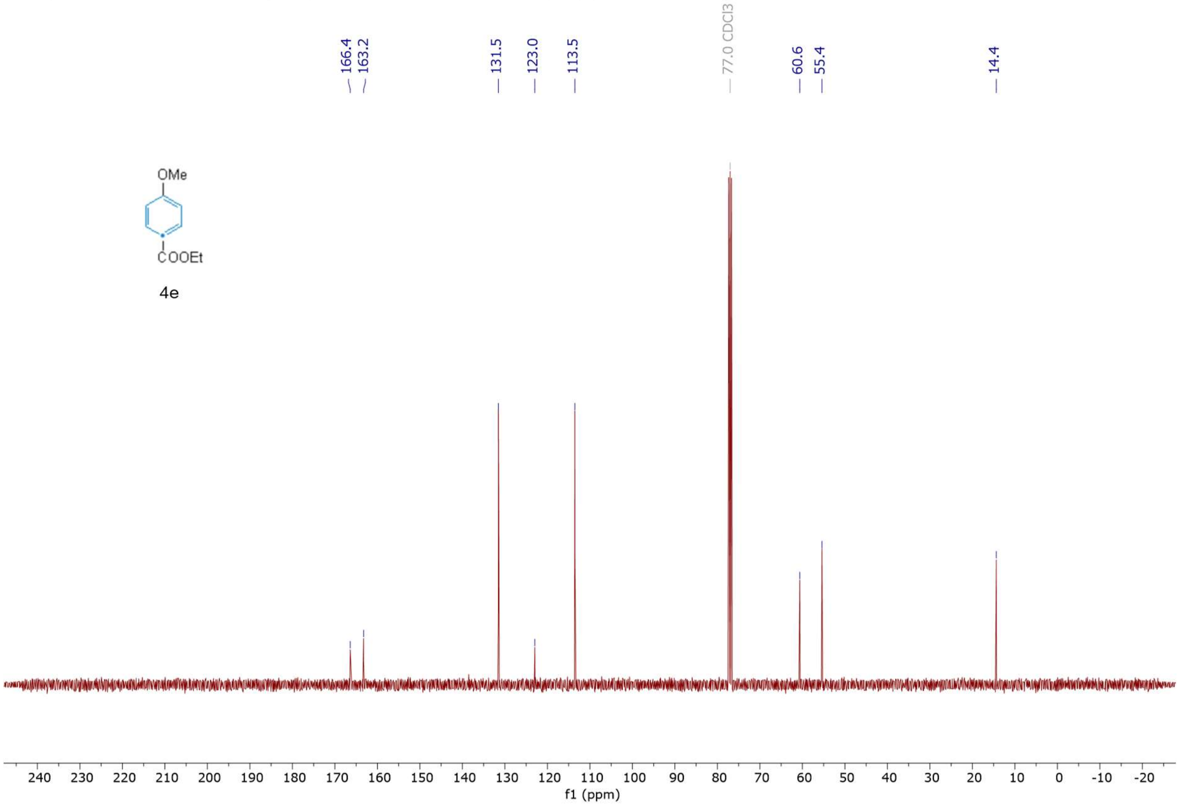

513/wfp-wb-513-1-proton-CDCl3 — glo wfp wb 513 1 — proton CDCl3 /opt/topspin av1 1 — 400.35MHz

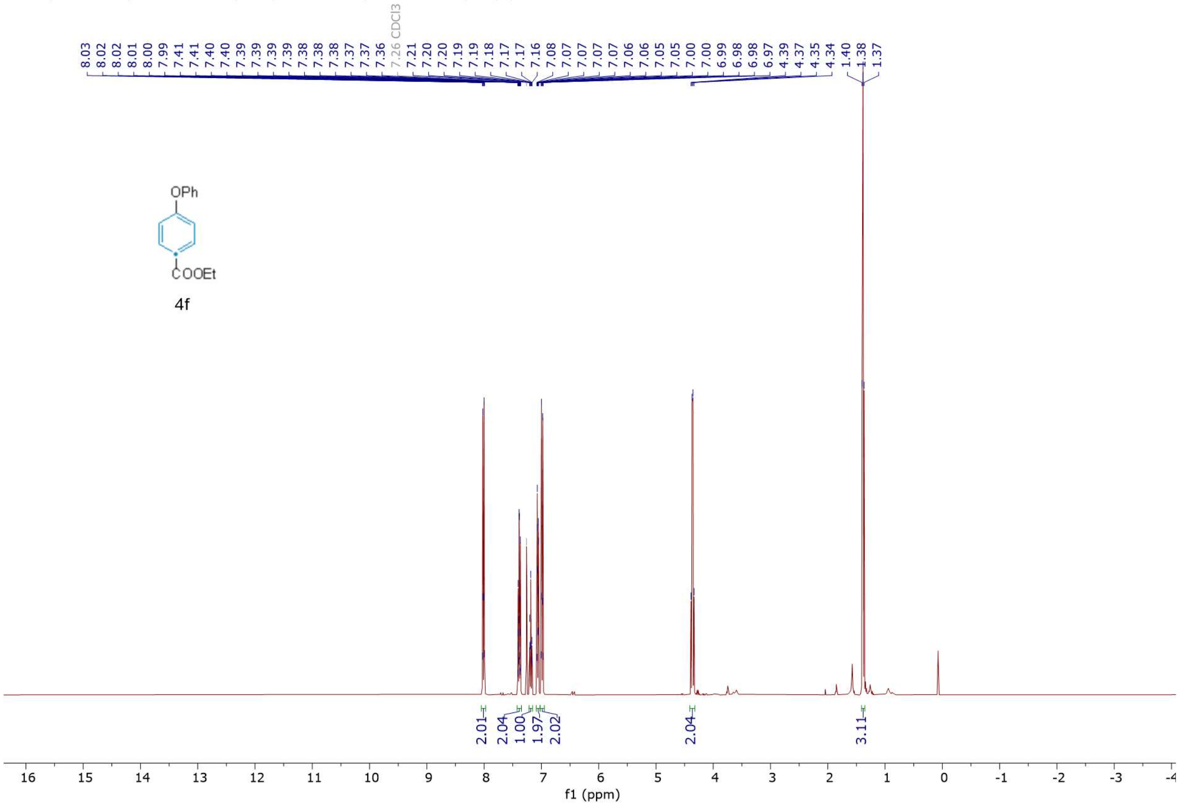

513/wfp-wb-513-1-carbon_256-CDCl3 — glo wfp wb 513 1 — carbon_256 CDCl3 /opt/topspin av1 1 — 100.68MHz

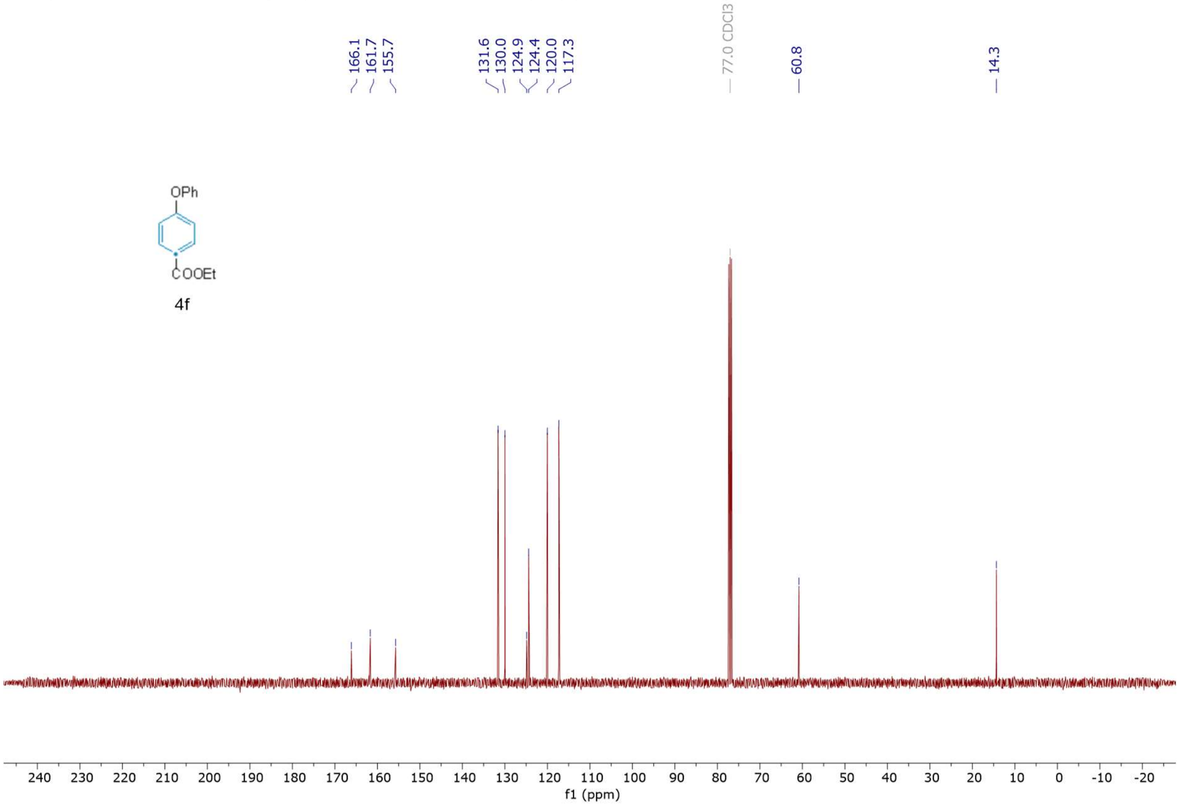

503/wfp-wb-503-1-proton-CDCl3 — glo wfp wb 503 1 — proton CDCl3 /opt/topspin av1 11 — 400.35MHz

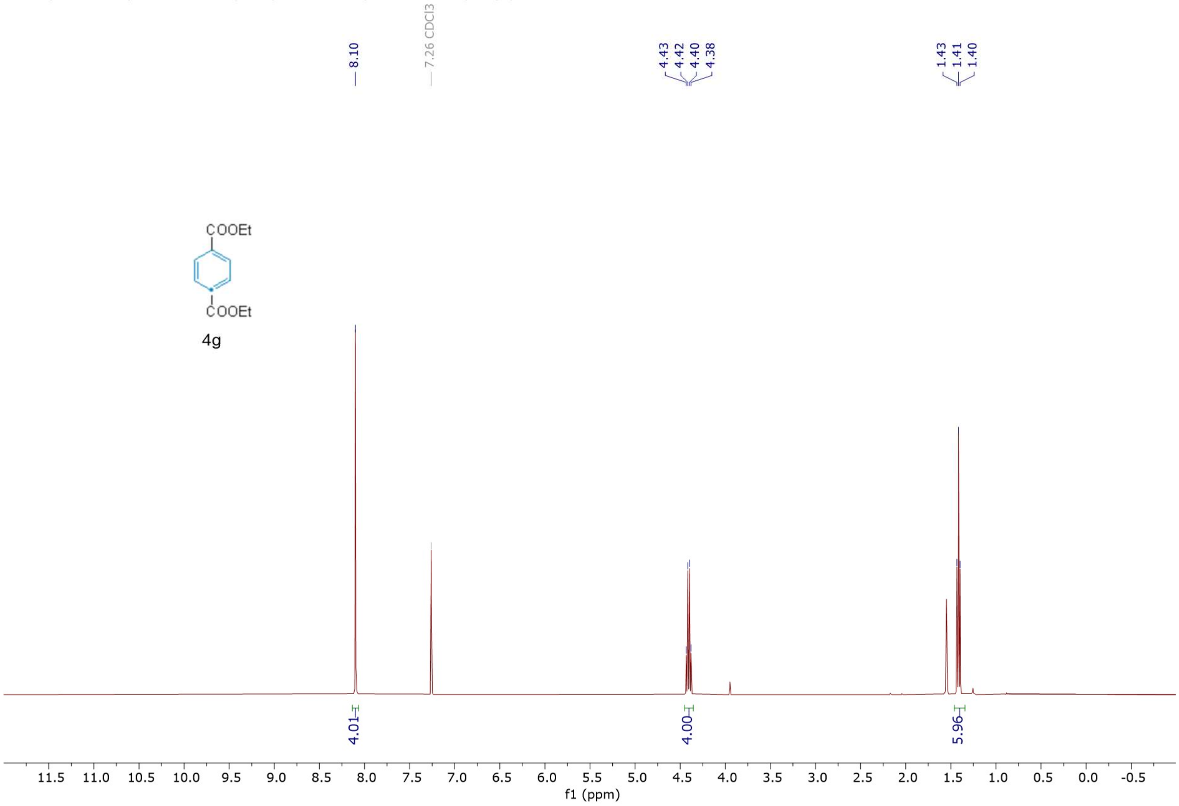

503/wfp-wb-503-1-carbon_256-CDCl3 — glo wfp wb 503 1 — carbon_256 CDCl3 /opt/topspin av1 11 — 100.68MHz

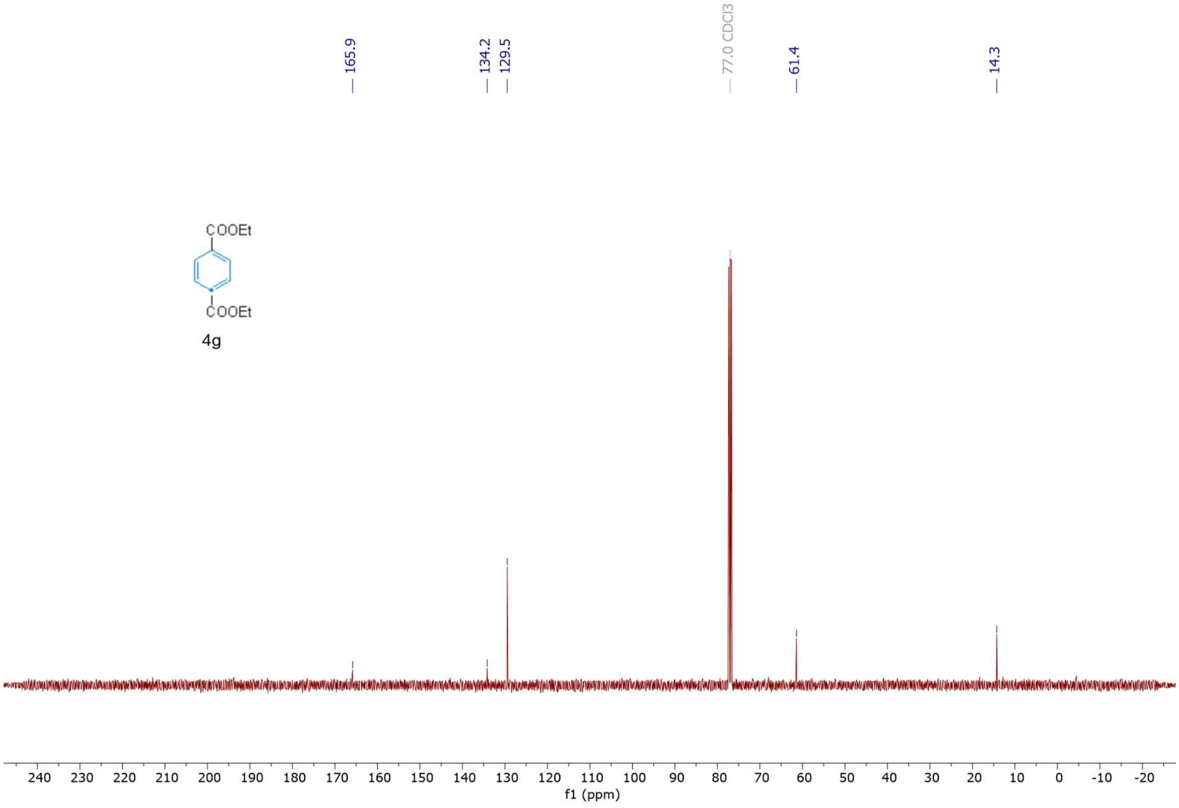

506/wfp-wb-503-3-proton-CDCl3 — glo wfp wb 503 3 — proton CDCl3 /opt/topspin av1 13 — 400.35MHz

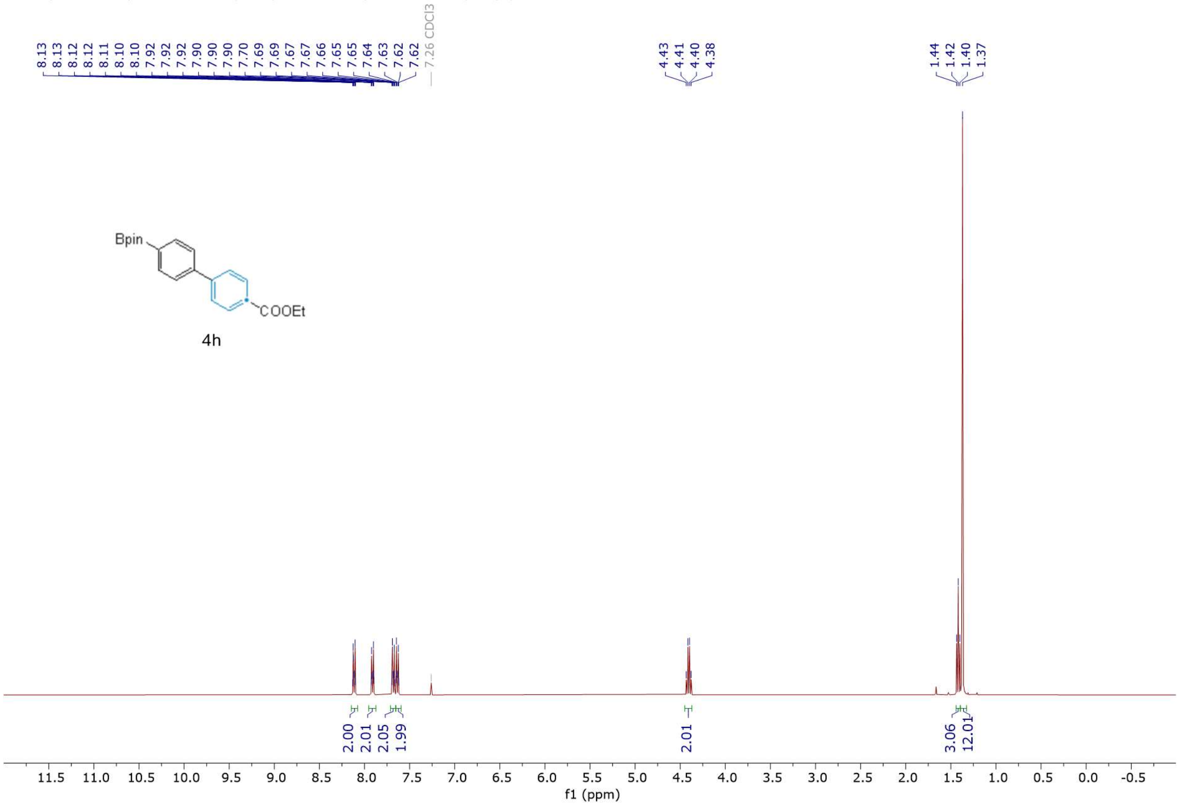

506/wfp-wb-503-3-carbon_256-CDCl3 — glo wfp wb 503 3 — carbon_256 CDCl3 /opt/topspin av1 13 — 100.68MHz

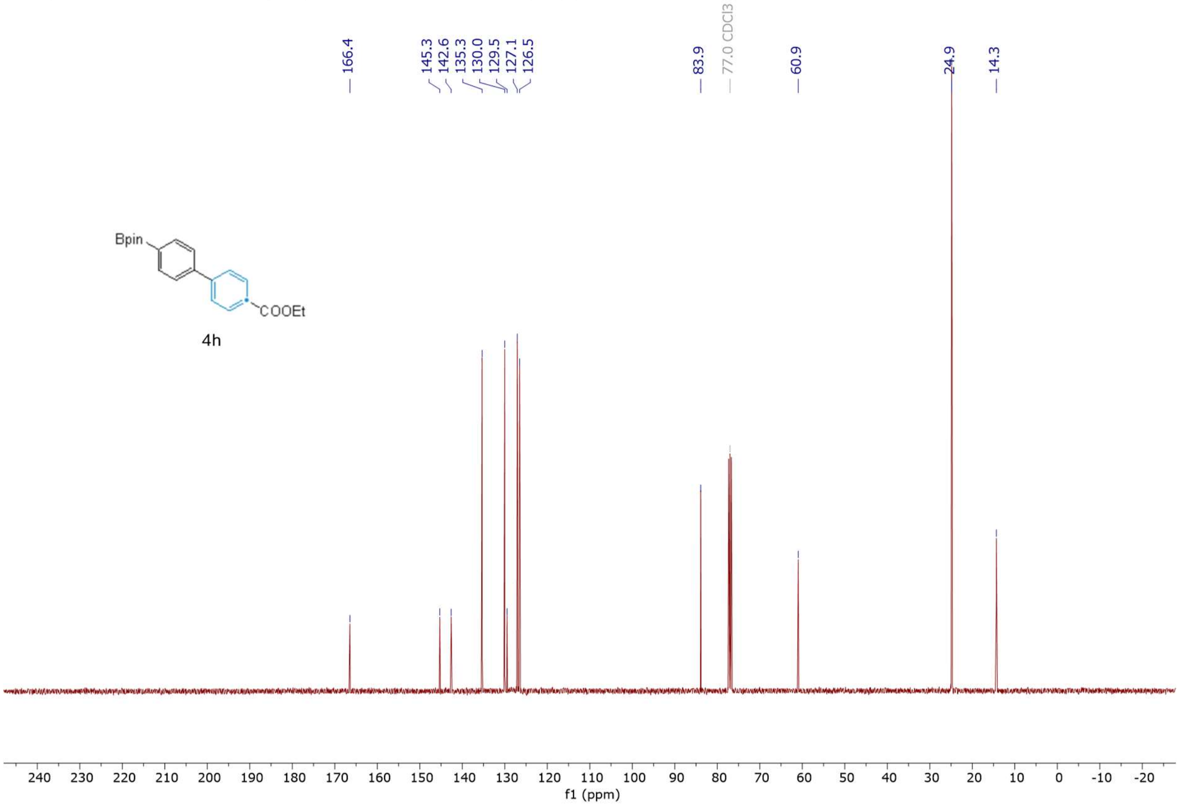

507/wfp-wb-507-1-proton-CDCl3 — glo wfp wb 507 1 — proton CDCl3 /opt/topspin av1 11 — 400.35MHz

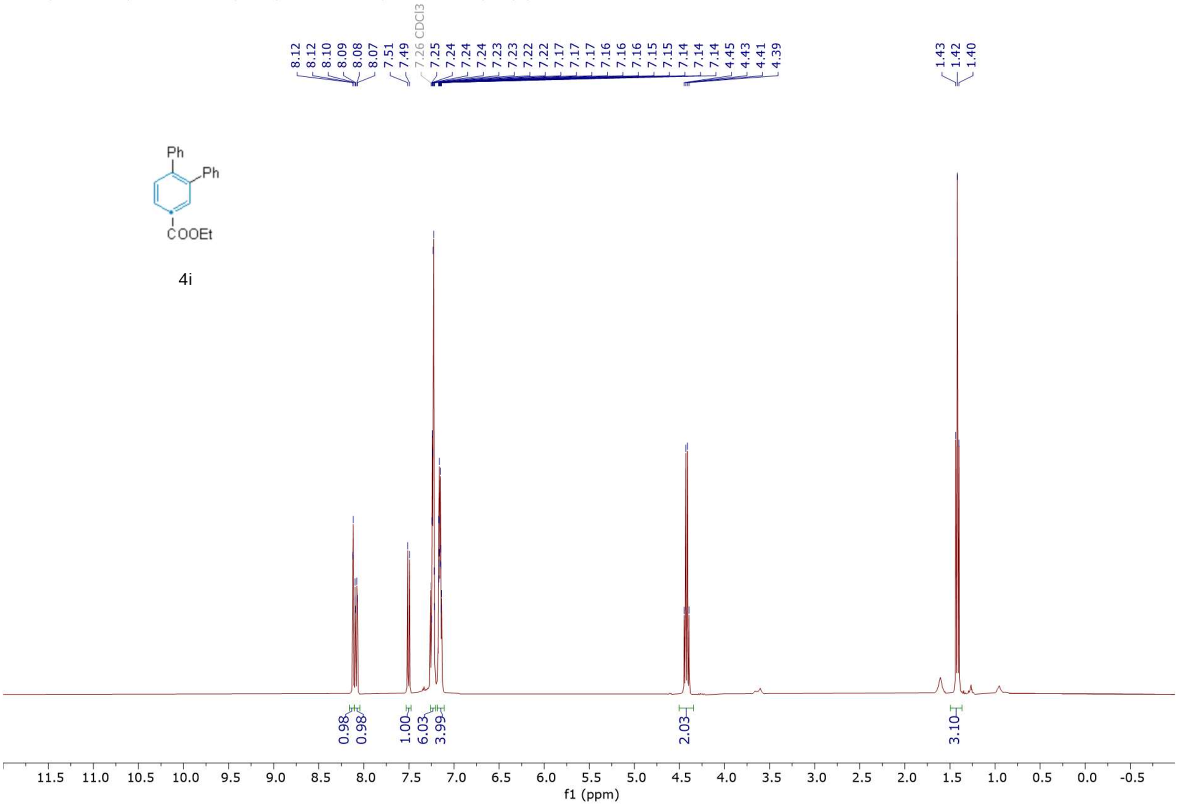

4i

507/wfp-wb-507-1-carbon_256-CDCl3 — glo wfp wb 507 1 — carbon_256 CDCl3 /opt/topspin av1 11 — 100.68MHz

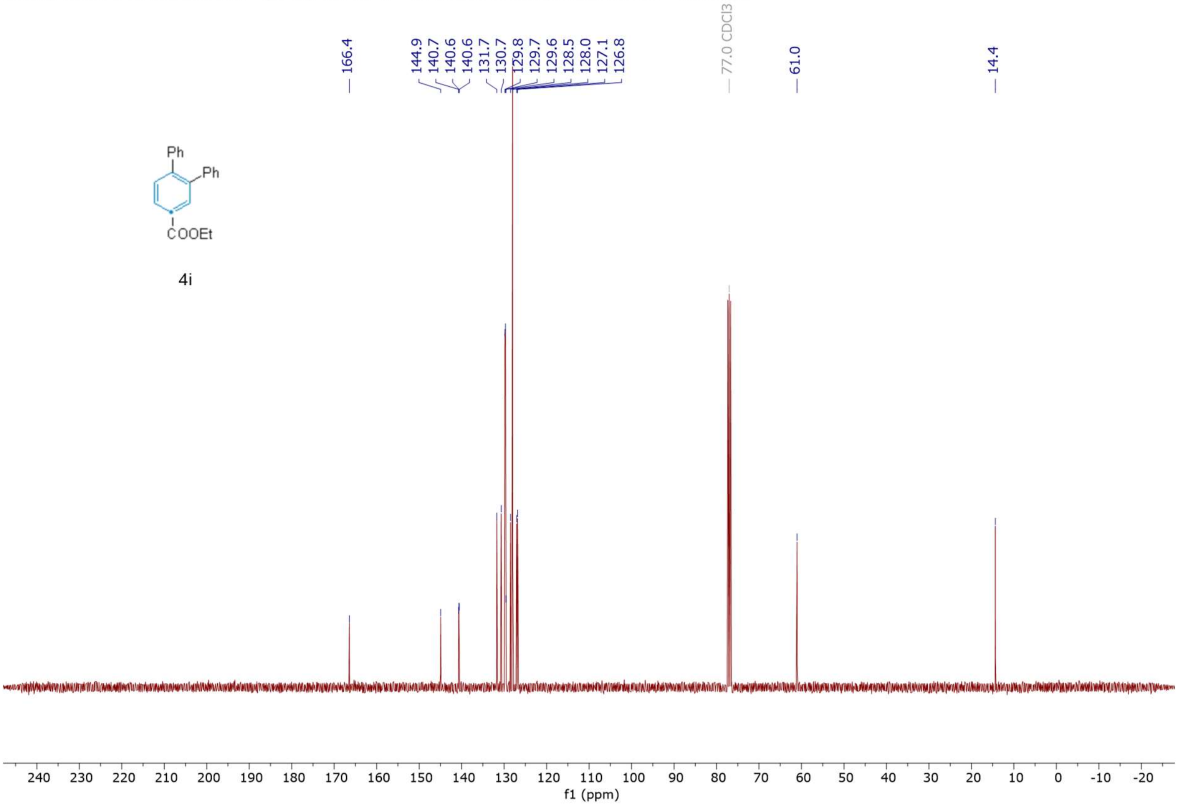

4i

RAW Data/wfp-wb-524-proton-CDCl3 — glo wfp wb 524 — proton CDCl3 /opt/topspin av1 3 — 400.35MHz

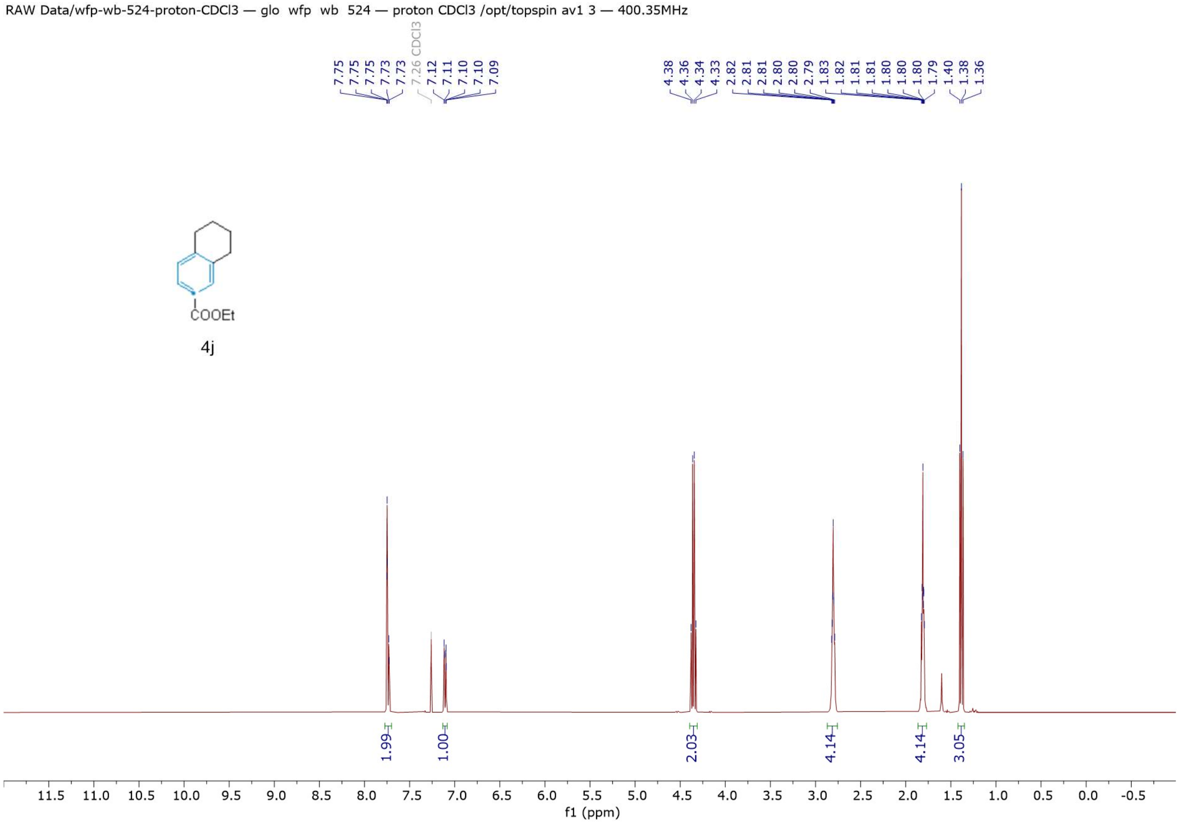

RAW Data/wfp-wb-524-carbon-CDCl3 — glo wfp wb 524 — carbon CDCl3 /opt/topspin av1 3 — 100.68MHz

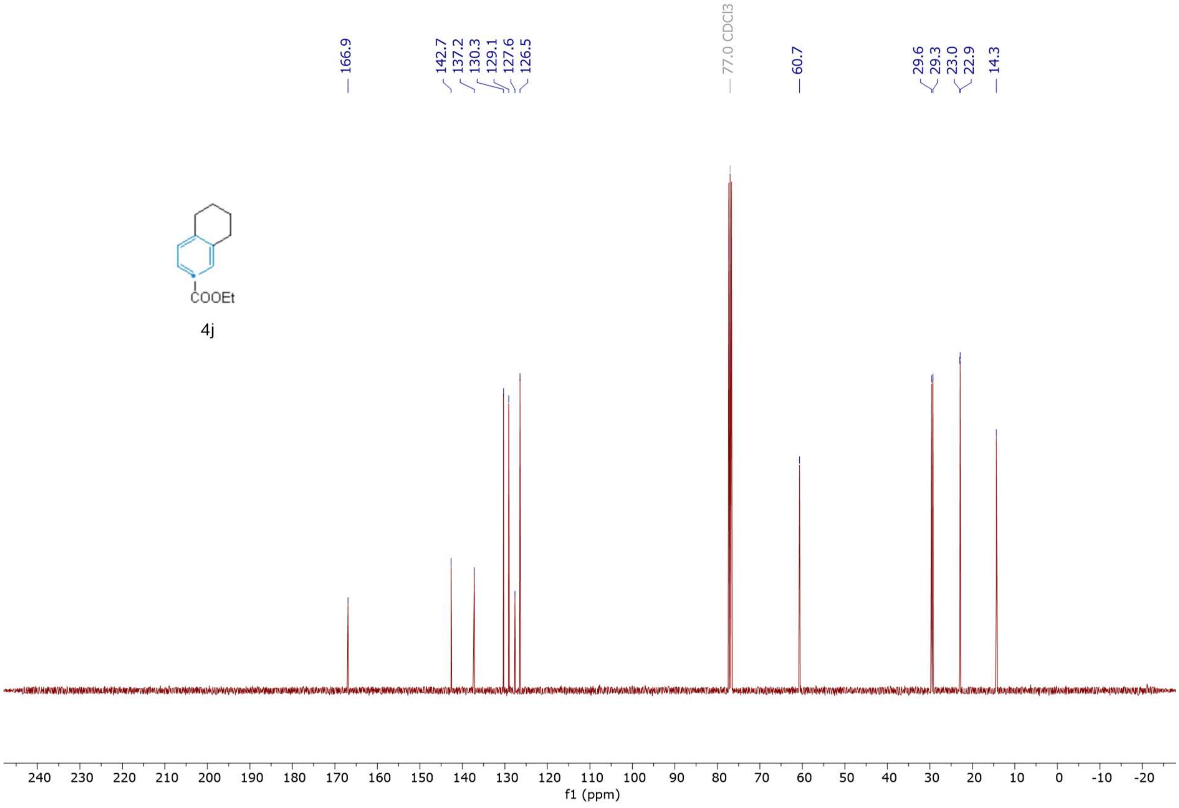

488/wfp-wb-488-proton-CDCl3-2 — glo wfp wb 488 — proton CDCl3 /opt/topspin av1 2 — 400.35MHz

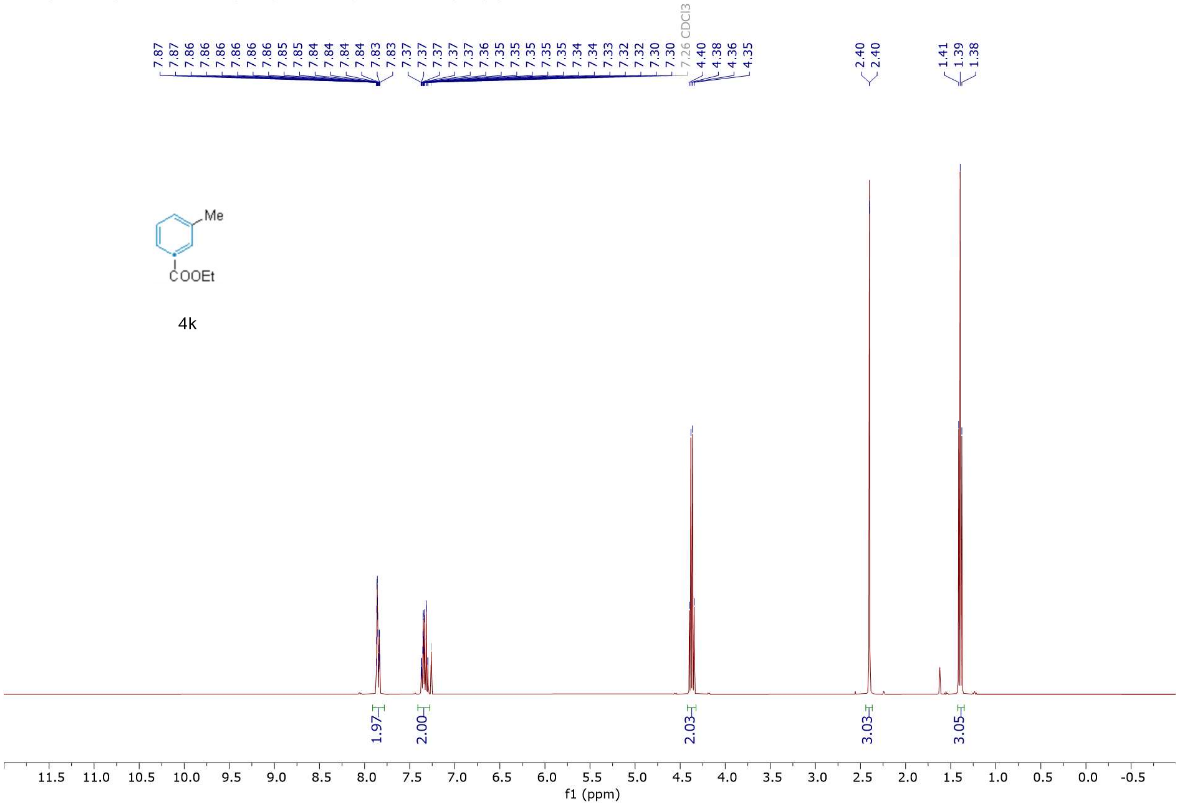

488/wfp-wb-488-carbon_256-CDCl3-2 — glo wfp wb 488 — carbon_256 CDCl3 /opt/topspin av1 2 — 100.68MHz

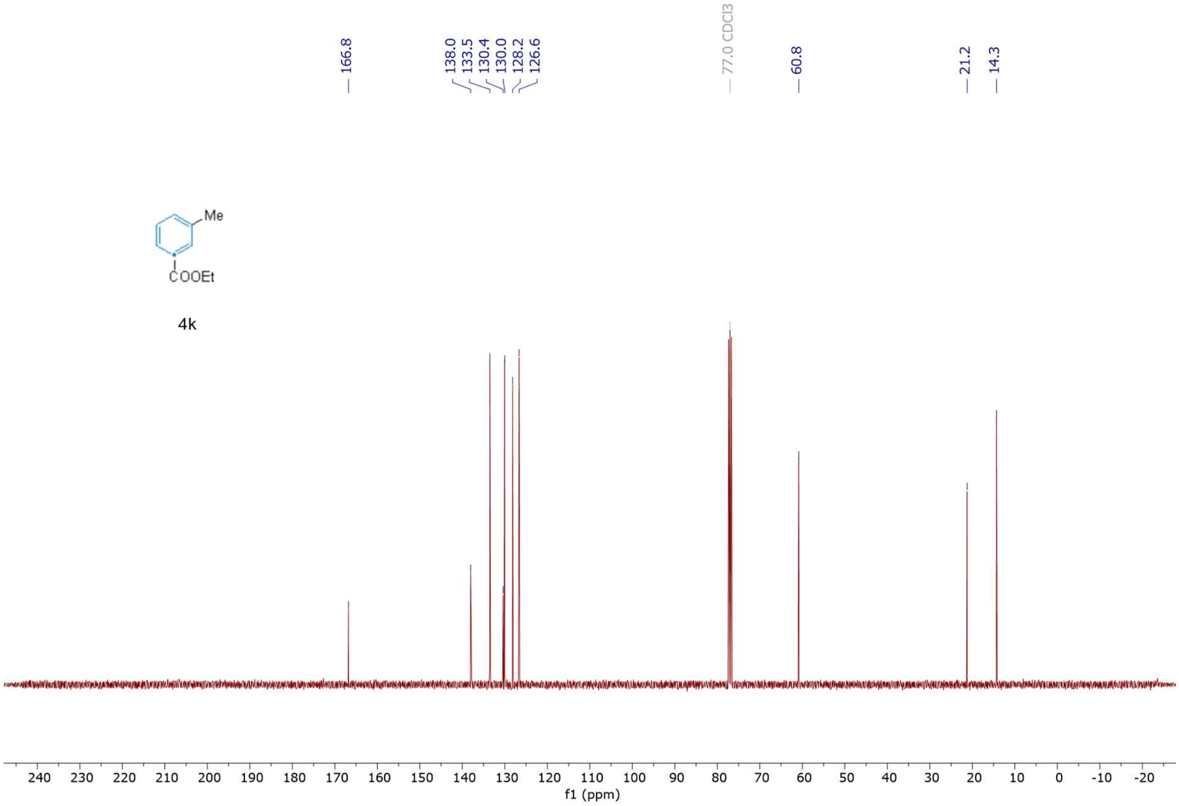

501/wfp-wb-501-2-proton-CDCl3 — glo wfp wb 501 2 — proton CDCl3 /opt/topspin av1 22 — 400.13MHz

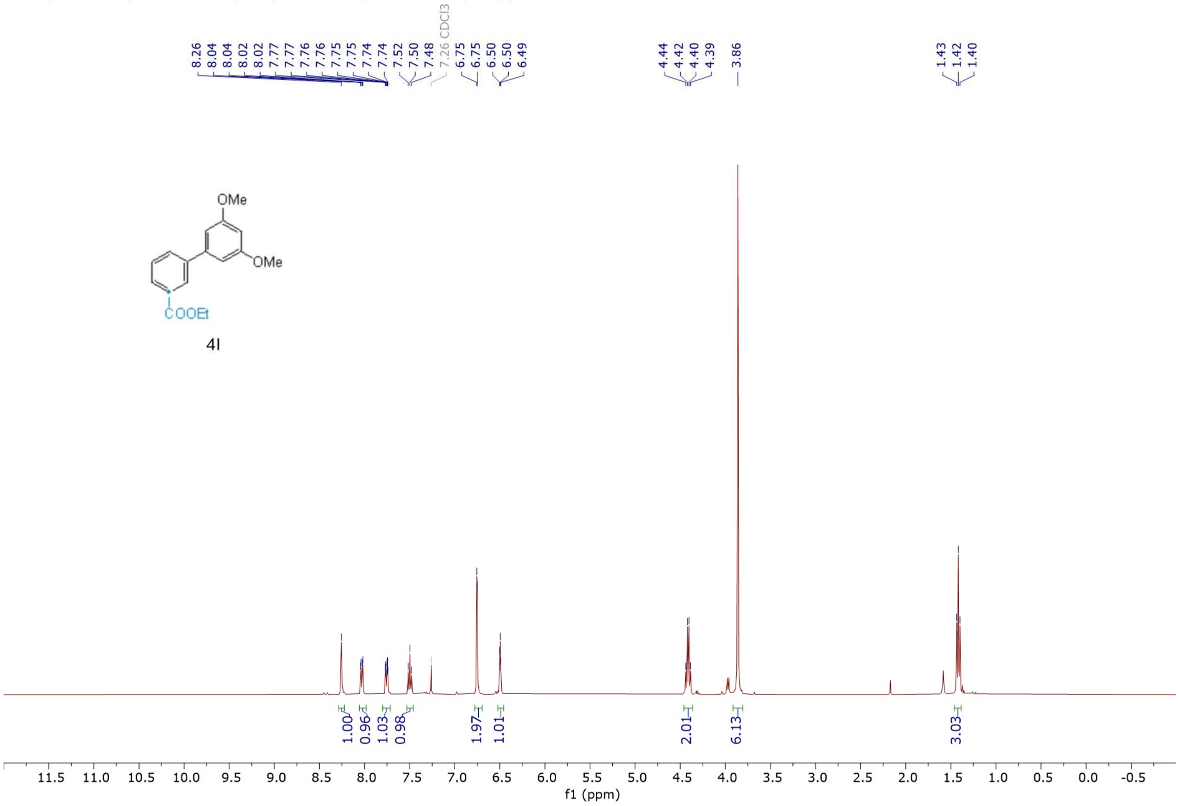

501/wfp-wb-501-2-carbon_256-CDCl3 — glo wfp wb 501 2 — carbon_256 CDCl3 /opt/topspin av1 22 — 100.62MHz

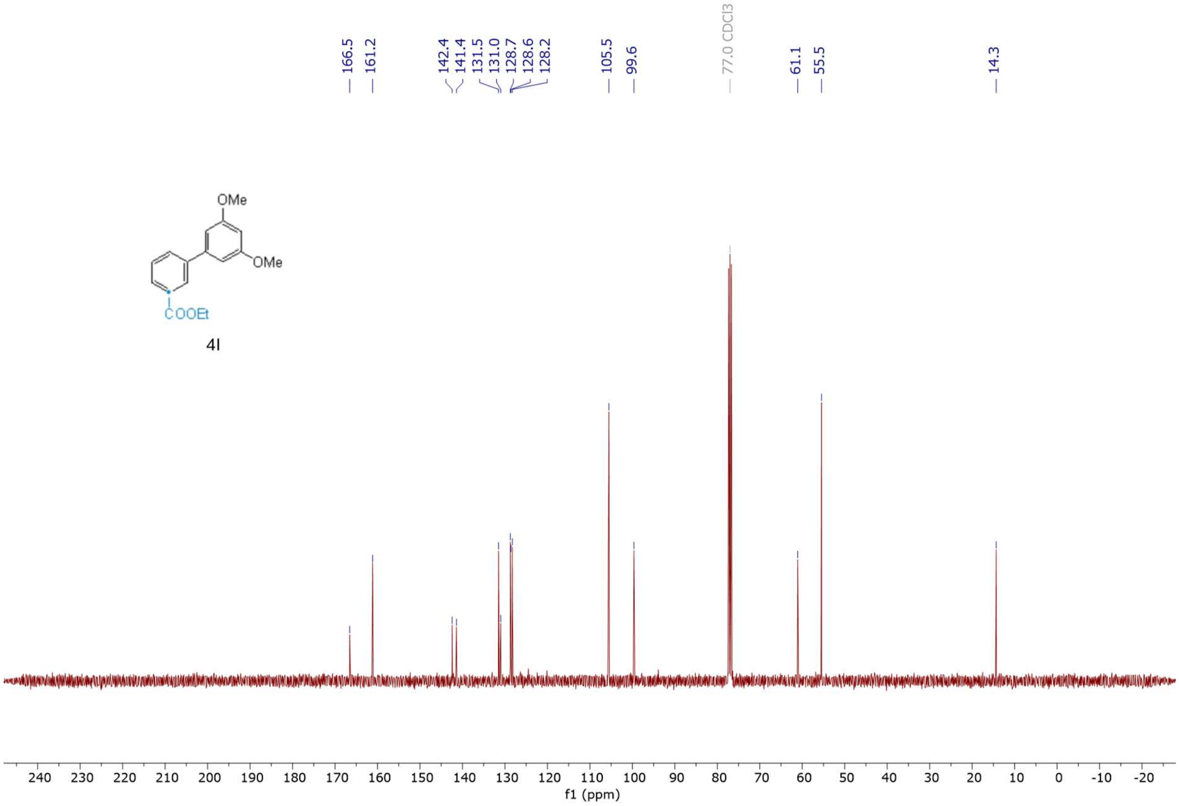

RAW Data/wfp-wb-503-4-proton-CDCl3 — glo wfp wb 503 4 — proton CDCl3 /opt/topspin av1 14 — 400.35MHz

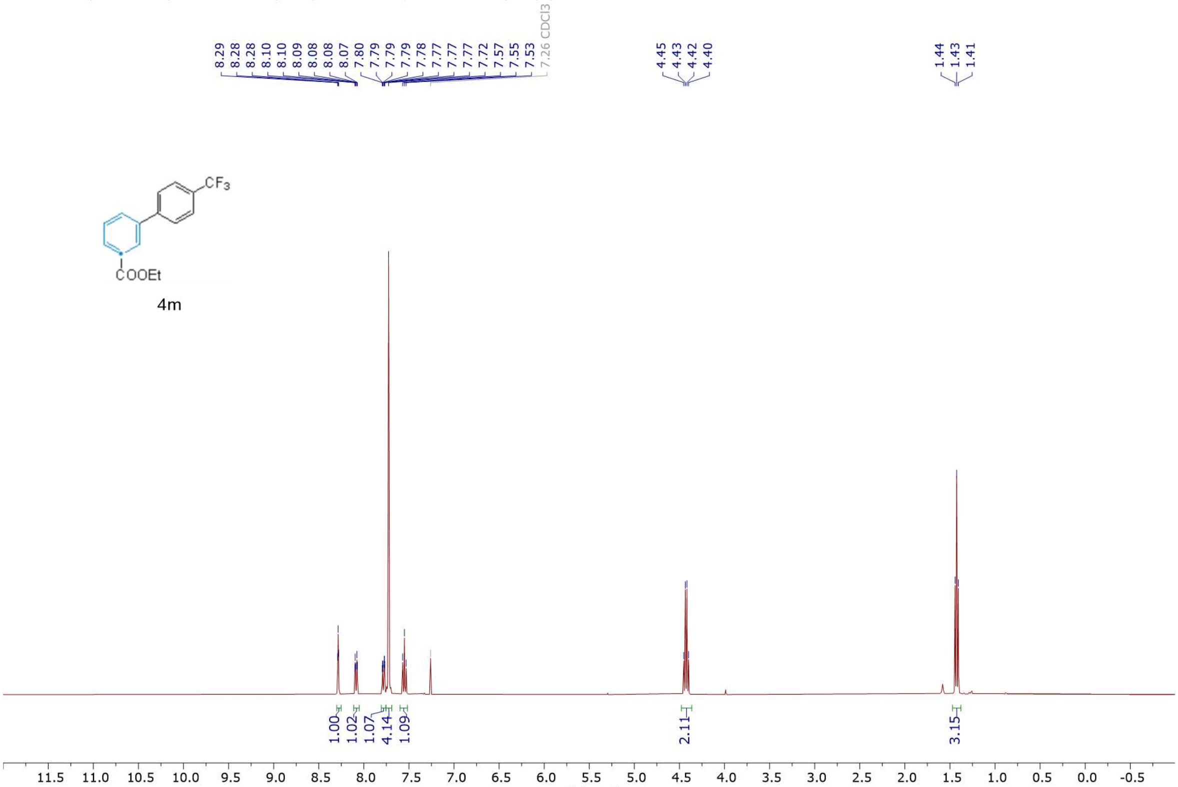

500/wfp-wb-503-4-carbon_256-CDCl3 — glo wfp wb 503 4 — carbon_256 CDCl3 /opt/topspin av1 14 — 100.68MHz

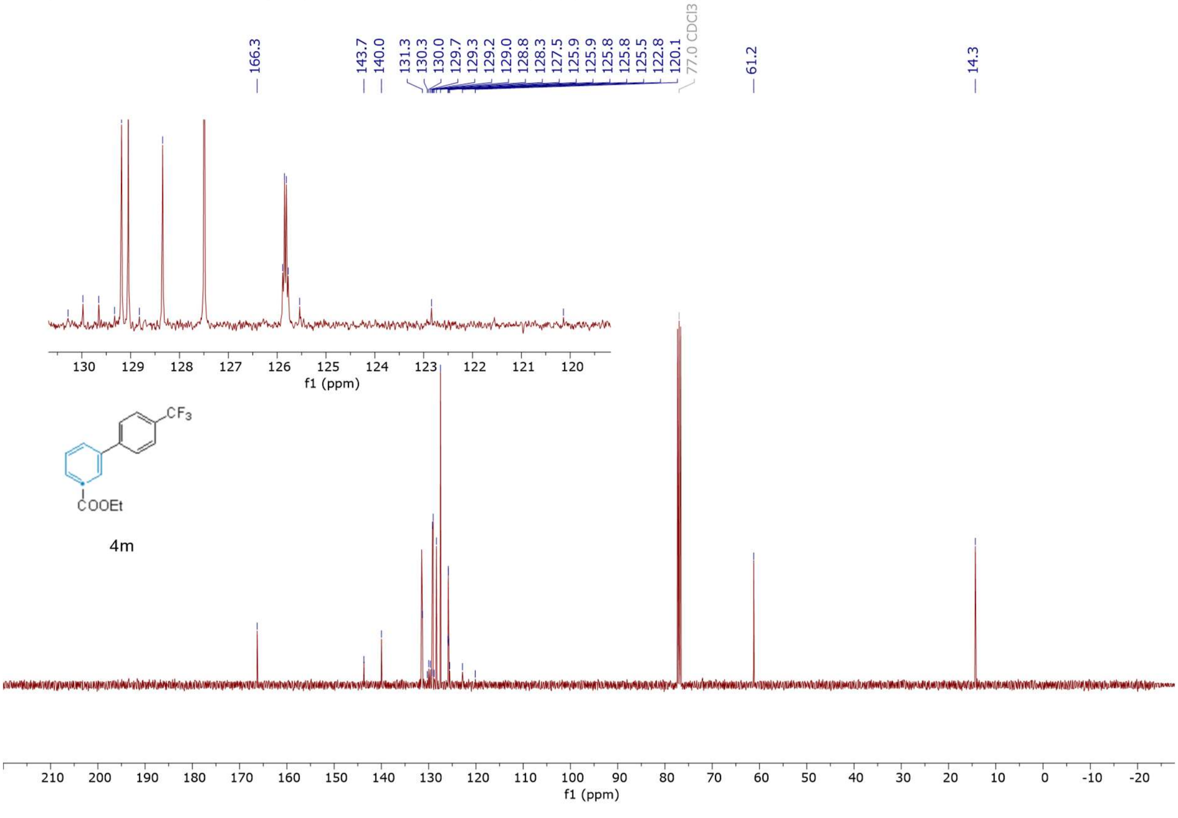

508/wfp-wb-507-2-proton-CDCl3 — glo wfp wb 507 2 — proton CDCl3 /opt/topspin av1 12 — 400.35MHz

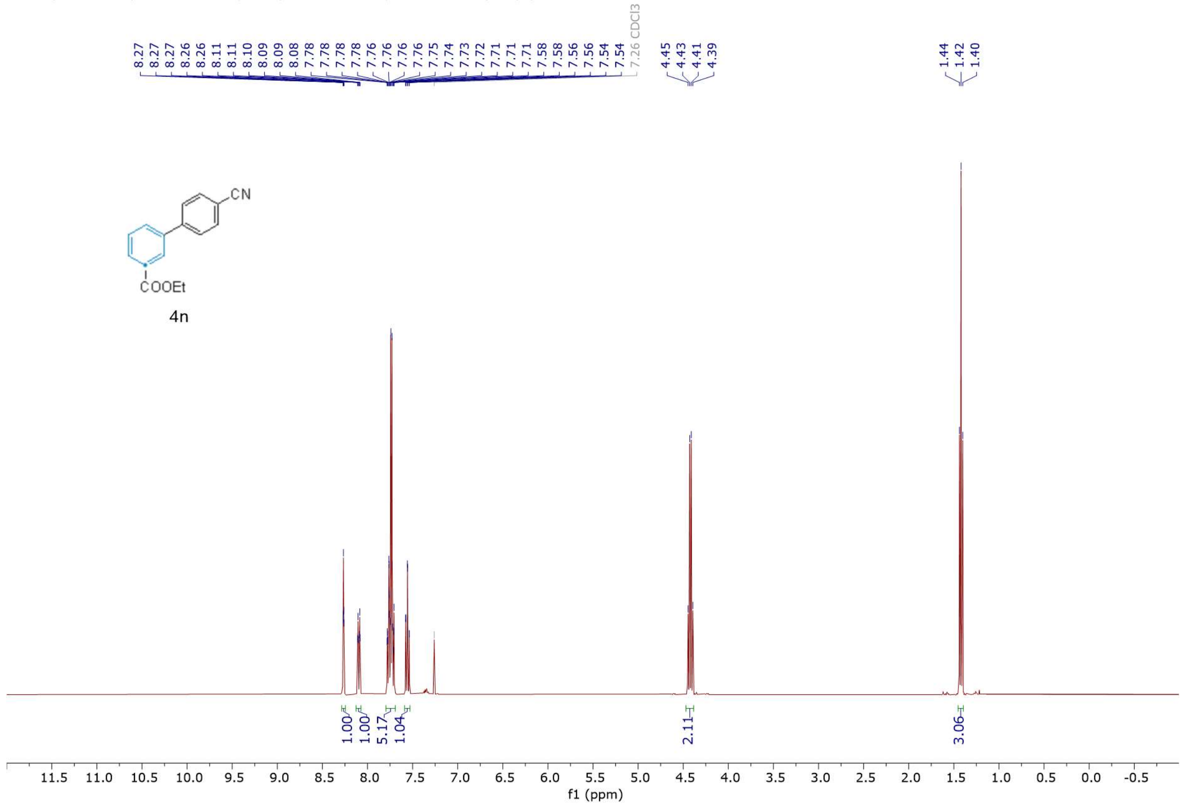

508/wfp-wb-507-2-carbon_256-CDCl3 — glo wfp wb 507 2 — carbon_256 CDCl3 /opt/topspin av1 12 — 100.68MHz

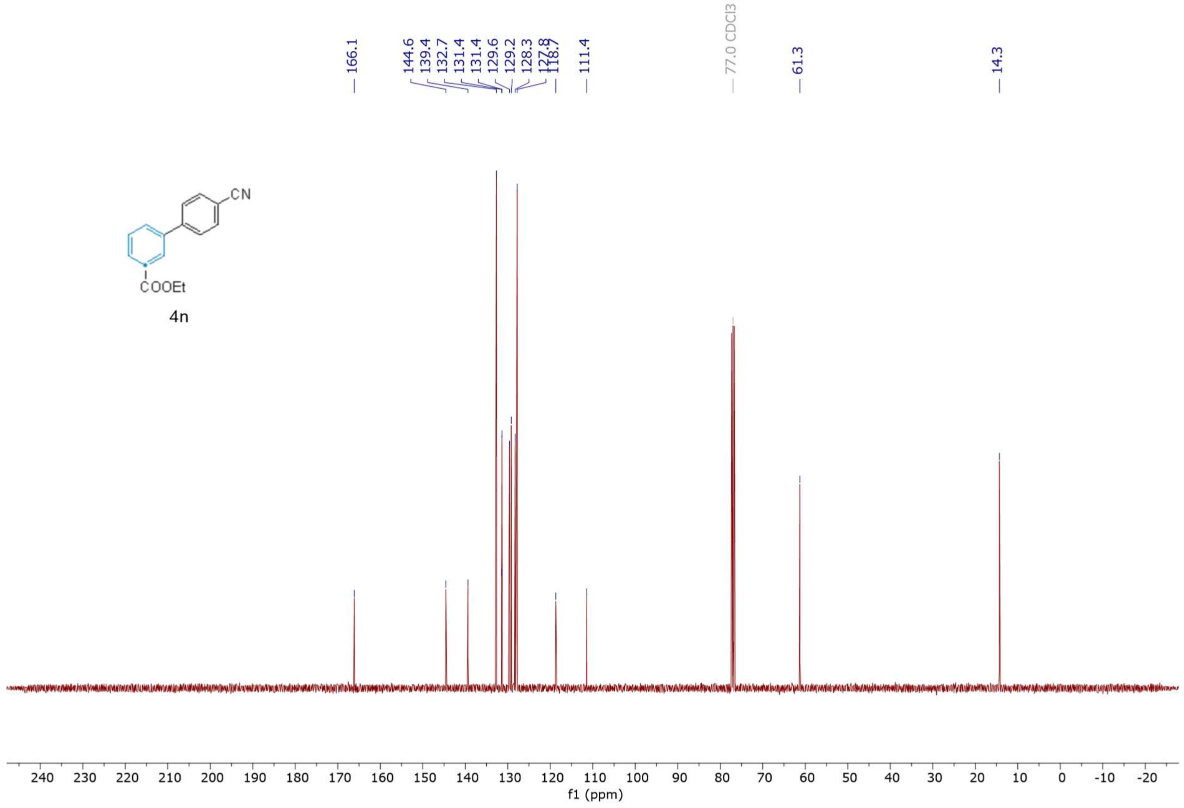

RAW Data/wfp-wb-501-3-proton-CDCl3 — glo wfp wb 501 3 — proton CDCl3 /opt/topspin av1 23 — 400.13MHz

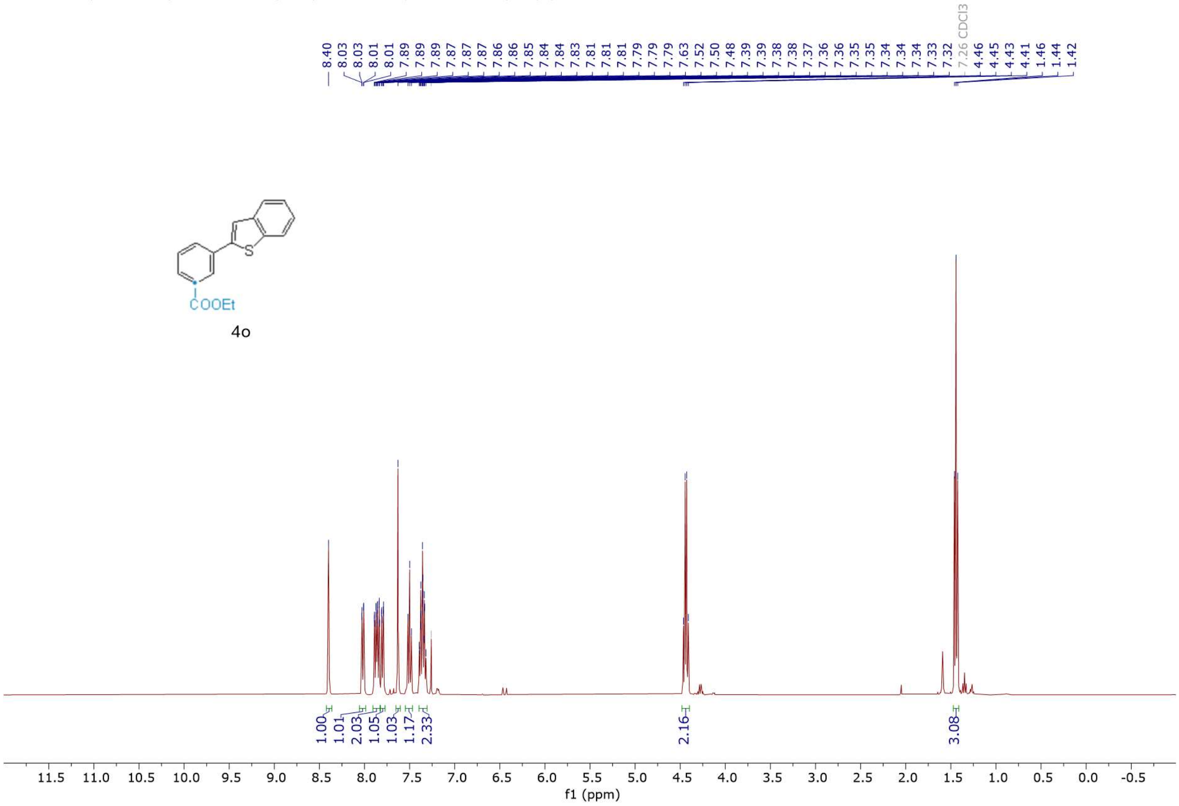

RAW Data/wfp-wb-501-3-carbon_256-CDCl3 — glo wfp wb 501 3 — carbon_256 CDCl3 /opt/topspin av1 23 — 100.62MHz

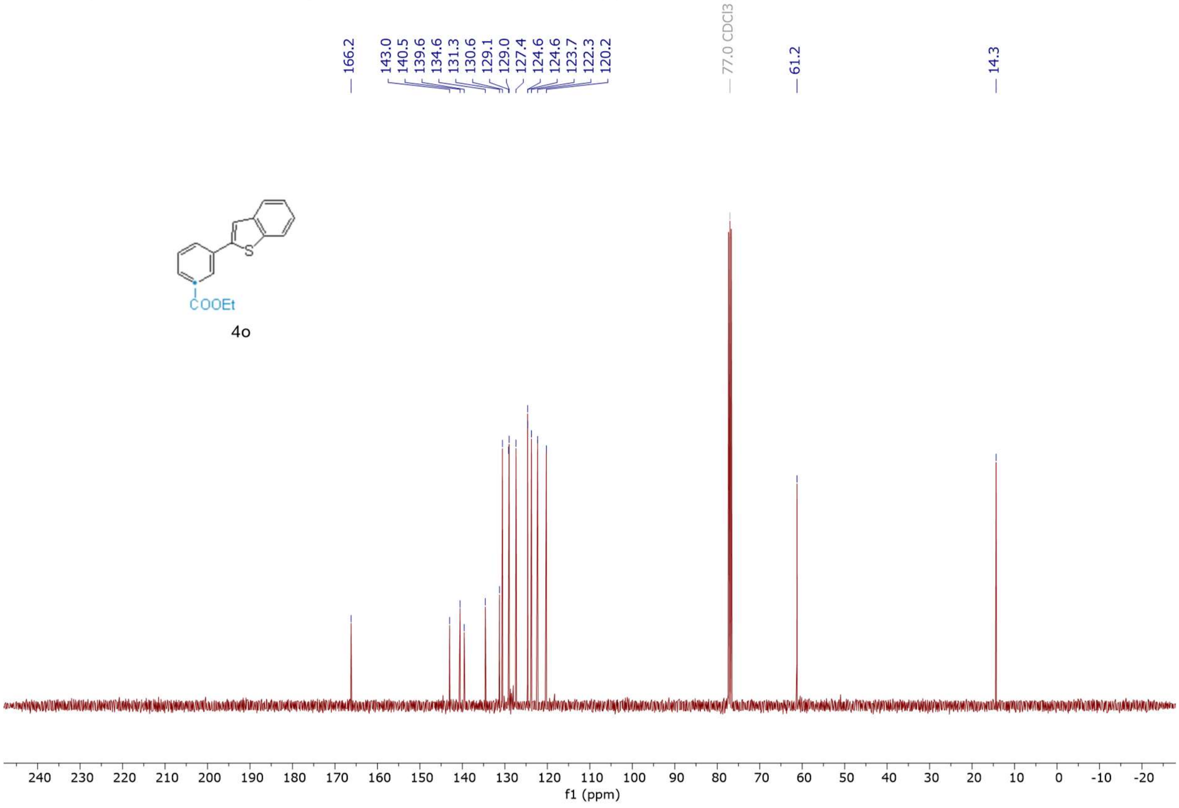

RAW Data/wfp-wb-523-proton-CDCl3 — glo wfp wb 523 — proton CDCl3 /opt/topspin av1 14 — 400.35MHz

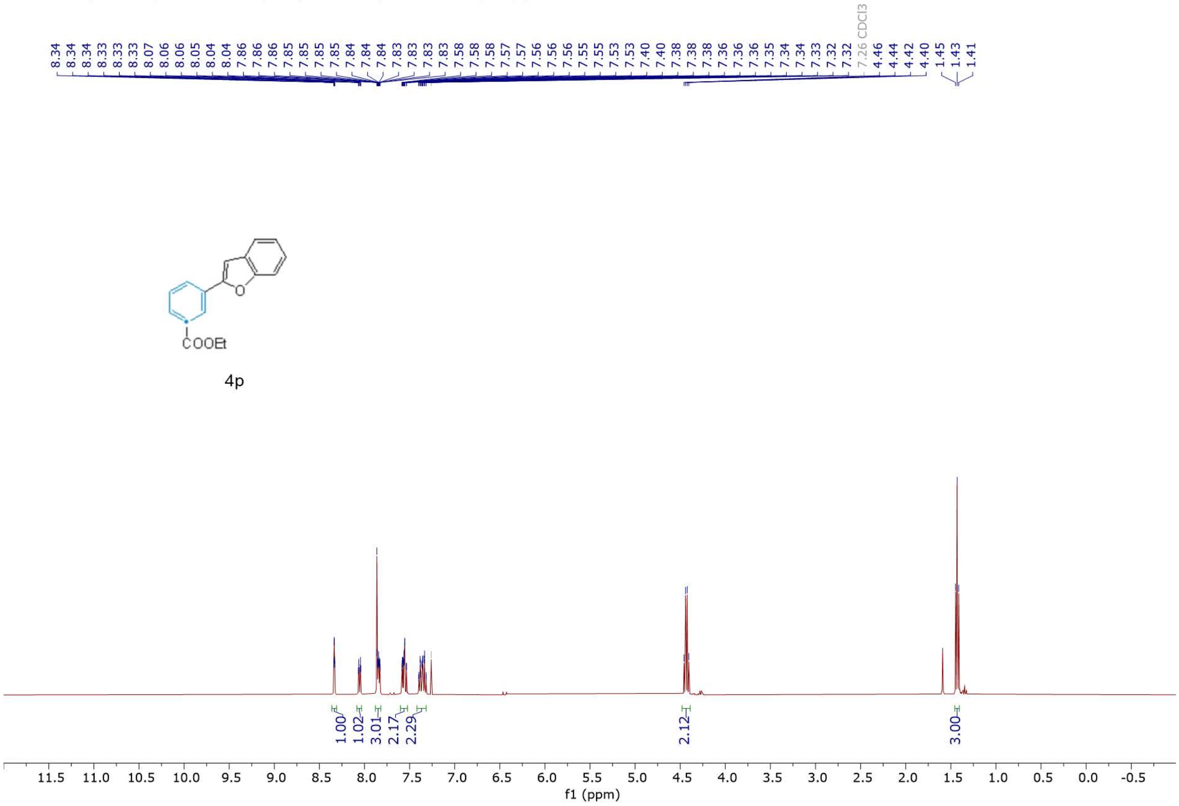

RAW Data/wfp-wb-523-carbon_256-CDCl3 — glo wfp wb 523 — carbon_256 CDCl3 /opt/topspin av1 14 — 100.68MHz

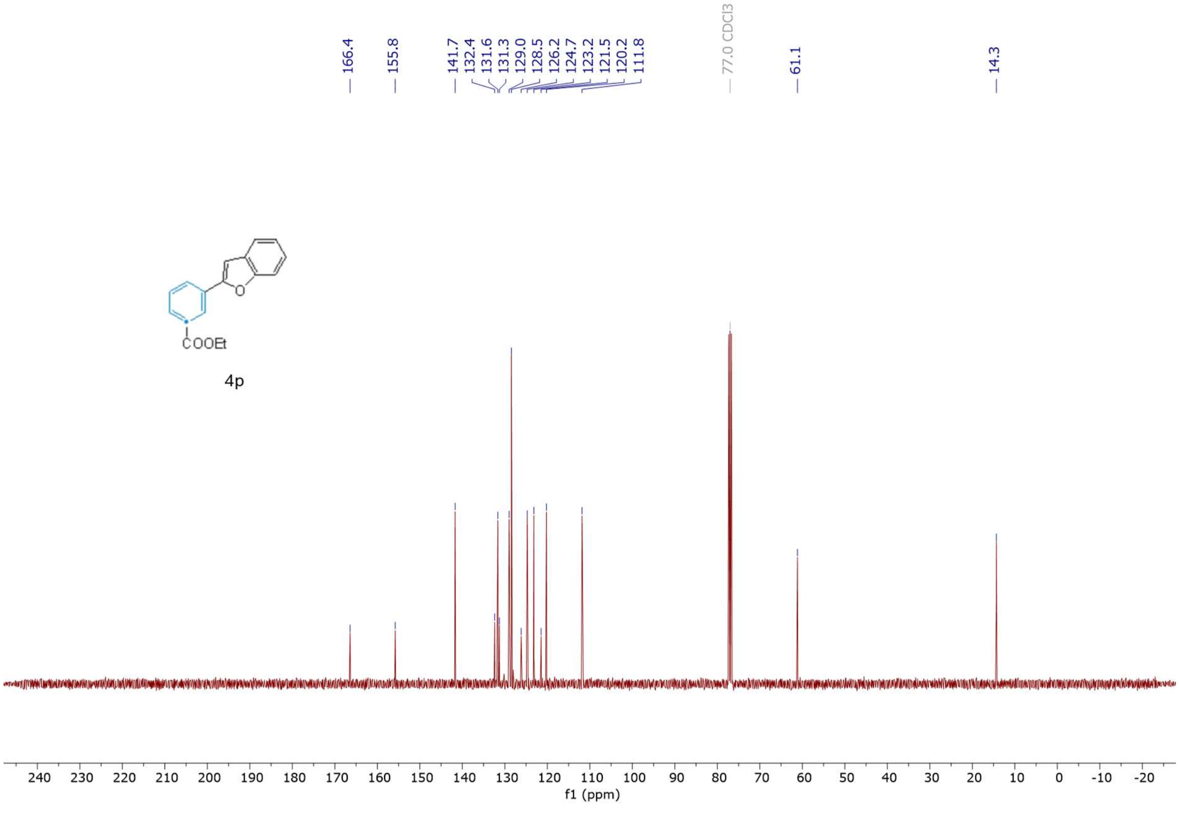

RAW Data/wfp-wb-522-proton-CDCl3 — glo wfp wb 522 — proton CDCl3 /opt/topspin av1 13 — 400.35MHz

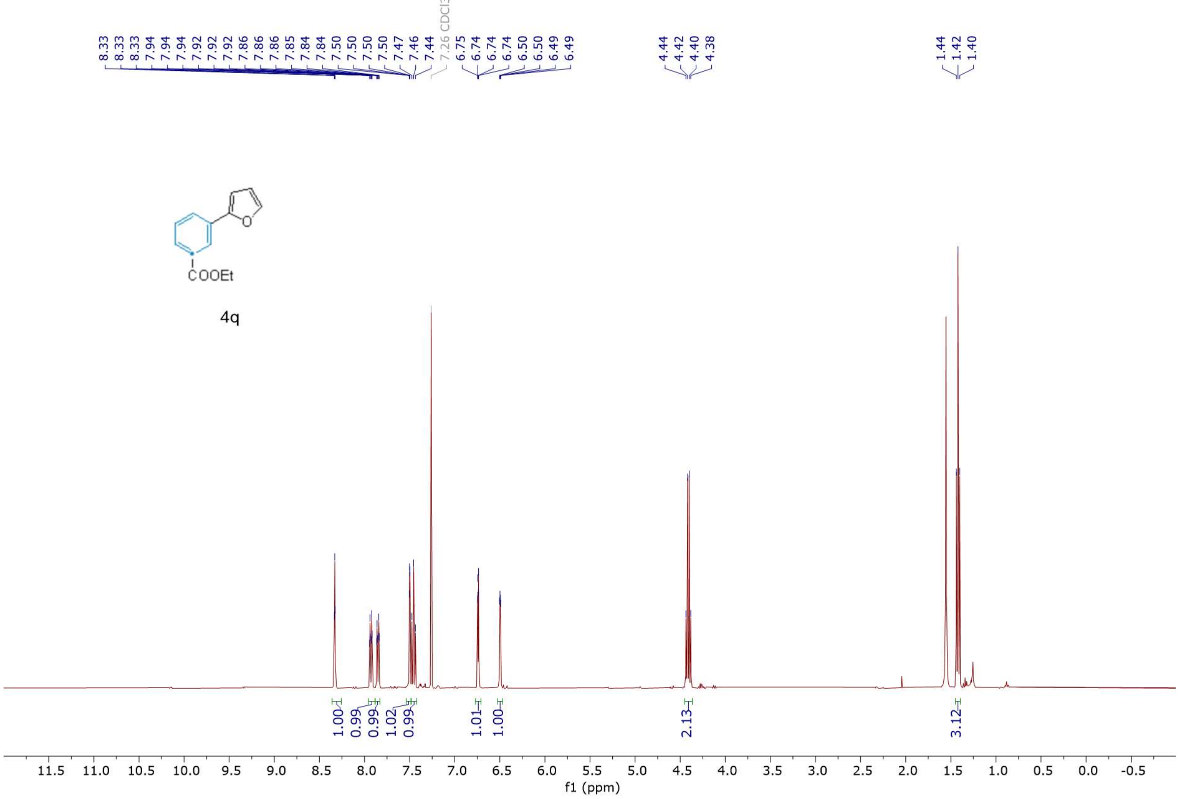

RAW Data/wfp-wb-522-carbon_256-CDCl3 — glo wfp wb 522 — carbon_256 CDCl3 /opt/topspin av1 13 — 100.68MHz

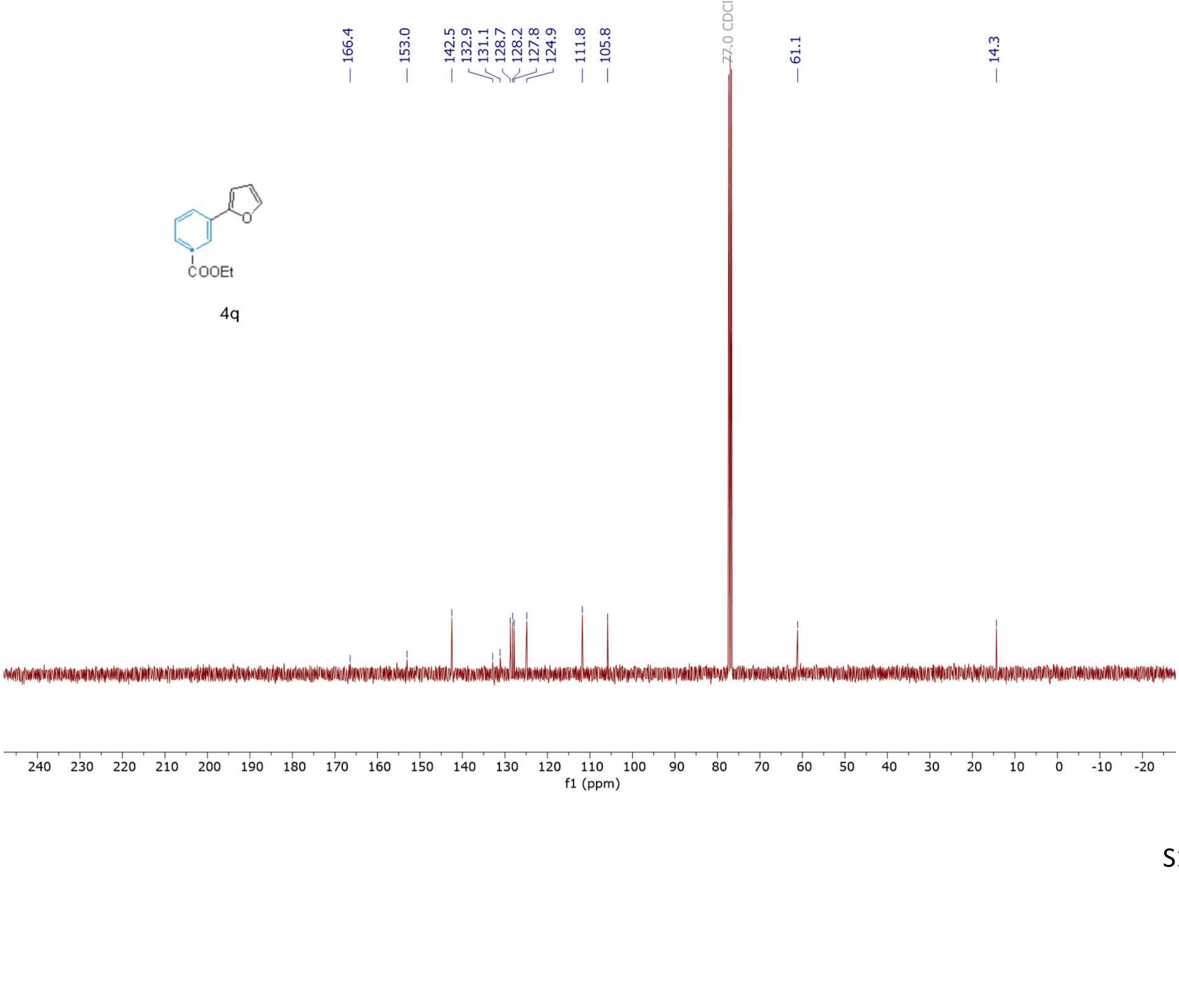

521/wfp-wb-521-proton-CDCl3 — glo wfp wb 521 — proton CDCl3 /opt/topspin av1 12 — 400.35MHz

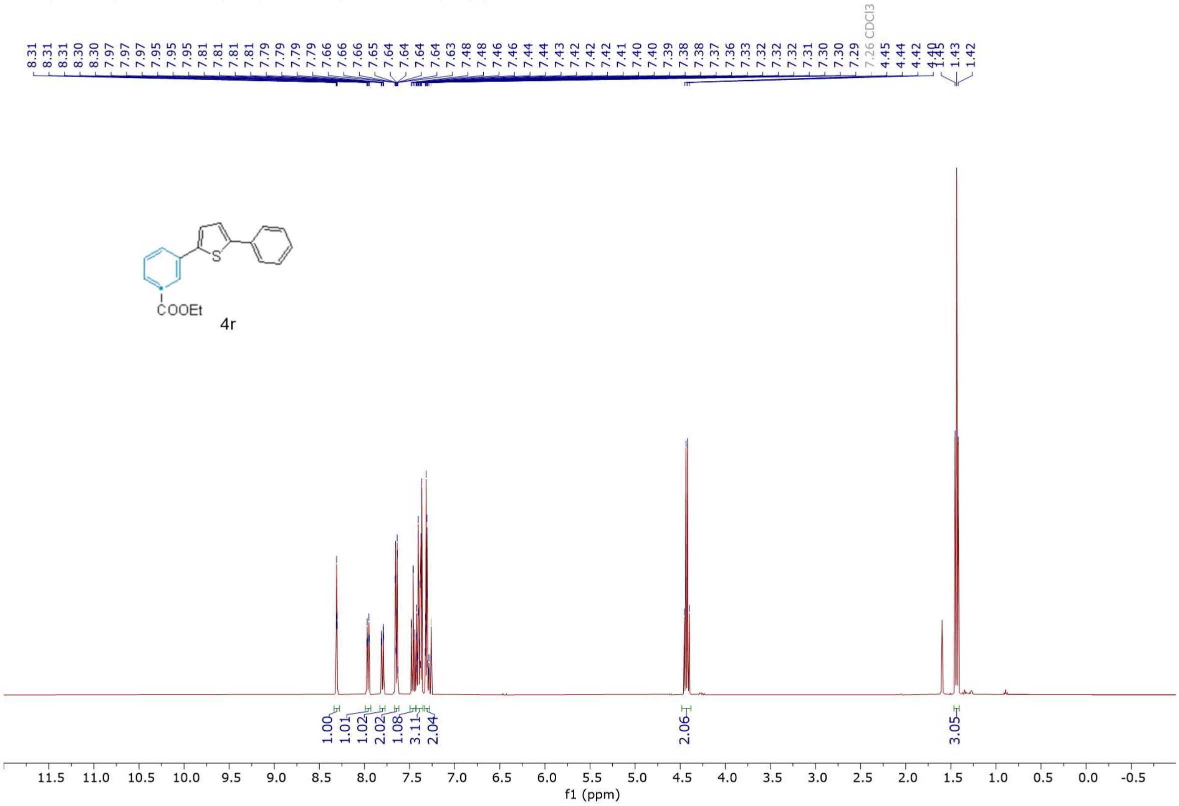

521/wfp-wb-521-carbon_256-CDCl3 — glo wfp wb 521 — carbon_256 CDCl3 /opt/topspin av1 12 — 100.68MHz

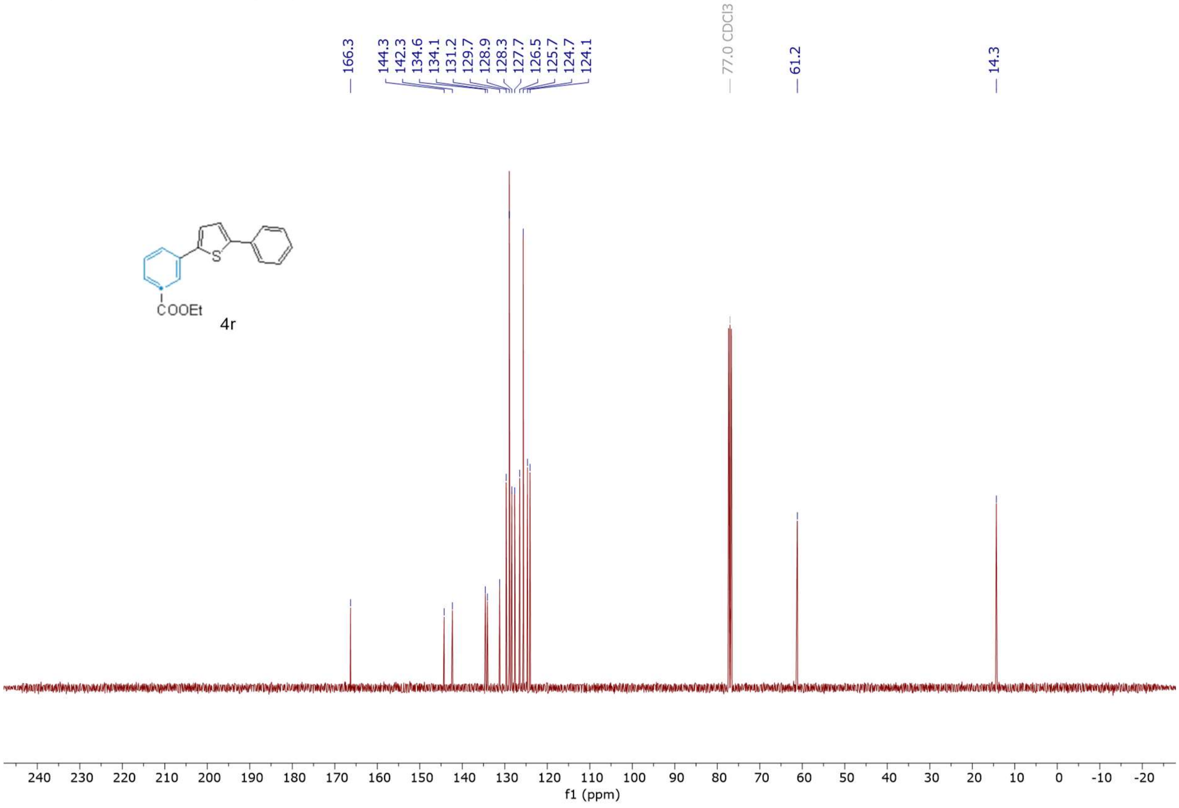

RAW Data/wfp-wb-635-3-proton-CDCl3 — glo wfp wb 635-3 — proton CDCl3 /opt/topspin av1 32 — 400.23MHz

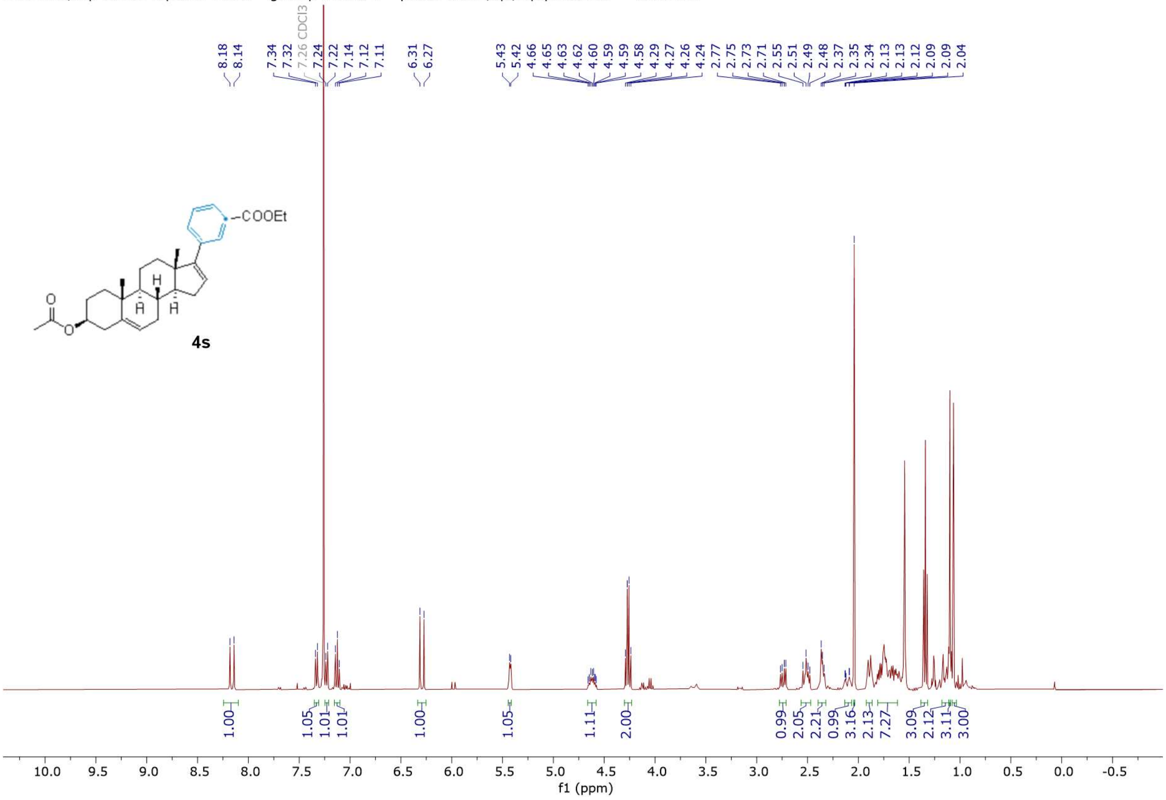

RAW Data/wfp-wb-635-carbon_5120-CDCl3 — glo wfp wb 635 — carbon_5120 CDCl3 /opt/topspin av1 20 — 100.68MHz

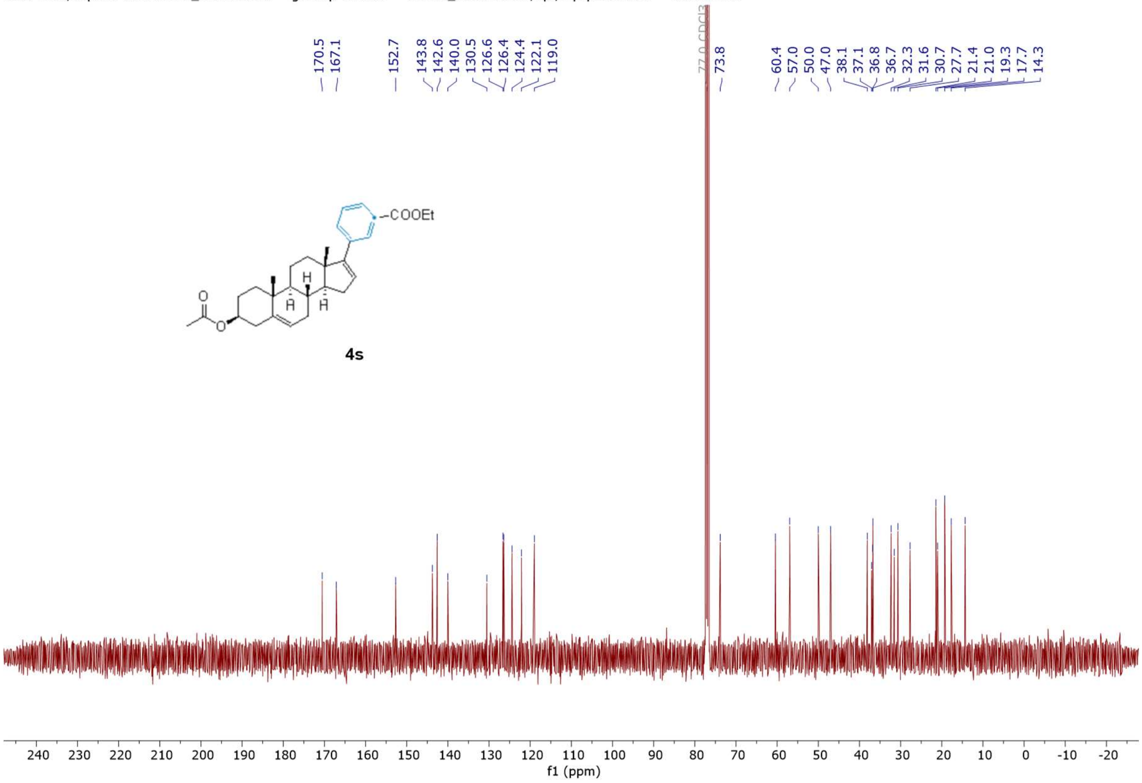

RAW Data/wfp-wb-634-proton-CDCl3 — glo wfp wb 634 — proton CDCl3 /opt/topspin av1 3 — 400.23MHz

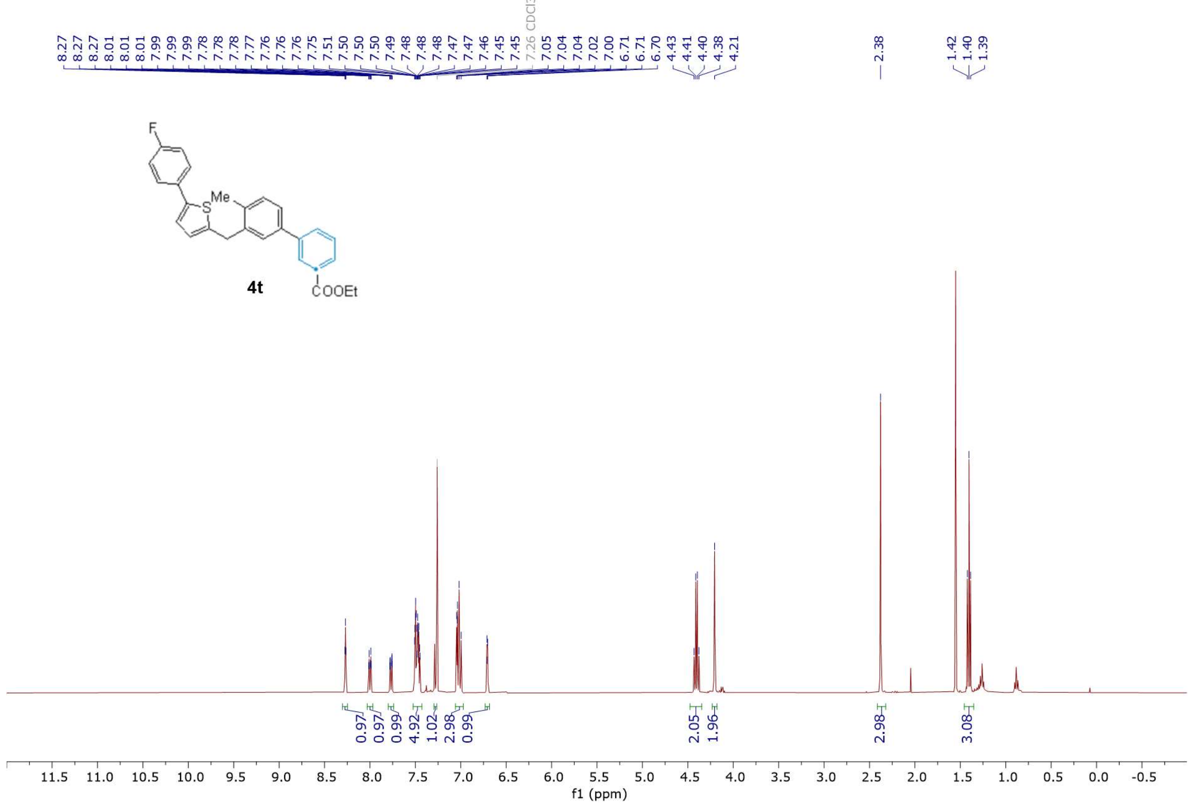

RAW Data/wfp-wb-634-carbon_5120-CDCl3 — glo wfp wb 634 — carbon_5120 CDCl3 /opt/topspin av1 19 — 100.68MHz

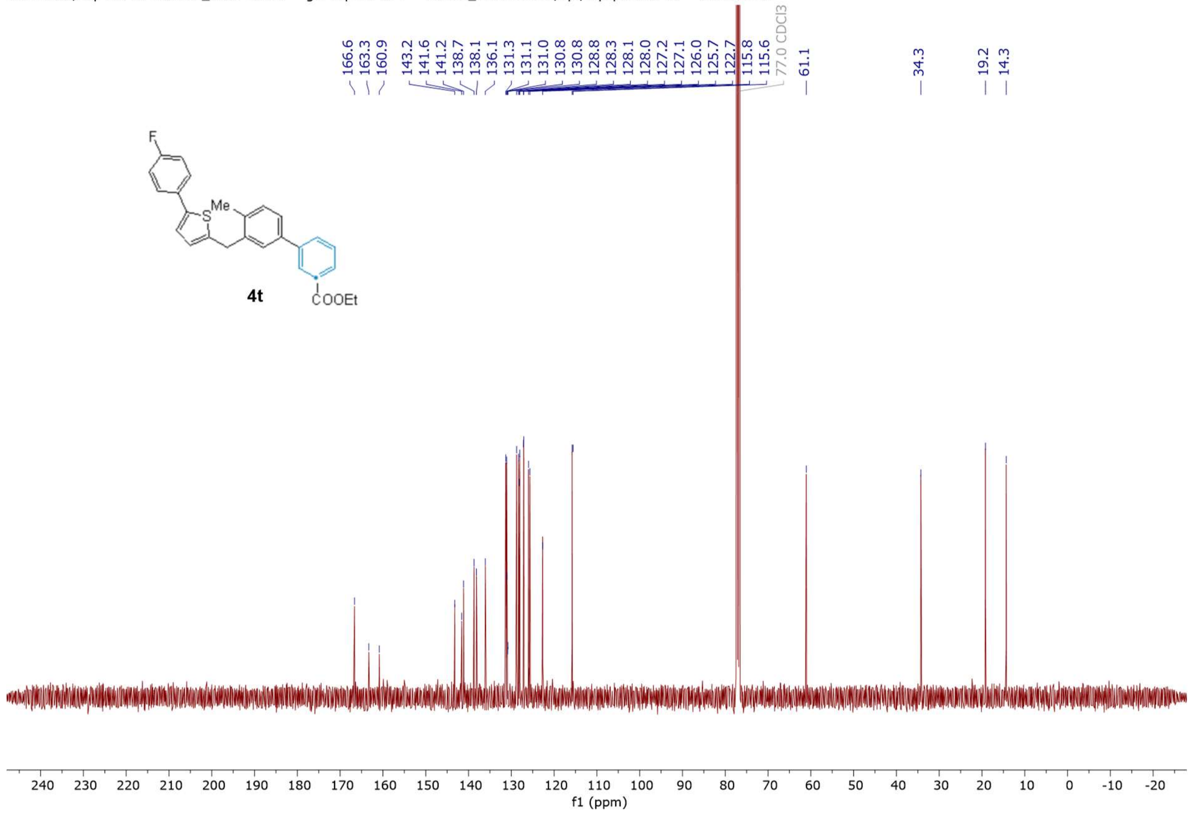

516/wfp-wb-516-proton-CDCl3 — glo wfp wb 513 3 — proton CDCl3 /opt/topspin av1 3 — 400.35MHz

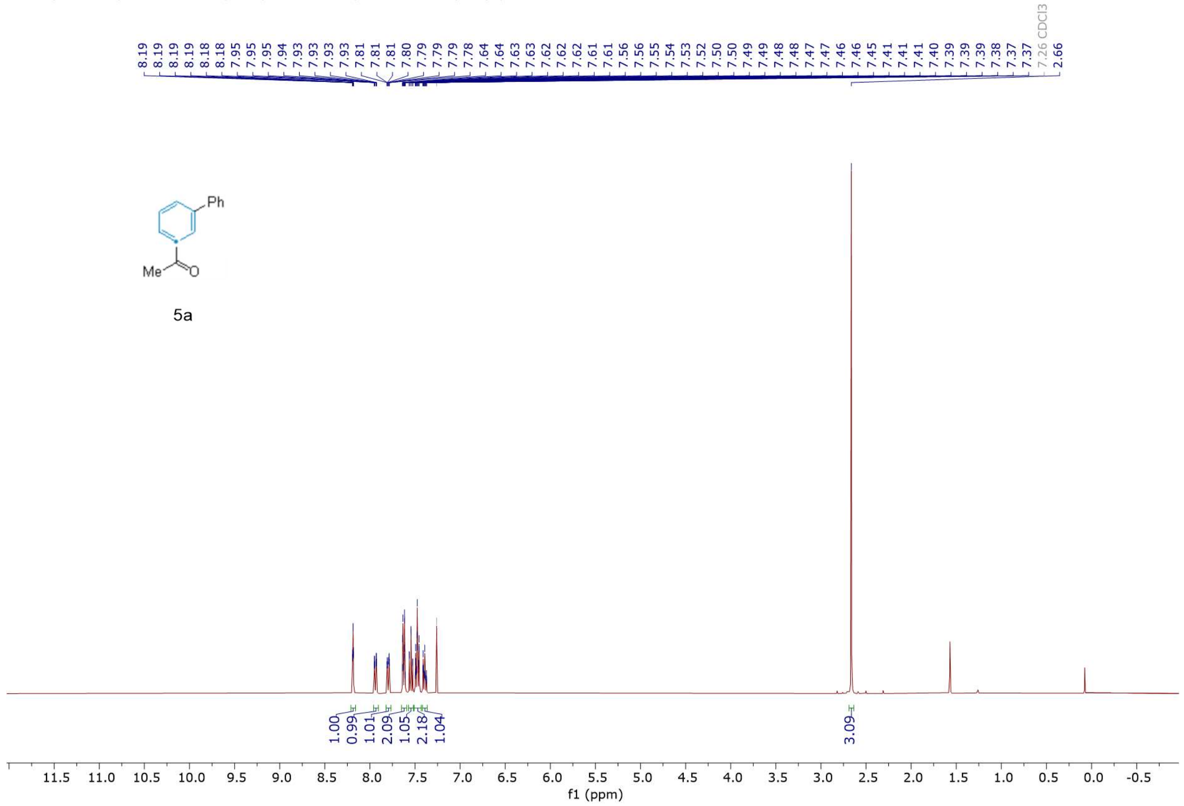

516/wfp-wb-516-carbon_256-CDCl3 — glo wfp wb 513 3 — carbon_256 CDCl3 /opt/topspin av1 3 — 100.68MHz

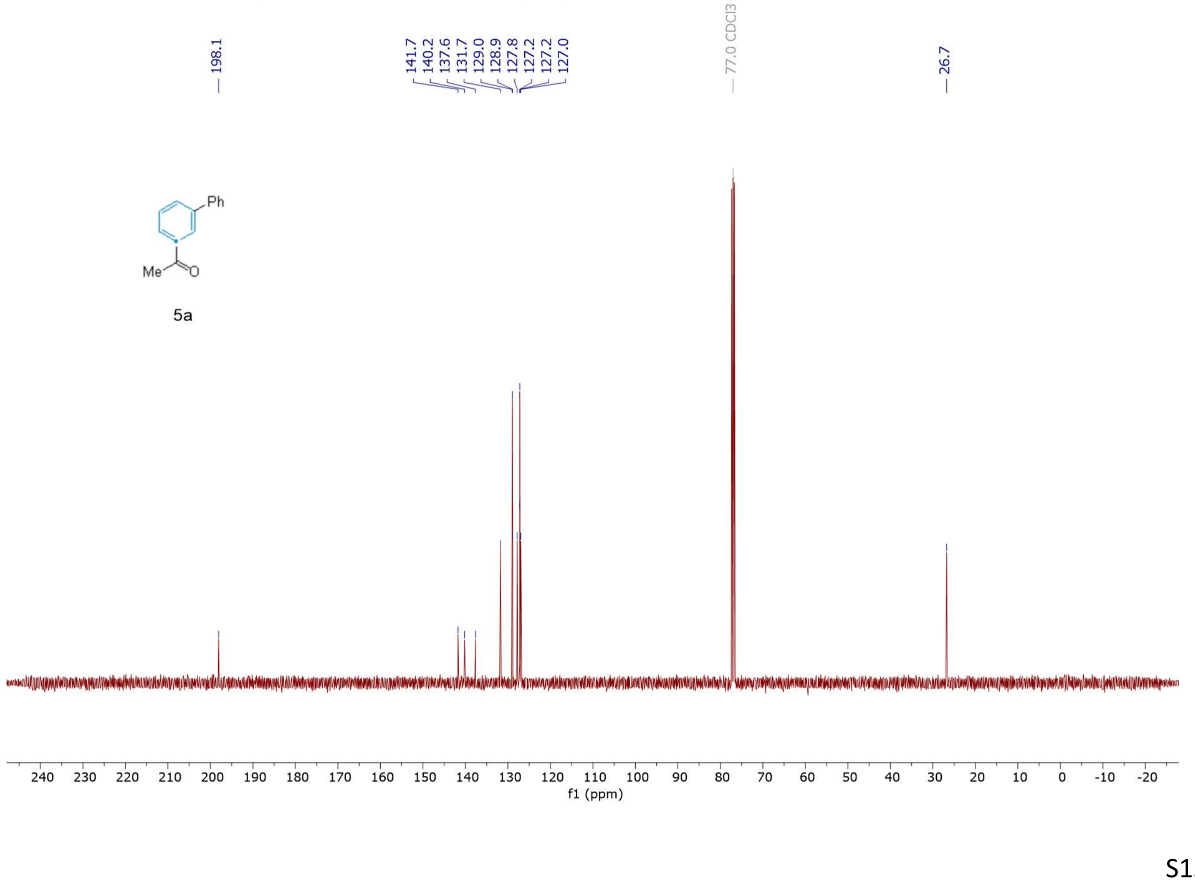

519/wfp-wb-519-proton-CDCl3 — glo wfp wb 519 — proton CDCl3 /opt/topspin av1 15 — 400.35MHz

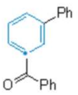

5b

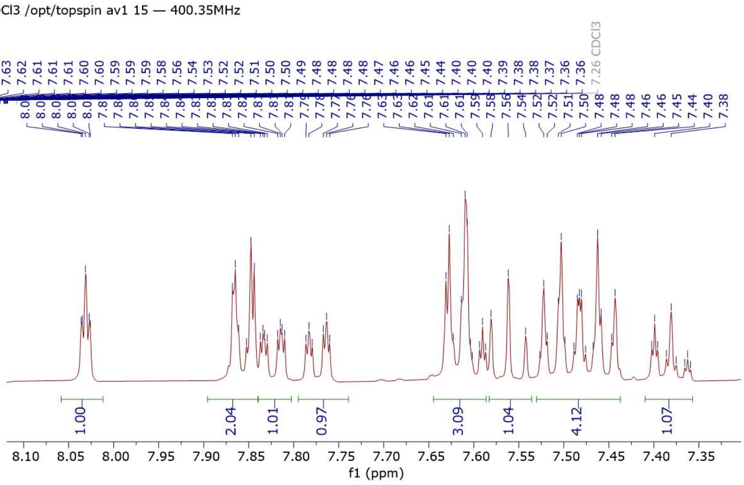

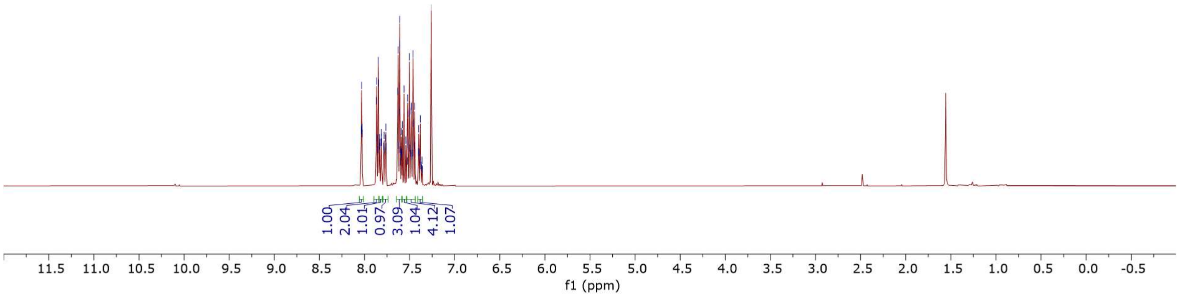

519/wfp-wb-519-carbon-CDCl3 — glo wfp wb 519 — carbon CDCl3 /opt/topspin av1 15 — 100.68MHz

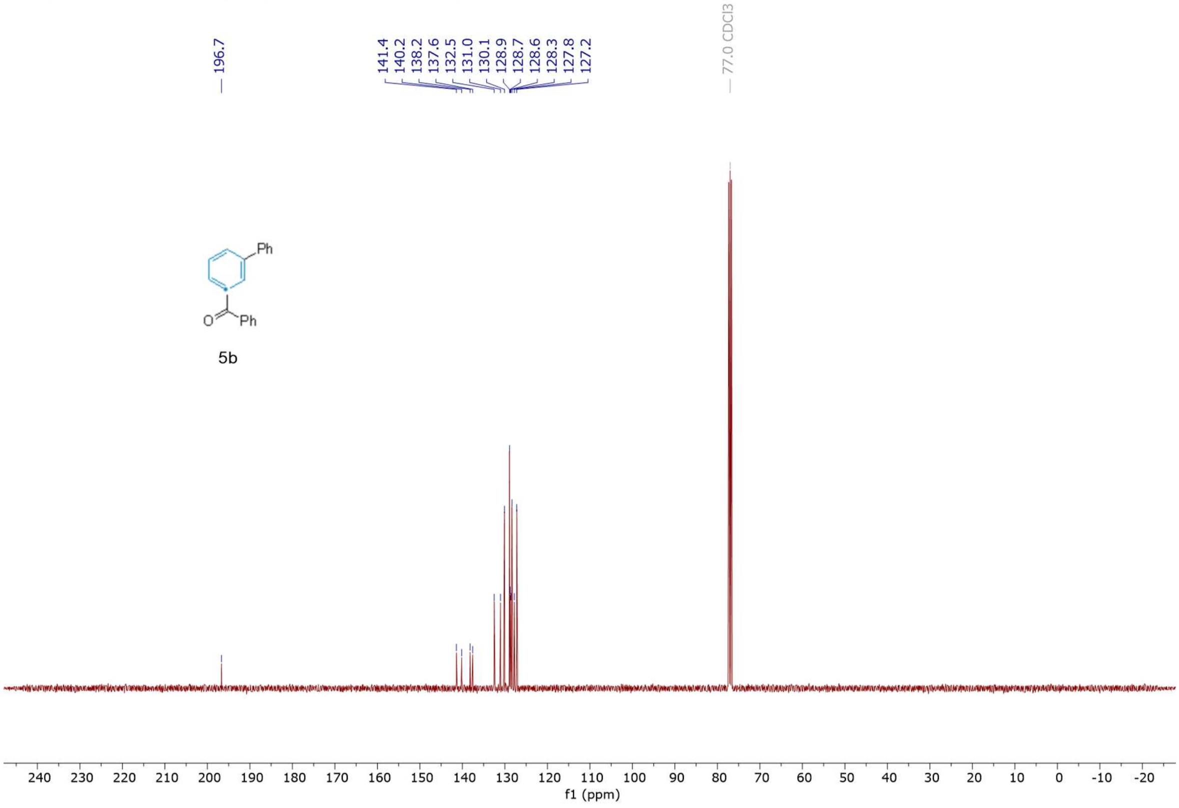

5b

517/wfp-wb-517-proton-CDCl3 — glo wfp wb 513 4 — proton CDCl3 /opt/topspin av1 4 — 400.35MHz

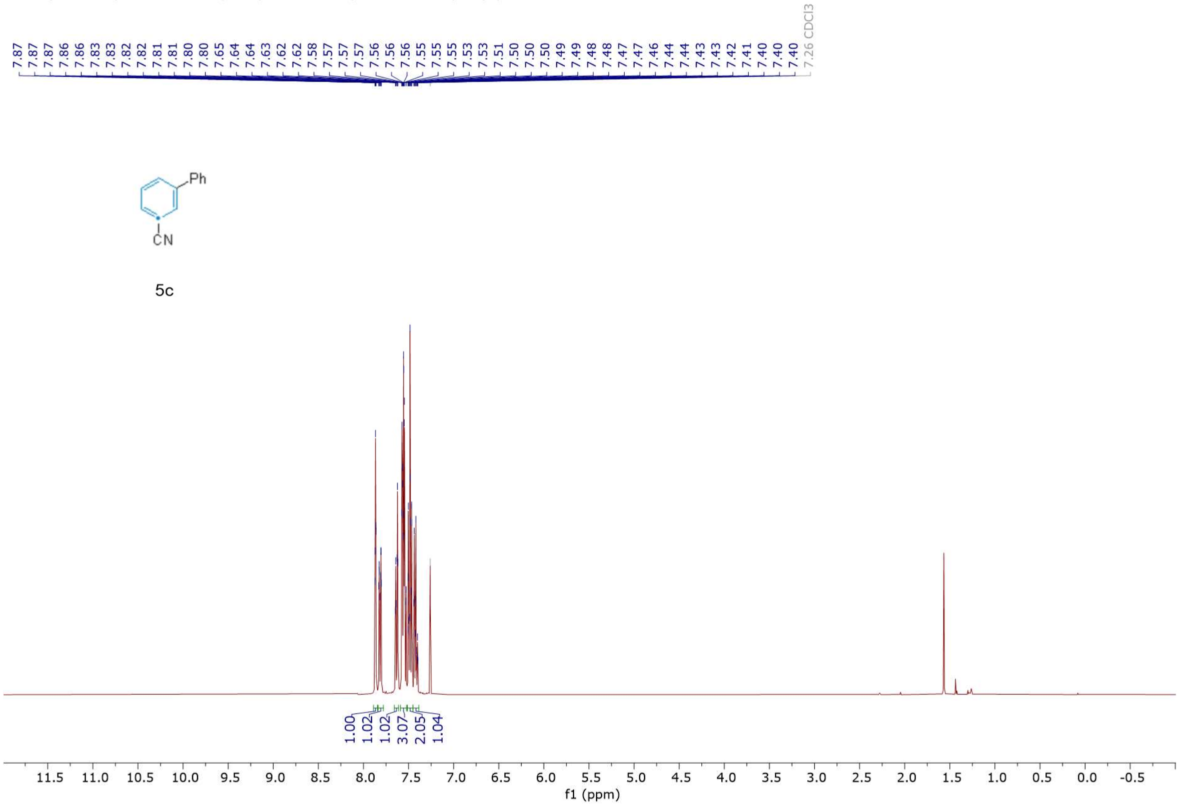

517/wfp-wb-517-carbon_256-CDCl3 — glo wfp wb 513 4 — carbon_256 CDCl3 /opt/topspin av1 4 — 100.68MHz

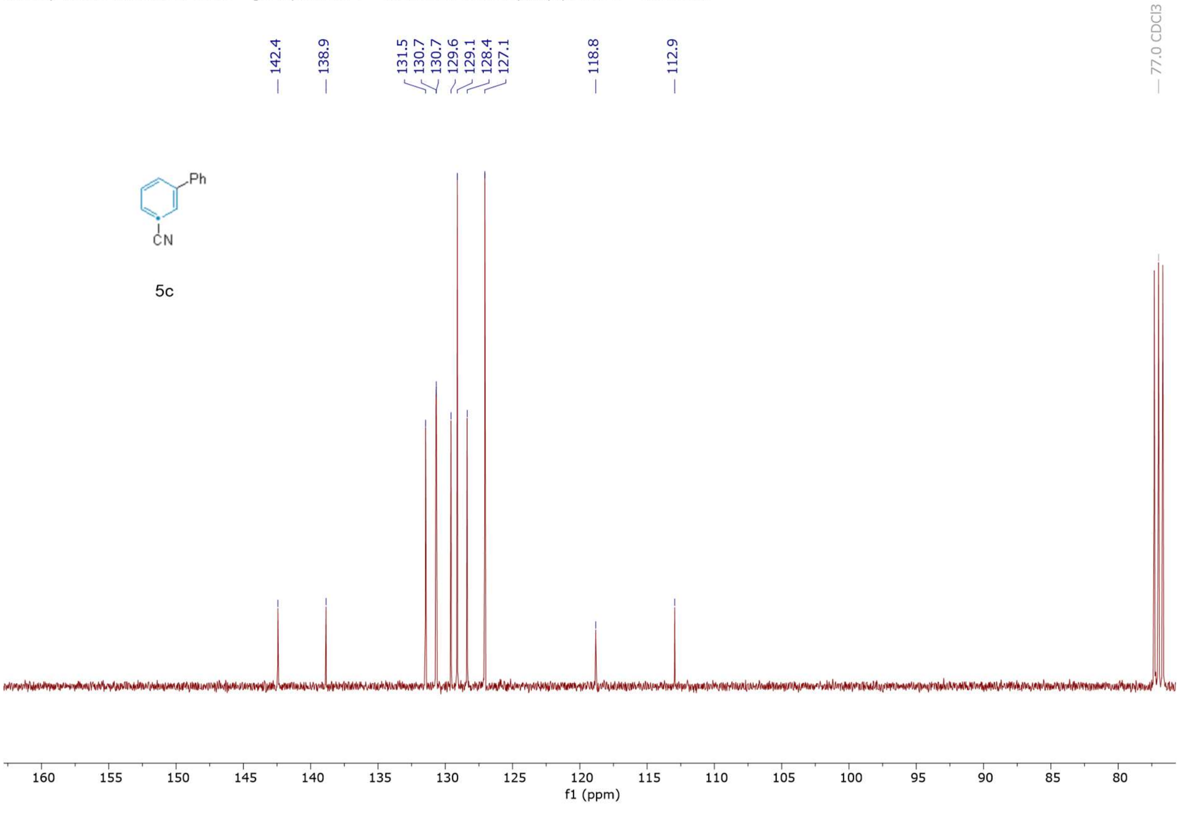

518/wfp-wb-518-proton-CDCl3 — glo wfp wb 518 — proton CDCl3 /opt/topspin av1 2 — 400.35MHz

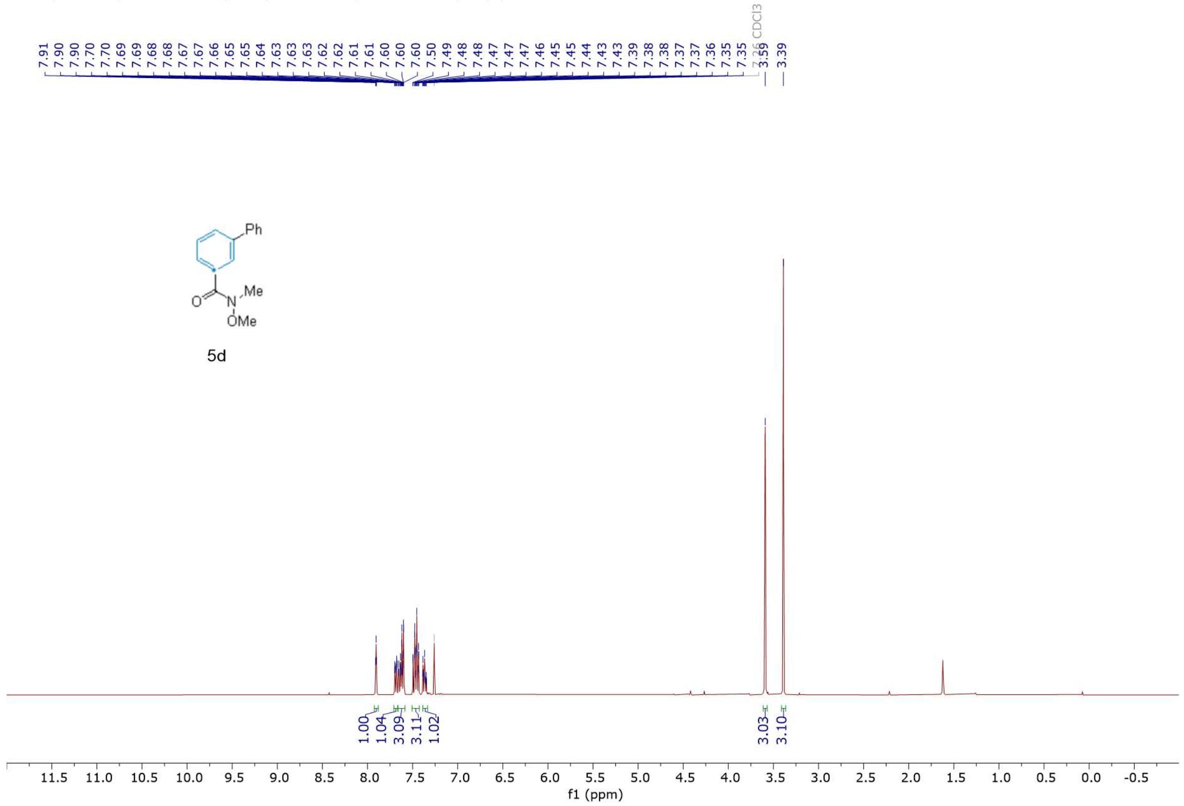

518/wfp-wb-518-carbon-CDCl3 — glo wfp wb 518 — carbon CDCl3 /opt/topspin av1 2 — 100.68MHz

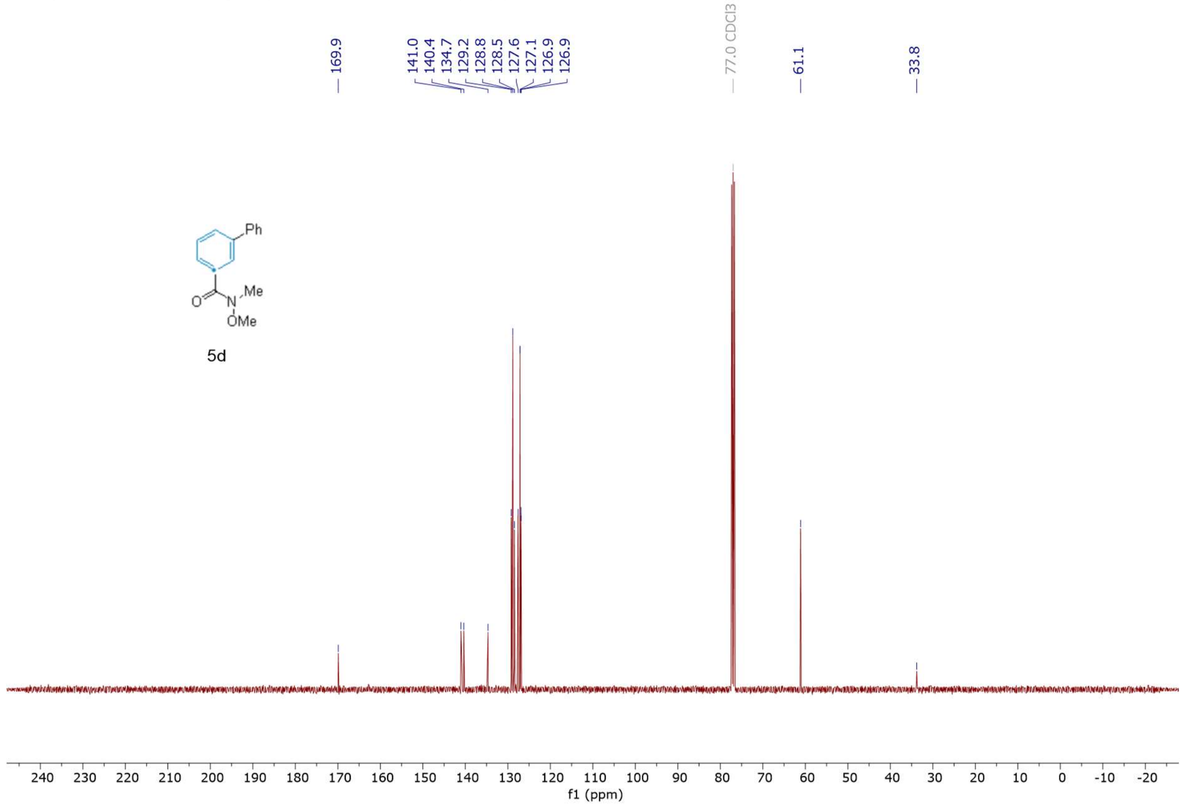

RAW Data/wfp-wb-520-proton-CDCl3-2 — glo wfp wb 520 — proton CDCl3 /opt/topspin av1 3 — 400.35MHz

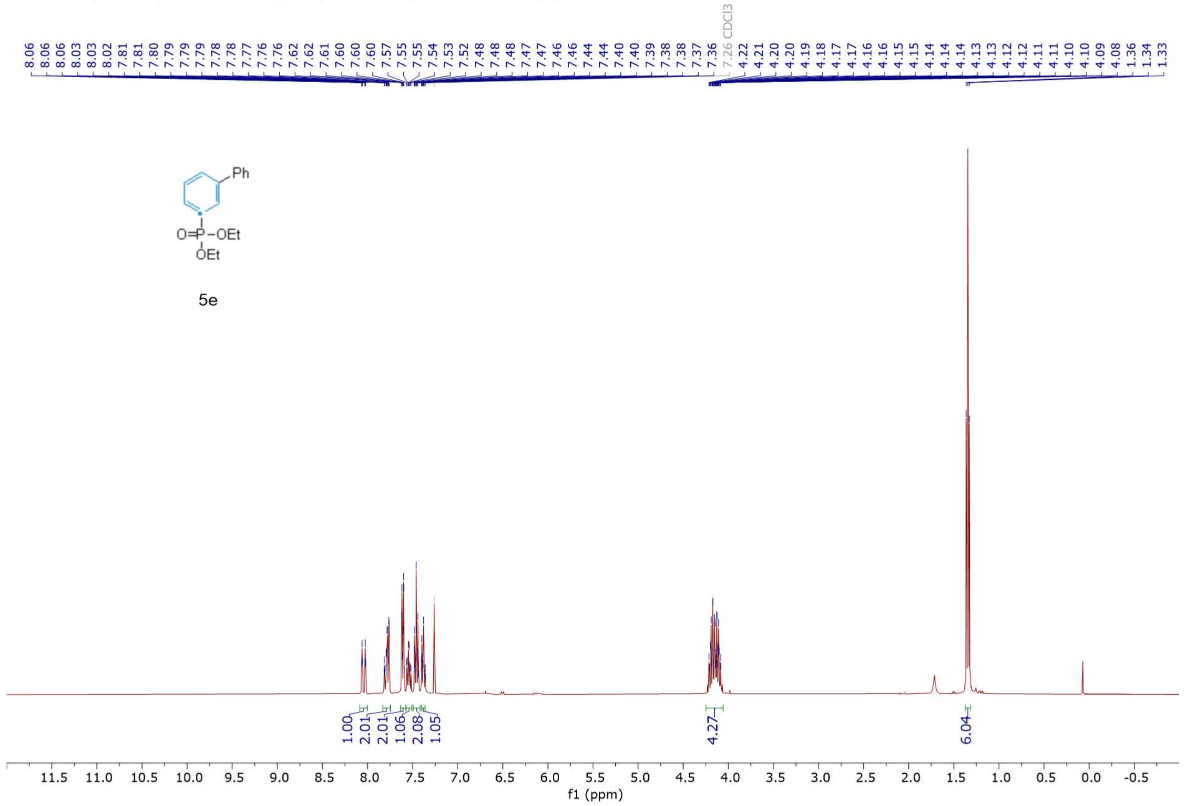

RAW Data/wfp-wb-520-carbon-CDCl3-2 — glo wfp wb 520 — carbon CDCl3 /opt/topspin av1 3 — 100.68MHz

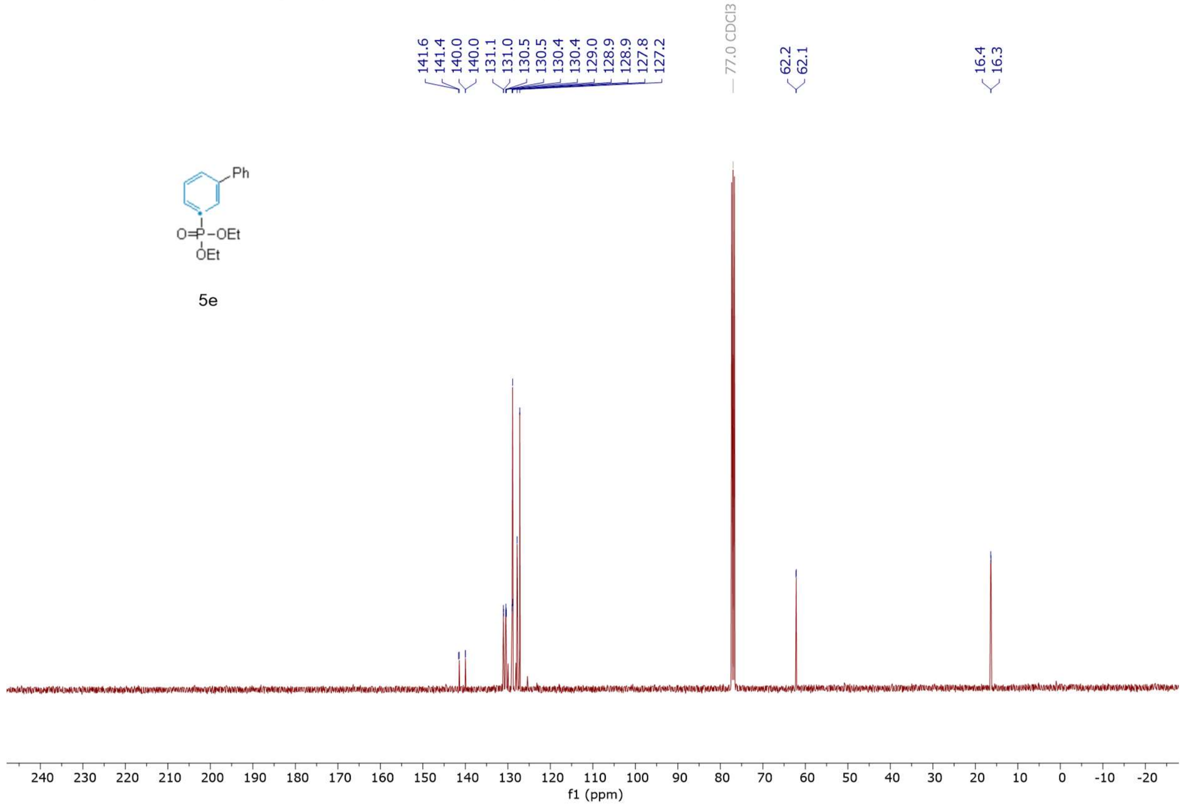

RAW Data/wfp-wb-520-p31cpd-CDCl3-2 — glo wfp wb 520 — p31cpd CDCl3 /opt/topspin av1 3 — 162.06MHz

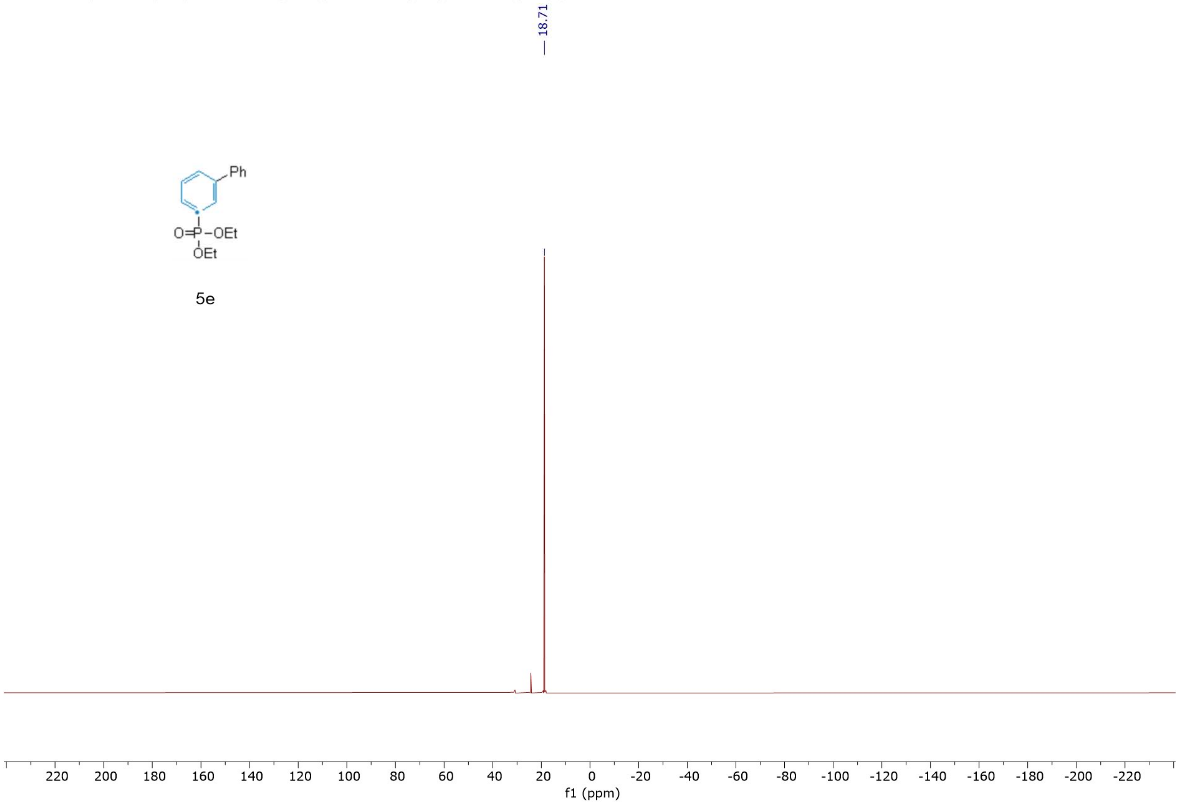

RAW Data/wfp-wb-528-proton-CDCl3 — glo wfp wb 528 — proton CDCl3 /opt/topspin av1 3 — 400.35MHz

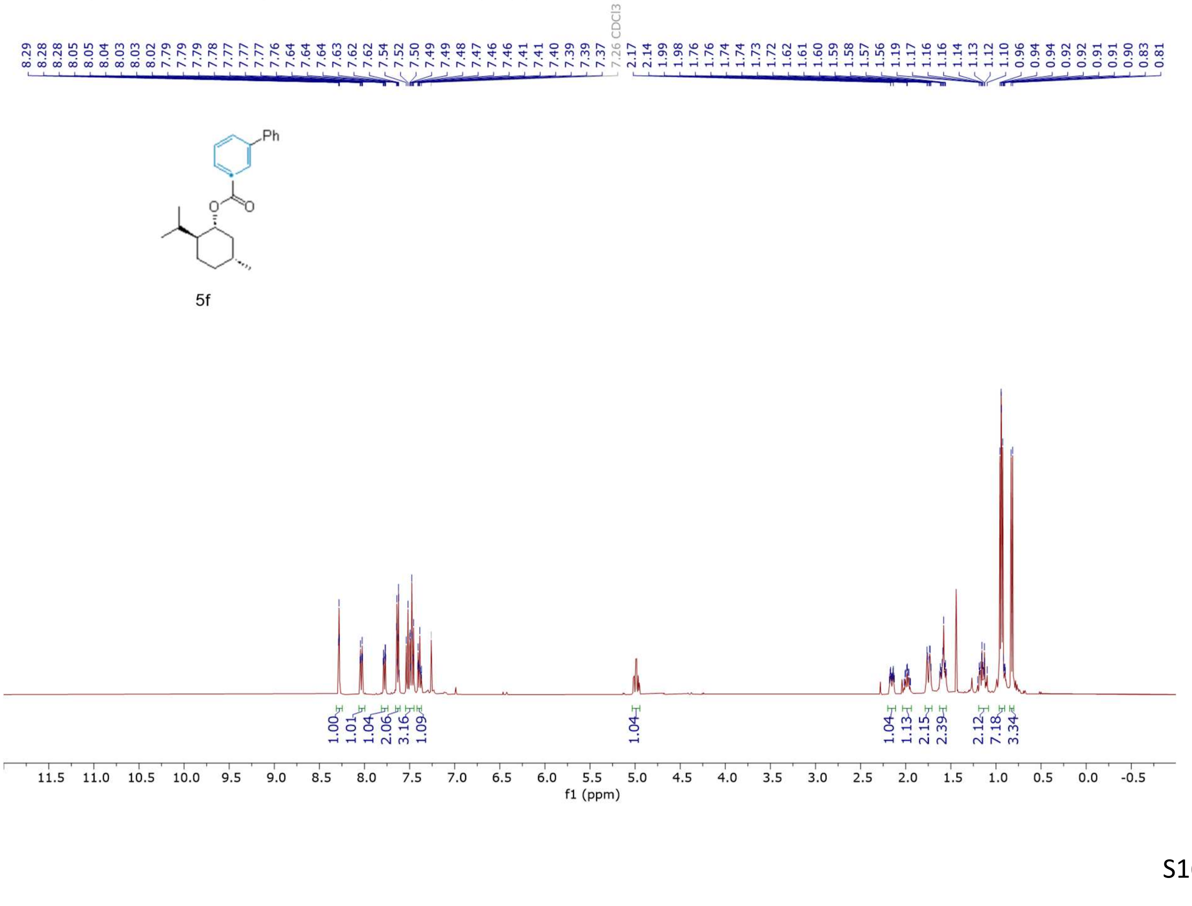

RAW Data/wfp-wb-528-proton-CDCl3 — glo wfp wb 528 — proton CDCl3 /opt/topspin av1 3 — 400.35MHz

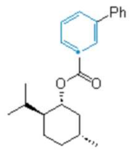

5f

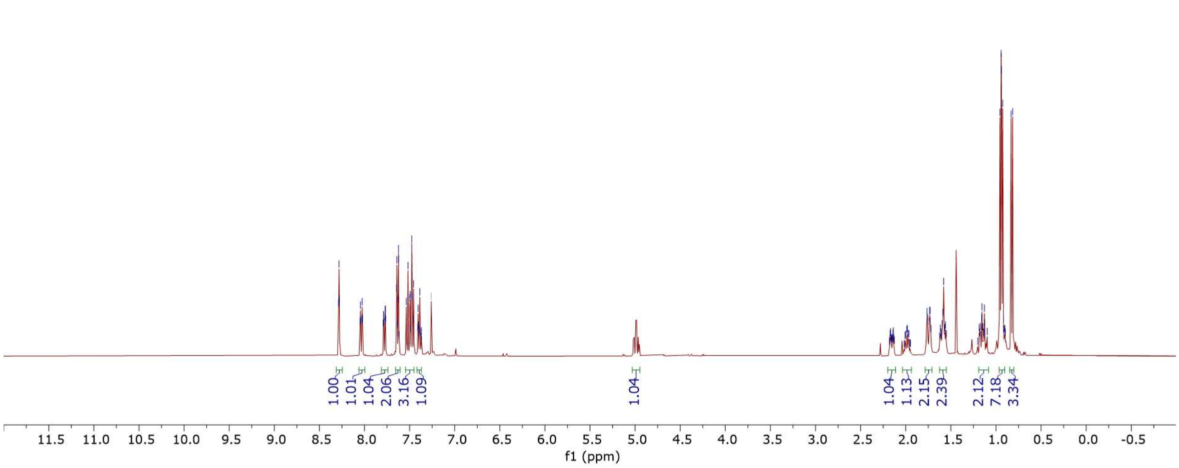

Supplement: SC-015-D4SC04413D-s001 [file SC-015-D4SC04413D-s001.pdf]
